# Epigenetic Signatures in an Italian Cohort of Parkinson’s Disease Patients from Sicily

**DOI:** 10.3390/brainsci16010031

**Published:** 2025-12-25

**Authors:** Maria Grazia Salluzzo, Francesca Ferraresi, Luca Marcolungo, Chiara Pirazzini, Katarzyna Malgorzata Kwiatkowska, Daniele Dall’Olio, Gastone Castellani, Claudia Sala, Elisa Zago, Davide Gentilini, Francesca A. Schillaci, Michele Salemi, Giuseppe Lanza, Raffaele Ferri, Paolo Garagnani

**Affiliations:** 1 Oasi Research Institute—IRCCS, 94018 Troina, Italy; msalluzzo@oasi.en.it (M.G.S.); faschillaci@oasi.en.it (F.A.S.); msalemi@oasi.en.it (M.S.); glanza@oasi.en.it (G.L.); 2 Department of Chemistry “Giacomo Ciamician”, University of Bologna, 40126 Bologna, Italy; francesca.ferraresi6@unibo.it; 3 Personal Genomics S.r.l., 37136 Verona, Italy; luca.marcolungo@personalgenomics.it (L.M.); elisa.zago@personalgenomics.it (E.Z.); 4 Department of Medical and Surgical Sciences (DIMEC), University of Bologna, 40126 Bologna, Italy; chiara.pirazzini5@unibo.it (C.P.); katarzyn.kwiatkowsk2@unibo.it (K.M.K.); daniele.dallolio@unibo.it (D.D.); gastone.castellani@unibo.it (G.C.); claudia.sala3@unibo.it (C.S.); paolo.garagnani2@unibo.it (P.G.); 5 IRCCS Azienda Ospedaliero-Universitaria di Bologna, 40138 Bologna, Italy; 6 Department of Brain and Behavioral Sciences, University of Pavia, 27100 Pavia, Italy; davide.gentilini@unipv.it; 7 Bioinformatics and Statistical Genomics Unit, Istituto Auxologico Italiano IRCCS, 20095 Cusano Milanino, Italy; 8 Department of Surgery and Medical-Surgical Specialties, University of Catania, 95124 Catania, Italy

**Keywords:** Parkinson’s disease (PD), DNA methylation, epigenomic association study (EWAS)

## Abstract

**Background/Objectives**: Parkinson’s disease (PD) is an adult-onset neurodegenerative disorder whose pathogenesis is still not completely understood. Several lines of evidence suggest that alterations in epigenetic architecture may contribute to the development of this condition. Here, we present a pilot DNA methylation study from peripheral blood in a cohort of Sicilian PD patients and matched controls. Peripheral tissue analysis has previously been shown to reflect molecular and functional profiles relevant to neurological diseases, supporting their validity as a proxy for studying brain-related epigenetic mechanisms. **Methods**: We analyzed 20 PD patients and 20 healthy controls (19 males and 21 females overall), matched for sex, with an age range of 60–87 years (mean 72.3 years). Peripheral blood DNA was extracted and processed using the Illumina Infinium MethylationEPIC v2.0 BeadChip, which interrogates over 935,000 CpG sites across the genome, including promoters, enhancers, CpG islands, and other regulatory elements. The assay relies on sodium bisulfite conversion of DNA to detect methylation status at single-base resolution. **Results**: Epigenome-wide association study (EWAS) data allowed for multiple levels of analysis, including immune cell-type deconvolution, estimation of biological age (epigenetic clocks), quantification of stochastic epigenetic mutations (SEMs) as a measure of epigenomic stability, and differential methylation profiling. Immune cell-type inference revealed an increased but not significant proportion of monocytes in PD patients, consistent with previous reports. In contrast, epigenetic clock analysis did not reveal significant differences in biological age acceleration between cases and controls, partially at odds with earlier studies—likely due to the limited sample size. SEMs burden did not differ significantly between groups. Epivariations reveal genes involved in pathways known to be altered in dopaminergic neuron dysfunction and α-synuclein toxicity. Differential methylation analysis, however, yielded 167 CpG sites, of which 55 were located within genes, corresponding to 54 unique loci. Gene Ontology enrichment analysis highlighted significant overrepresentation of pathways with neurological relevance, including regulation of synapse structure and activity, axonogenesis, neuron migration, and synapse organization. Notably, alterations in *KIAA0319*, a gene involved in neuronal migration, synaptic formation, and cortical development, have previously been associated with Parkinson’s disease at the gene expression level, while methylation changes in *FAM50B* have been reported in neurotoxic and cognitive contexts; our data suggest, for the first time, a potential epigenetic involvement of both genes in Parkinson’s disease. **Conclusions**: This pilot study on a Sicilian population provides further evidence that DNA methylation profiling can yield valuable molecular insights into PD. Despite the small sample size, our results confirm previously reported findings and highlight biological pathways relevant to neuronal structure and function that may contribute to disease pathogenesis. These data support the potential of epigenetic profiling of peripheral blood as a tool to advance the understanding of PD and generate hypotheses for future large-scale studies.

## 1. Introduction

Parkinson’s disease (PD) is a neurodegenerative disorder characterized by the pathologic loss of dopaminergic neurons in the substantia nigra and the accumulation of misfolded α-synuclein aggregates in Lewy bodies. This neuronal damage leads to motor symptoms, such as tremor and rigidity, which may evolve into complex motor fluctuations and dyskinesias, as well as autonomic dysfunctions. In addition, non-motor symptoms, including depression and cognitive decline, are commonly observed, particularly in advanced Parkinson’s disease (APD) [1].

Parkinson’s disease progresses slowly but consistently. Recent data indicate that patients with disease onset before the age of 70 have a median survival of approximately 18 years, substantially longer than the ~9 years reported in the pre-levodopa era [2]. Despite this increased longevity, quality of life and independence typically decline gradually, imposing a significant economic and social burden on healthcare systems and patients’ families [3].

Although several genetic and environmental risk factors have been identified, their interaction is increasingly recognized as a critical determinant of susceptibility to Parkinson’s disease [4].

At the intersection of genetics and environment lies epigenetics. In recent years, DNA methylation (DNAm) studies and several epigenome-wide association studies (EWASs) have been applied across a broad range of diseases, including Parkinson’s disease, to identify potential biomarkers for clinical use and uncover novel pathogenic mechanisms.

EWASs performed on post-mortem brain tissue have provided direct evidence of epigenetic alterations in neurons and regions affected by neurodegeneration, supporting a functional role for epigenetic mechanism in PD pathogenesis [5].

DNA methylation studies performed on whole blood or isolated peripheral cell populations offer a less invasive approach with the potential to identify epigenetic biomarkers relevant at different stages of Parkinson’s disease. Recent DNA methylation studies using Illumina-based arrays have identified DNAm signatures associated with cognitive decline and clinical progression in PD [6,7].

However, substantial heterogeneity across studies in terms of analytical methods, tissue type, and cohort size complicates reproducibility and hinders the identification of robust biomarkers or genes whose epigenetic modulation can be linked to the disease. Furthermore, according to Giuliani et al. [8], epigenetic and, in particular, DNA methylation variability is influenced by population-specific characteristics, indicating that a portion of variability observed in case–control EWASs may reflect the geographic origin of the analyzed cohort.

These limitations underscore the importance of new analyses in small, but methodologically robust, cohorts to identify signals that are both replicable and biologically coherent with PD pathophysiology.

In this context, the present pilot study aimed to explore genome-wide DNA methylation patterns in Parkinson’s disease in a cohort of Sicilian patients. We employed a multi-faceted analytical approach, integrating group- and individual-level assessments, to identify potential epigenetic signatures associated with the disorder, or to confirm previous results reported in the literature.

## 2. Materials and Methods

### 2.1. Patients’ Enrolment

This pilot study investigated genome-wide DNA methylation patterns in a cohort of 40 subjects, including 20 PD patients all diagnosed according to the latest diagnostic criteria for PD [9], and 20 healthy controls (CTRL). All the participants were Caucasian and of Sicilian ancestry and were recruited from the Oasi Research Institute–IRCCS in Troina (Italy). Overall, 9 patients exhibited an akinetic-rigid phenotype, and 12 were tremor-dominant. At the time of examination, 10 patients were drug-naive, while the others were being treated with one or more anti-parkinsonian drugs. Given the limited sample size, subgroup analyses by motor phenotype or medication status were not performed. Informed consent was obtained from all enrolled patients or, if needed, from their relatives. The Ethics Committee of the Oasi Research Institute–IRCCS in Troina (Italy) approved the protocol on 5 April 2022 (approval code: 2022/04/05/CE-IRCCS-OASI/52), and the study was carried out according to the Declaration of Helsinki in 1964 and its later amendments.

### 2.2. DNA Extraction and Methylation Assay

DNA was extracted from the buffy coat fraction obtained from peripheral blood collected in EDTA tubes, following the cost-effective and safe protocol of Lahiri and Nurnberger [10]. Genomic DNA was bisulfite-converted using the EZ-96 Deep Well DNA Methylation Kit (Zymo Research, Irvine, CA, USA) and analyzed using the Illumina HumanMethylationEPICv2 BeadChip (Illumina Inc., San Diego, CA, USA), according to the respective manufacturer’s instructions. Accurate randomization of the samples and phenotypic groups was performed for all processing steps.

### 2.3. Data Preprocessing

Raw IDAT files were processed in R version 4.3.1 (R Foundation for Statistical Computing, Vienna, Austria) using the sesame package for normalization and generation of β-values. Quality control included assessment of detection *p*-values (threshold 0.05), removal of probes with more than 10% unreliable values, and exclusion of samples with more than 5% failed probes. Probes associated with SNPs, cross-hybridization, and sex chromosomes were filtered out using the rmSNPandCH function from the DMRcate package v2.16.1 (Bioconductor project, R Foundation for Statistical Computing, Vienna, Austria). Exploratory analyses, including density plots, multidimensional scaling (MDS), and inter-sample distance heatmaps, were performed using ggplot2 and limma.

### 2.4. DNAm-Based Cell Count Estimation

The cell-type composition was estimated from the beta values using the IDOL algorithm with the FlowSorted.BloodExtended.EPIC R package v0.99.0, as described in the peripheral blood immune profiling deconvolution method [11]. Twelve immune cell populations were quantified, including neutrophils, eosinophils, basophils, monocytes, naïve and memory B cells, naïve and memory CD4^+^ and CD8^+^ T cells, natural killer cells, and regulatory T cells. Data normality was assessed with the Shapiro–Wilk test, and group differences were evaluated using either a Student’s *t*-test or Wilcoxon rank-sum test as appropriate. *p*-values were adjusted for multiple testing across the 12 immune cell populations using the Benjamini–Hochberg False Discovery Rate (FDR) procedure.

### 2.5. Epigenetic Estimates Analysis

Epigenetic age was estimated using established DNA methylation clocks, including Horvath, Hannum, PhenoAge, and GrimAge, which predict biological age from specific sets of CpG sites through an open-access tool available to https://dnamage.clockfoundation.org/ (accessed on 1 June 2025) [12]. Other DNA methylation–based biomarkers, such as AltumAge [13], DunedinPoAm [14], DunedinPACE [15], PC clock [16], RetroAge [17], and epigenetic scores, as well as EpiScores [18], EpiTOC [19], and DNAm-CRP [20] were also computed; these do not estimate chronological age but rather reflect biological processes or the molecular pace of aging, and therefore no classical acceleration metric was derived for them.

Epigenetic age acceleration was computed by comparing control and Parkinson’s disease groups: first, a linear regression model was fitted within the control group, and the obtained model was then applied to the entire cohort to compute age-adjusted residuals (reflecting epigenetic acceleration). Group comparisons of residuals were performed using Student’s *t*-test, and nominal *p*-values were adjusted for multiple testing using the Benjamini–Hochberg procedure.

### 2.6. Epigenetic Burden

Stochastic epigenetic mutations, a measure of entropy proposed by Gentilini et al. [21], are defined based on the CTRL population range for each CpG as [Q1 − 3 × IQR; Q3 + 3 × IQR], where IQR is the interquartile range (Q3 − Q1), with Q1—25th percentile and Q3—75th percentile.

SEMs consist of an individual value that quantifies the number of CpGs that deviate from the established reference range for each subject. After the implementation of a three-standard deviation outlier test, a linear regression model adjusted for age, sex, and cell count was employed to investigate any differences between the two groups.

The following analysis involves the enrichment of SEMs, known as epivariations, to investigate genes particularly susceptible to SEMs. Epivariated genes were analyzed by ORA using WebGestalt 2019 (WEB-based GEne SeT AnaLysis Toolkit, Baylor College of Medicine, Houston, TX, USA) for GO Biological Process; significance was tested by the hypergeometric test with FDR.

### 2.7. Differential Methylation Analysis

After filtering and removing CpGs with missing values, probes on sex chromosomes, or known polymorphisms, bi- or tri-modal CpGs were excluded using DBSCAN R package v1.1.11 (CRAN, R Foundation for Statistical Computing, Vienna, Austria). Differential methylation position analysis (DMP) with limma and Bacon adjustment identified multiple CpGs significantly associated with the group of interest. QQ plots and the genomic inflation factor (λ) were generated to evaluate the distribution of test statistics. Genes involved in DMPs were analyzed by ORA using WebGestalt for GO Biological Process and KEGG pathway enrichment; significance was tested by the hypergeometric test with FDR. Differentially methylated regions (DMRs) were detected using comb-p (Python, Andrew Jaffe Lab, GitHub, USA). The acf.py script from the comb-p repository (combined-pvalues-master) was executed using Python v3.7.6 in a conda environment. Results were annotated for genomic and regulatory region, providing a foundation for functional interpretation of the observed methylation changes.

## 3. Results

### 3.1. Cohort Description

The study cohort included 40 subjects: 20 patients with Parkinson’s disease (PD) and 20 healthy controls (CTRL), all recruited at the Oasi Research Institute–IRCCS.

The PD group consisted of 10 males and 10 females, with a mean age of 73.1 ± 7.3 years, while the CTRL group had a mean age of 73.2 ± 7.4 years. No significant age difference was observed between the two groups (*p* = 0.999).

### 3.2. Summary of Preprocessing and Filtering

Among the 40 initial samples, 39 passed quality control criteria, each showing less than 5% failed probes based on detection *p*-values. Among the 937,690 total probes, 13,799 were removed due to poor detection performance, and additional probes were excluded based on SNP overlap, cross-hybridization, and localization on sex chromosomes. Genomic inflation was evaluated using QQ plots and the Bacon correction applied to both limma and CATE models. The genomic inflation factors were λ = 1.156 for Limma+Bacon and λ = 1.15 for CATE+Bacon, indicating mild inflation. Given the comparable results, downstream analyses focused on Limma+Bacon results. *p*-values were FDR-corrected using the BH procedure, resulting in 1272 significant CpGs (Limma+Bacon) at FDR < 0.05. However, the majority of these CpGs showed small effect sizes (Δβ < 0.05) and were therefore not further interpreted. Downstream analyses focused on CpGs meeting more stringent criteria for both statistical significance and effect size.

The final dataset included 903,690 high-quality probes across 39 samples. Density plots revealed the expected bimodal distribution of β-values, while MDS and distance heatmap analyses confirmed sample homogeneity and the absence of significant outliers.

This preprocessing step ensured that technical variability was minimized and that the downstream differential methylation analysis reflected true biological variation rather than array noise.

### 3.3. Immune Cell Population

No significant differences were observed between CTRL and PD groups in the estimated cell populations (Figure S1), with 95% confidence intervals including zero for the mean differences. Monocyte proportions showed a trend toward higher levels in the PD group (unadjusted *p* = 0.012, FDR-adjusted q = 0.141, 95% CI: 0.0045–0.0275), but the effect remains uncertain. These results suggest that the methylation differences identified in subsequent analyses are unlikely to be driven by major differences in blood cell composition.

### 3.4. Epigenetic Clocks and DNAm-Based Biomarkers

**Figure 1:**
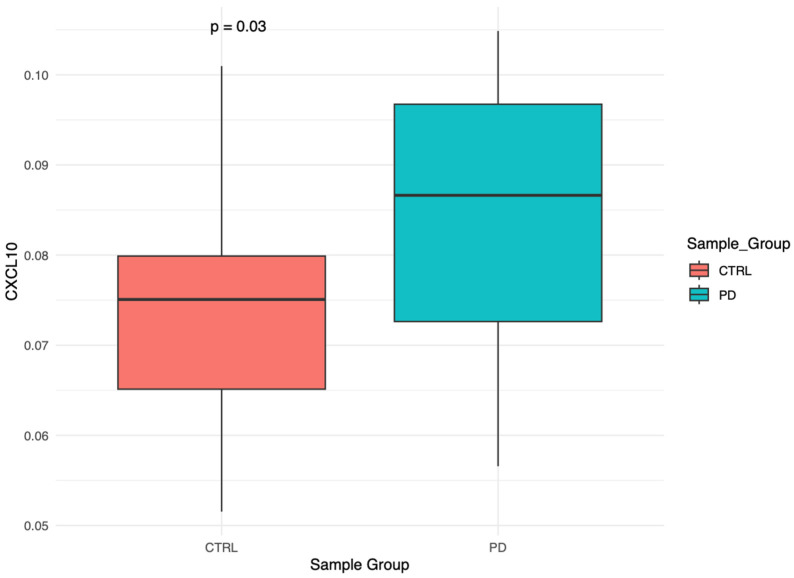
DNAm-based biomarker CXCL10: Comparison of CXCL10 levels between CTRL and PD groups. The *p*-value was calculated as described in Section 2.5.

No statistically significant differences were observed between CTRL and PD groups in epigenetic age acceleration or in any of the DNAm-based biomarkers, with 95% confidence intervals encompassing zero for all mean differences (Figures S2–S4), suggesting that overall biological aging is not accelerated in PD within this cohort. However, among the 116 EpiScores, only CXCL10 showed a nominally significant difference (*p* = 0.03 95% CI: 0.001–0.0188; Figure 1), but this did not remain significant after FDR adjustment, highlighting the uncertainty of small effect sizes.

### 3.5. Stochastic Epigenetic Mutations and Epivariations

The observed range of SEMs was from a minimum of 1232 in a CTRL to a maximum of 29,023 in a CTRL. The linear regression model revealed no statistically significant differences in the SEMs between the groups (*p* = 0.7), even when the SEMs were categorized as hypo- and hyper-methylated (Figure S5). The 95% confidence interval for the PD effect encompassed both negative and positive values, indicating that small to moderate effects cannot be excluded.

Nevertheless, the epivariations analysis revealed 19 genes with SEMs enrichment, all hypomethylated. Among the 14 genes that present epivariations in PD subjects only (Table S1), the GO analysis (Table 1) indicated some pathway linked to PD such as Golgi vesicles transport, membrane fission, fatty acid derivative metabolic process, membrane docking, lipoprotein metabolic process, and nucleus organization, pathways known to be altered in dopaminergic neuron dysfunction and α-synuclein toxicity. However, none of these pathways remained significant after correction for multiple testing (all FDR = 1), and therefore these findings should be considered exploratory and hypothesis-generating.

### 3.6. Differentially Methylated Positions (DMPs)

**Figure 2:**
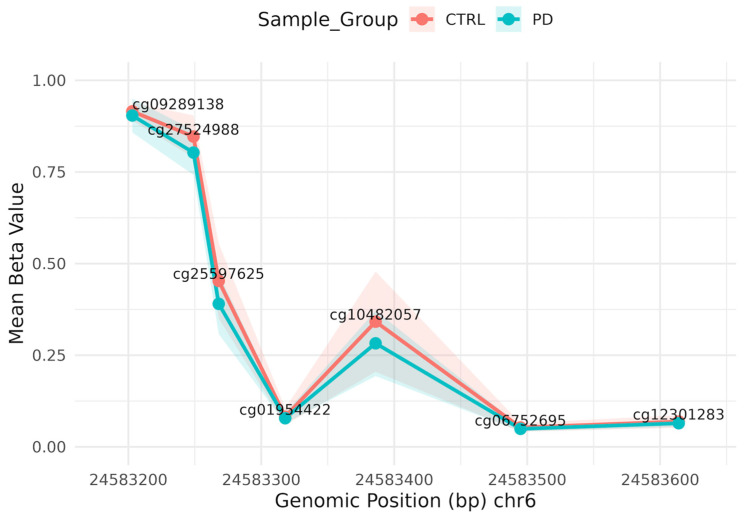
DMP cg2559762 plot: Line plot showing DNA methylation of six CpGs near the DMP cg25597625 (two upstream and four downstream). The x-axis represents genomic position on chromosome 6 (hg38), while the *y*-axis shows the mean of the DNA methylation beta values.

Two CpGs were identified as DMPs using the most stringent filter (adjusted *p*-value < 0.001, controlling for multiple testing, and Δβ > 0.1, representing the absolute difference in DNA methylation between groups). One of these CpGs, cg25597625 (Figure 2), is located in a genic region and is associated with the *KIAA0319* gene, which is involved in the development of the cerebral cortex.

Using a less stringent threshold (adjusted *p*-value < 0.05 and Δβ > 0.05), 167 DMPs were identified between CTRL and PD, with 54 CpGs annotated to unique genes. A heatmap of these CpGs (Figure S6) shows a tendency for PD subjects to cluster together. Gene Ontology analysis highlighted biologically relevant processes, including regulation of synapse structure and activity, axogenesis, neuron migration, and synapse organization (Table 2). Pathway enrichment analysis revealed significant involvement of neurodegeneration-related pathways and neuroactive ligand–receptor interaction (Table S2). Although several pathways were nominally enriched, none remained significant after FDR correction, indicating that these results should be interpreted with caution and require independent replication.

### 3.7. Differentially Methylated Regions (DMRs)

**Figure 3:**
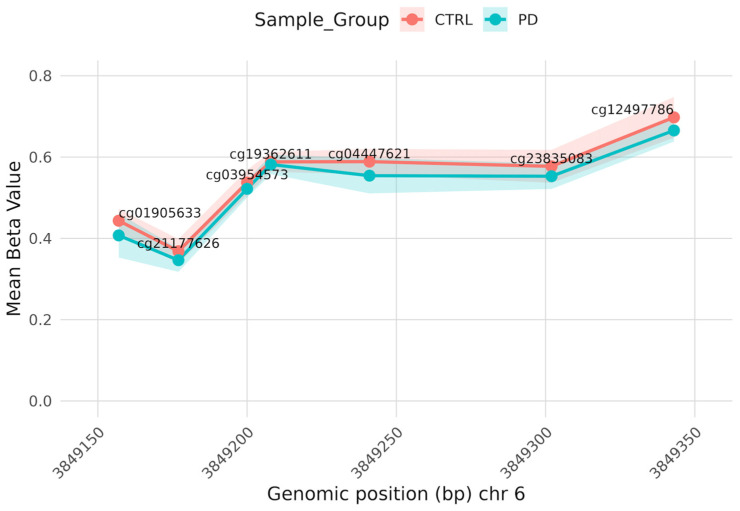
DMR in *FAM50B* gene: Line plot of the methylation profile across the DMR annotated in the *FAM50B* gene in PD and CTRL. The x-axis represents the genomic position on chromosome 6 (chr6:3849157-3849344, hg38). The y-axis represents the mean of DNA methylation beta value.

Three DMRs result with adjusted *p*-value < 0.001 and region length with at least three CpGs. Two regions are associated with unique genes: *CABP5* (Figure S7), a calcium binding protein on chromosome 19, and *FAM50B*, a functional retrotransposon on chromosome 6 (Figure 3).

The identification of DMRs in loci involved in calcium signaling and neuronal regulation reinforces the hypothesis that peripheral methylation signatures may reflect molecular events relevant to the central nervous system.

## 4. Discussion

This study investigates the methylation data from different perspective on a small cohort of Parkinson’s patients. Specifically, in the study epigenetic clocks and combined biomarkers, epimutations, and genome-wide differential methylation have been carried out comparing PD patients and a cohort of unaffected controls. While limited sample size constrains statistical power, the study provides hypothesis-generating insights into peripheral epigenetic alterations in PD.

Analysis of peripheral blood cell populations revealed no significant differences between the groups. Notably, monocytes showed a nominal but not significant increase in PD (unadjusted *p* = 0.012; FDR q = 0.141). We mention this result since the topic of monocyte altered count in PD is already present in the literature, without any conclusive result. Our finding somehow contributes to keeping the topic alive, since of all the cell populations that we can assess from the methylation profile, the monocytes are those presenting the highest difference between PD and controls. Notably, also in our results, the PD showed the highest count, as reported in previous reports [22,23].

No significant differences were observed in DNA methylation–based epigenetic clocks, and no consistent trends emerged, aligning with the heterogeneous literature on epigenetic aging in PD [24,25], where accelerated aging is reported inconsistently, suggesting minimal impact on PD neurodegeneration. However, this group of negative results could be due to the low sample size of our cohort.

Analysis of individual EpiScores revealed a nominal increase in CXCL10, a chemokine involved in immune cell recruitment, which may reflect subtle immune-related epigenetic changes consistent with neuroinflammation [26,27]. As this association did not survive multiple testing correction, it is considered exploratory. It is worthwhile to link this result with the monocyte observation, since altogether they suggest a possible specific pro-inflammatory signature linked to PD.

While stochastic epigenetic mutations (SEMs) and rare epivariations have been studied in neurological and neuropsychiatric contexts [28,29], in our cohort, we did not reveal global differences. Gene Ontology analysis of epivariated genes highlighted pathways relevant to PD, including Golgi integrity and vesicular trafficking, which are implicated in dopaminergic neuron vulnerability and α-synuclein homeostasis (Table 1). Alterations in these processes have been previously observed in neurons containing α-synuclein inclusions [30], and α-synuclein is known to modulate synaptic vesicle docking depending on the lipid environment [31], as well as to interact with lipid and lipoprotein metabolism, contributing to neuronal degeneration [32,33].

However, as GO enrichment did not survive FDR correction, these findings are interpreted as biologically plausible but exploratory and intended primarily to support hypothesis generation rather than definitive pathway-level conclusions. Similar epivariation-based approaches in other neurodegenerative diseases, such as amyotrophic lateral sclerosis, have likewise identified biologically relevant but statistically modest signals [34].

The most interesting evidence emerged from the differential methylation analysis, with a robust signal observed at cg25597625, annotated to the *KIAA0319* gene, which is involved in neuronal migration, synaptic organization, and cortical development. Our exploratory methylation results are consistent with published transcriptomic studies: RNA-sequencing from substantia nigra (GSE114517) shows significant downregulation of *KIAA0319* (FDR < 0.05) [35], and integrative PPI and LASSO analyses highlight *KIAA0319* as a candidate gene [36]. In addition, our exploratory comparison with the Simchovitz expression dataset (FDR < 0.05, |logFC| ≥ 1) identified 23 gene-associated CpGs shared between the two datasets, including *KIAA0319*, further supporting this candidate. Interestingly, the area of different methylation, peaking in cg25597625, map within the gene-body of *KIAA0319* and is hypomethylated in PD. The general rule indicates that the effect of methylation in the gene-body is opposite to that in CpG islands; thus, the highest is the methylation, and the lowest is the expression. Accordingly, this result meets the picture emerged in both the two previous studies that investigate the gene expression of substantia nigra in PD and control, where the results are concordant in indicating a downregulation of the gene in PD. In the Simchovitz dataset, the *CXCL10* gene is also differentially expressed with the same direction as observed in our EpiScores analysis, namely, up-regulated.

*KIAA0319* has also been implicated in Alzheimer’s disease through independent molecular studies. Transcriptomic analyses have reported under-expression of *KIAA0319* in Alzheimer’s brain tissue, mediating disease risk [37], and experimental studies indicate that KIAA0319 modulates astrocytic oxidative stress responses via microRNA-dependent mechanisms [38]. Furthermore, genome-wide association studies have linked variants to cognition, memory, and depression [39]. Separately, epigenetic and genetic studies in non-neurodegenerative contexts associate *KIAA0319* promoter methylation and regulatory variation with cognitive and language-related traits [40,41,42]. *KIAA0319* is also connected to cuproptosis and dopaminergic function [43] and experimentally regulates axon growth via Smad2 signaling and the neuroepithelial cell cycle [44,45].

The association, based on a single CpG site, should be considered exploratory, as independent replication and functional validation are lacking, despite being consistent with prior evidence linking *KIAA0319* to neurodevelopment.

Analysis of the most relevant DMRs identified *FAM50B*, an imprinted gene that has been reported in studies of cognitive and neurodevelopmental alterations. Notably, previous studies have reported that *FAM50B* hypomethylation correlates with reduced intellectual performance in children [46,47]. While no studies have directly linked *FAM50B* to Parkinson’s disease, its identification in our cohort suggests potential biological relevance in neurodegenerative contexts. Nevertheless, replication and functional validation are required to clarify the role in disease pathogenesis.

Overall, most pathway enrichment results and nominal associations (e.g., CXCL10 EpiScore, monocyte trends, epivariations) did not survive multiple testing and are therefore considered exploratory. Their biological relevance was assessed based on consistency with established PD mechanisms rather than statistical significance alone.

Several limitations must be acknowledged: (i) The small sample size (20 PD vs. 20 controls) limits statistical power, meaning that only relatively large DNA methylation differences are likely to be detected. Smaller but potentially biologically relevant effects may remain undetected, increasing the risk of false negatives. To reflect this uncertainty, non-significant results are reported, with 95% confidence intervals, emphasizing that lack of significance does not imply absence of effect. (ii) PD patients vary in motor subtype and treatment, which may influence methylation, but the sample size does not allow stratified analyses. (iii) Population-specific genetic and environmental factors in this Sicilian cohort may limit generalizability. (iv) Environmental and clinical factors such as tobacco exposure, chronic inflammation, infections, and long-term pharmacological treatment have been shown to influence peripheral DNA methylation and may represent important sources of residual confounding [48,49]. Replication in larger and independent cohorts will be required to confirm these results.

In conclusion, this study represents a pilot exploratory investigation of peripheral methylation in PD. Despite the exploratory nature of the study, some of the results such as those on *KIAA0319* gene are linked with biological function coherent with the pathogenesis of the disease and, maybe most importantly, already emerged in previous studies, some of which were executed directly on the substantia nigra of PD and controls. In any case, this is a cross-sectional study on diagnosed PD patients; thus, it is not possible to assess whether these interesting hints are part of the pathogenesis of the neurodegeneration or, rather, a consequence of the derangement of the alpha-synucleinopathy.

## Figures and Tables

**Table 1:** Gene Ontology (GO) enrichment analysis of epivariation loci in PD patients. The table shows the top enriched biological processes identified in the epivariated genes. Ratio indicates the observed versus expected number of genes in the pathway. *p*-value corresponds to the statistical significance of enrichment. Although the uncorrected *p*-values suggest enrichment, none of the pathways remained significant after FDR correction for multiple testing (all FDR = 1).

GO ID	Description	Ratio	*p*-Value	FDR
GO:0048193	Golgi vesicle transport	14.296	0.0078	1
GO:0090148	Membrane fission	48.412	0.0205	1
GO:1901568	Fatty acid derivative metabolic process	28.786	0.0342	1
GO:0022406	Membrane docking	23.154	0.0424	1
GO:1903509	Liposaccharide metabolic process	19.190	0.0510	1
GO:0051321	Meiotic cell cycle (nucleus organization)	14.844	0.0073	1

**Table 2:** Gene Ontology: GO enrichment for 54 genes annotated from 167 DMPs (adjusted *p*-value < 0.05 and Δβ > 0.05). Only terms with nominal *p* < 0.01 are shown. FDR values indicate none of the terms are significant after multiple testing correction.

GO ID	Description	Ratio	*p*-Value	FDR
GO:0050803	Regulation of synapse structure or activity	8.22	3.26 × 10^−4^	0.191
GO:0007409	Axonogenesis	5.35	7.85 × 10^−4^	0.191
GO:0001764	Neuron migration	8.96	9.94 × 10^−4^	0.191
GO:0050808	Synapse organization	5.06	1.05 × 10^−3^	0.191

## Data Availability

The original data presented in the study are openly available in Gene Expression Omnibus (GEO) at https://www.ncbi.nlm.nih.gov/geo/query/acc.cgi?acc=GSE312046 (accessed on 29 November 2025).

## Supplementary Materials

The following supporting information can be downloaded at https://www.mdpi.com/article/10.3390/brainsci16010031/s1. Figure S1: Boxplot of the estimated cell population across CTRL and PD. No significant differences were observed between CTRL and PD groups in the estimated cell populations. Monocyte proportions showed a trend toward higher levels in the PD group (unadjusted *p* = 0.012, FDR-adjusted q = 0.141); Figure S2: Boxplots of DNA methylation age acceleration estimates derived from multiple epigenetic clocks (Horvath DNAmAge, Hannum DNAmAge, PhenoAge, Skin & Blood Clock, Zhang clock, FitAge, and GrimAge based on predicted and real age) comparing CTRL and PD groups. For each clock, distributions are shown for CTRL and PD individuals; “ns” indicates no statistically significant between-group difference. Figure S3: Boxplots of epigenetic mitotic clock–based measures (epiTOC1, HypoClock, RepliTali, and stemTOC) comparing CTRL and PD groups. Distributions are shown for each score in CTRL and PD individuals; *p*-values indicate between-group comparisons, with no statistically significant differences observed. Figure S4: Distribution of epigenetic clock and epigenetic biomarker scores comparing CTRL and PD groups. Boxplots show group differences for multiple DNA methylation–based clocks, pace-of-aging measures, lifestyle-related scores, and protein-related EpiScores. For each panel, values are displayed for CTRL and PD individuals; statistical significance refers to between-group comparisons performed as in the main analyses. Figure S5: Boxplot of log (SEMs). Stochastic epigenetic mutations across group. *p*-value calculated from a linear regression model adjusted for age, sex, and cell count. Figure S6: Heatmap of the 54cg DMP (threshold: adjusted *p*-value < 0.05 and Δβ > 0.05) annotated in unique genes. Figure S7: Methylation profile for the DMR annotated in *CABP5* gene. The x-axis is the genomic position of chromosome 19 (chr19:48044057_48044106, hg38). The y-axis is the mean of beta value. Table S1: Epivariations: list of genes with epivariations in CTRL and PD subjects. Gene: Gene name (annotation hg38). Meth: Methylation status (Down = hypomethylated, Up = hypermethylated). CTRL and PD: number of subjects with at least one epivariation in that gene. Table S2: Pathway enrichment analysis of 54 genes annotated from 167 DMPs (Δβ > 0.05). Only pathways with nominal *p* < 0.08 are shown. None of the pathways reached significance after multiple testing correction (FDR).
