# Supplementary material for: Epigenetic Signatures in an Italian Cohort of Parkinson’s Disease Patients from Sicily
Source: Brain Sci. 2025 Dec 25;16(1):31. doi: 10.3390/brainsci16010031 (PMC12839038; doi:10.3390/brainsci16010031)
Supplement: Supplementary file 1 [file brainsci-16-00031-s001.zip › brainsci-4049051-supplementary.pdf]

Figure S1. Boxplot of the estimated cell population across CTRL and PD. No significant differences were observed between CTRL and PD groups in the estimated cell populations. Monocyte proportions showed a trend toward higher levels in the PD group (unadjusted p = 0.012, FDR-adjusted q = 0.141).

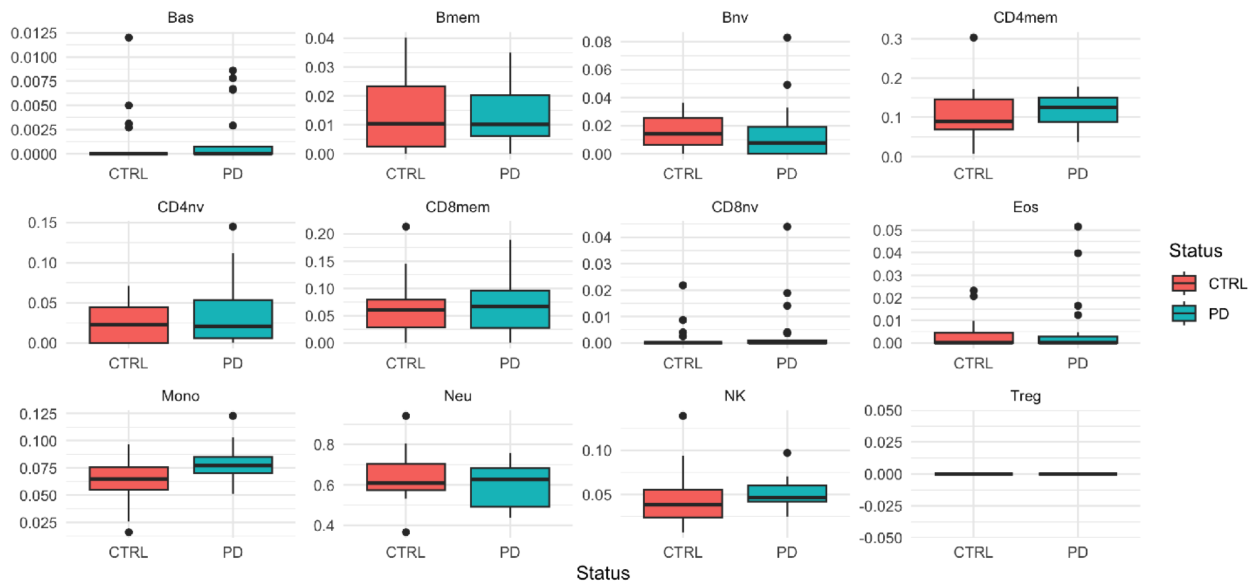

Figure S2. Boxplots of DNA methylation age acceleration estimates derived from multiple epigenetic clocks (Horvath DNAmAge, Hannum DNAmAge, PhenoAge, Skin & Blood Clock, Zhang clock, FitAge, and GrimAge based on predicted and real age) comparing CTRL and PD groups. For each clock, distributions are shown for CTRL and PD individuals; "ns" indicates no statistically significant between-group difference.

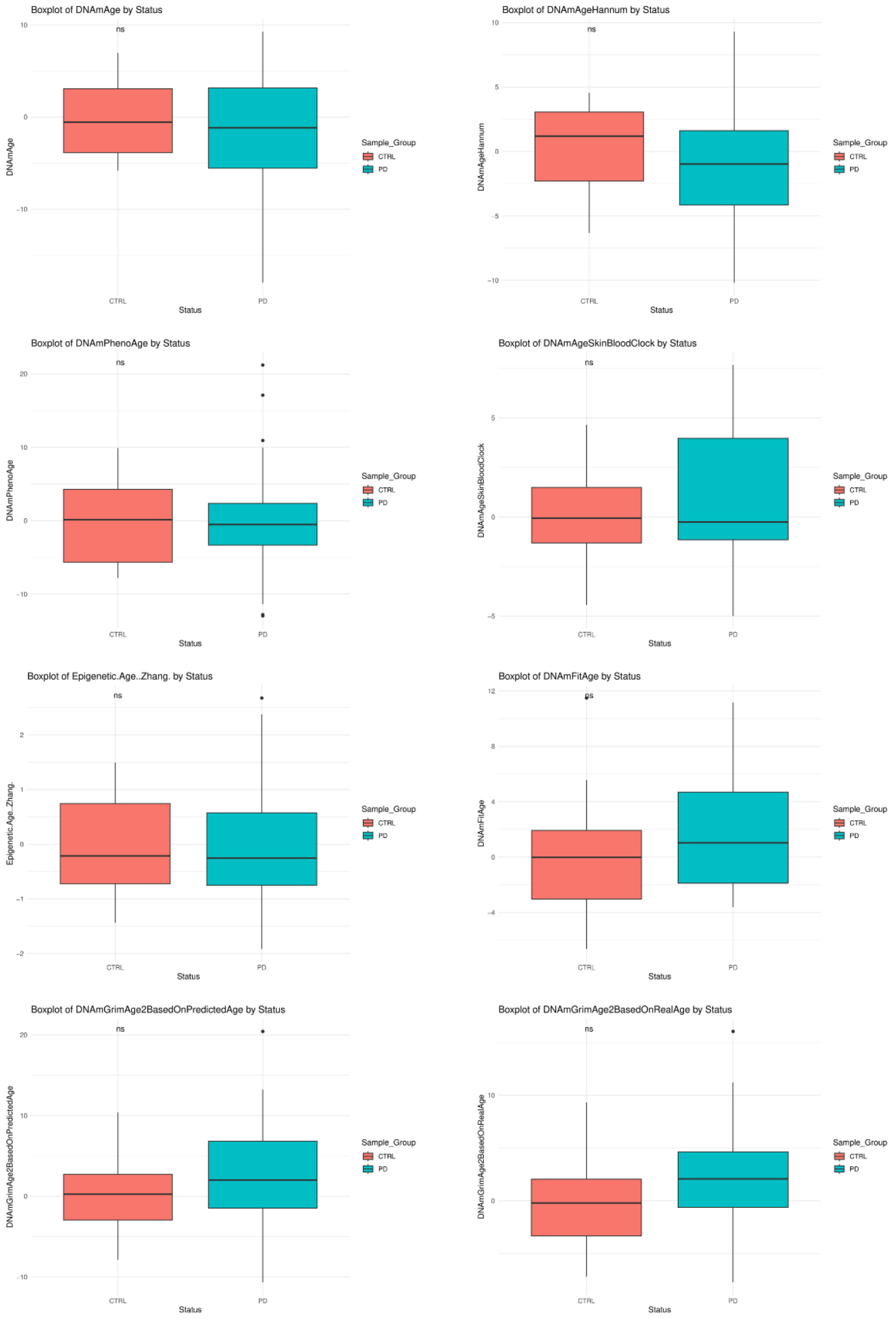

Figure S3. Boxplots of epigenetic mitotic clock–based measures (epiTOC1, HypoClock, RepliTali, and stemTOC) comparing CTRL and PD groups. Distributions are shown for each score in CTRL and PD individuals; p-values indicate between-group comparisons, with no statistically significant differences observed.

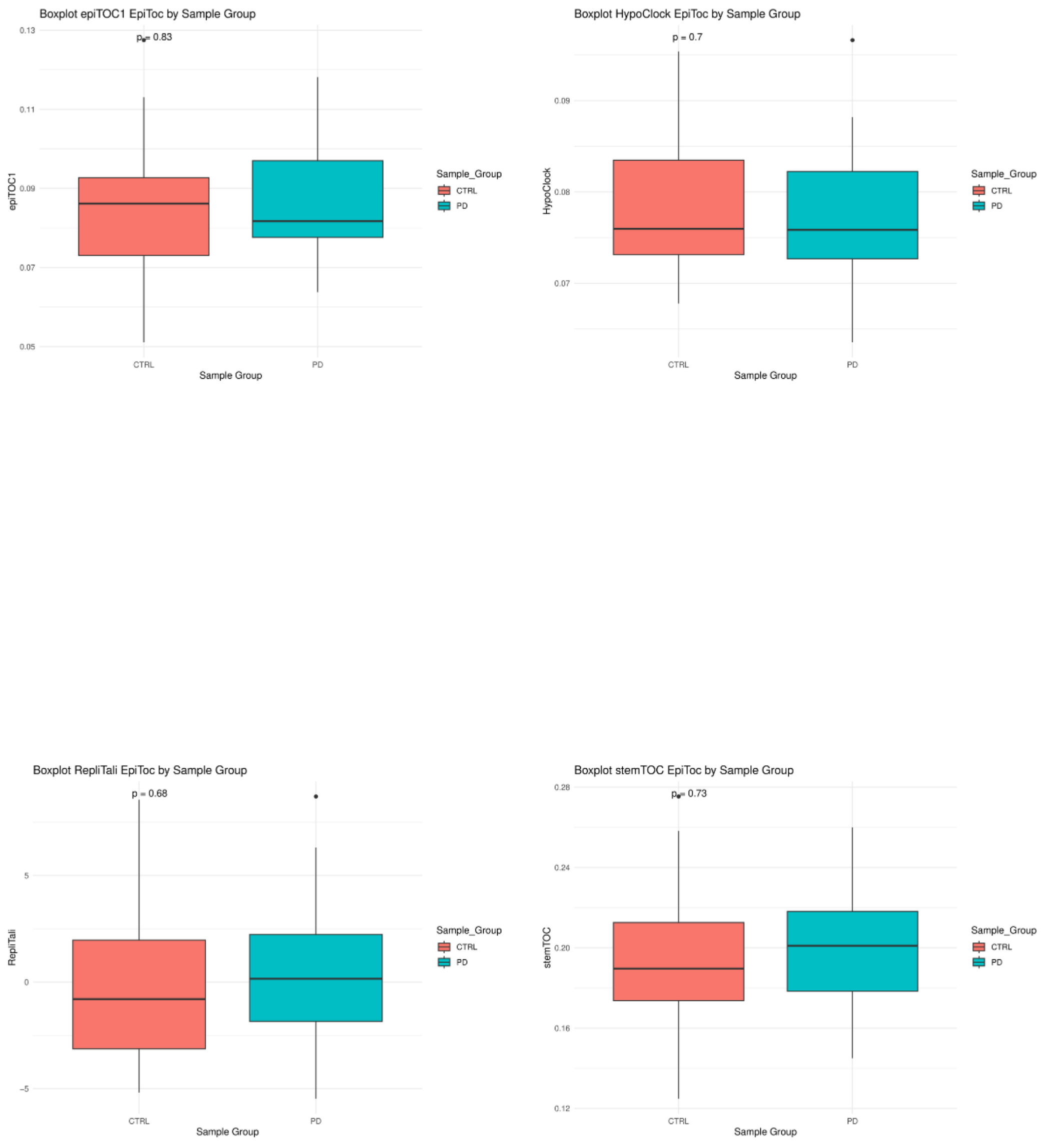

Figure S4. Distribution of epigenetic clock and epigenetic biomarker scores comparing CTRL and PD groups. Boxplots show group differences for multiple DNA methylation–based clocks, pace-of-aging measures, lifestyle-related scores, and protein-related EpiScores. For each panel, values are displayed for CTRL and PD individuals; statistical significance refers to between-group comparisons performed as in the main analyses.

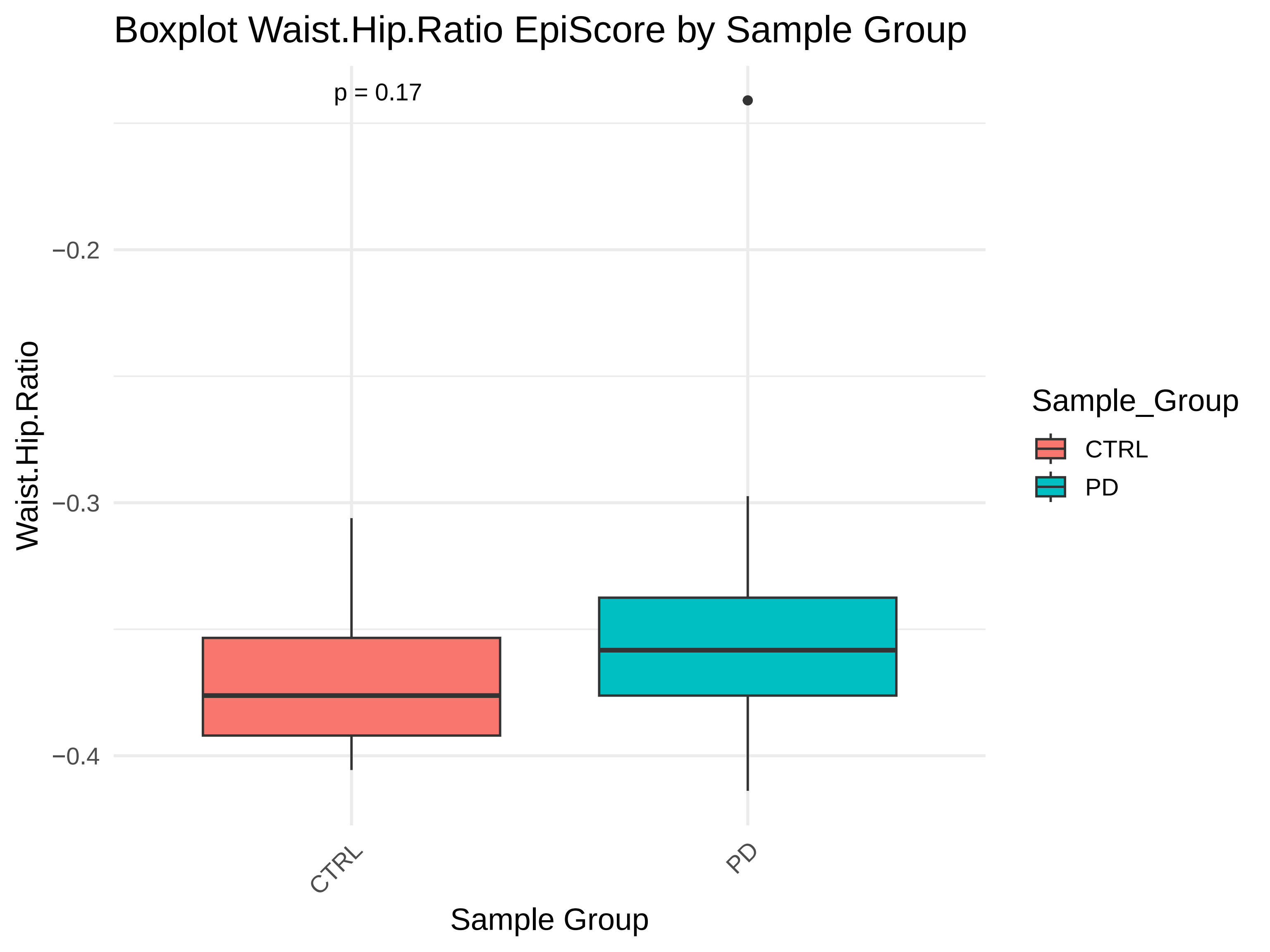

Boxplot Waist.Hip.Ratio EpiScore by Sample Group
p = 0.17
−0.2
−0.3
−0.4
Waist.Hip.Ratio
CTRL
PD
Sample Group
Sample_Group
CTRL
PD

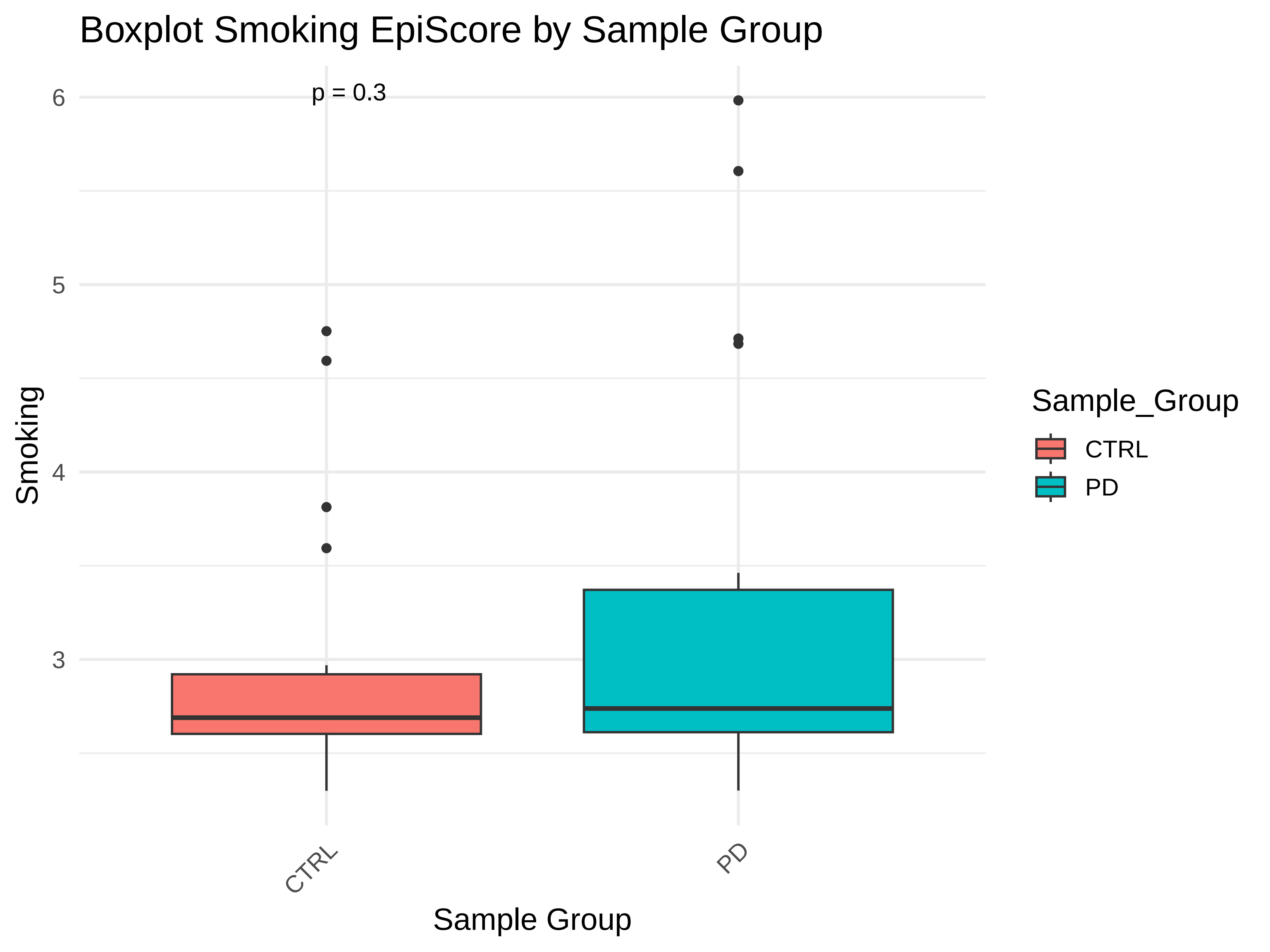

Boxplot Smoking EpiScore by Sample Group
p = 0.3
6
5
4
3
Smoking
CTRL
PD
Sample Group
Sample_Group
CTRL
PD

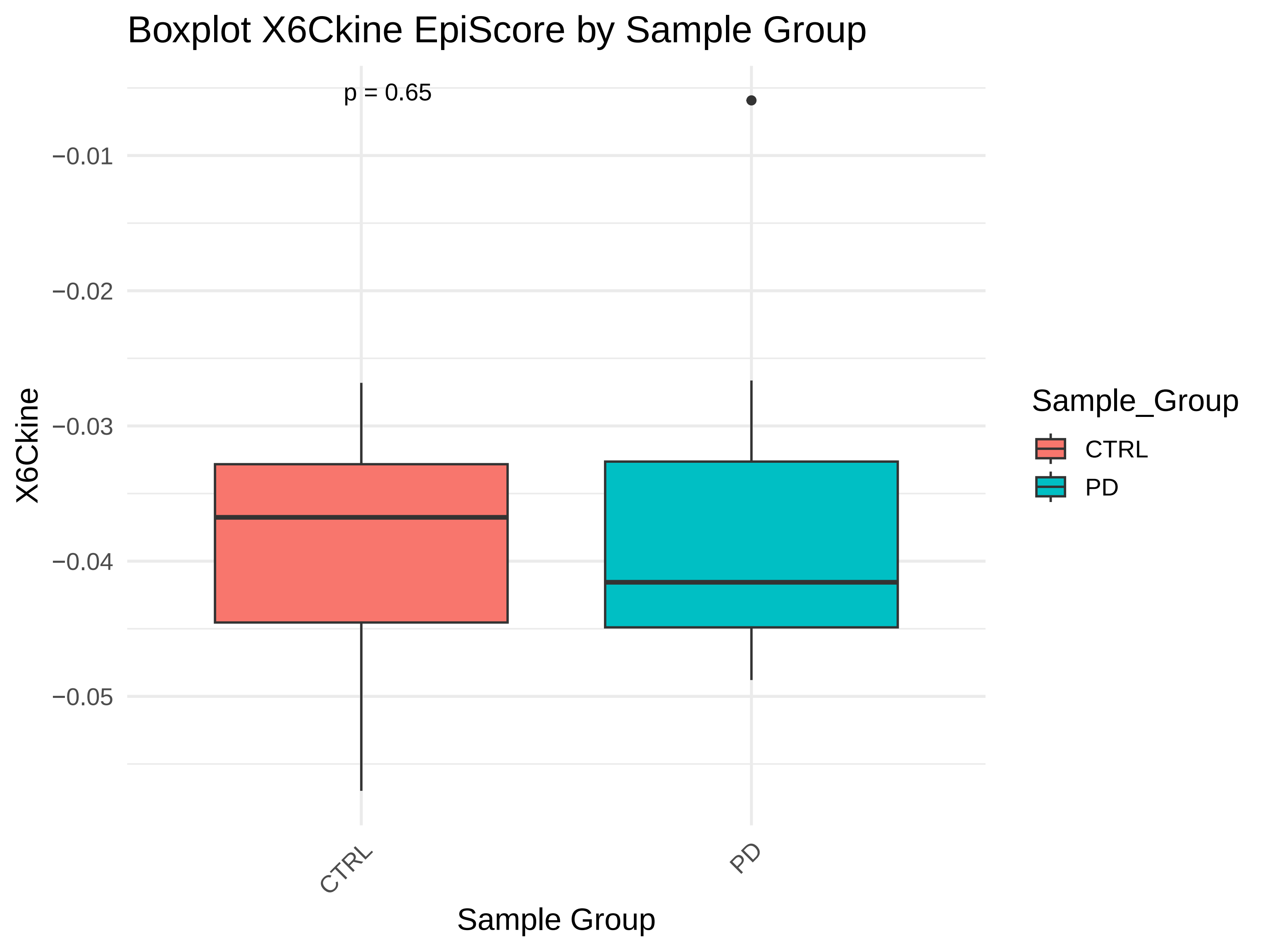

Boxplot X6Ckine EpiScore by Sample Group
p = 0.65
−0.01
−0.02
−0.03
−0.04
−0.05
X6Ckine
CTRL
PD
Sample Group
Sample_Group
CTRL
PD

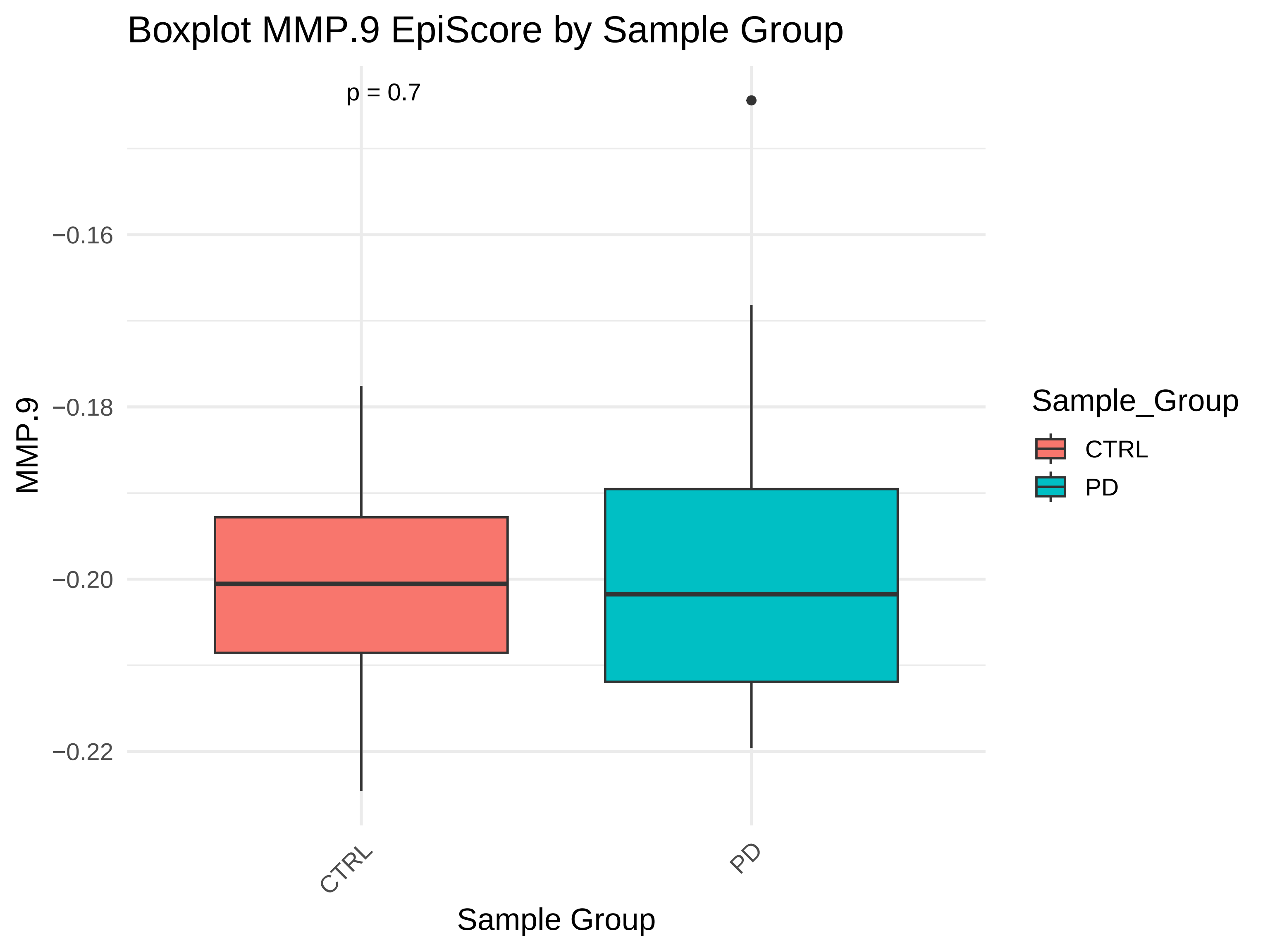
Boxplot MMP.9 EpiScore by Sample Group
p = 0.7
MMP.9
−0.16
−0.18
−0.20
−0.22
CTRL
PD
Sample Group
Sample_Group
CTRL
PD

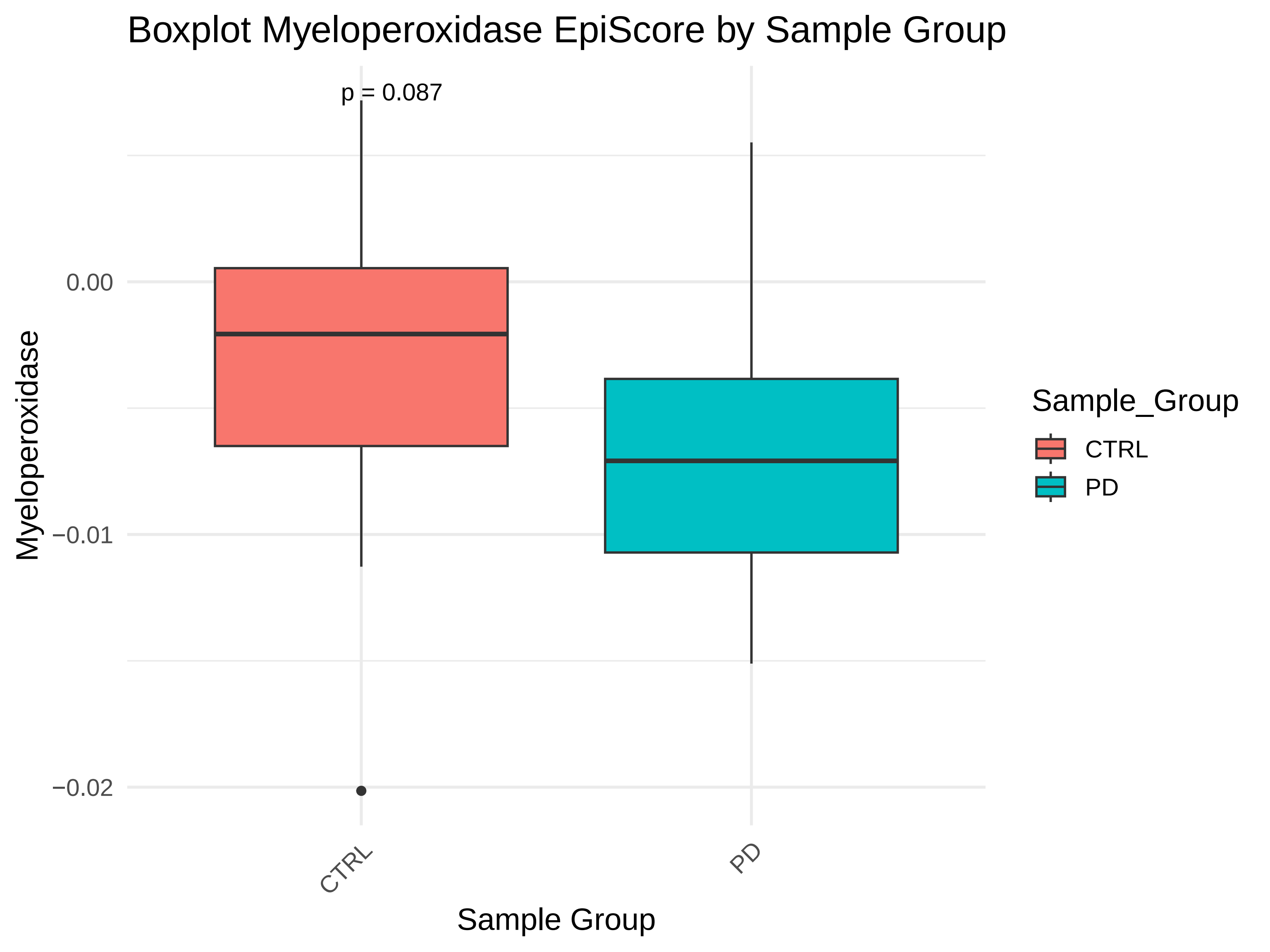
Boxplot Myeloperoxidase EpiScore by Sample Group
p = 0.087
0.00
−0.01
−0.02
Myeloperoxidase
CTRL
PD
Sample Group
Sample_Group
CTRL
PD

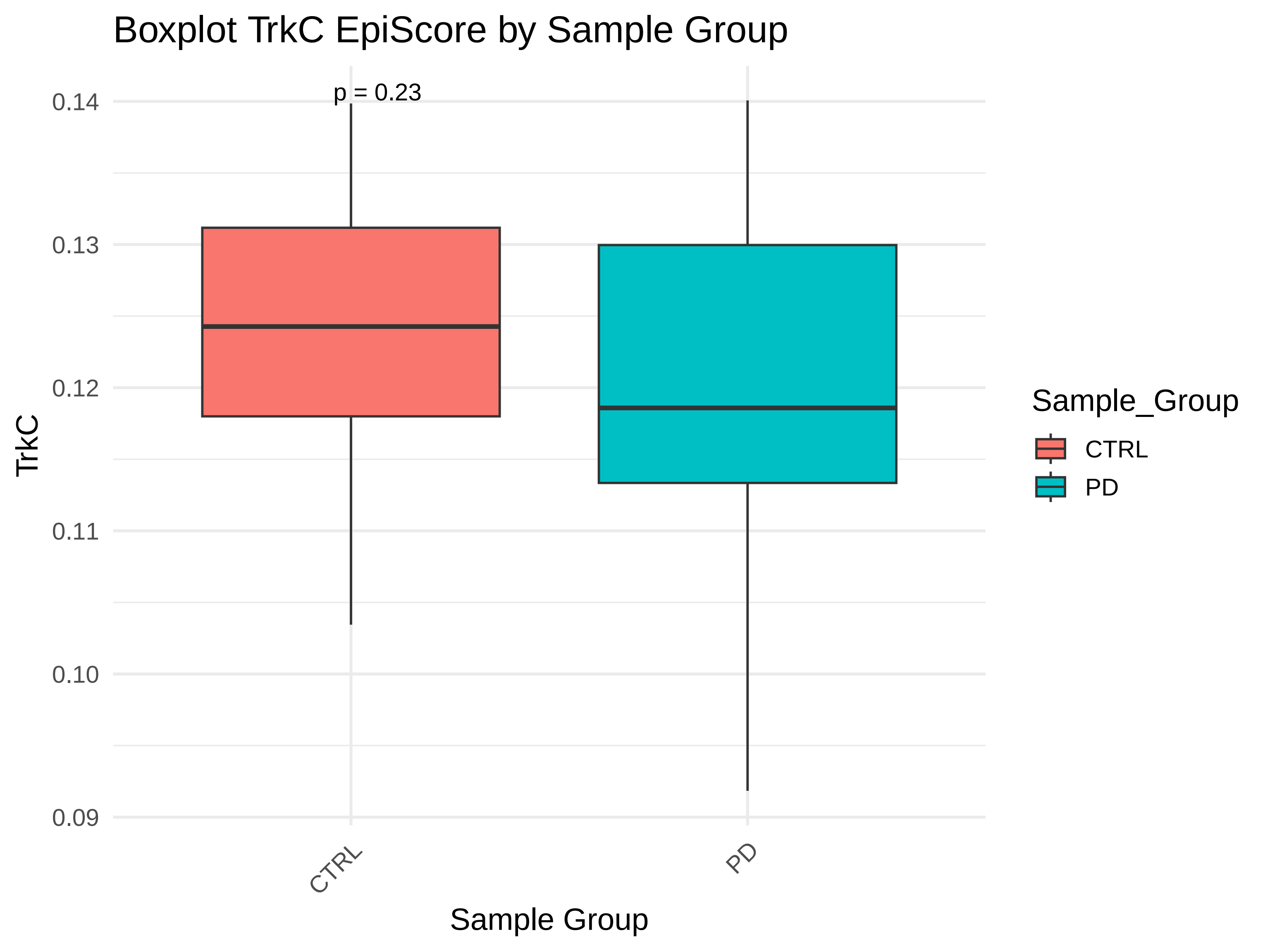
Boxplot TrkC EpiScore by Sample Group
p = 0.23
0.14
0.13
0.12
0.11
0.10
0.09
TrkC
CTRL
PD
Sample Group
Sample_Group
CTRL
PD

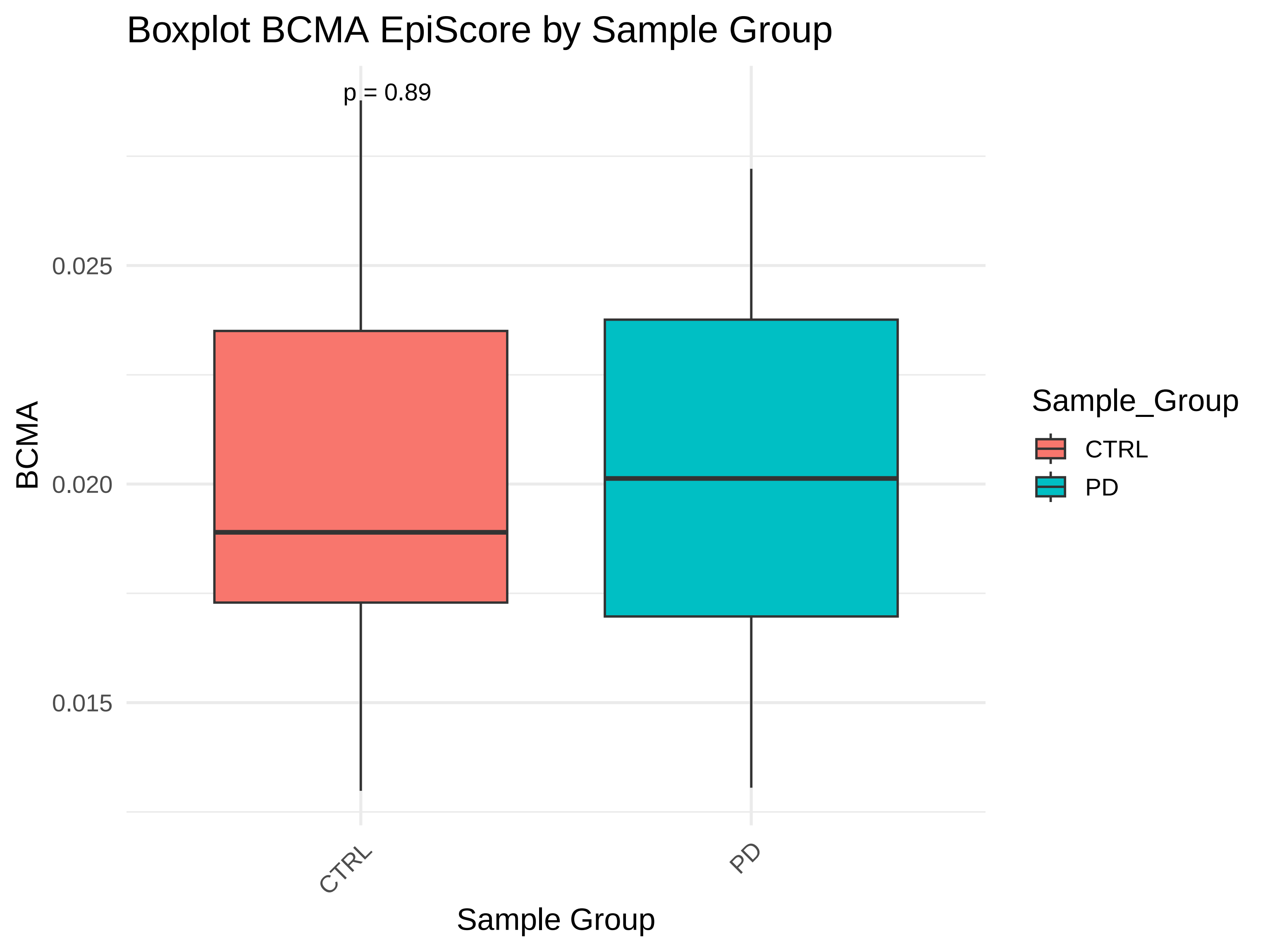
Boxplot BCMA EpiScore by Sample Group
p = 0.89
0.025
0.020
0.015
BCMA
CTRL
PD
Sample Group
Sample_Group
CTRL
PD

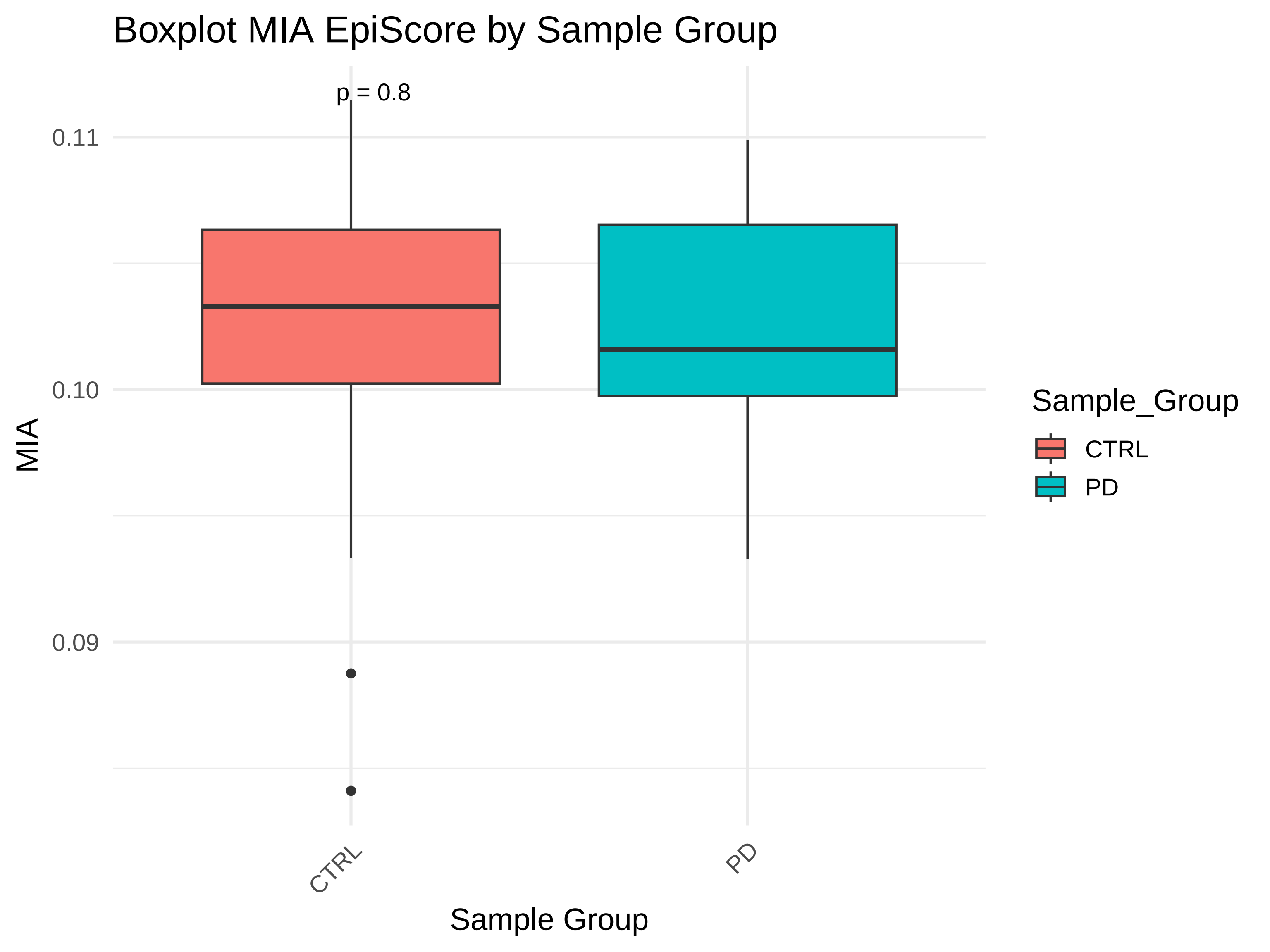
Boxplot MIA EpiScore by Sample Group
p = 0.8
0.11
0.10
0.09
MIA
CTRL
PD
Sample Group
Sample_Group
CTRL
PD

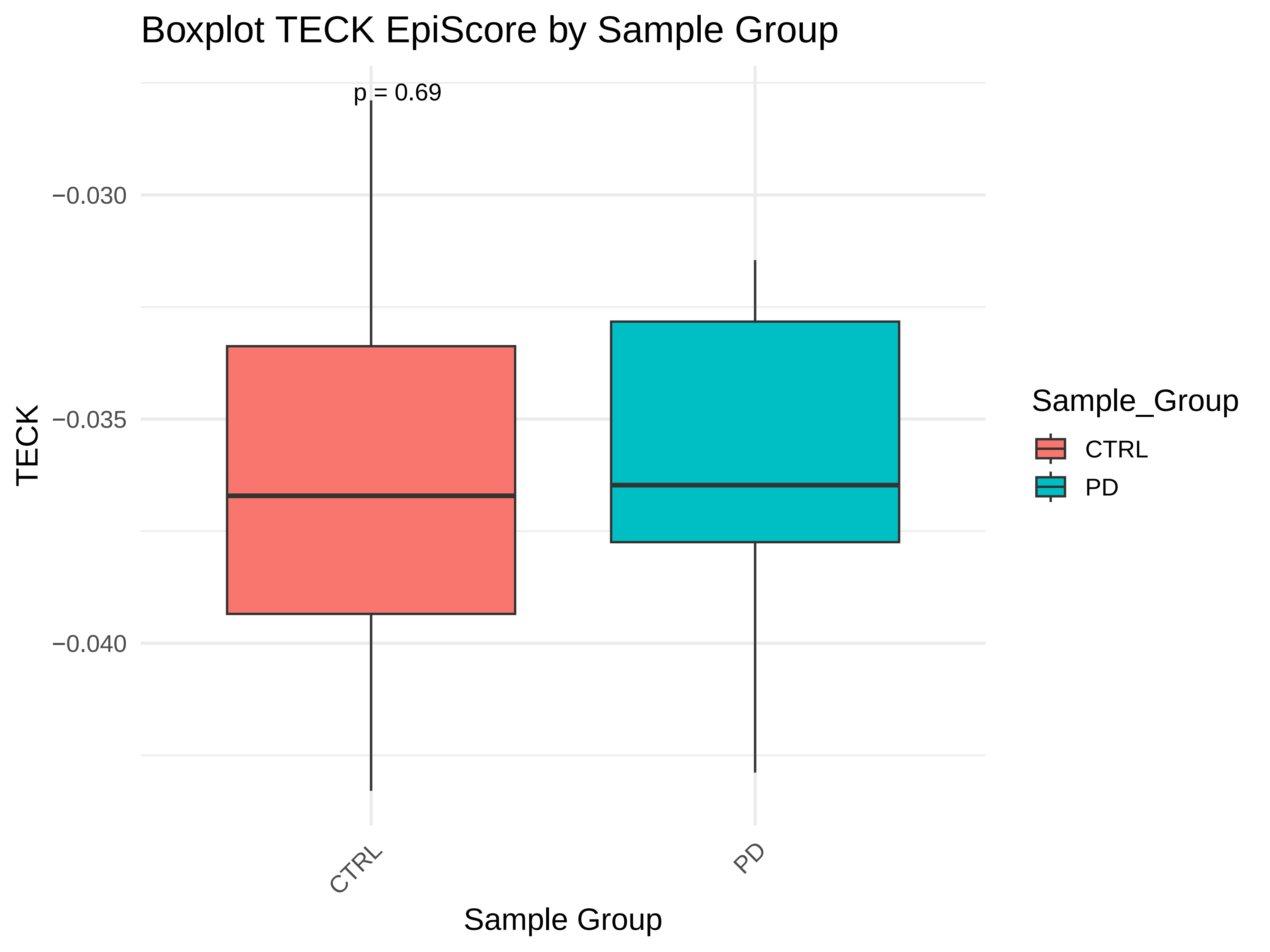

Boxplot TECK EpiScore by Sample Group
p = 0.69
−0.030
−0.035
−0.040
TECK
CTRL
PD
Sample Group
Sample_Group
CTRL
PD

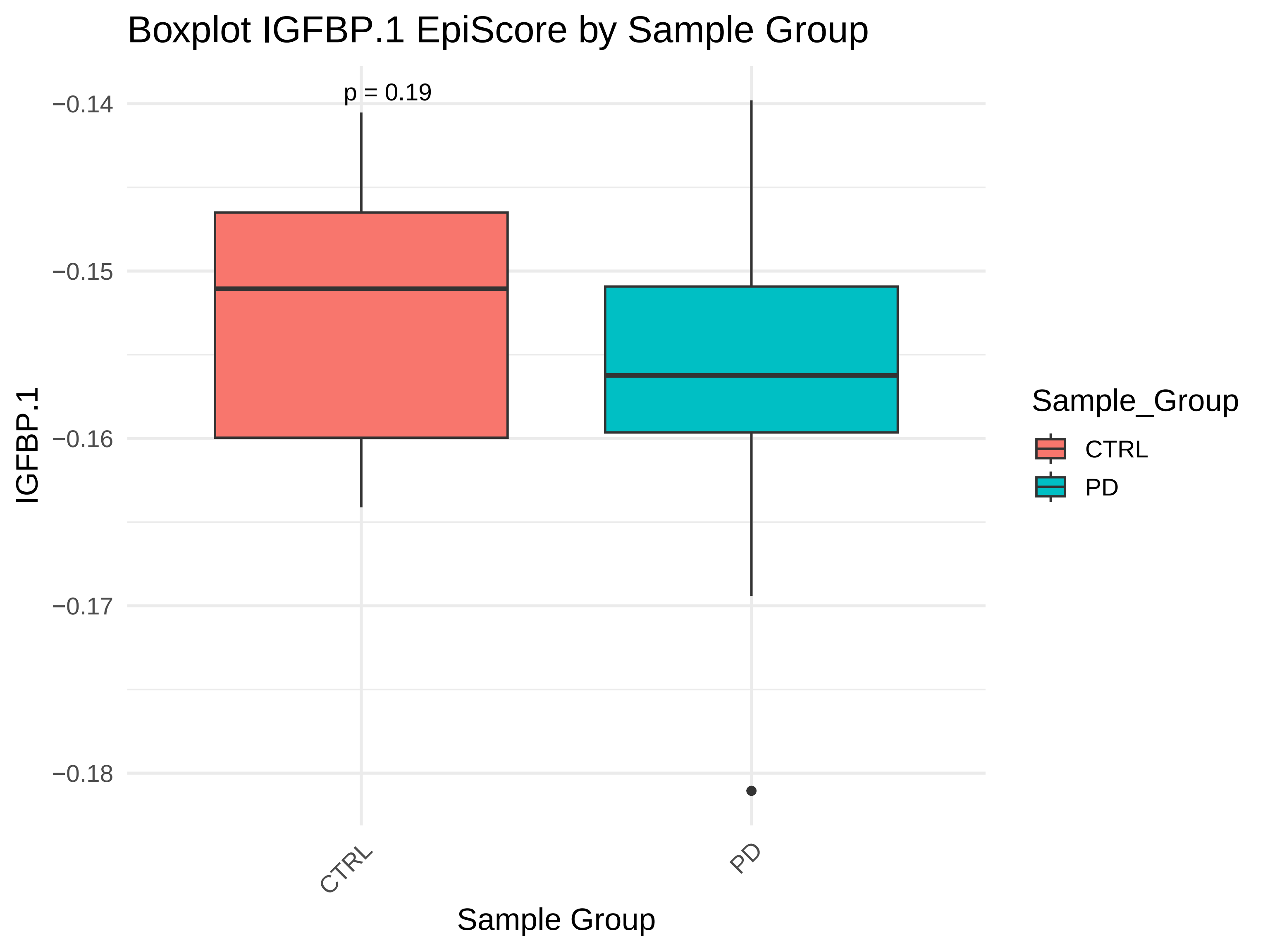
Boxplot IGFBP.1 EpiScore by Sample Group
p = 0.19
−0.14
−0.15
−0.16
−0.17
−0.18
IGFBP.1
CTRL
PD
Sample Group
Sample_Group
CTRL
PD

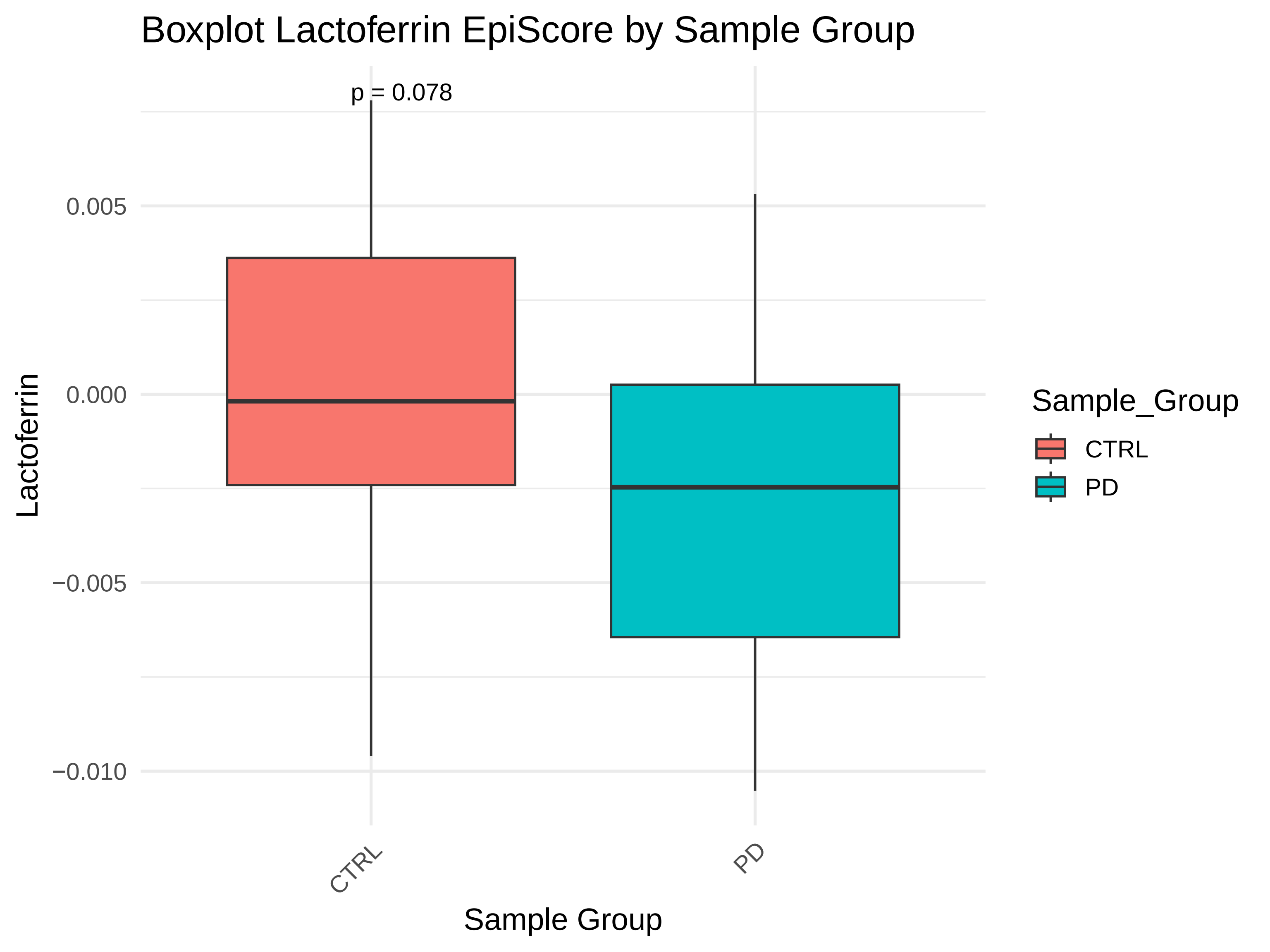
Boxplot Lactoferrin EpiScore by Sample Group
p = 0.078
0.005
0.000
−0.005
−0.010
Lactoferrin
CTRL
PD
Sample Group
Sample_Group
CTRL
PD

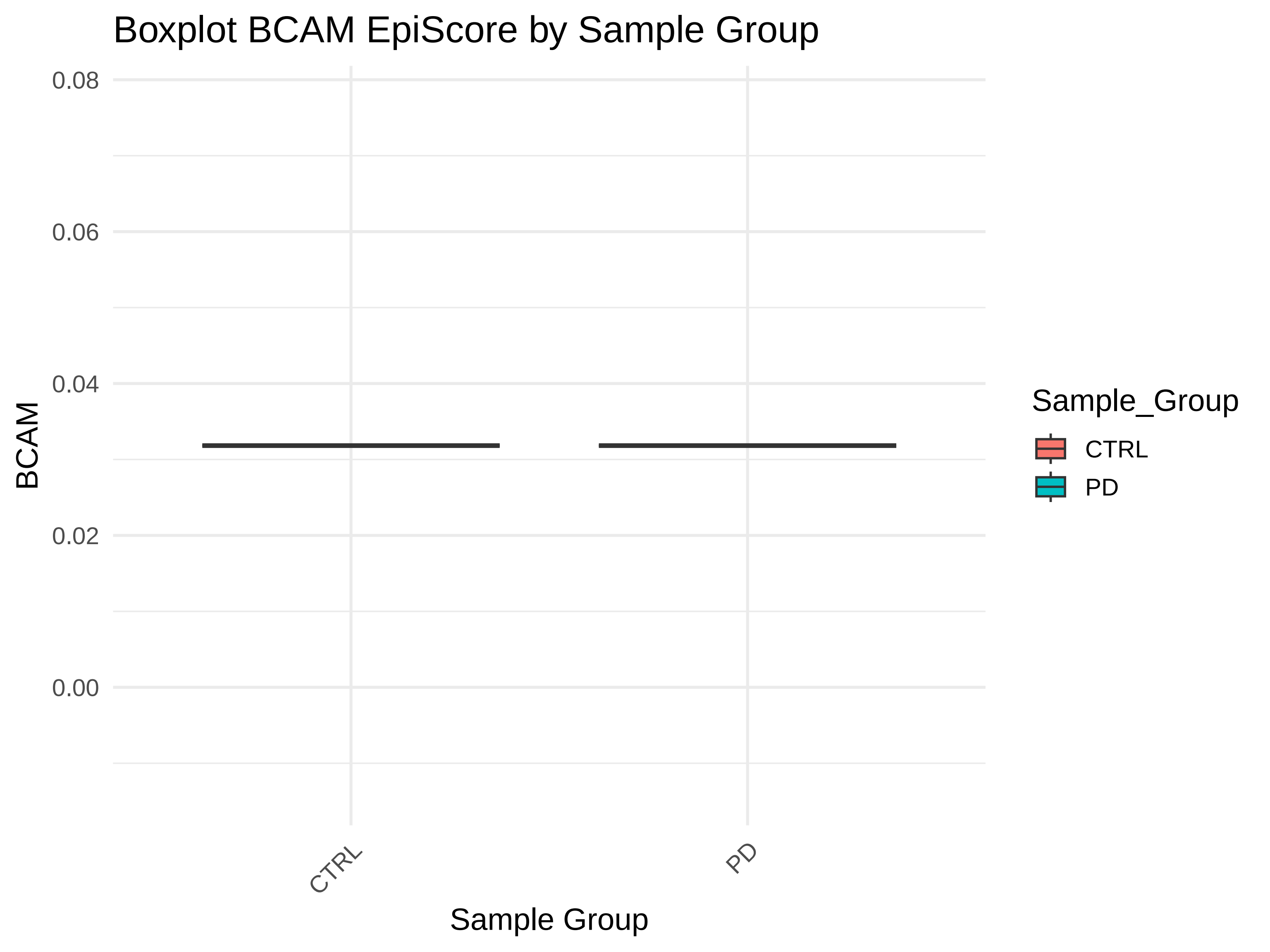

Boxplot BCAM EpiScore by Sample Group
0.08
0.06
0.04
0.02
0.00
BCAM
CTRL
PD
Sample Group
Sample_Group
CTRL
PD

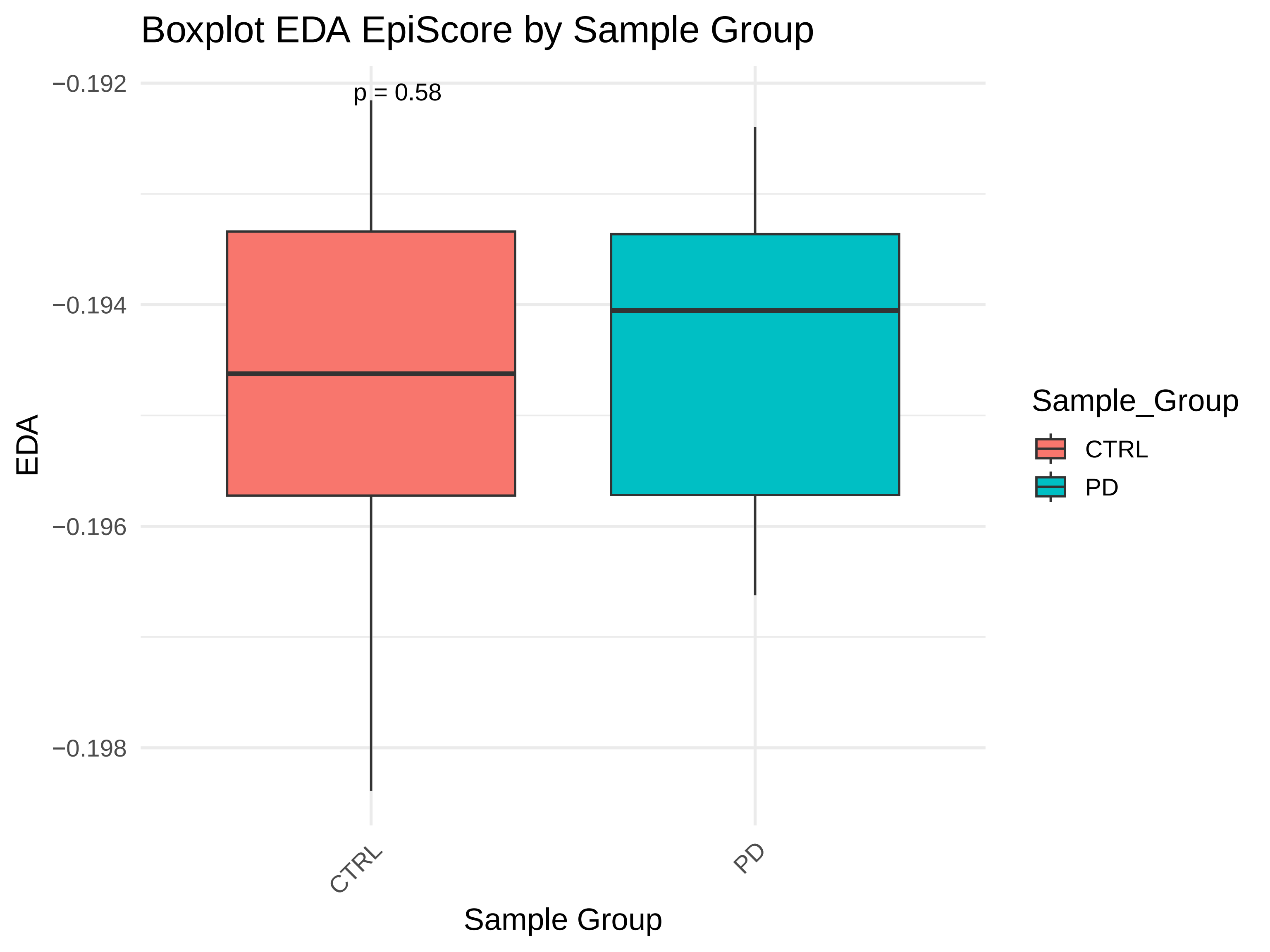
Boxplot EDA EpiScore by Sample Group
p = 0.58
−0.192
−0.194
−0.196
−0.198
EDA
CTRL
PD
Sample Group
Sample_Group
CTRL
PD

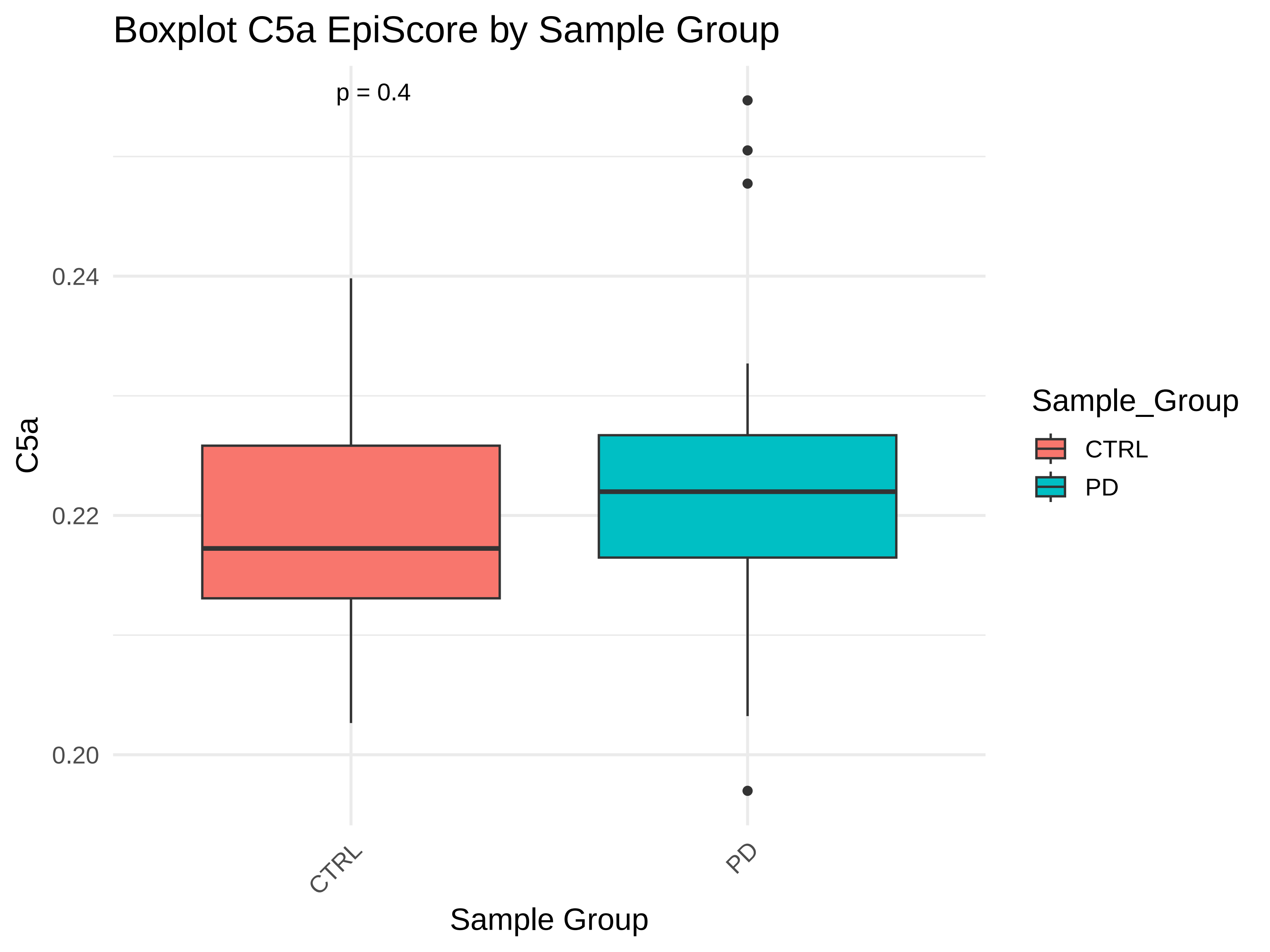
Boxplot C5a EpiScore by Sample Group
p = 0.4
0.24
0.22
0.20
C5a
CTRL
PD
Sample Group
Sample_Group
CTRL
PD

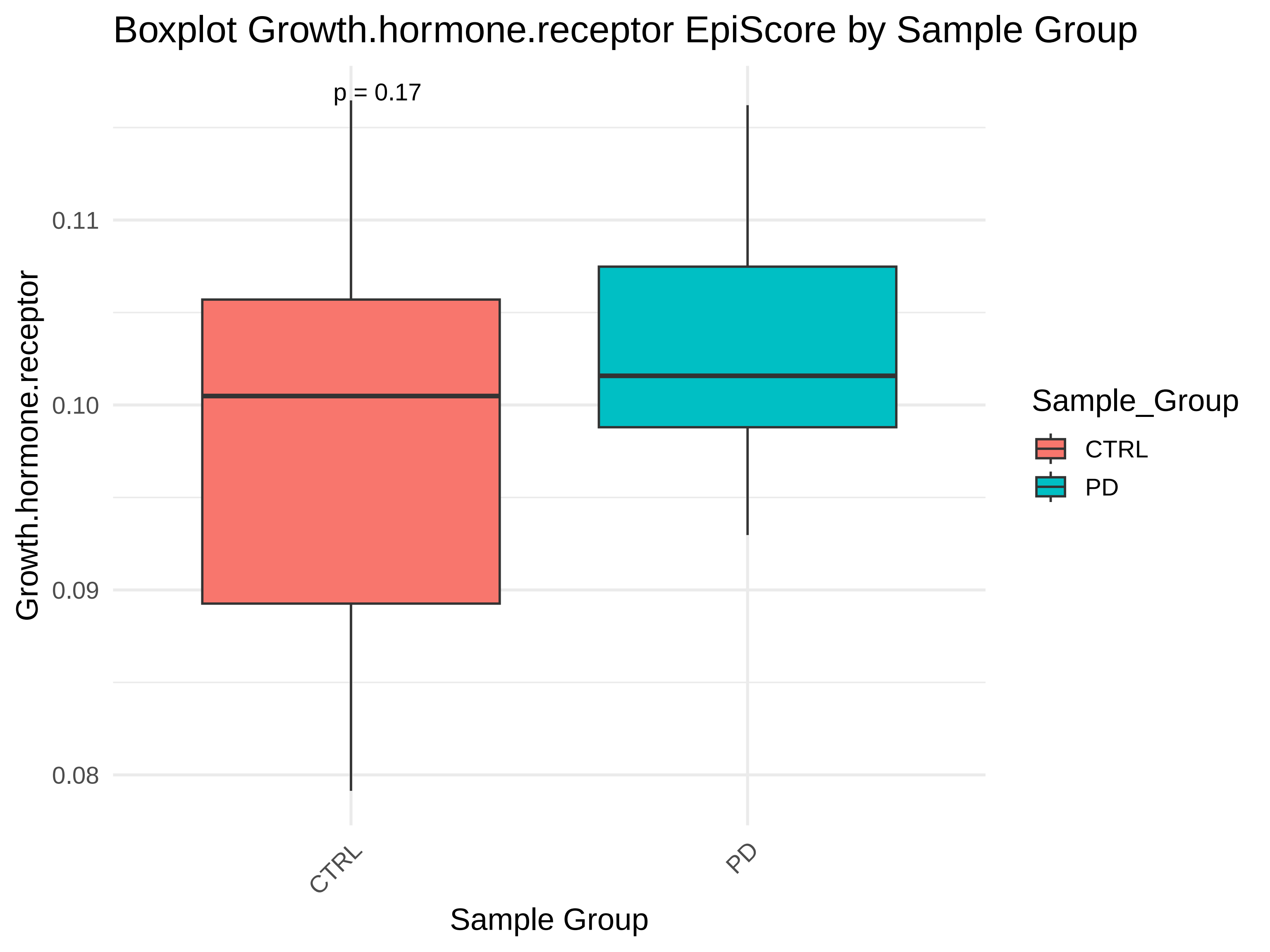

Boxplot Growth.hormone.receptor EpiScore by Sample Group
p = 0.17
Growth.hormone.receptor
0.11
0.10
0.09
0.08
CTRL
PD
Sample Group
Sample_Group
CTRL
PD

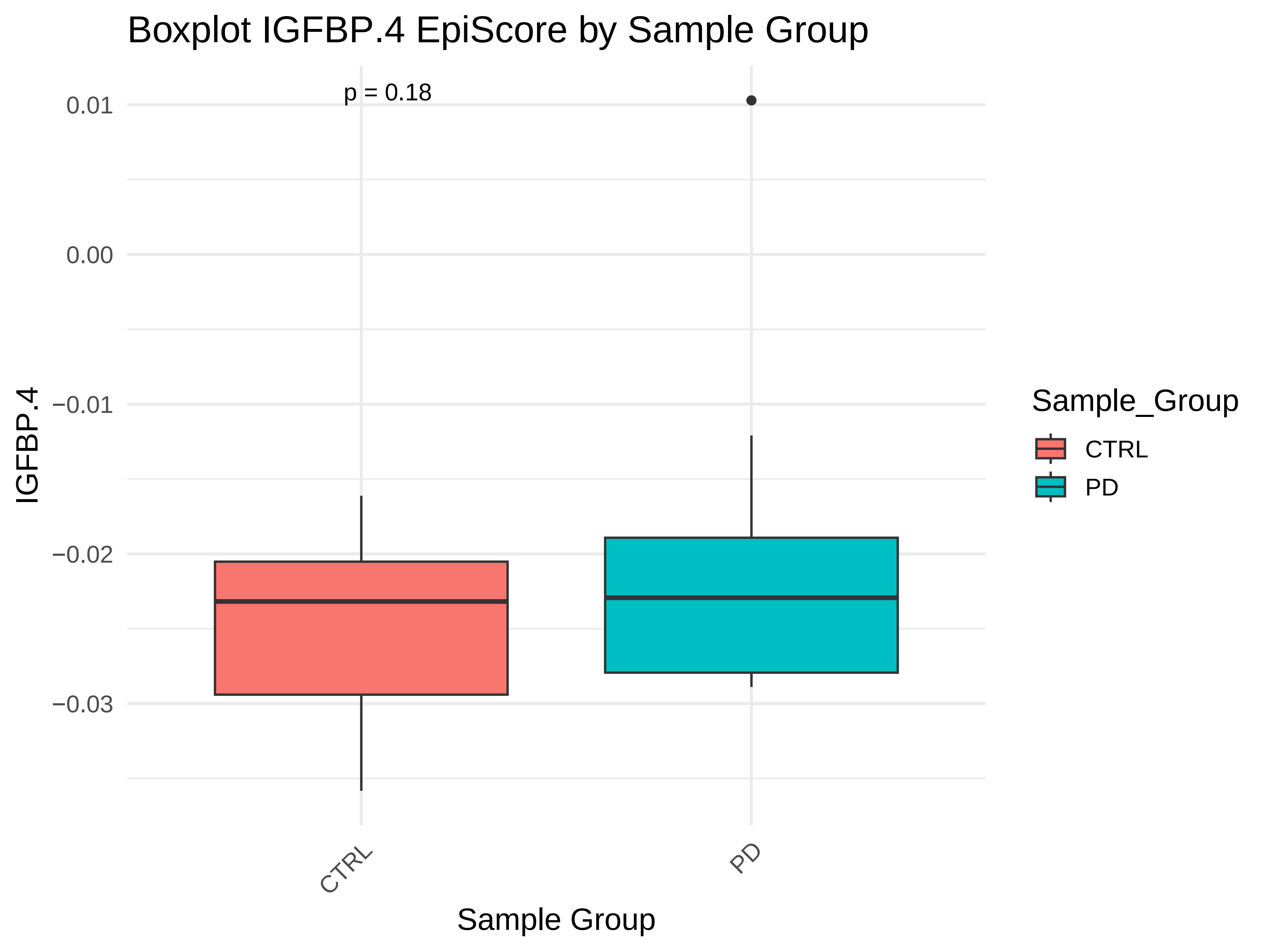
Boxplot IGFBP.4 EpiScore by Sample Group
p = 0.18
0.01
0.00
−0.01
−0.02
−0.03
IGFBP.4
CTRL
PD
Sample Group
Sample_Group
CTRL
PD

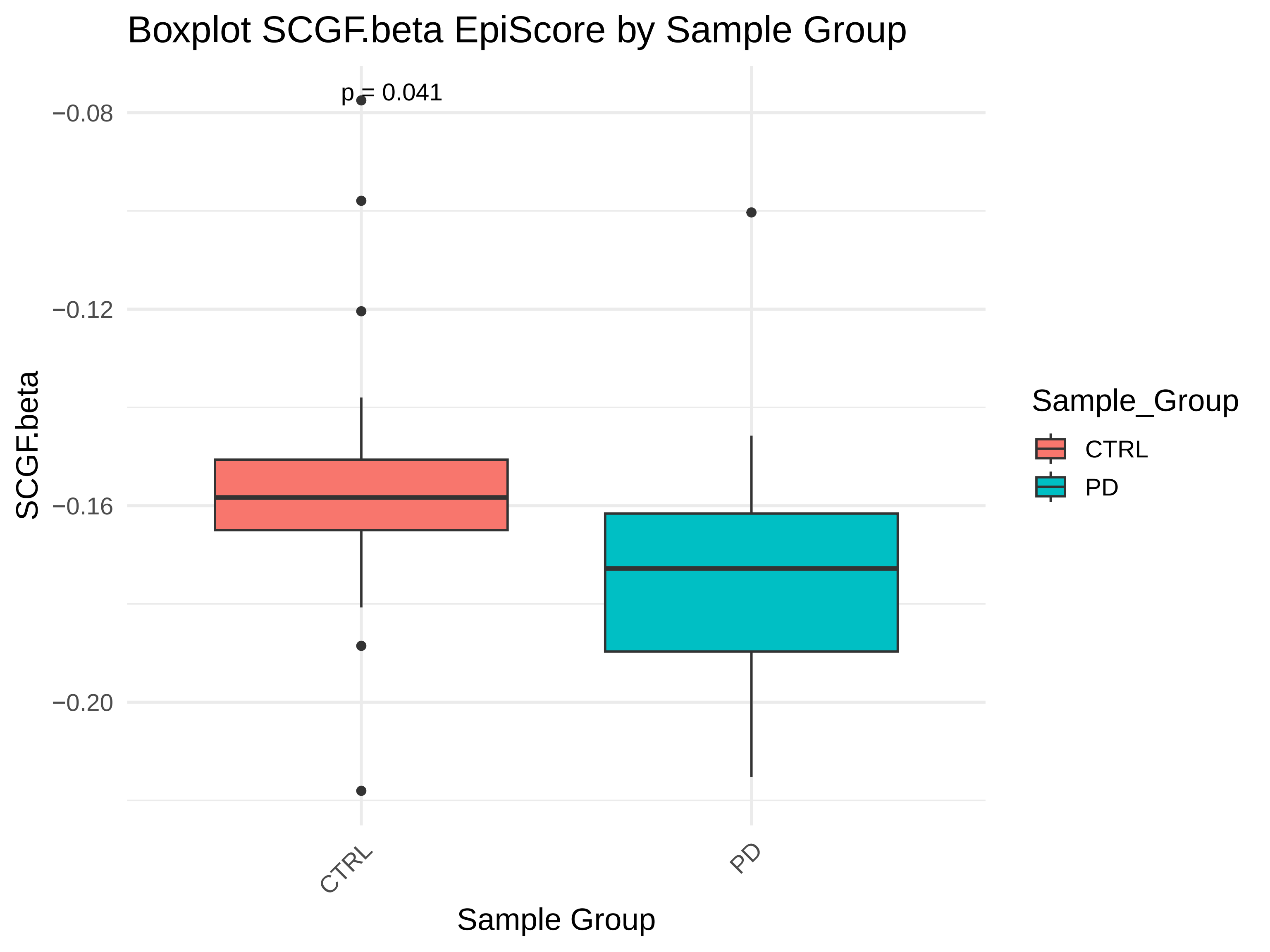

Boxplot SCGF.beta EpiScore by Sample Group
p = 0.041
−0.08
−0.12
−0.16
−0.20
SCGF.beta
CTRL
PD
Sample Group
Sample_Group
CTRL
PD

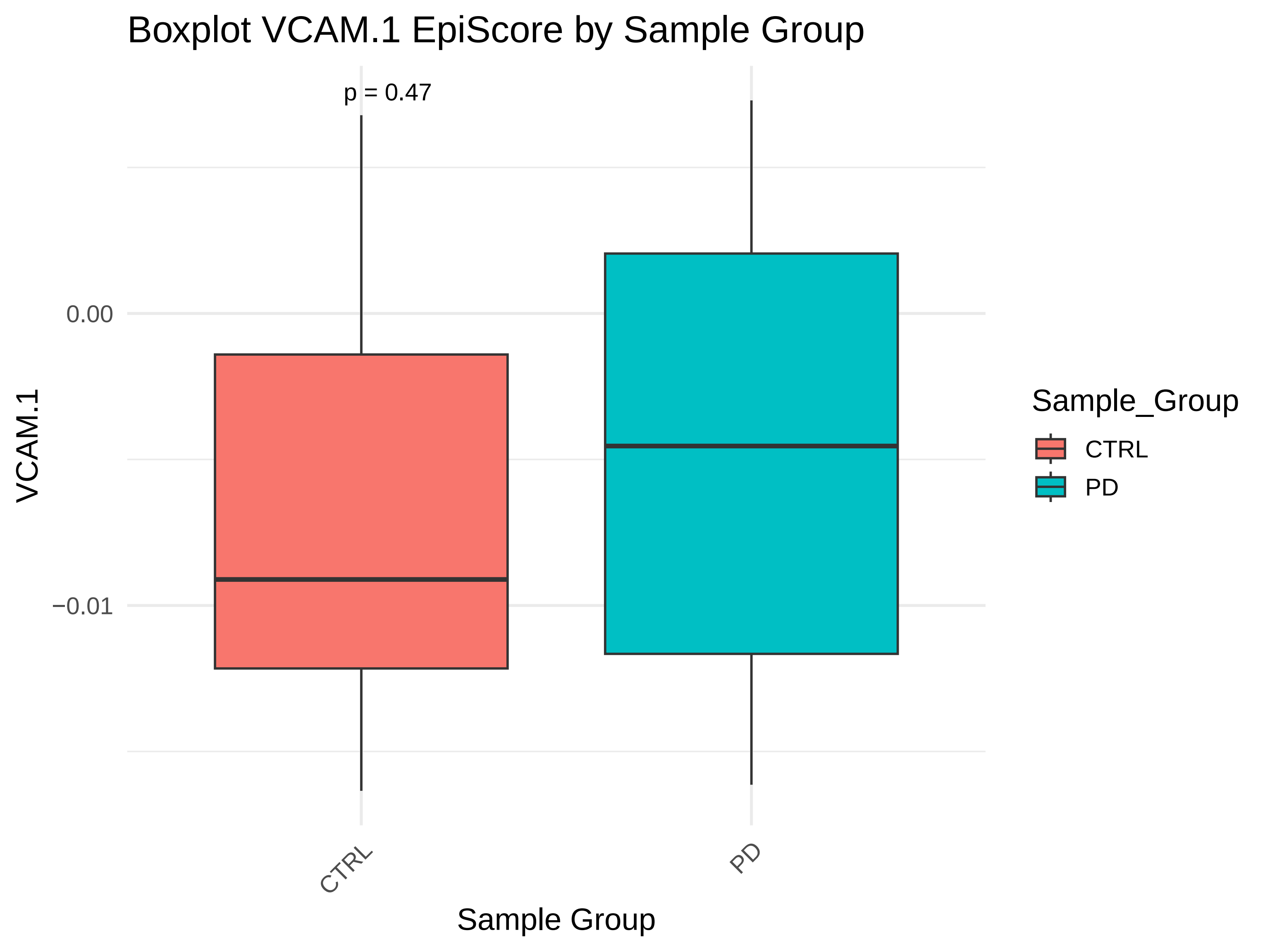

Boxplot VCAM.1 EpiScore by Sample Group
p = 0.47
0.00
−0.01
VCAM.1
CTRL
PD
Sample Group
Sample_Group
CTRL
PD

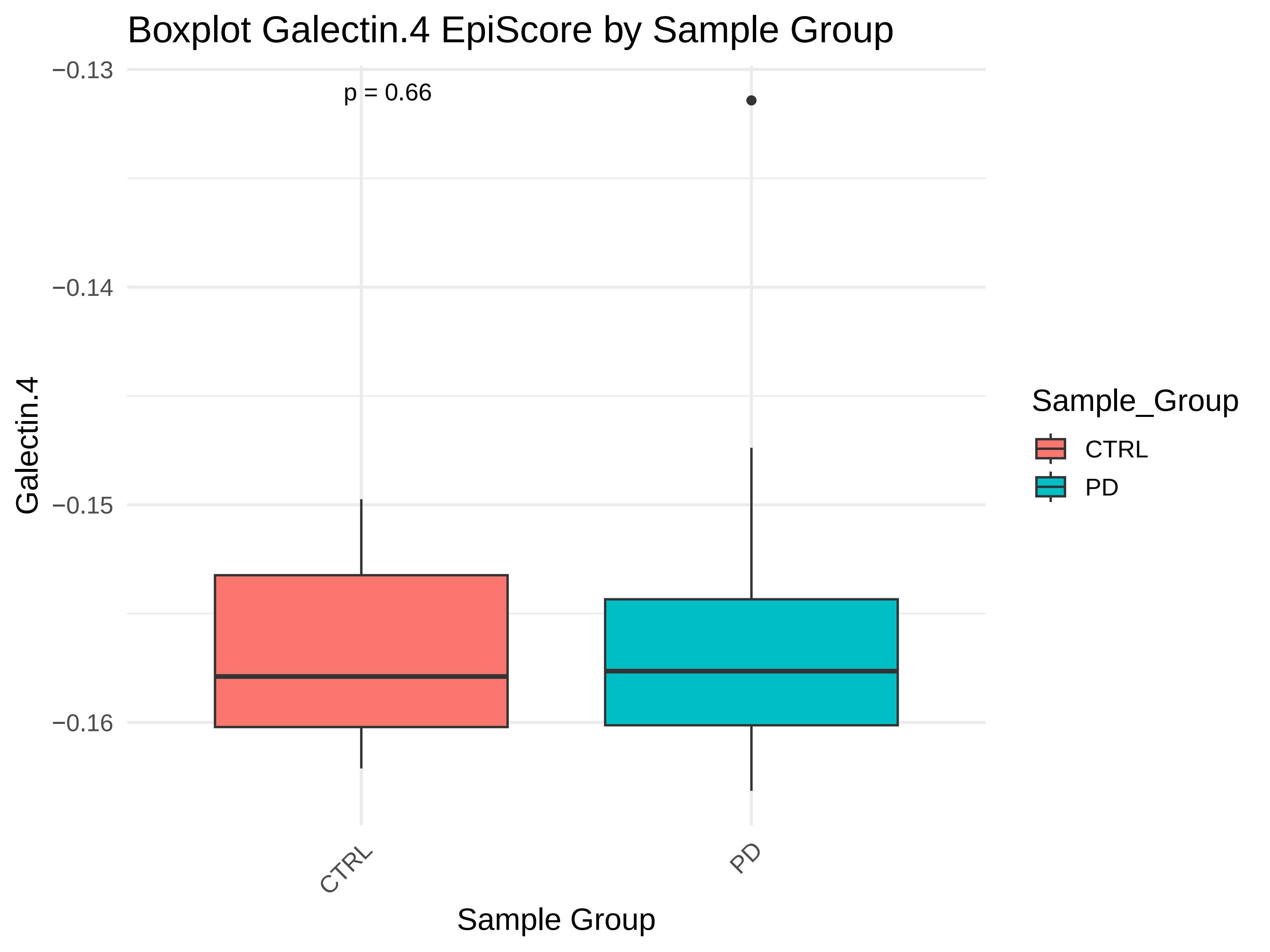

Boxplot Galectin.4 EpiScore by Sample Group
p = 0.66
−0.13
−0.14
−0.15
−0.16
Galectin.4
CTRL
PD
Sample Group
Sample_Group
CTRL
PD

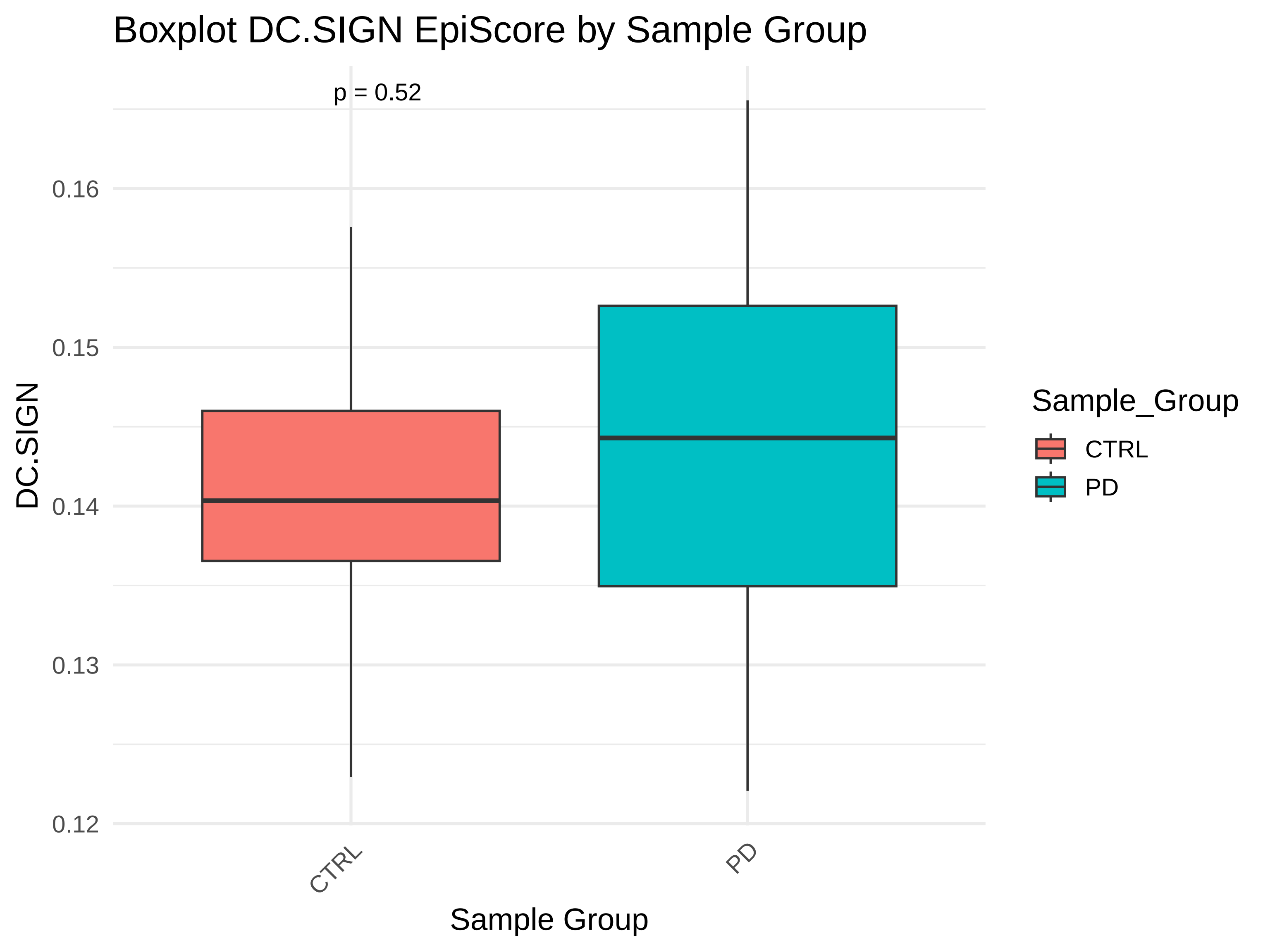

Boxplot DC.SIGN EpiScore by Sample Group
p = 0.52
DC.SIGN
0.16
0.15
0.14
0.13
0.12
CTRL
PD
Sample Group
Sample_Group
CTRL
PD

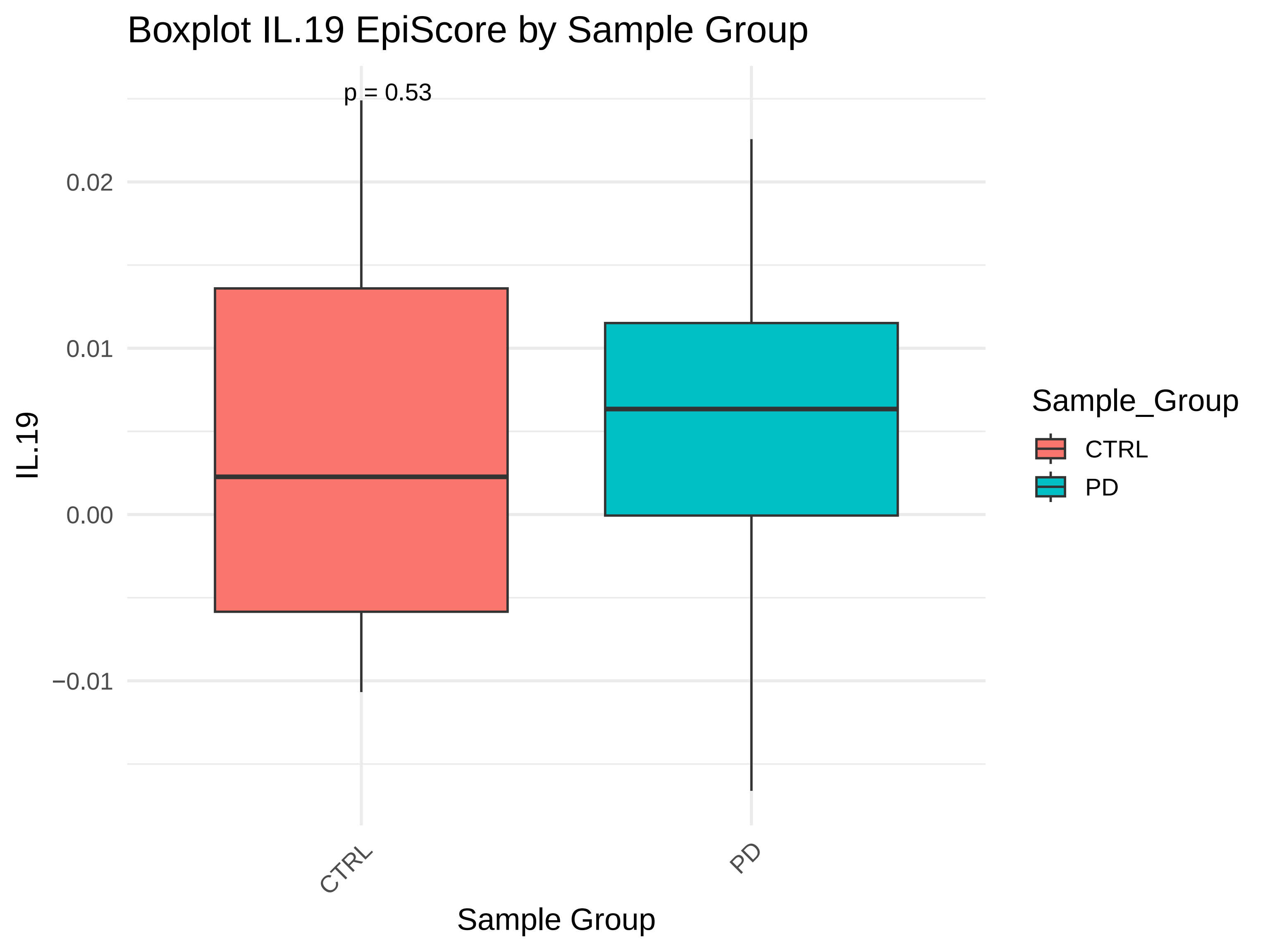

Boxplot IL.19 EpiScore by Sample Group
p = 0.53
0.02
0.01
0.00
−0.01
IL.19
CTRL
PD
Sample Group
Sample_Group
CTRL
PD

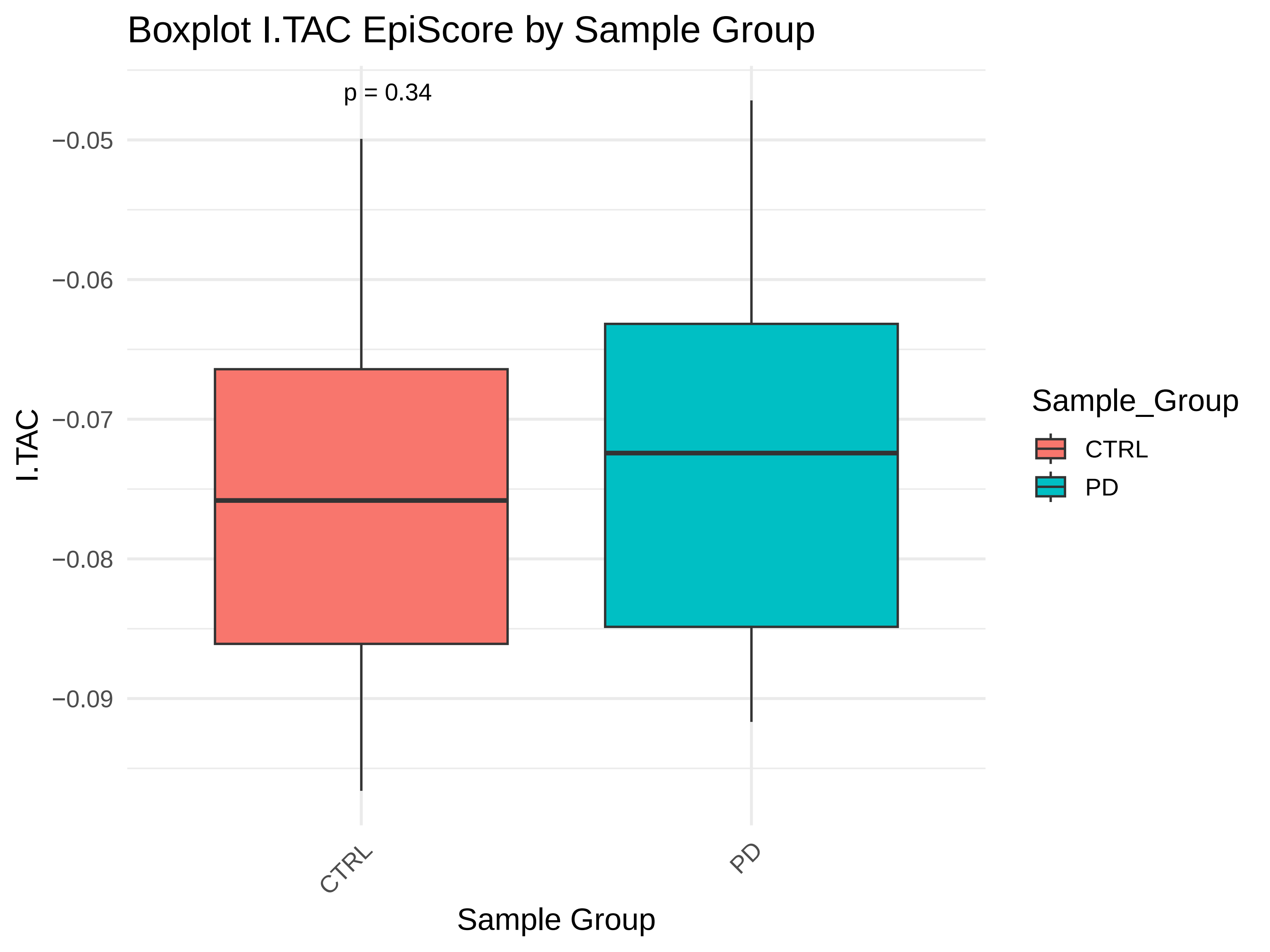

Boxplot I.TAC EpiScore by Sample Group
p = 0.34
−0.05
−0.06
−0.07
−0.08
−0.09
I.TAC
CTRL
PD
Sample Group
Sample_Group
CTRL
PD

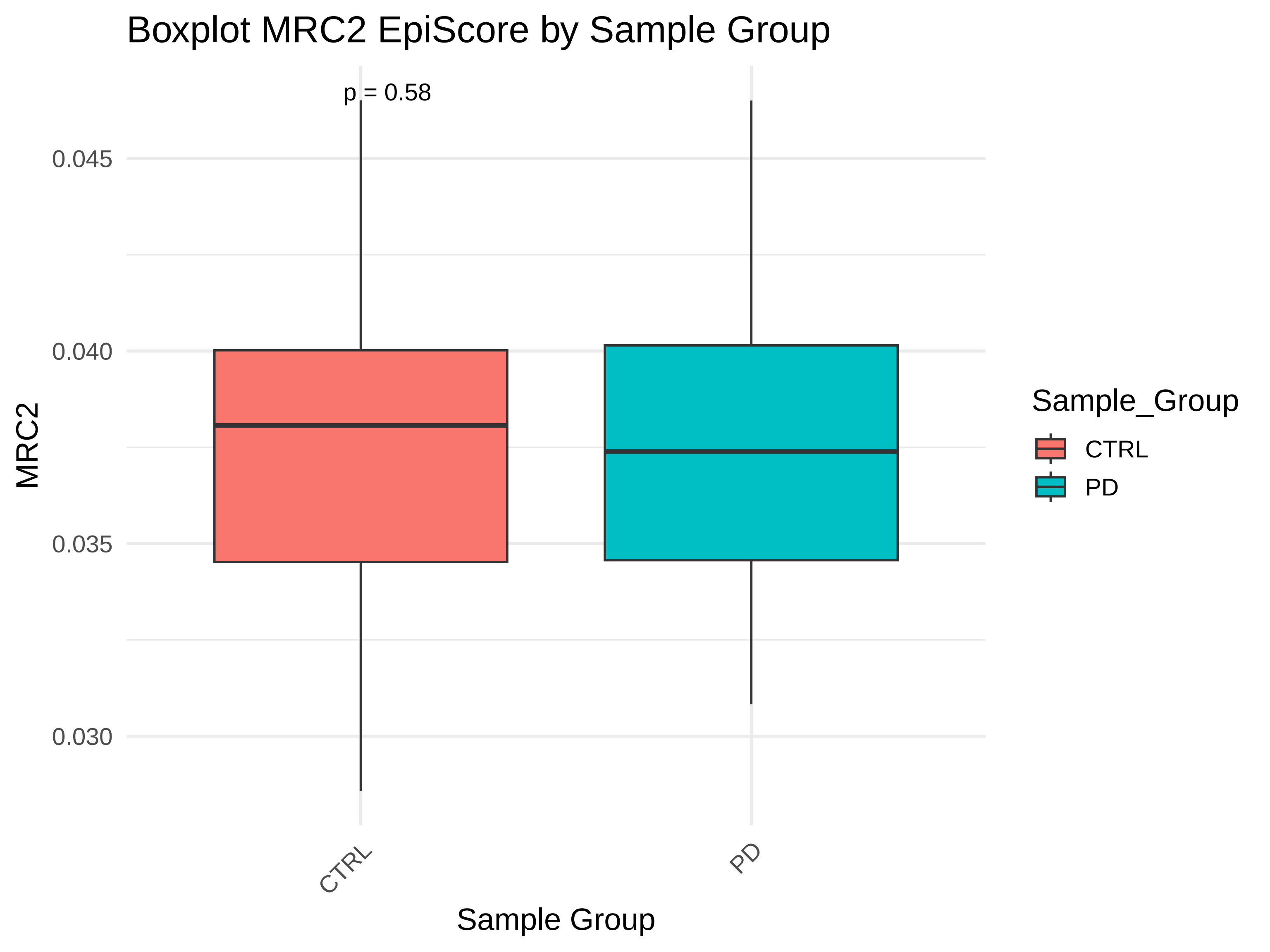
Boxplot MRC2 EpiScore by Sample Group
p = 0.58
0.045
0.040
0.035
0.030
MRC2
CTRL
PD
Sample Group
Sample_Group
CTRL
PD

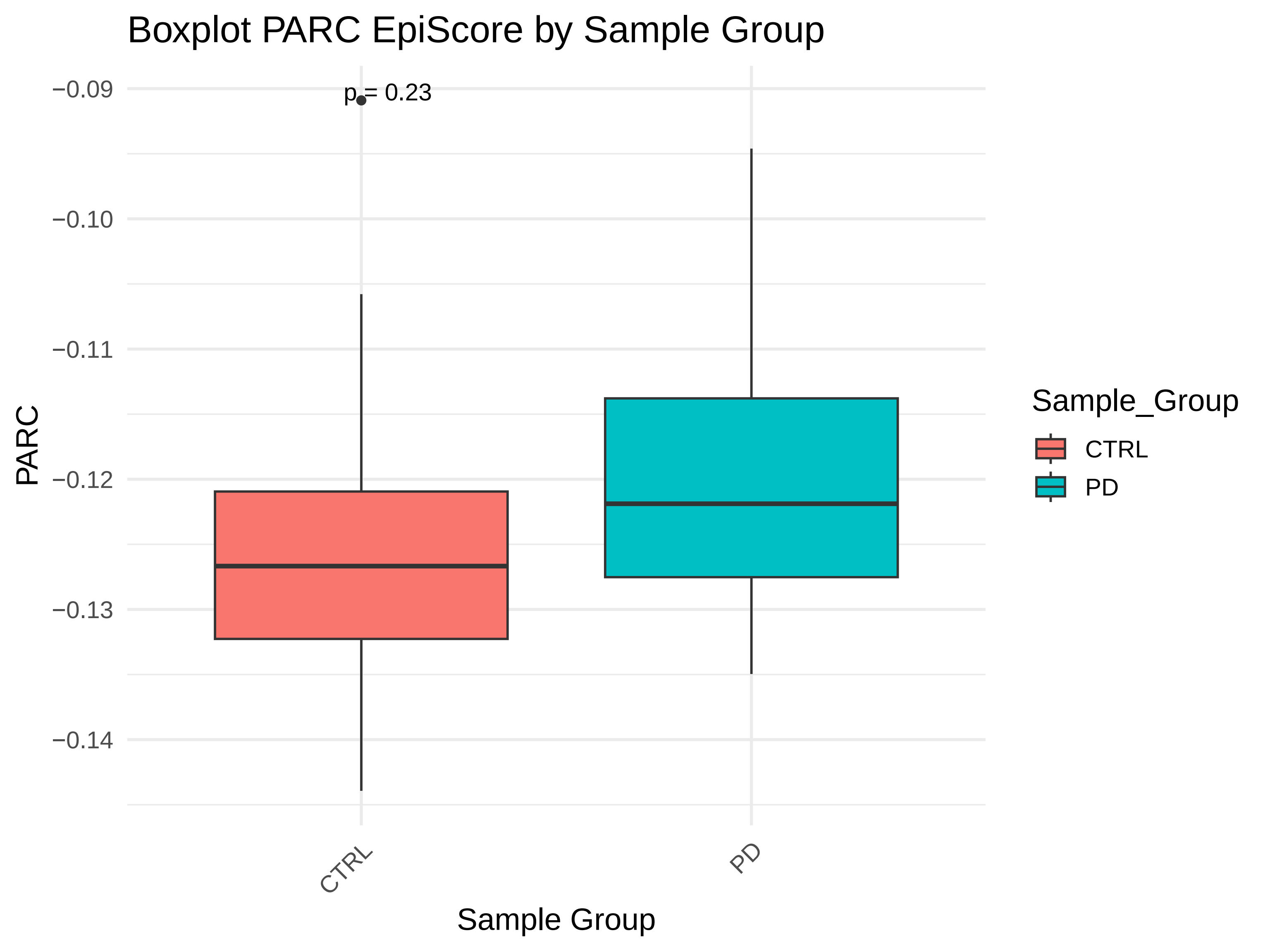
Boxplot PARC EpiScore by Sample Group
p = 0.23
−0.09
−0.10
−0.11
−0.12
−0.13
−0.14
PARC
CTRL
PD
Sample Group
Sample_Group
CTRL
PD

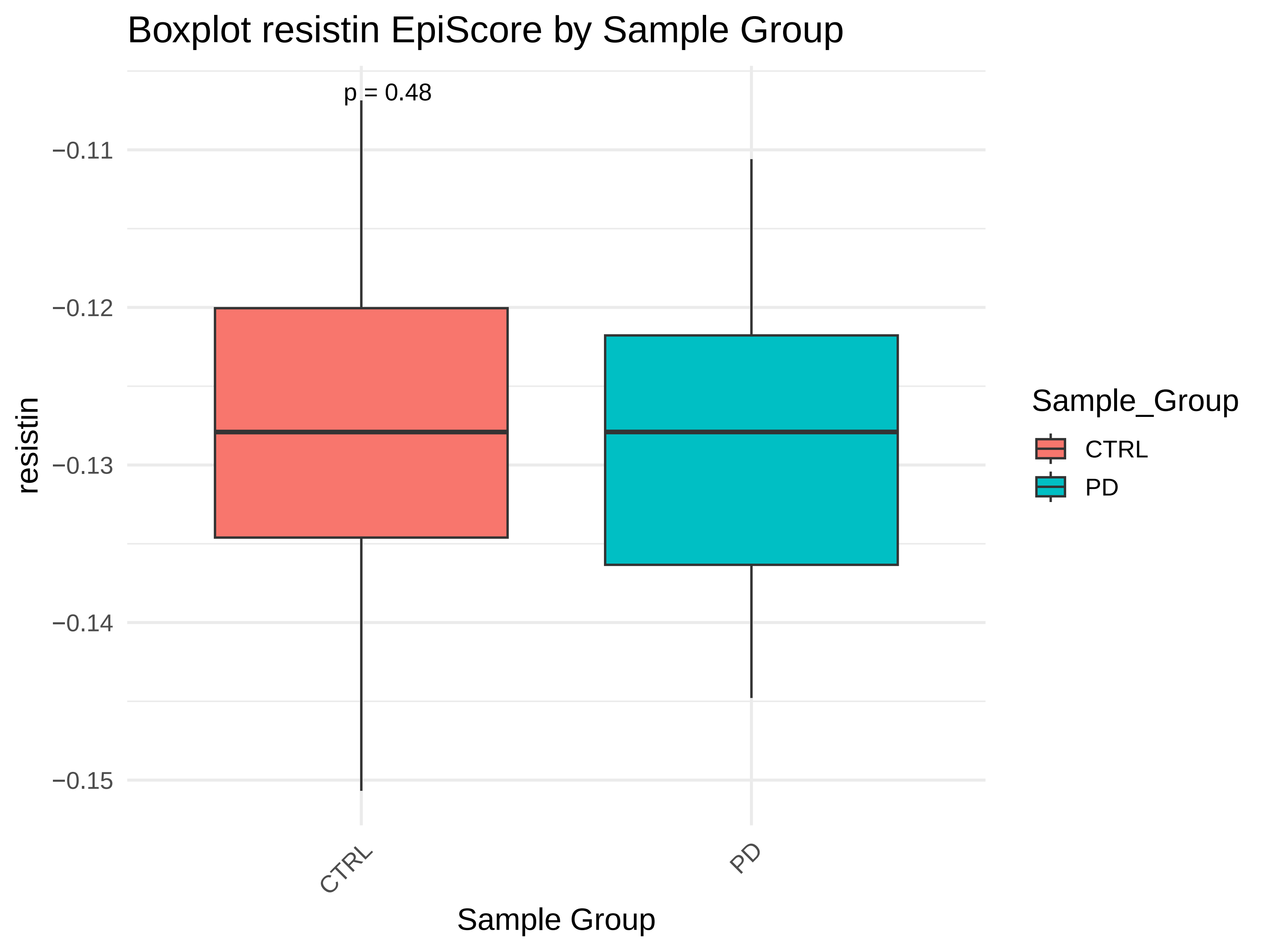

Boxplot resistin EpiScore by Sample Group
p = 0.48
−0.11
−0.12
−0.13
−0.14
−0.15
resistin
CTRL
PD
Sample Group
Sample_Group
CTRL
PD

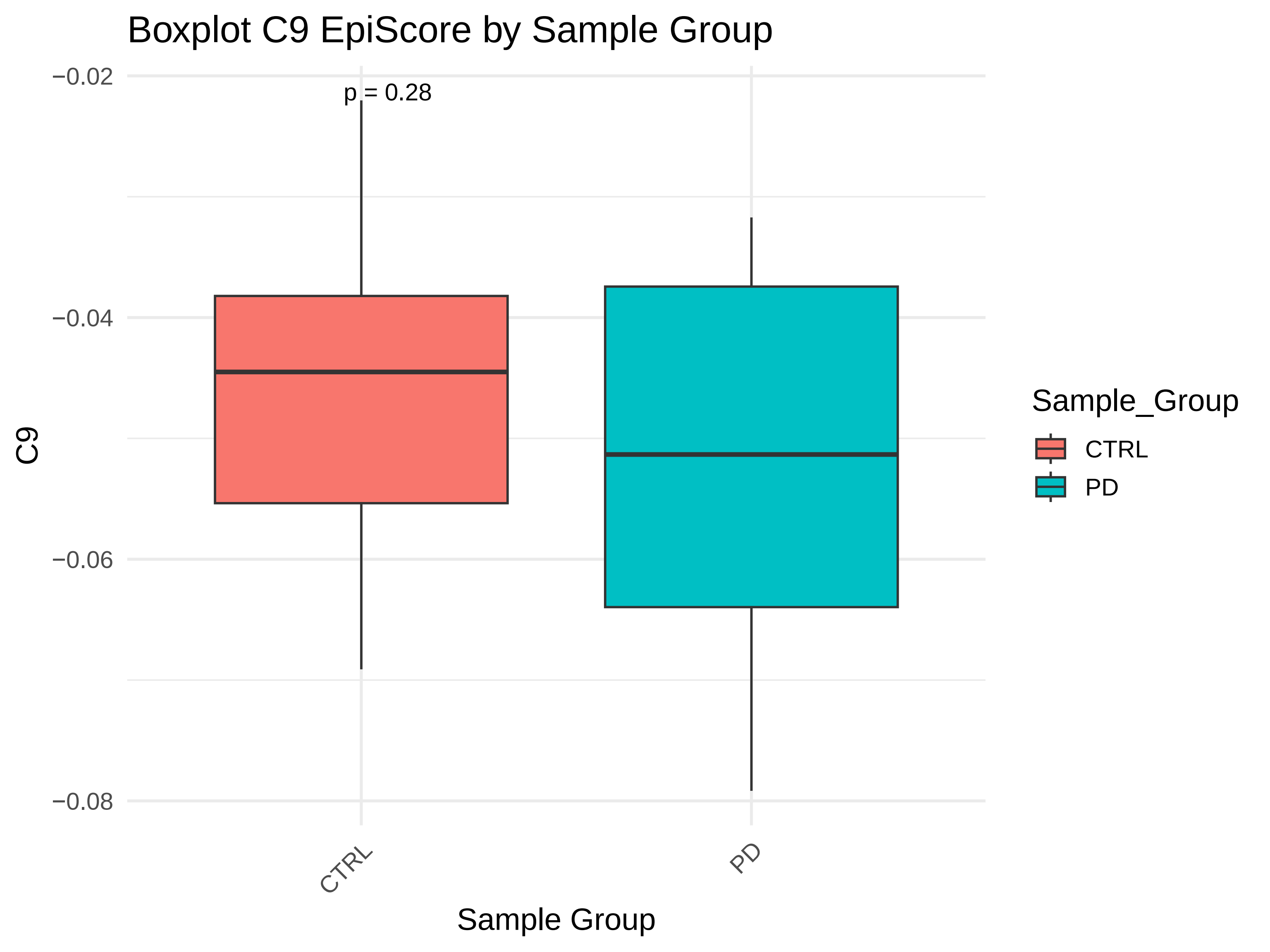

Boxplot C9 EpiScore by Sample Group
p = 0.28
−0.02
−0.04
−0.06
−0.08
C9
CTRL
PD
Sample Group
Sample_Group
CTRL
PD

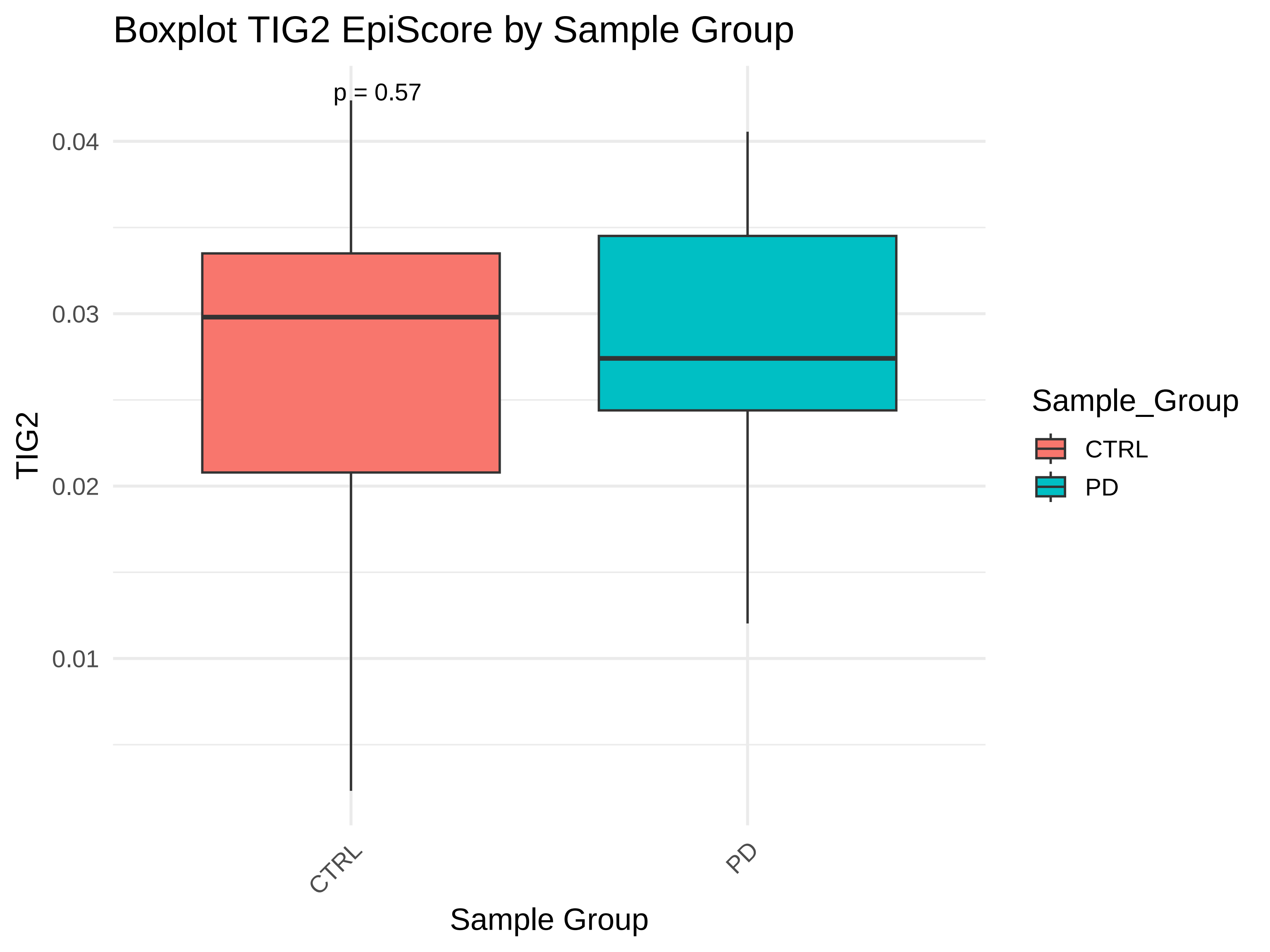

Boxplot TIG2 EpiScore by Sample Group
p = 0.57
0.04
0.03
0.02
0.01
TIG2
CTRL
PD
Sample Group
Sample_Group
CTRL
PD

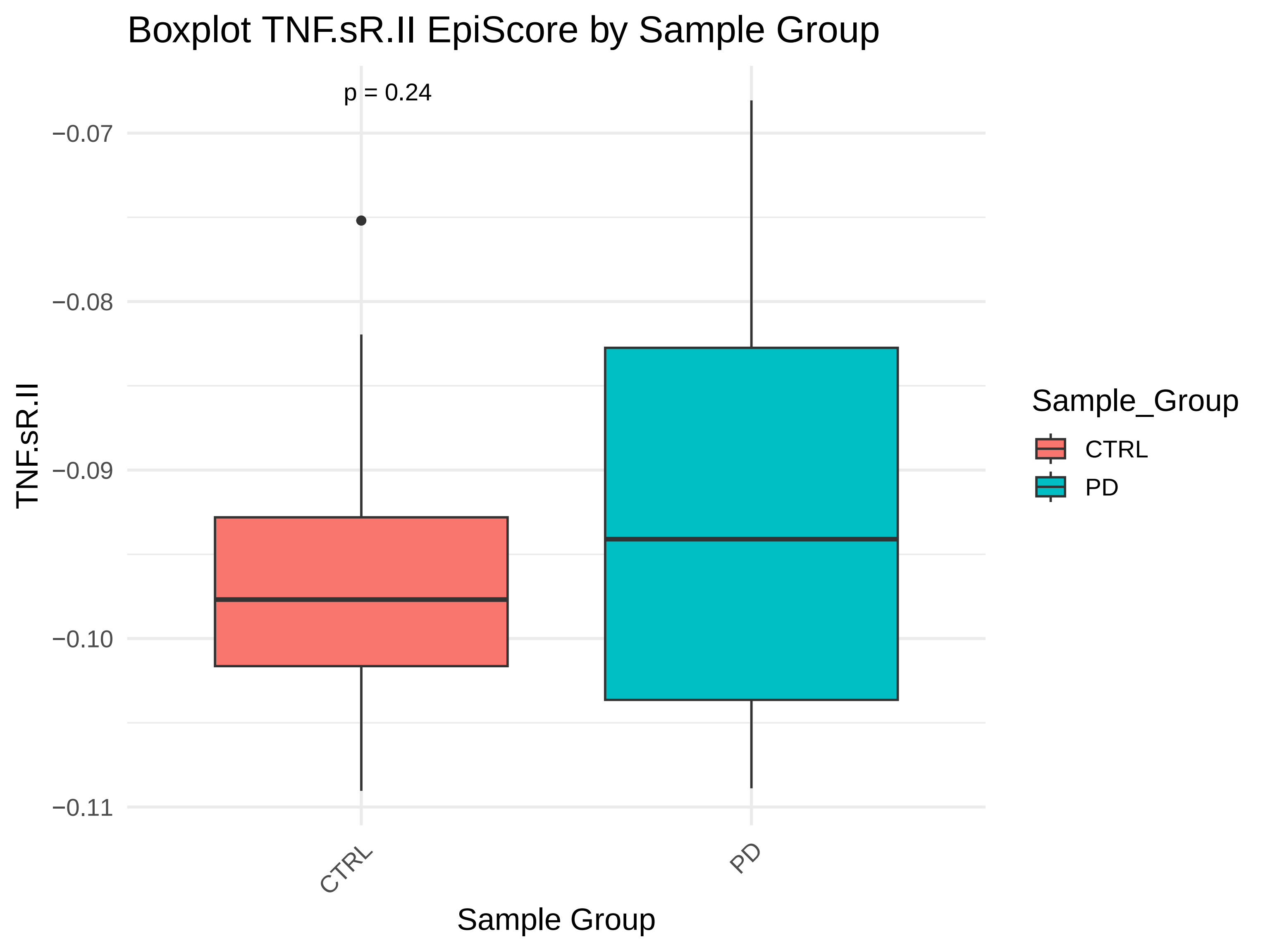
Boxplot TNF.sR.II EpiScore by Sample Group
p = 0.24
−0.07
−0.08
−0.09
−0.10
−0.11
TNF.sR.II
CTRL
PD
Sample Group
Sample_Group
CTRL
PD

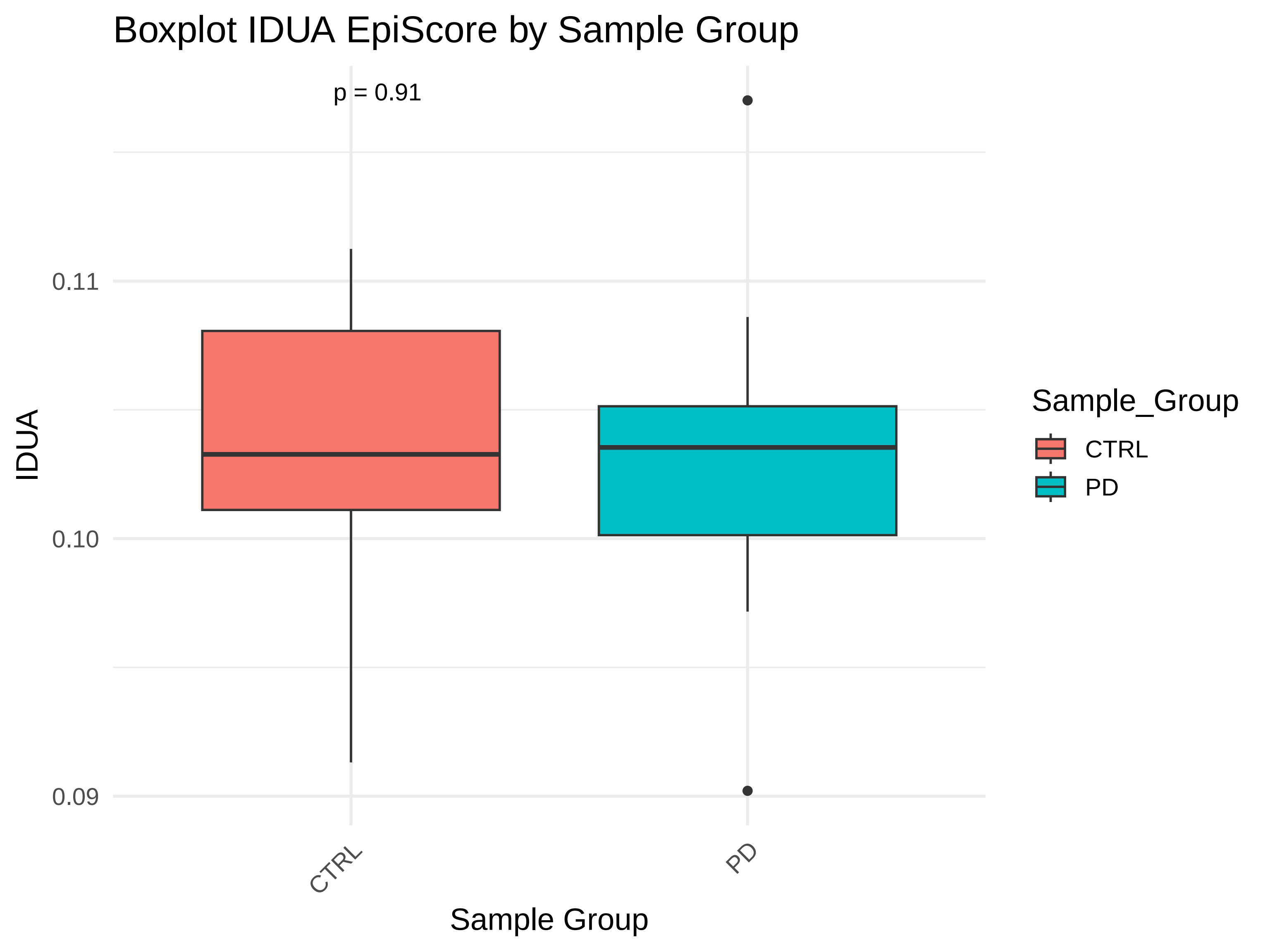
Boxplot IDUA EpiScore by Sample Group
p = 0.91
0.11
0.10
0.09
IDUA
CTRL
PD
Sample Group
Sample_Group
CTRL
PD

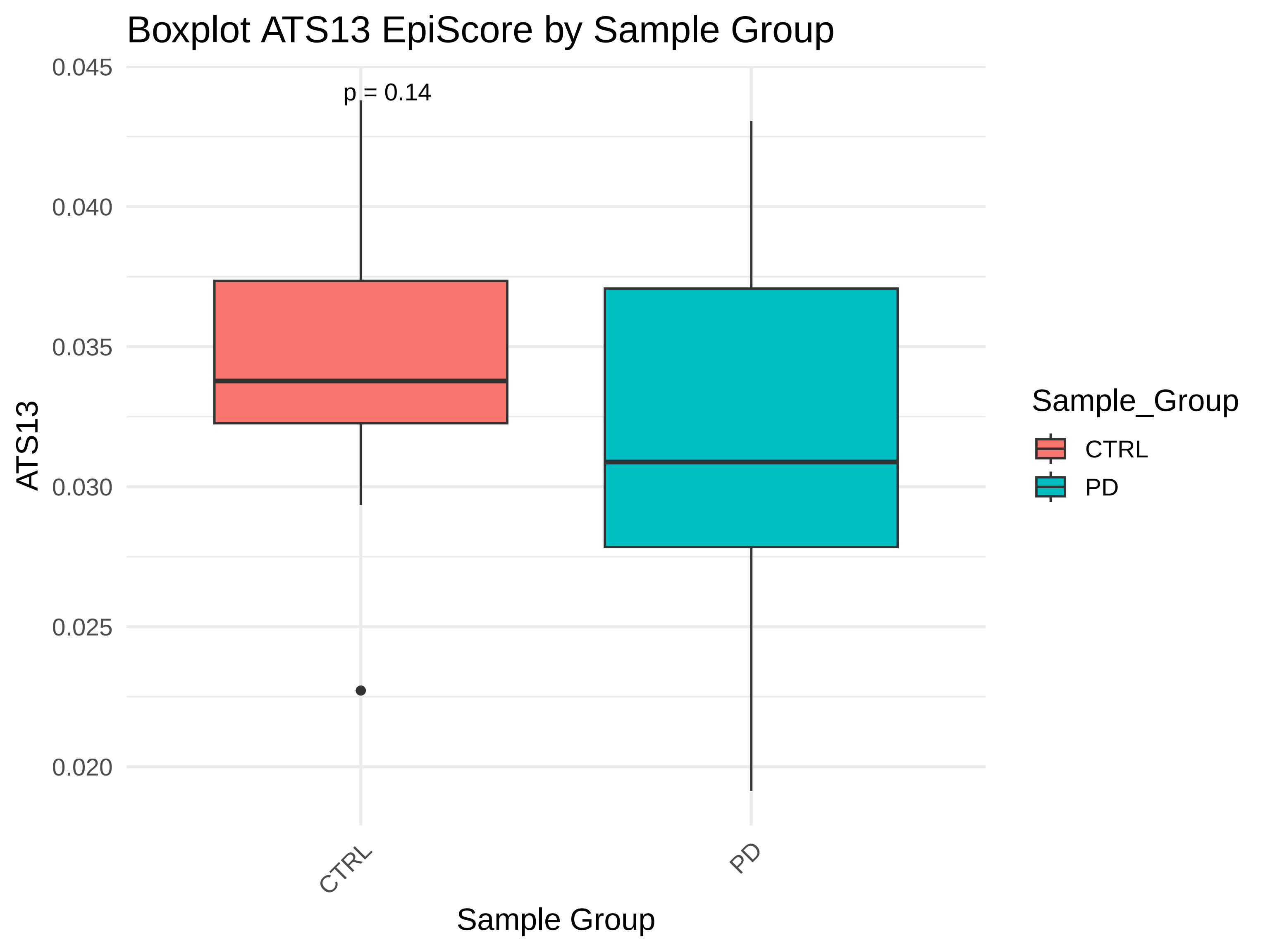

Boxplot ATS13 EpiScore by Sample Group
p = 0.14
0.045
0.040
0.035
0.030
0.025
0.020
ATS13
CTRL
PD
Sample Group
Sample_Group
CTRL
PD

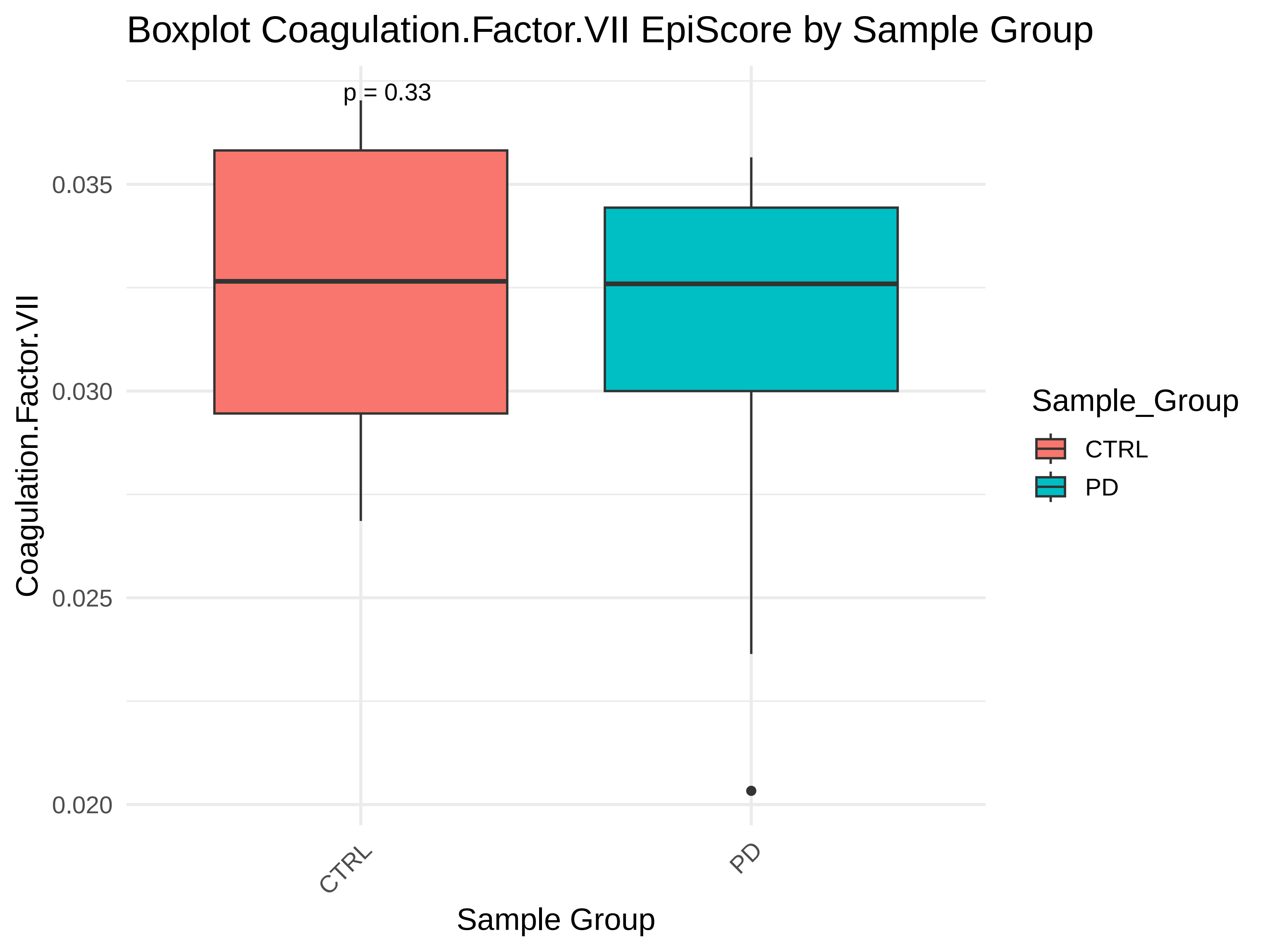

Boxplot Coagulation.Factor.VII EpiScore by Sample Group
p = 0.33
0.035
0.030
0.025
0.020
Coagulation.Factor.VII
CTRL
PD
Sample Group
Sample_Group
CTRL
PD

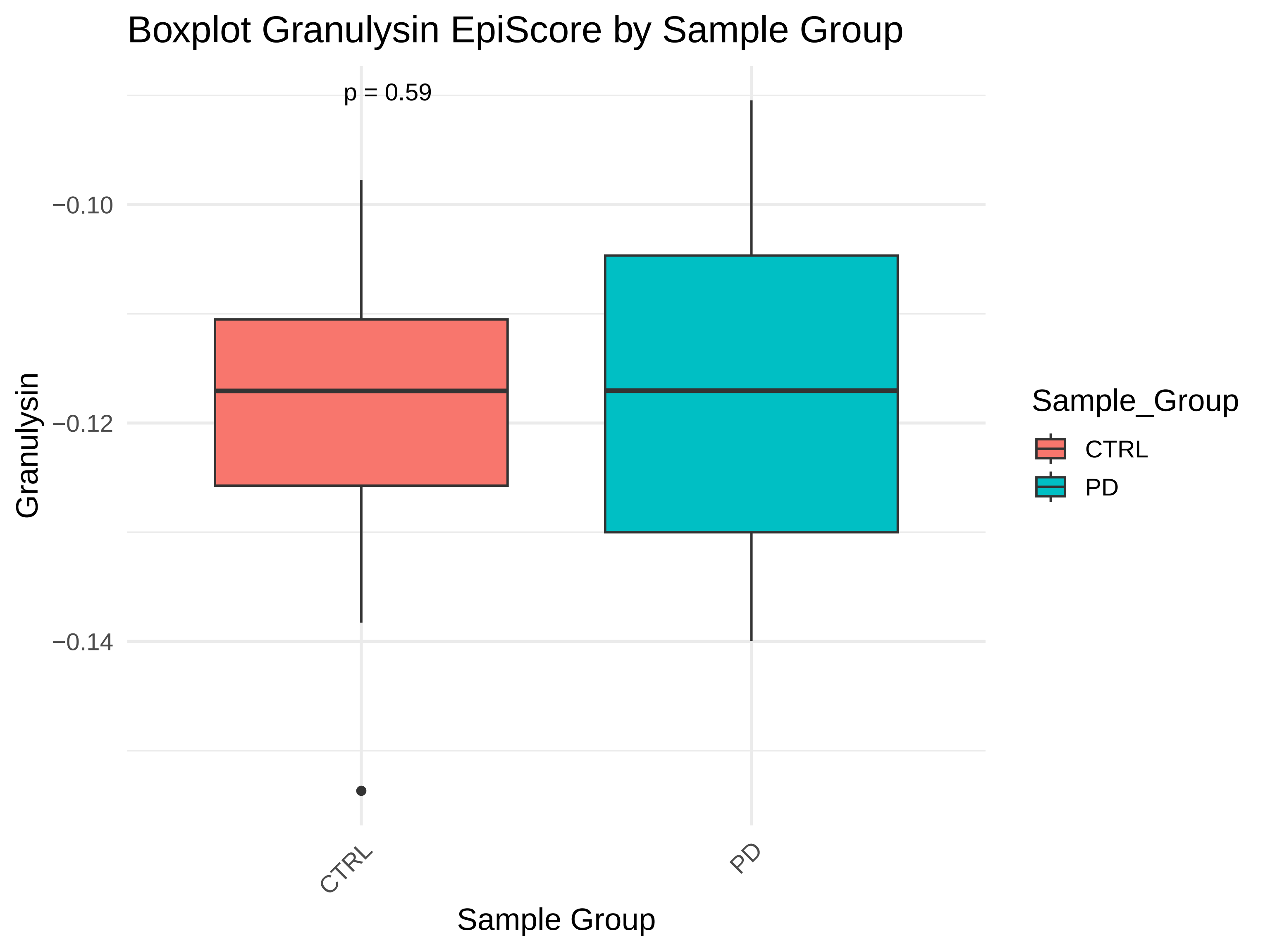
Boxplot Granulysin EpiScore by Sample Group
p = 0.59
−0.10
−0.12
−0.14
Granulysin
CTRL
PD
Sample Group
Sample_Group
CTRL
PD

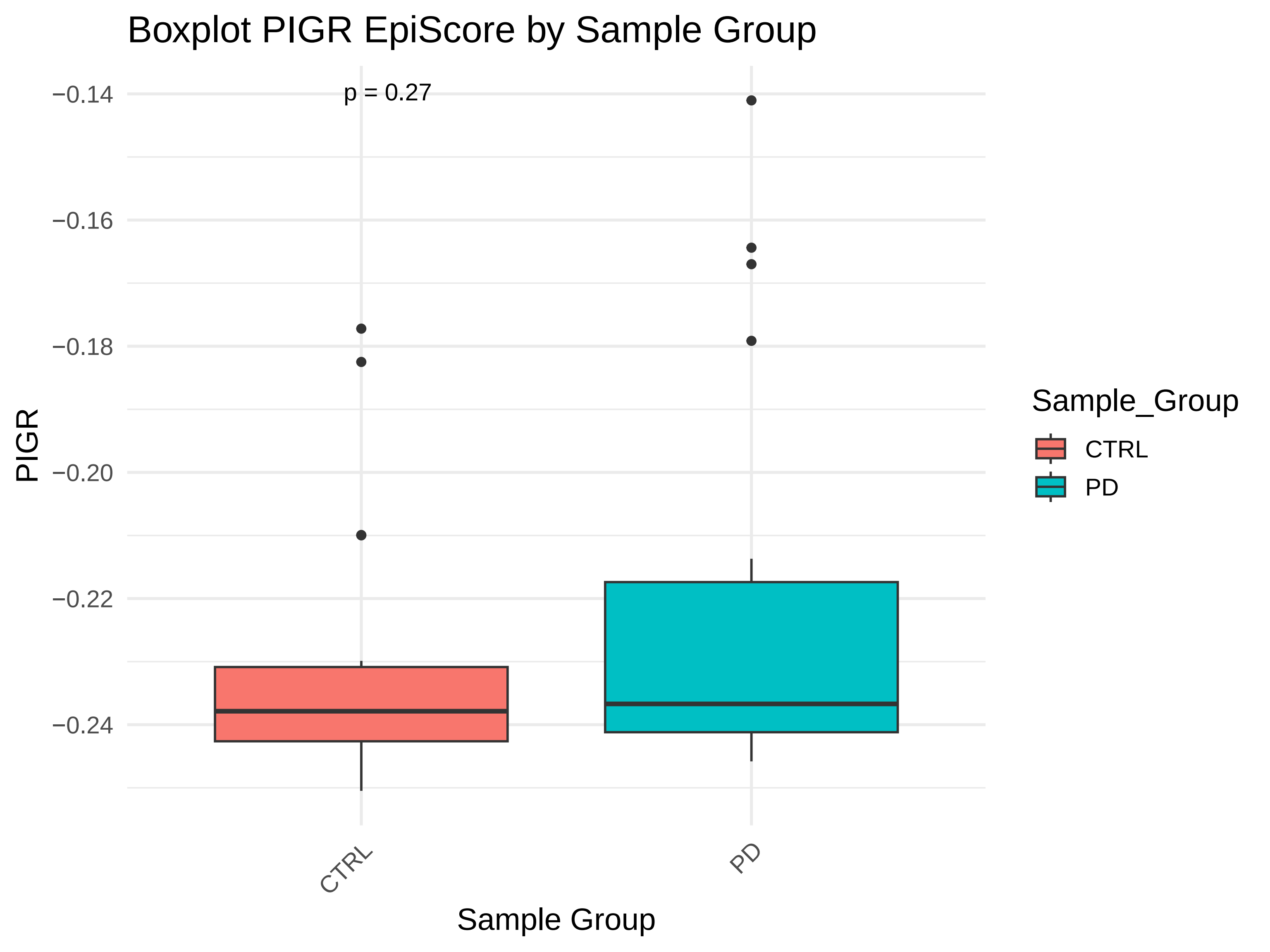
Boxplot PIGR EpiScore by Sample Group
p = 0.27
−0.14
−0.16
−0.18
−0.20
−0.22
−0.24
PIGR
CTRL
PD
Sample Group
Sample_Group
CTRL
PD

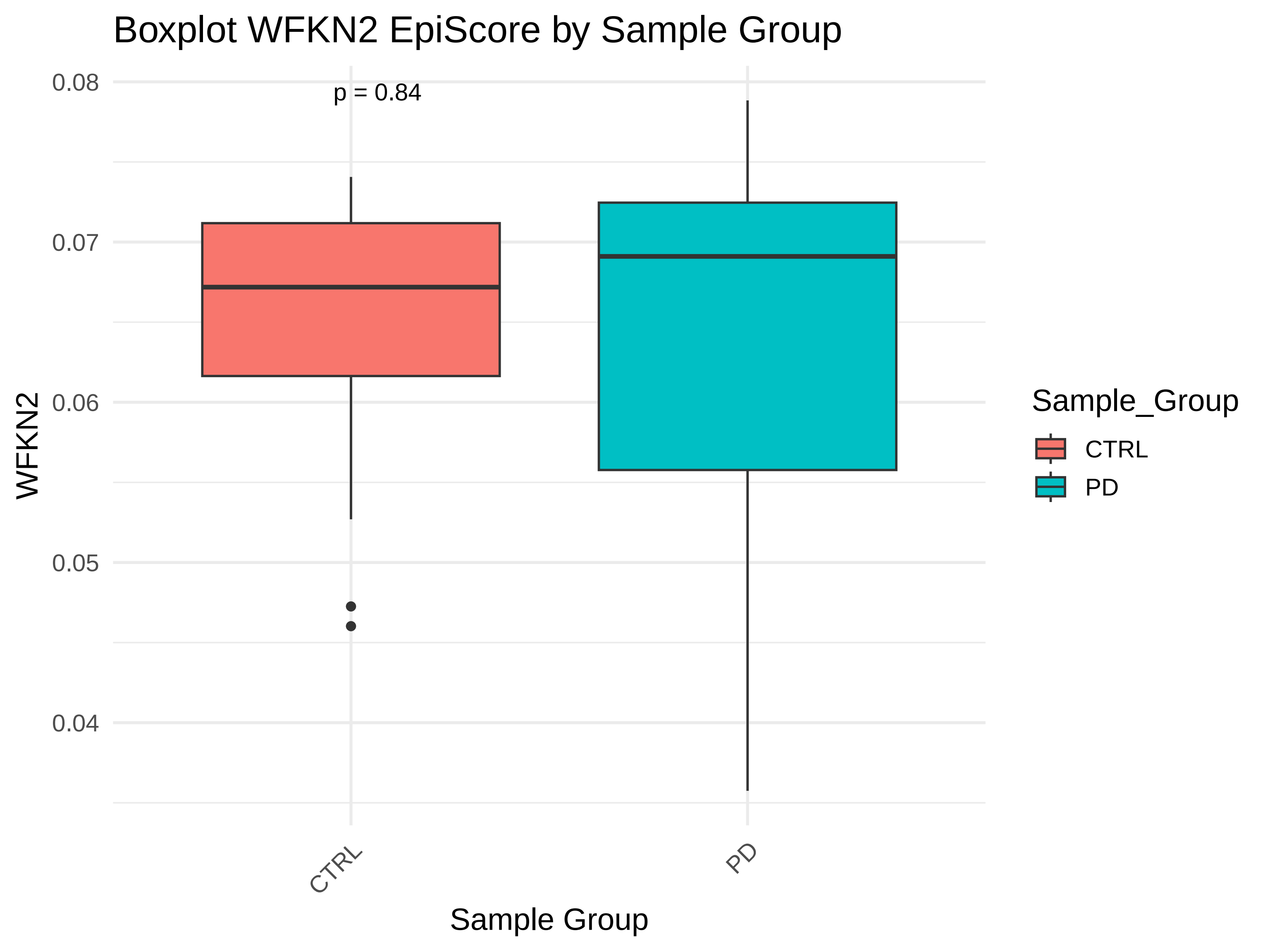

Boxplot WFKN2 EpiScore by Sample Group
p = 0.84
0.08
0.07
0.06
0.05
0.04
WFKN2
CTRL
PD
Sample Group
Sample_Group
CTRL
PD

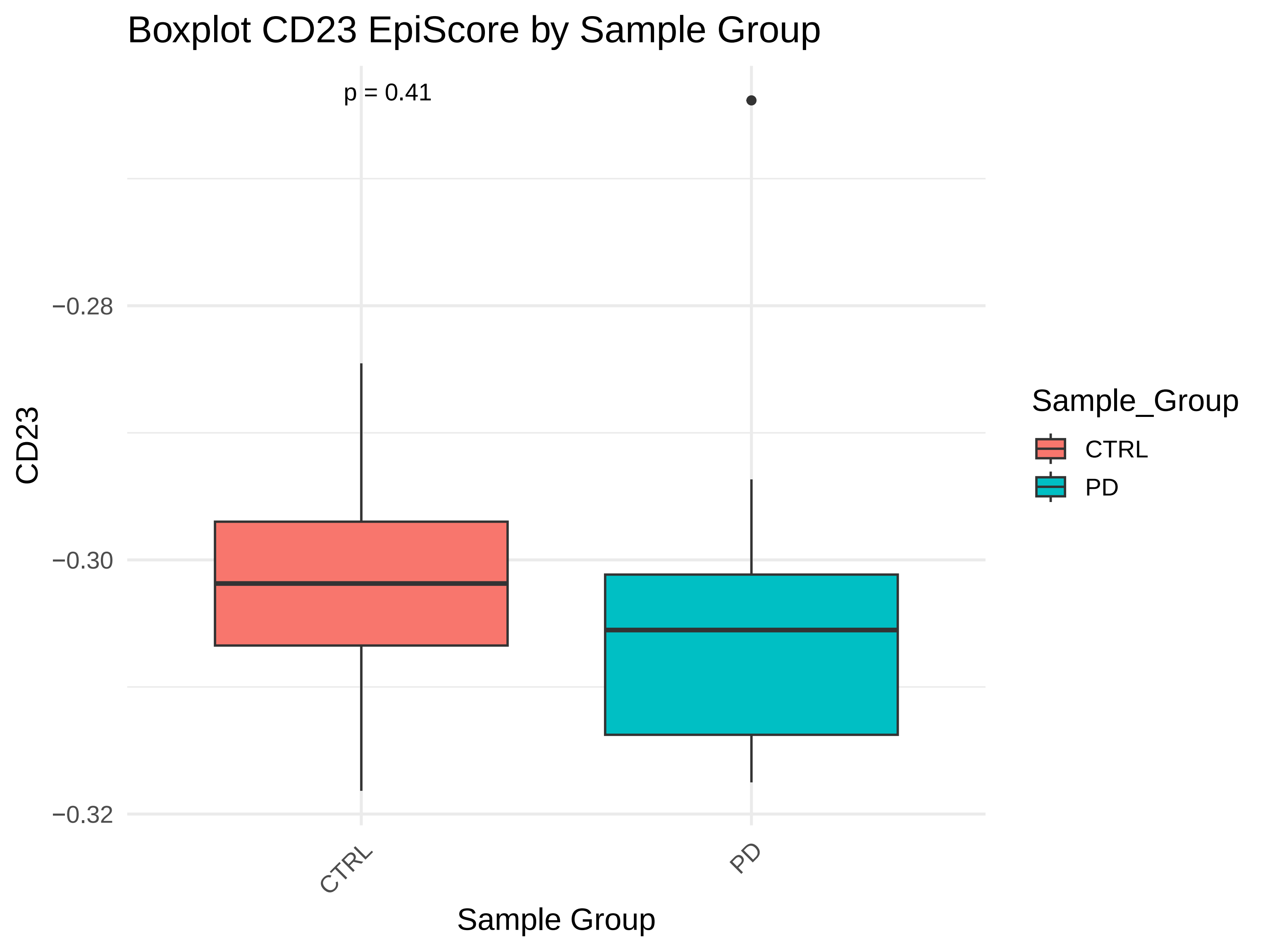

Boxplot CD23 EpiScore by Sample Group
p = 0.41
−0.28
−0.30
−0.32
CD23
CTRL
PD
Sample Group
Sample_Group
CTRL
PD

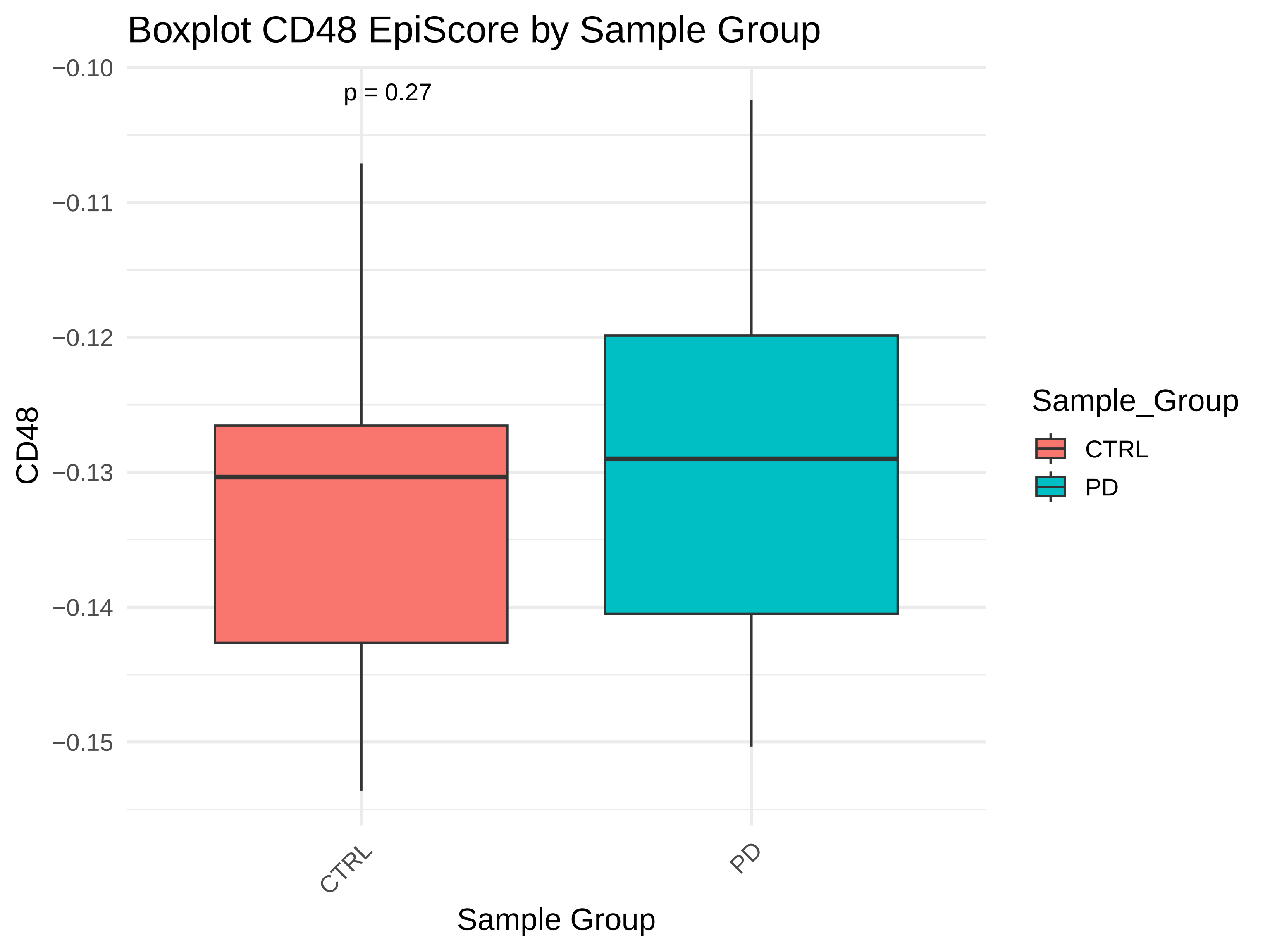

Boxplot CD48 EpiScore by Sample Group
p = 0.27
CD48
−0.10
−0.11
−0.12
−0.13
−0.14
−0.15
CTRL
PD
Sample Group
Sample_Group
CTRL
PD

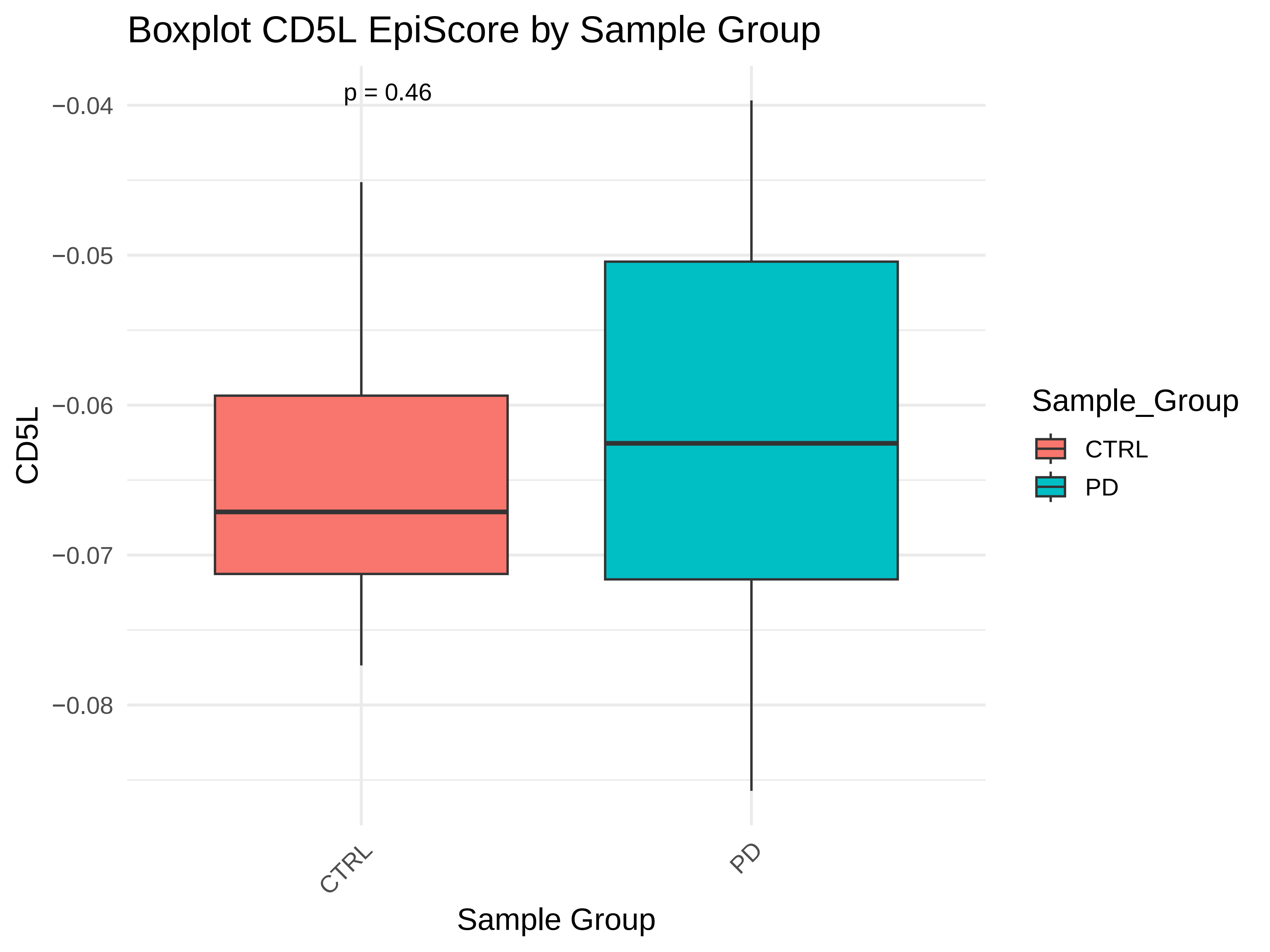

Boxplot CD5L EpiScore by Sample Group
p = 0.46
−0.04
−0.05
−0.06
−0.07
−0.08
CD5L
CTRL
PD
Sample Group
Sample_Group
CTRL
PD

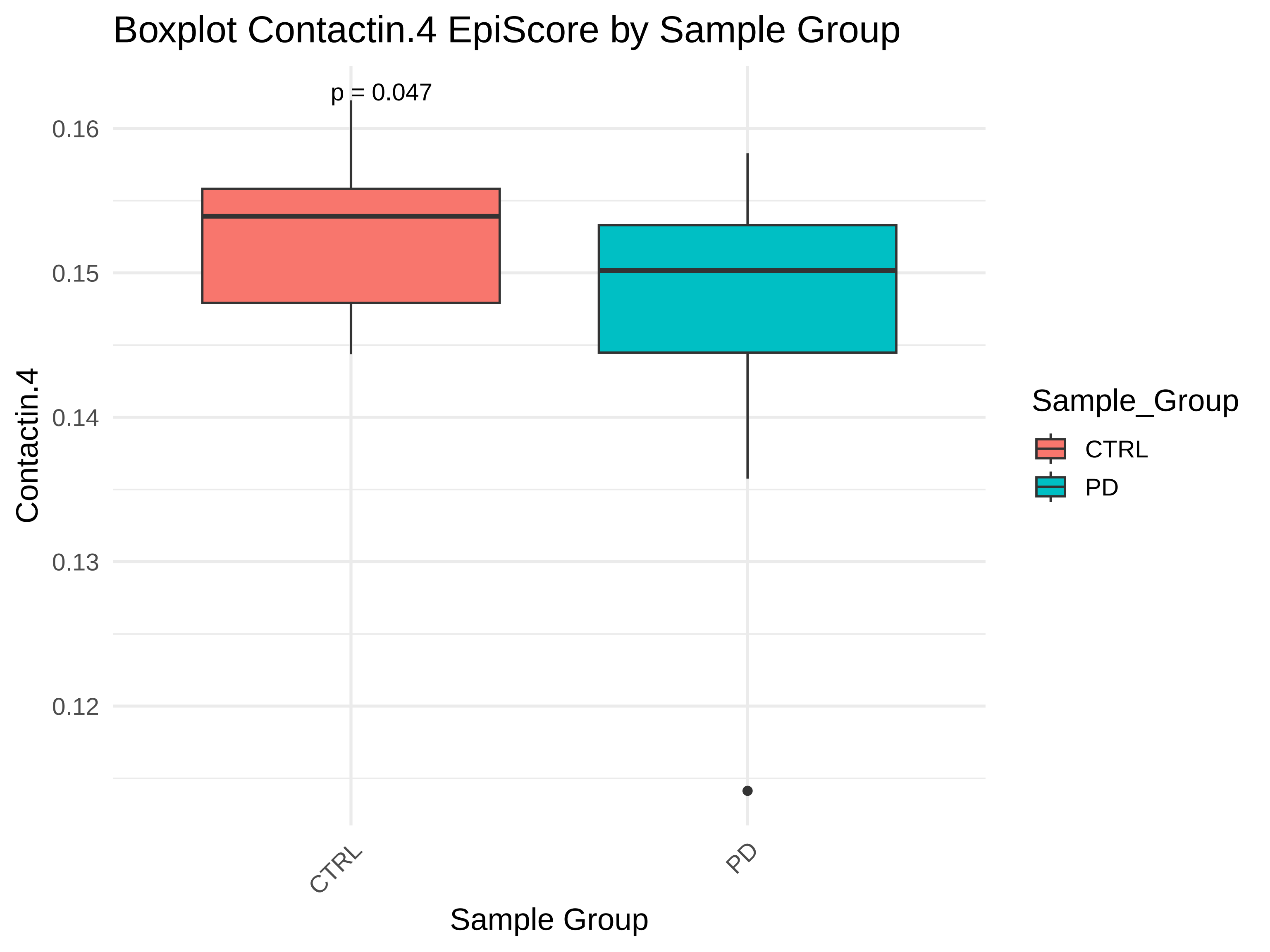

Boxplot Contactin.4 EpiScore by Sample Group
p = 0.047
0.16
0.15
0.14
0.13
0.12
Contactin.4
CTRL
PD
Sample Group
Sample_Group
CTRL
PD

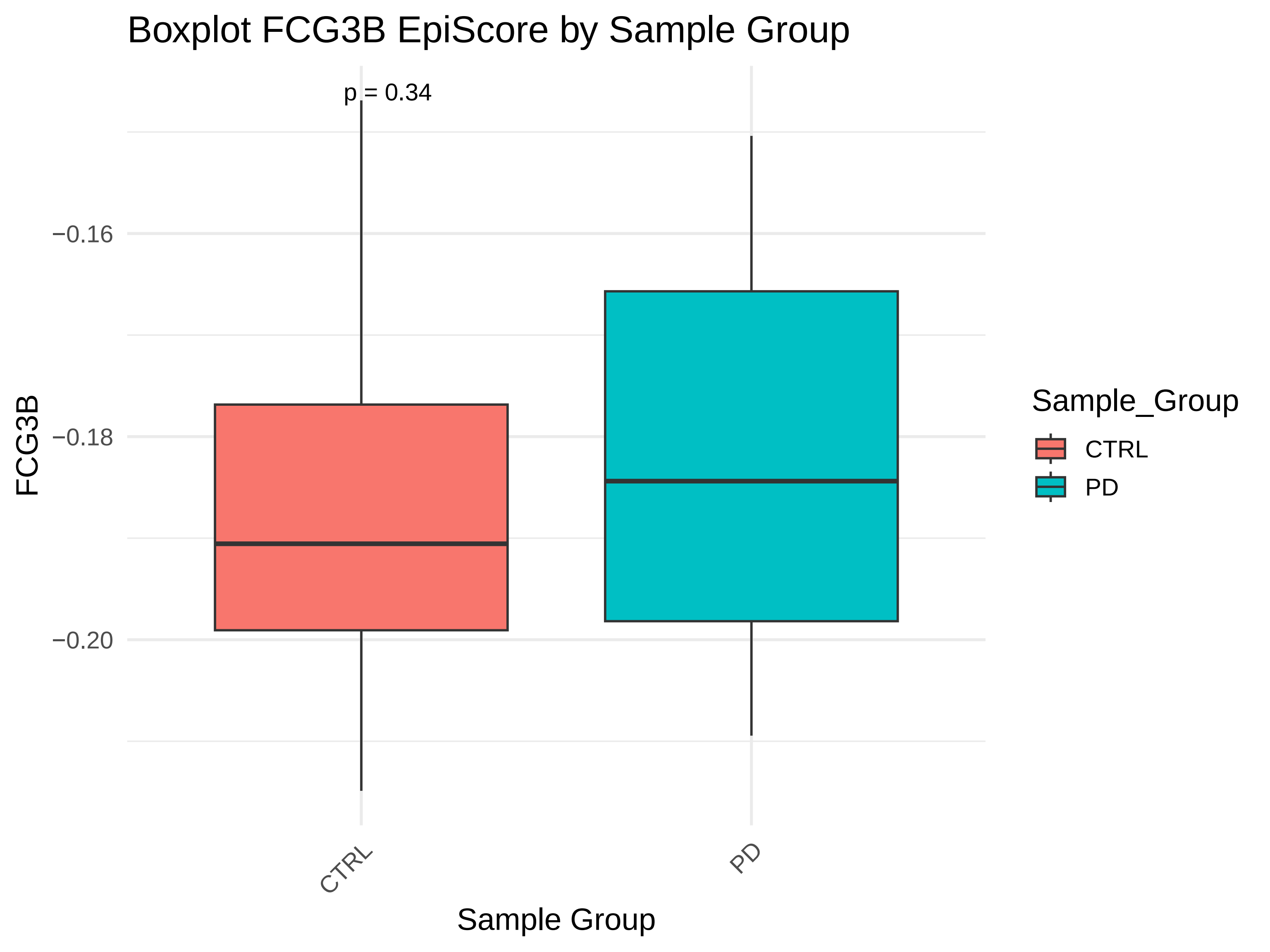
Boxplot FCG3B EpiScore by Sample Group
p = 0.34
−0.16
−0.18
−0.20
FCG3B
CTRL
PD
Sample Group
Sample_Group
CTRL
PD

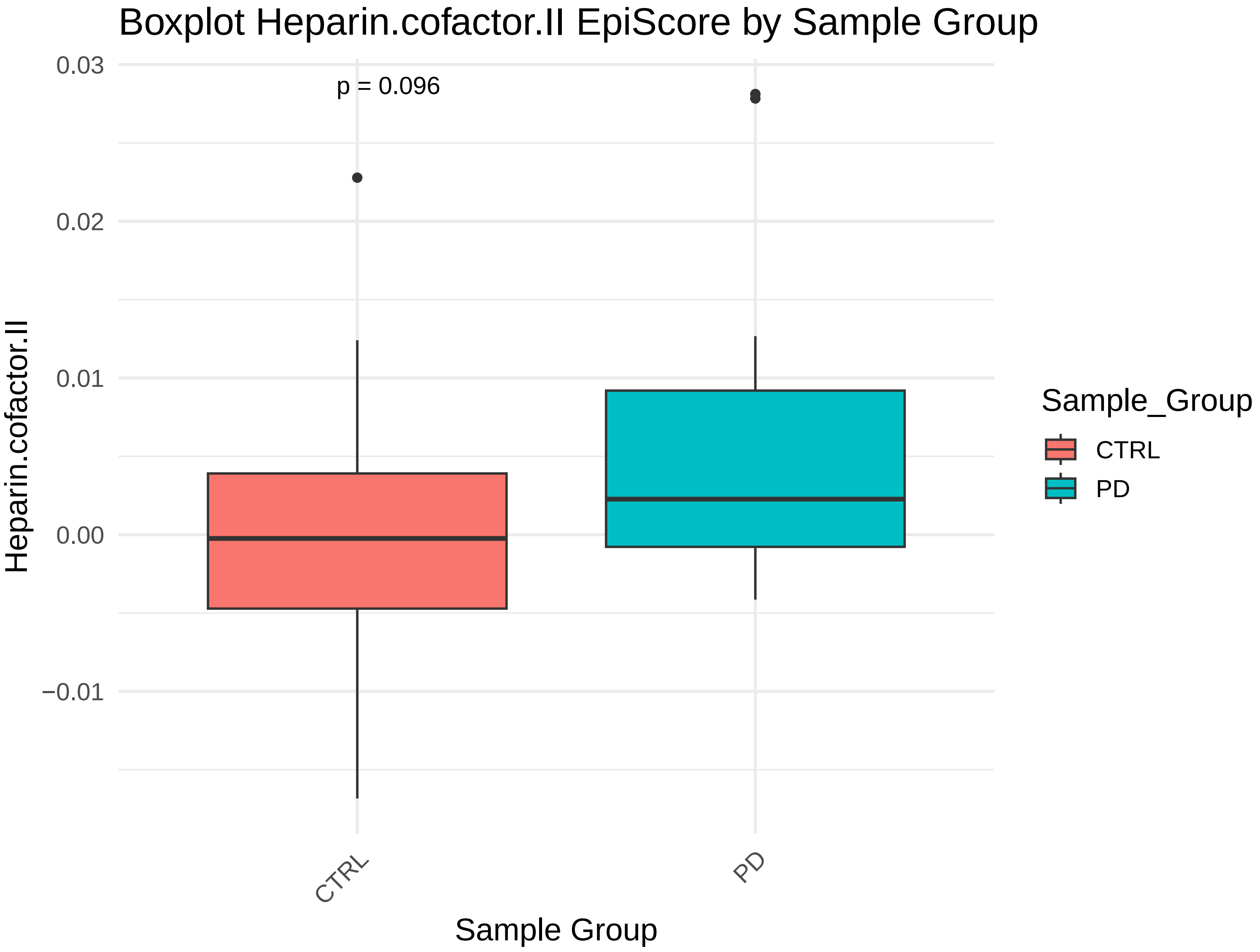

Boxplot Heparin.cofactor.II EpiScore by Sample Group
p = 0.096
0.03
0.02
0.01
0.00
−0.01
Heparin.cofactor.II
CTRL
PD
Sample Group
Sample_Group
CTRL
PD

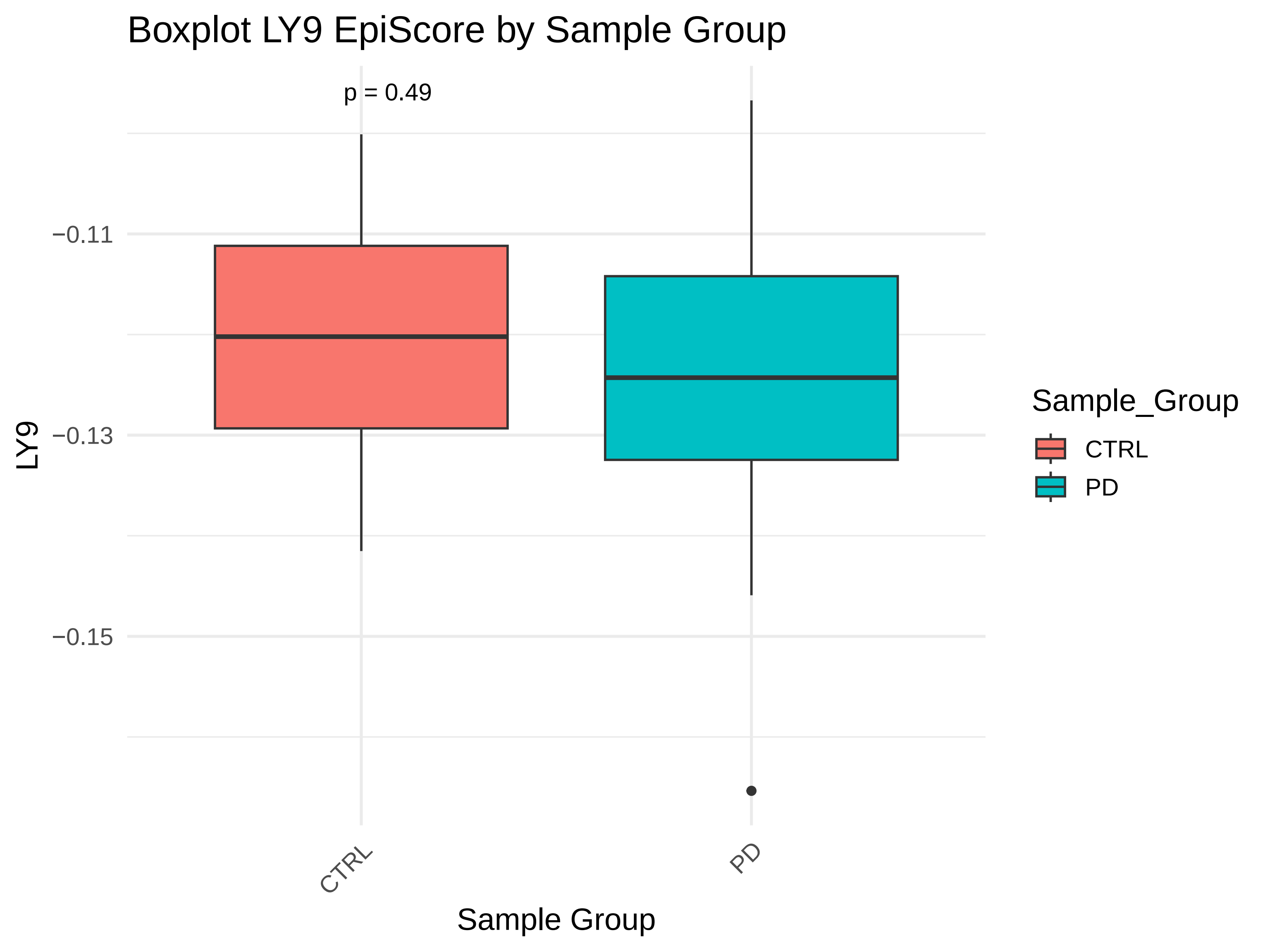
Boxplot LY9 EpiScore by Sample Group
p = 0.49
−0.11
−0.13
−0.15
LY9
CTRL
PD
Sample Group
Sample_Group
CTRL
PD

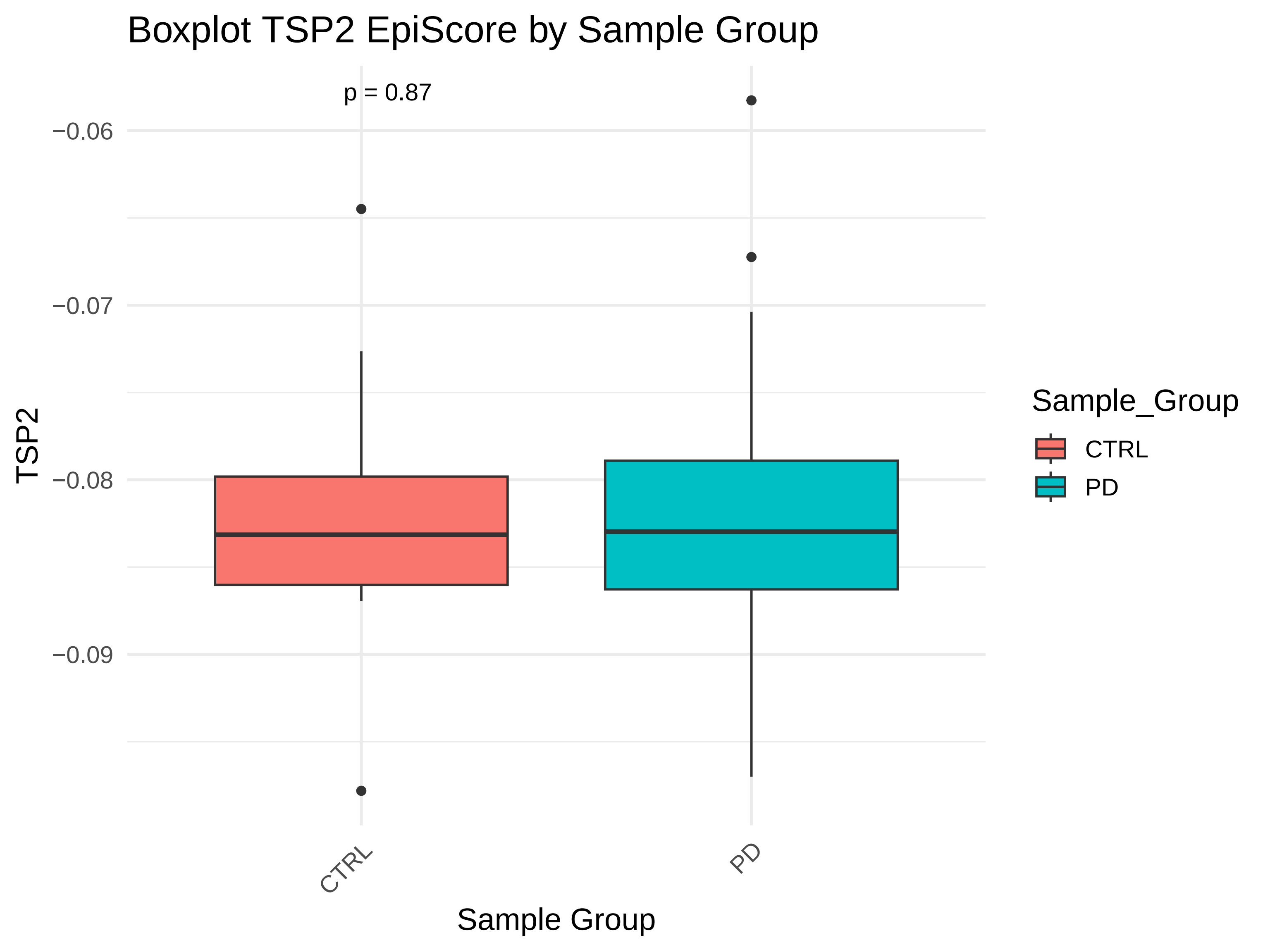

Boxplot TSP2 EpiScore by Sample Group
p = 0.87
−0.06
−0.07
−0.08
−0.09
TSP2
CTRL
PD
Sample Group
Sample_Group
CTRL
PD

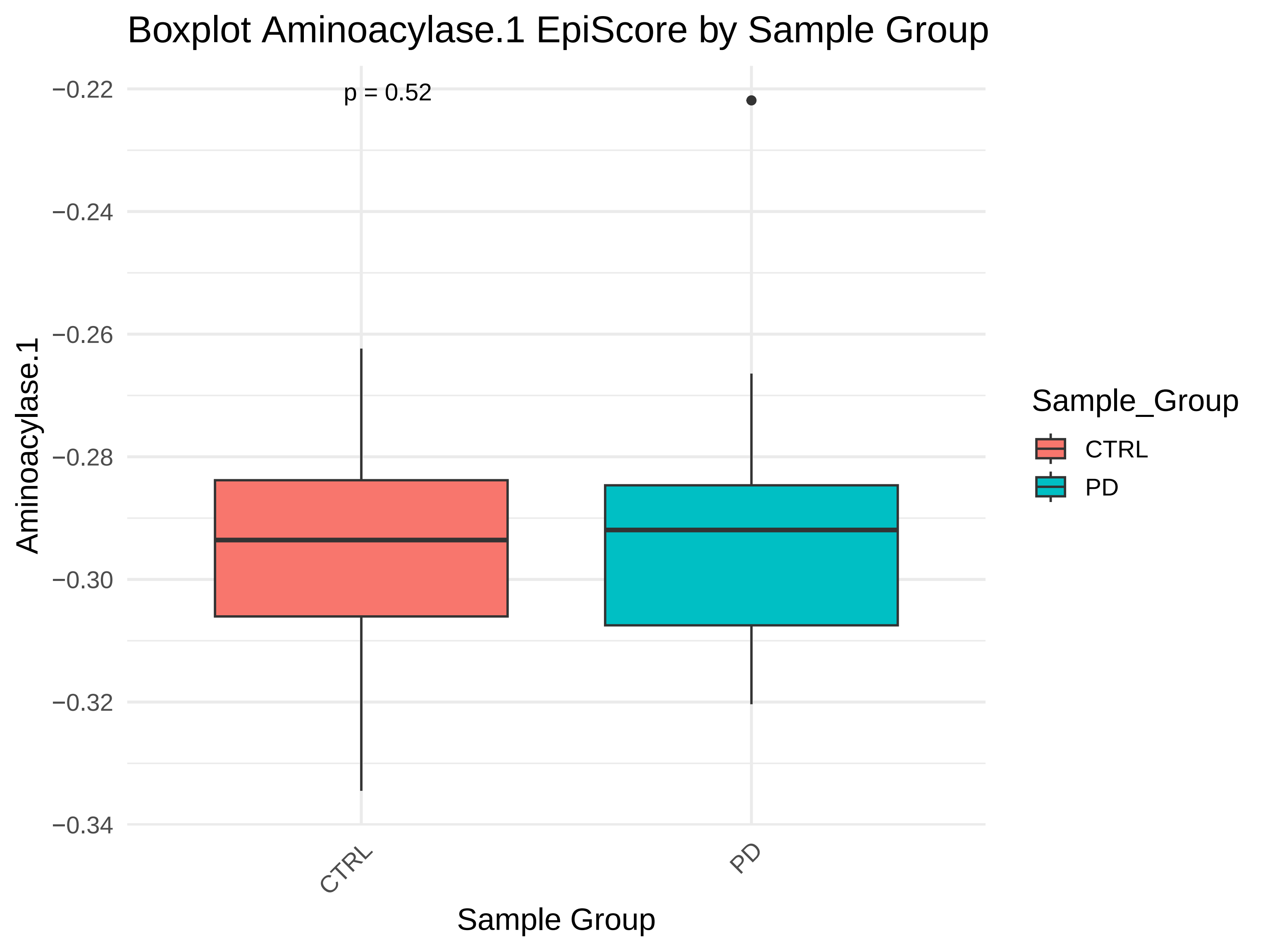

Boxplot Aminoacylase.1 EpiScore by Sample Group
p = 0.52
−0.22
−0.24
−0.26
−0.28
−0.30
−0.32
−0.34
Aminoacylase.1
CTRL
PD
Sample Group
Sample_Group
CTRL
PD

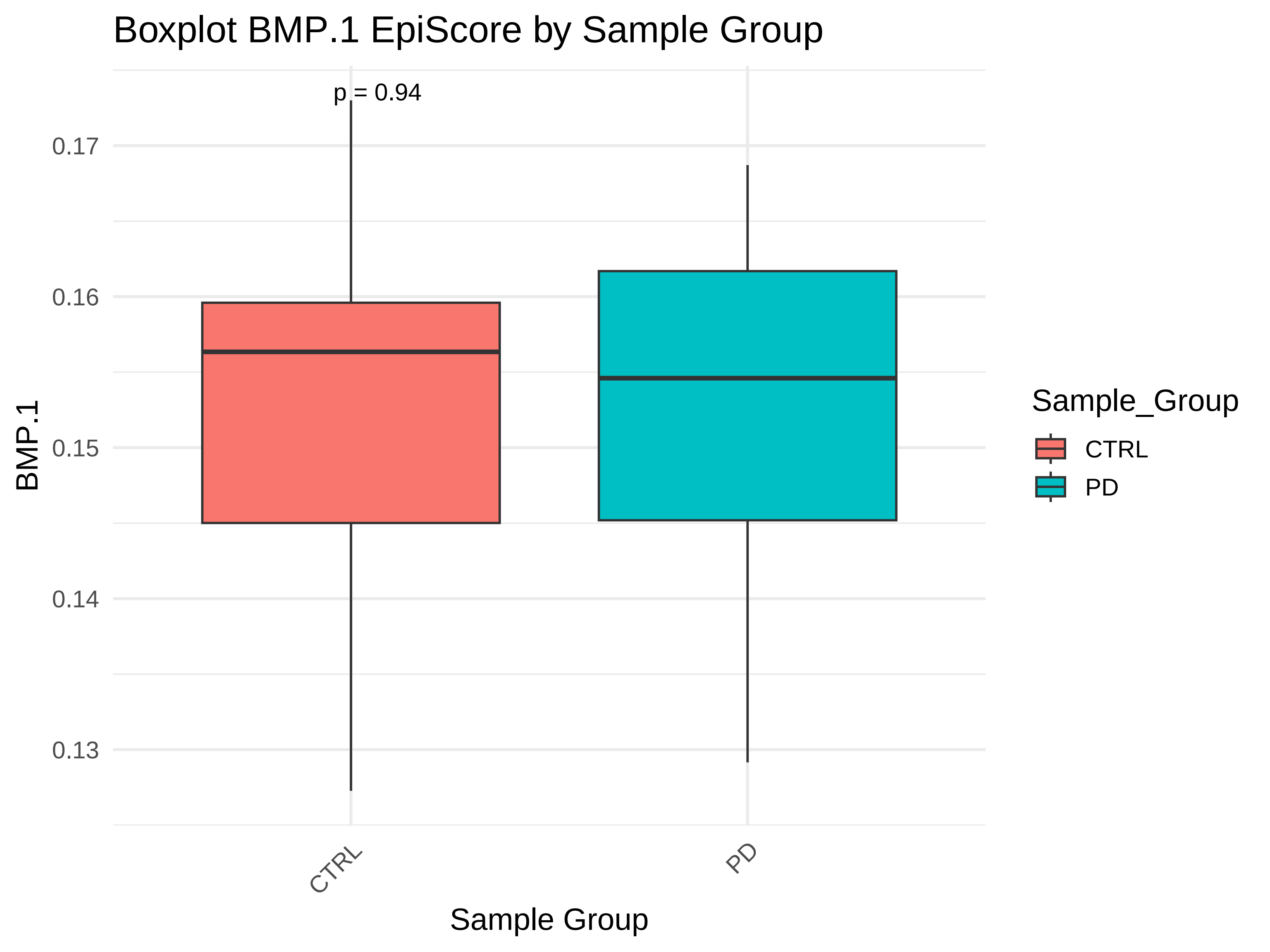
Boxplot BMP.1 EpiScore by Sample Group
p = 0.94
0.17
0.16
0.15
0.14
0.13
BMP.1
CTRL
PD
Sample Group
Sample_Group
CTRL
PD

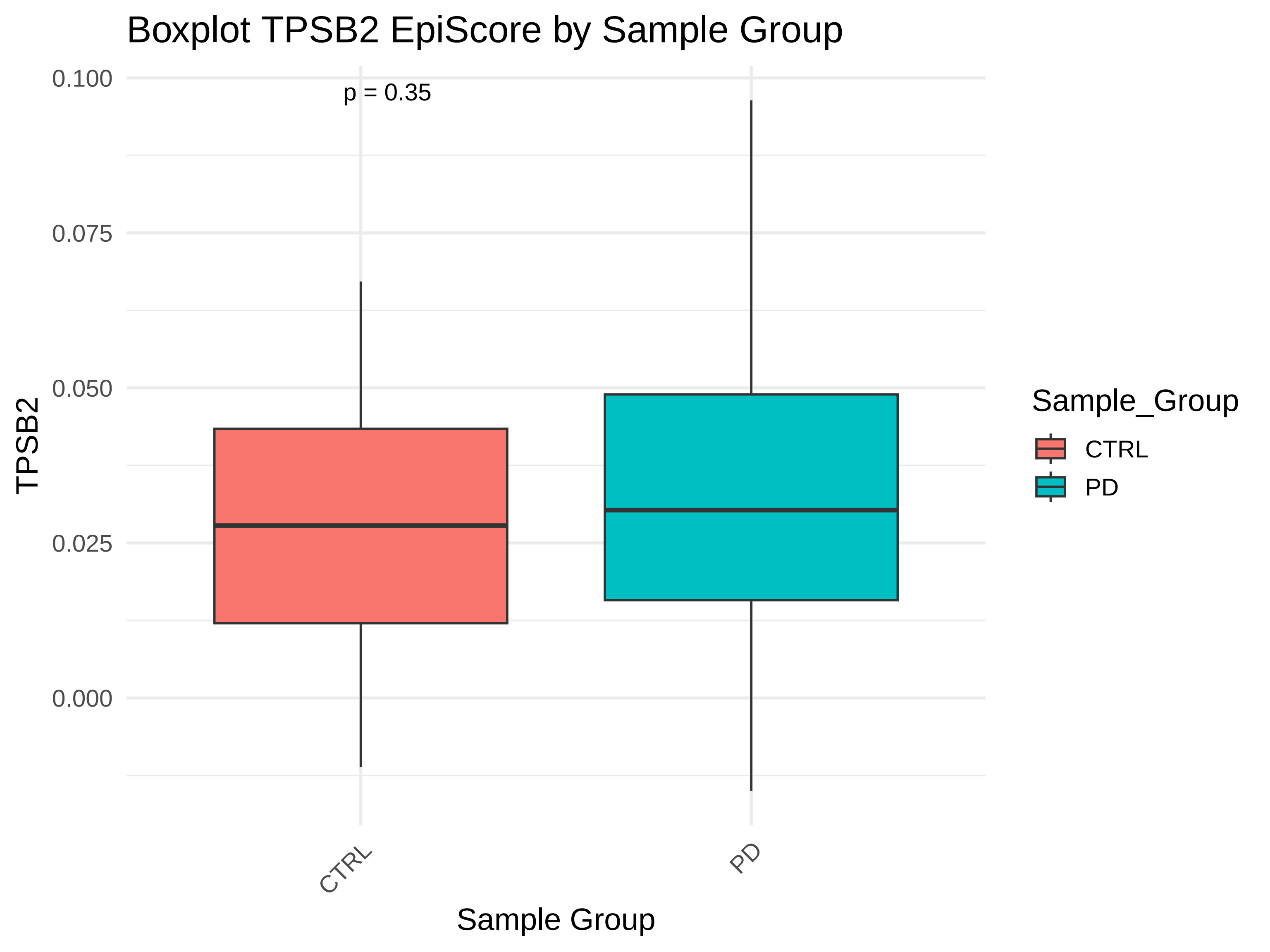

Boxplot TPSB2 EpiScore by Sample Group
p = 0.35
0.100
0.075
0.050
0.025
0.000
TPSB2
CTRL
PD
Sample Group
Sample_Group
CTRL
PD

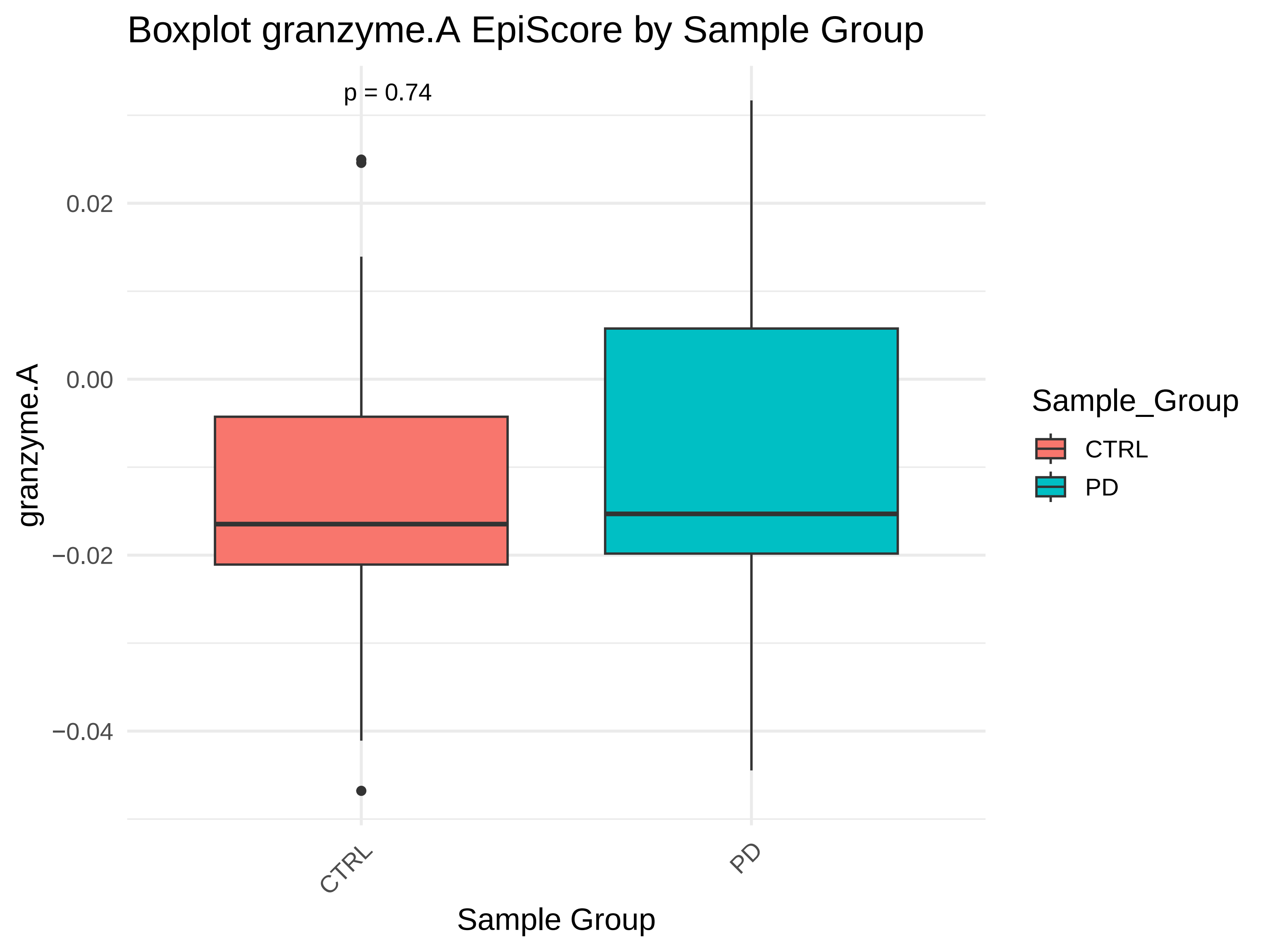

Boxplot granzyme.A EpiScore by Sample Group
p = 0.74
0.02
0.00
−0.02
−0.04
granzyme.A
CTRL
PD
Sample Group
Sample_Group
CTRL
PD

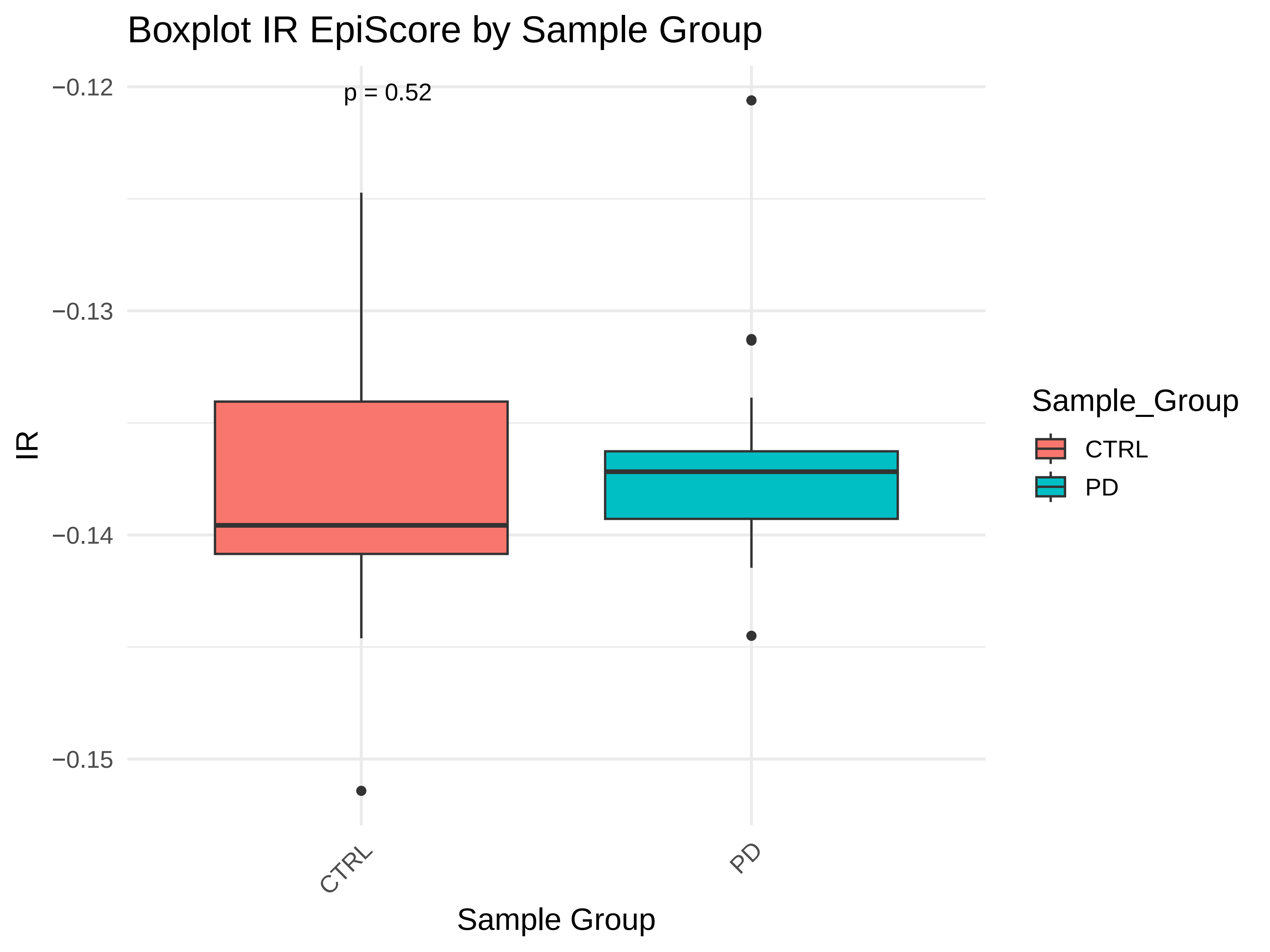
Boxplot IR EpiScore by Sample Group
p = 0.52
−0.12
−0.13
−0.14
−0.15
IR
CTRL
PD
Sample Group
Sample_Group
CTRL
PD

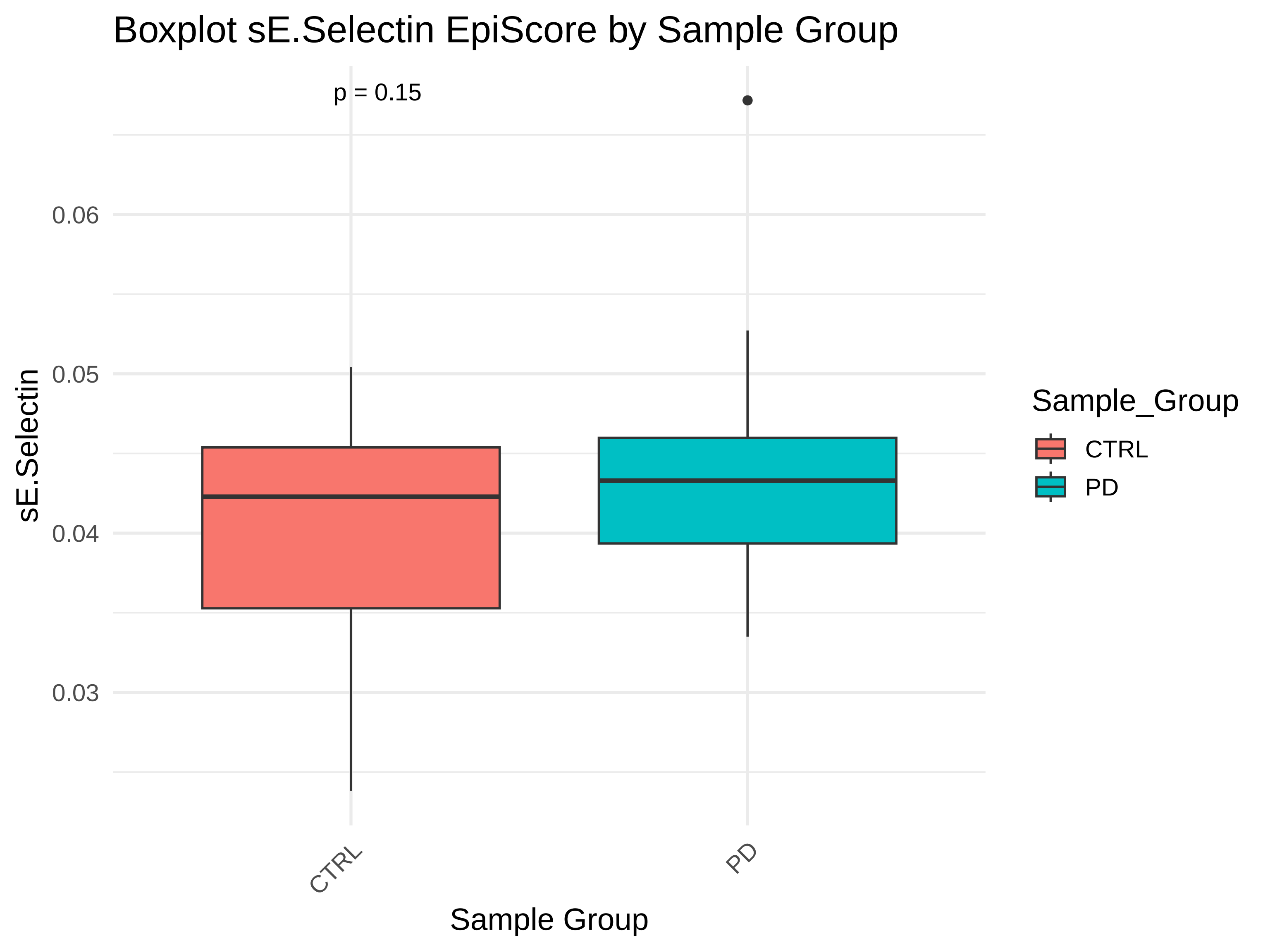
Boxplot sE.Selectin EpiScore by Sample Group
p = 0.15
0.06
0.05
0.04
0.03
sE.Selectin
CTRL
PD
Sample Group
Sample_Group
CTRL
PD

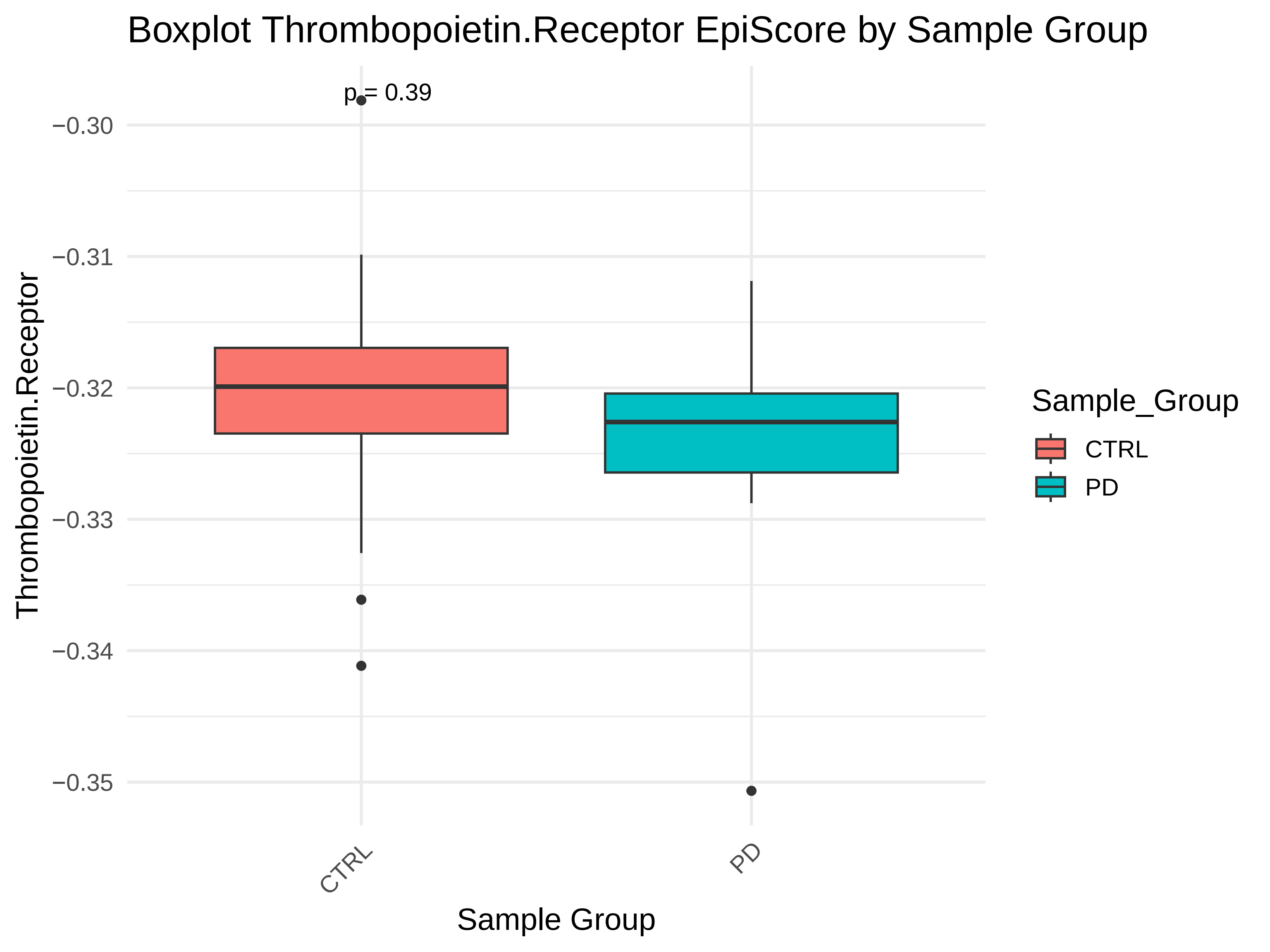

Boxplot Thrombopoietin.Receptor EpiScore by Sample Group
p = 0.39
−0.30
−0.31
−0.32
−0.33
−0.34
−0.35
Thrombopoietin.Receptor
CTRL
PD
Sample Group
Sample_Group
CTRL
PD

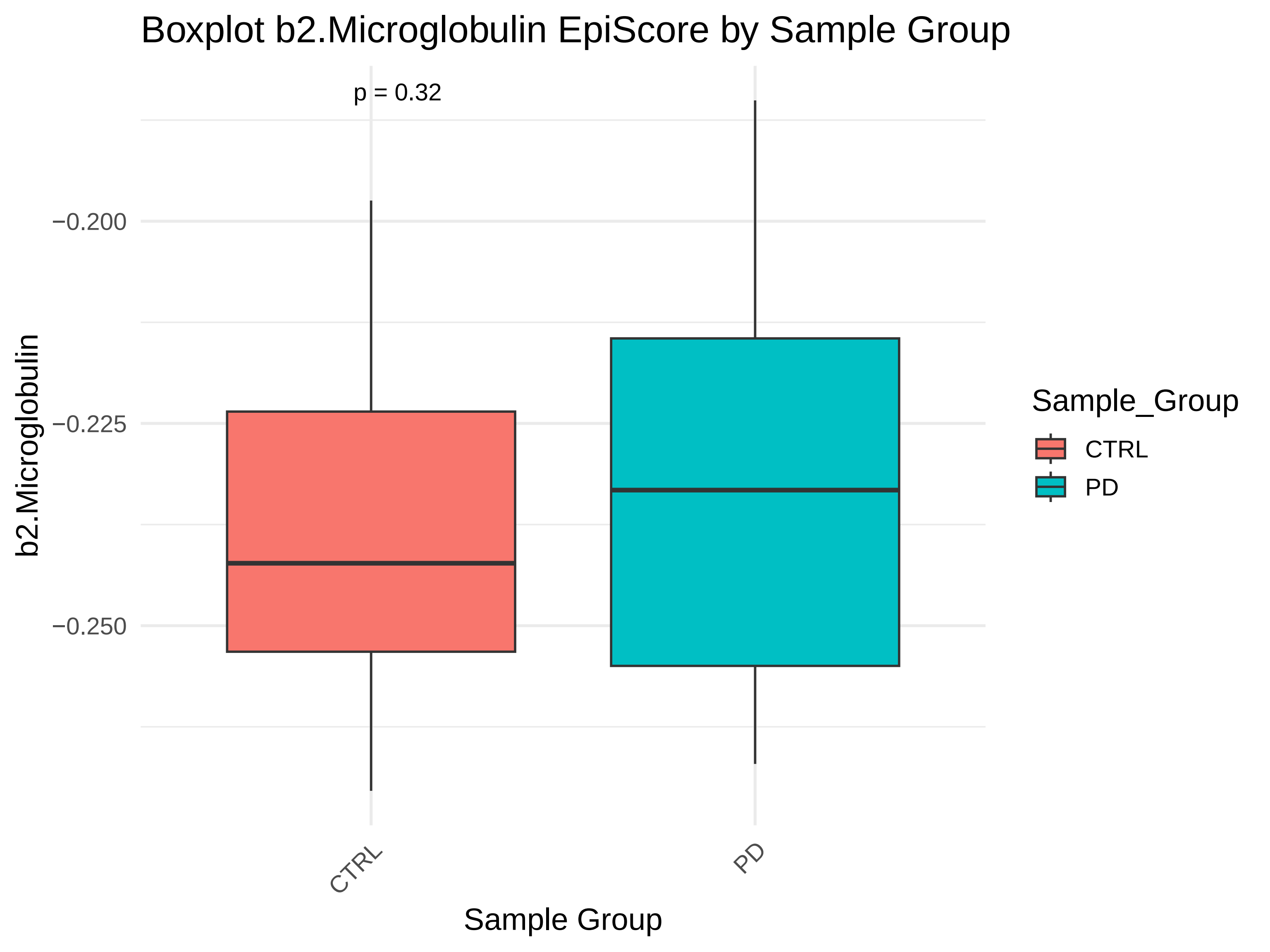
Boxplot b2.Microglobulin EpiScore by Sample Group
p = 0.32
−0.200
−0.225
−0.250
b2.Microglobulin
CTRL
PD
Sample Group
Sample_Group
CTRL
PD

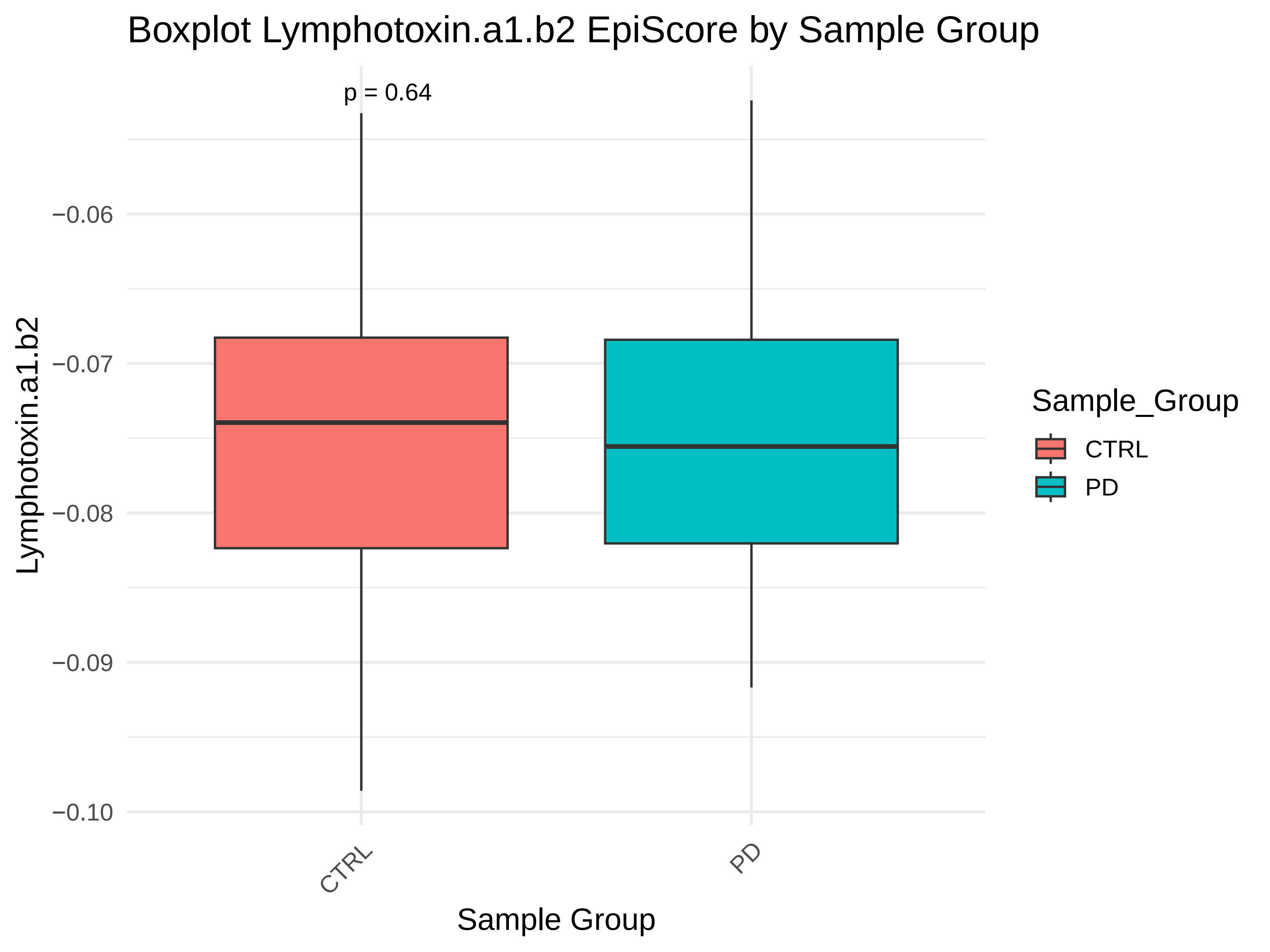

Boxplot Lymphotoxin.a1.b2 EpiScore by Sample Group
p = 0.64
−0.06
−0.07
−0.08
−0.09
−0.10
Lymphotoxin.a1.b2
CTRL
PD
Sample Group
Sample_Group
CTRL
PD

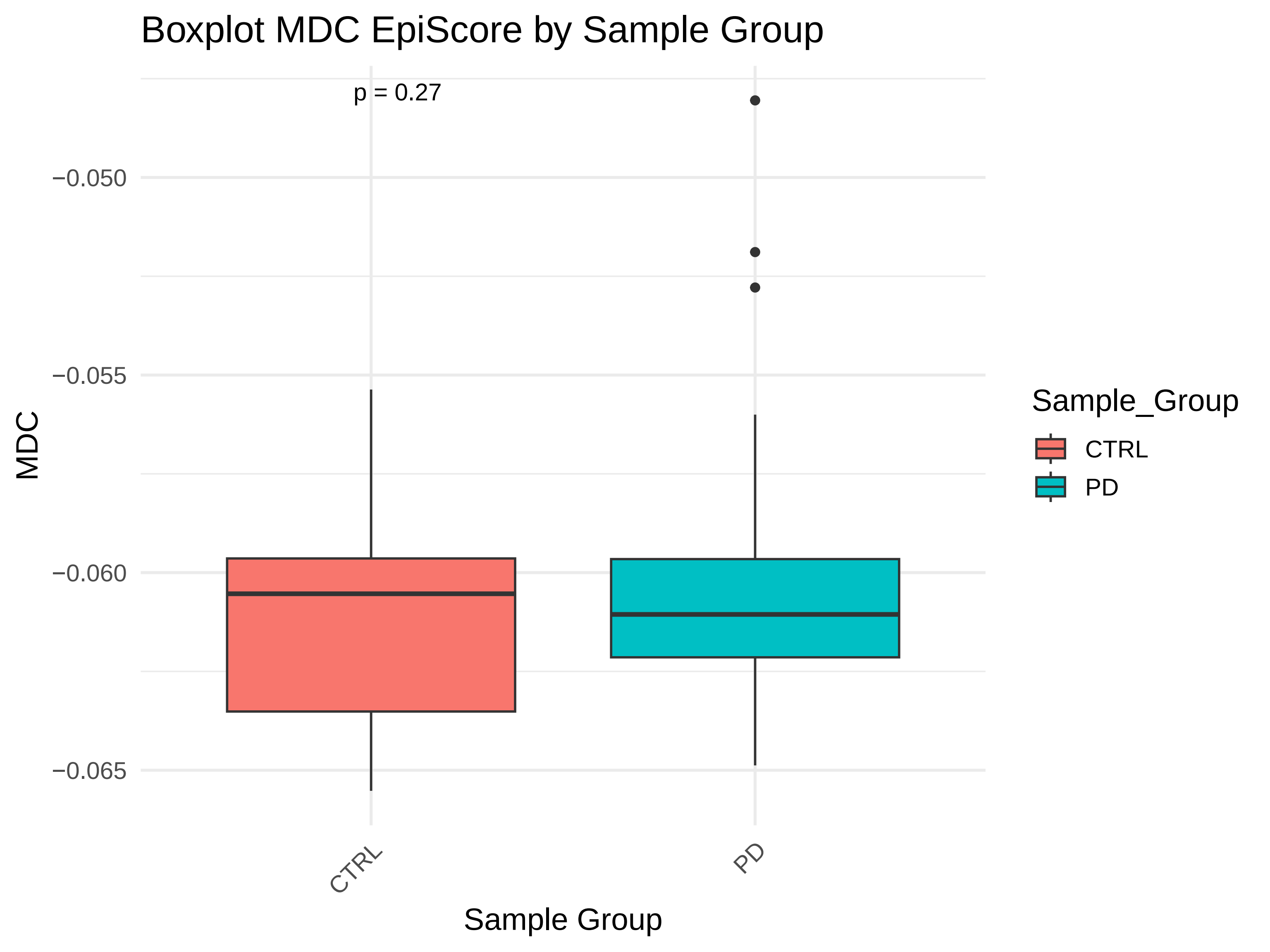

Boxplot MDC EpiScore by Sample Group
p = 0.27
−0.050
−0.055
−0.060
−0.065
MDC
CTRL
PD
Sample Group
Sample_Group
CTRL
PD

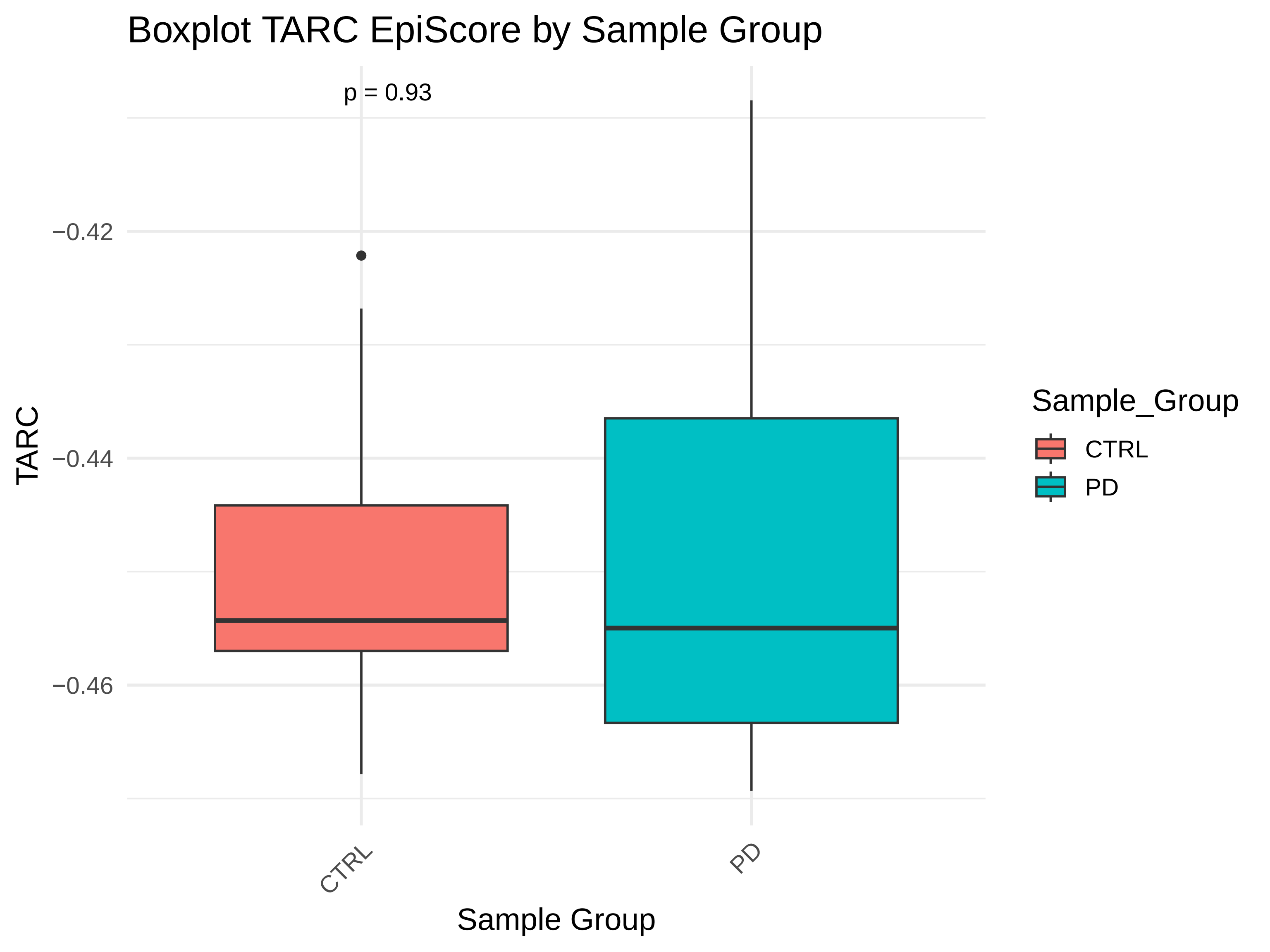

Boxplot TARC EpiScore by Sample Group
p = 0.93
TARC
−0.42
−0.44
−0.46
CTRL
PD
Sample Group
Sample_Group
CTRL
PD

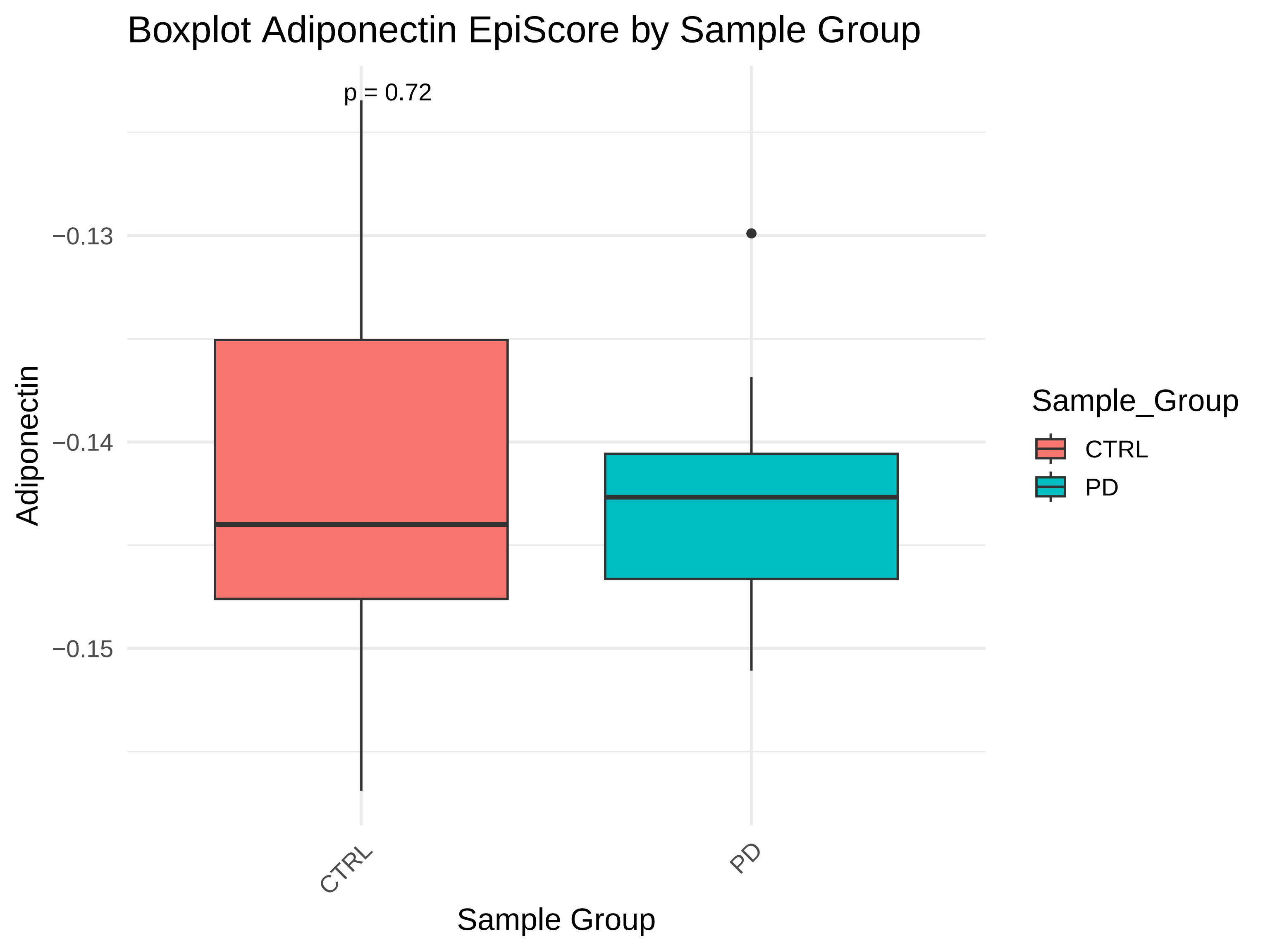

Boxplot Adiponectin EpiScore by Sample Group
p = 0.72
−0.13
−0.14
−0.15
Adiponectin
CTRL
PD
Sample Group
Sample_Group
CTRL
PD

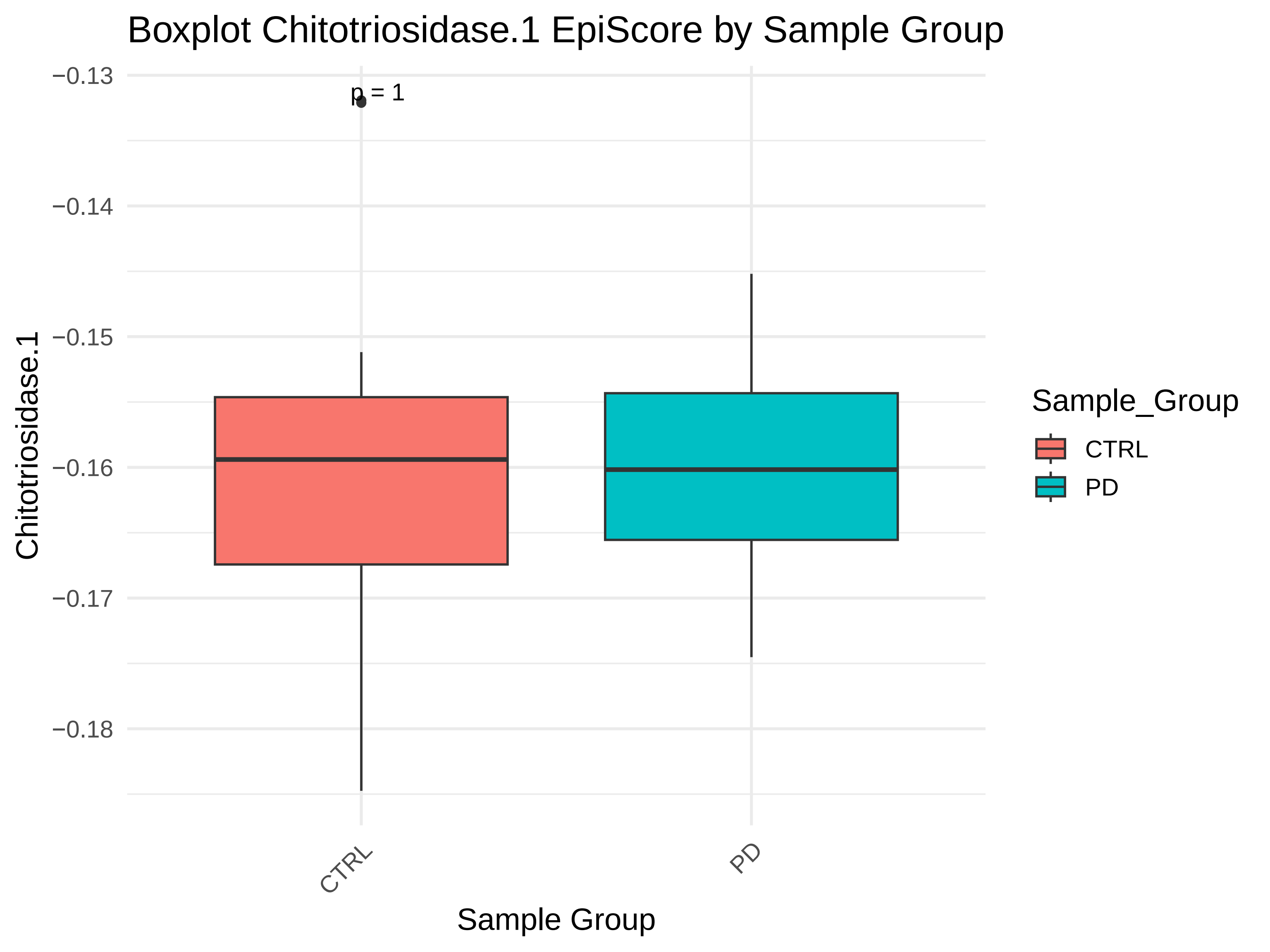

Boxplot Chitotriosidase.1 EpiScore by Sample Group
p = 1
−0.13
−0.14
−0.15
−0.16
−0.17
−0.18
Chitotriosidase.1
CTRL
PD
Sample Group
Sample_Group
CTRL
PD

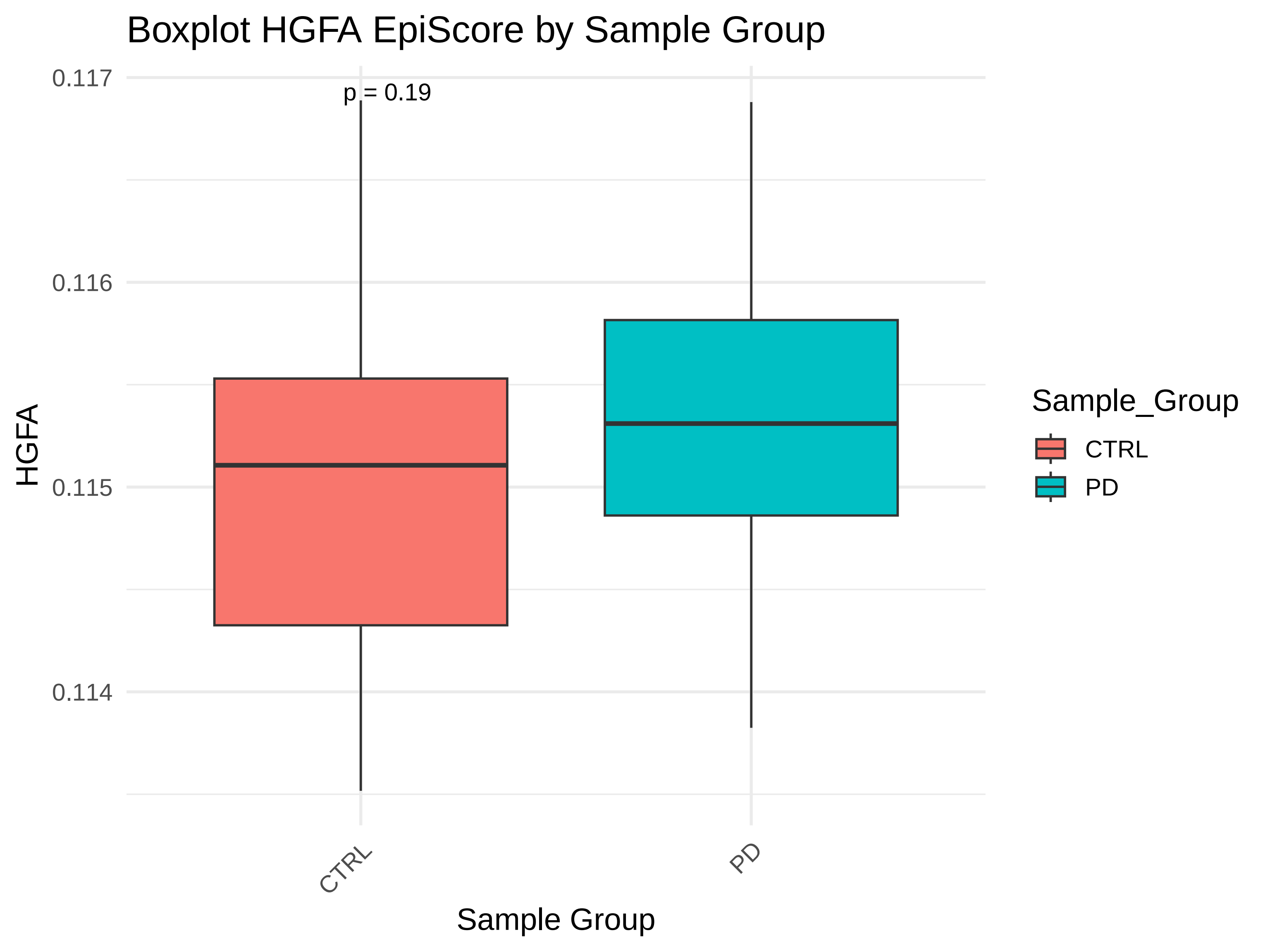

Boxplot HGFA EpiScore by Sample Group
p = 0.19
0.117
0.116
0.115
0.114
HGFA
CTRL
PD
Sample Group
Sample_Group
CTRL
PD

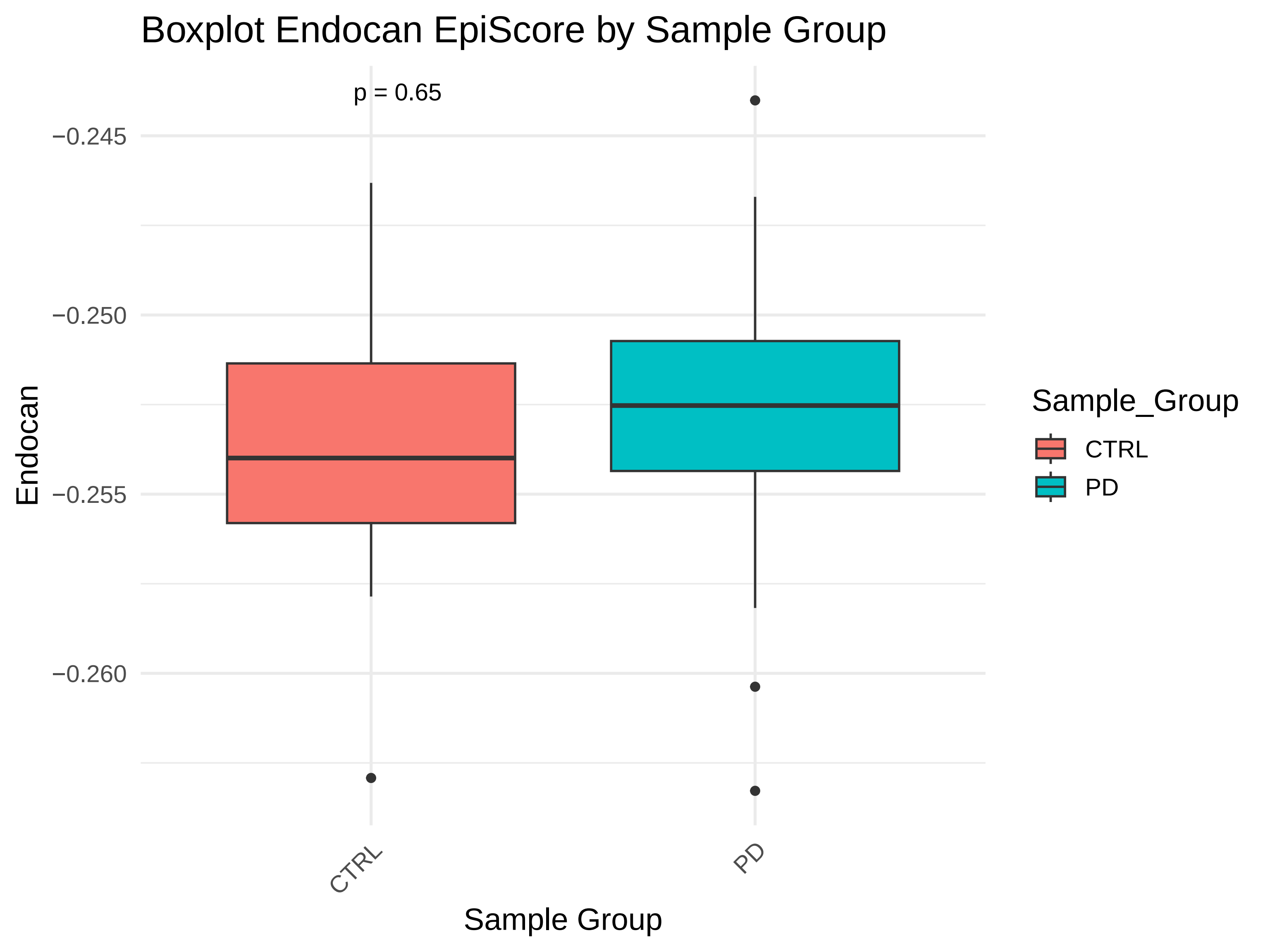
Boxplot Endocan EpiScore by Sample Group
p = 0.65
−0.245
−0.250
−0.255
−0.260
Endocan
CTRL
PD
Sample Group
Sample_Group
CTRL
PD

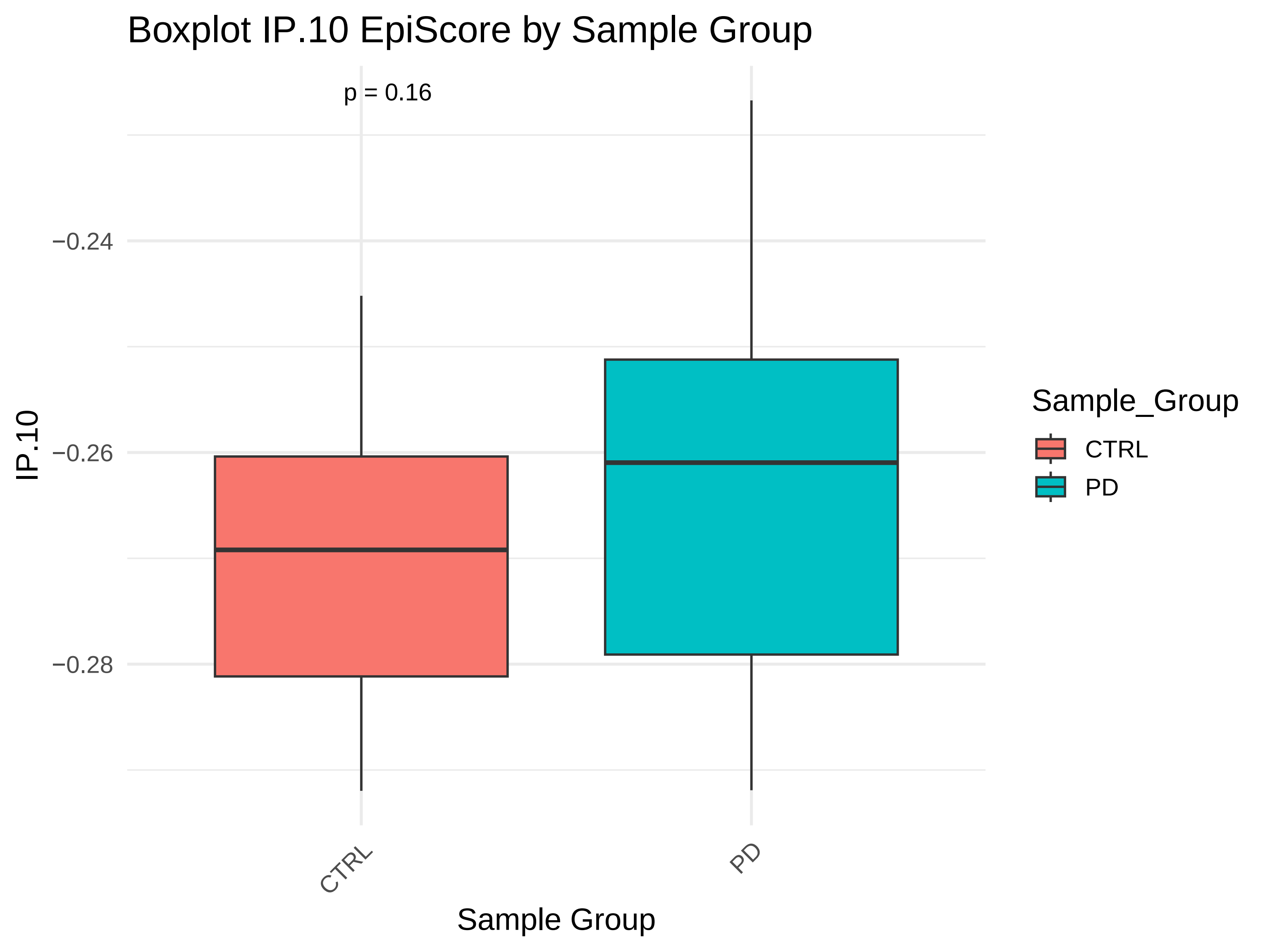

Boxplot IP.10 EpiScore by Sample Group
p = 0.16
−0.24
−0.26
−0.28
IP.10
CTRL
PD
Sample Group
Sample_Group
CTRL
PD

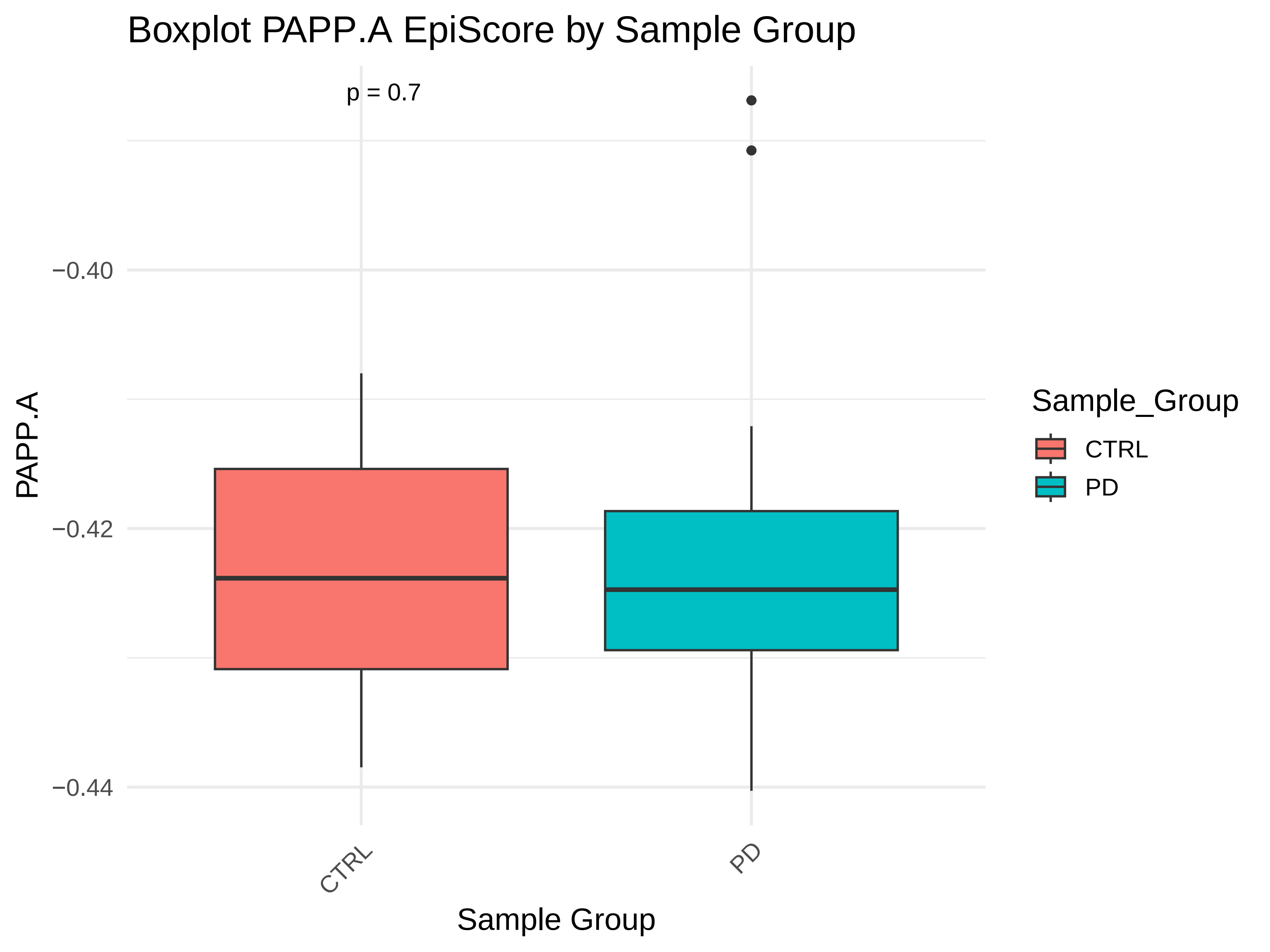

Boxplot PAPP.A EpiScore by Sample Group
p = 0.7
−0.40
−0.42
−0.44
PAPP.A
CTRL
PD
Sample Group
Sample_Group
CTRL
PD

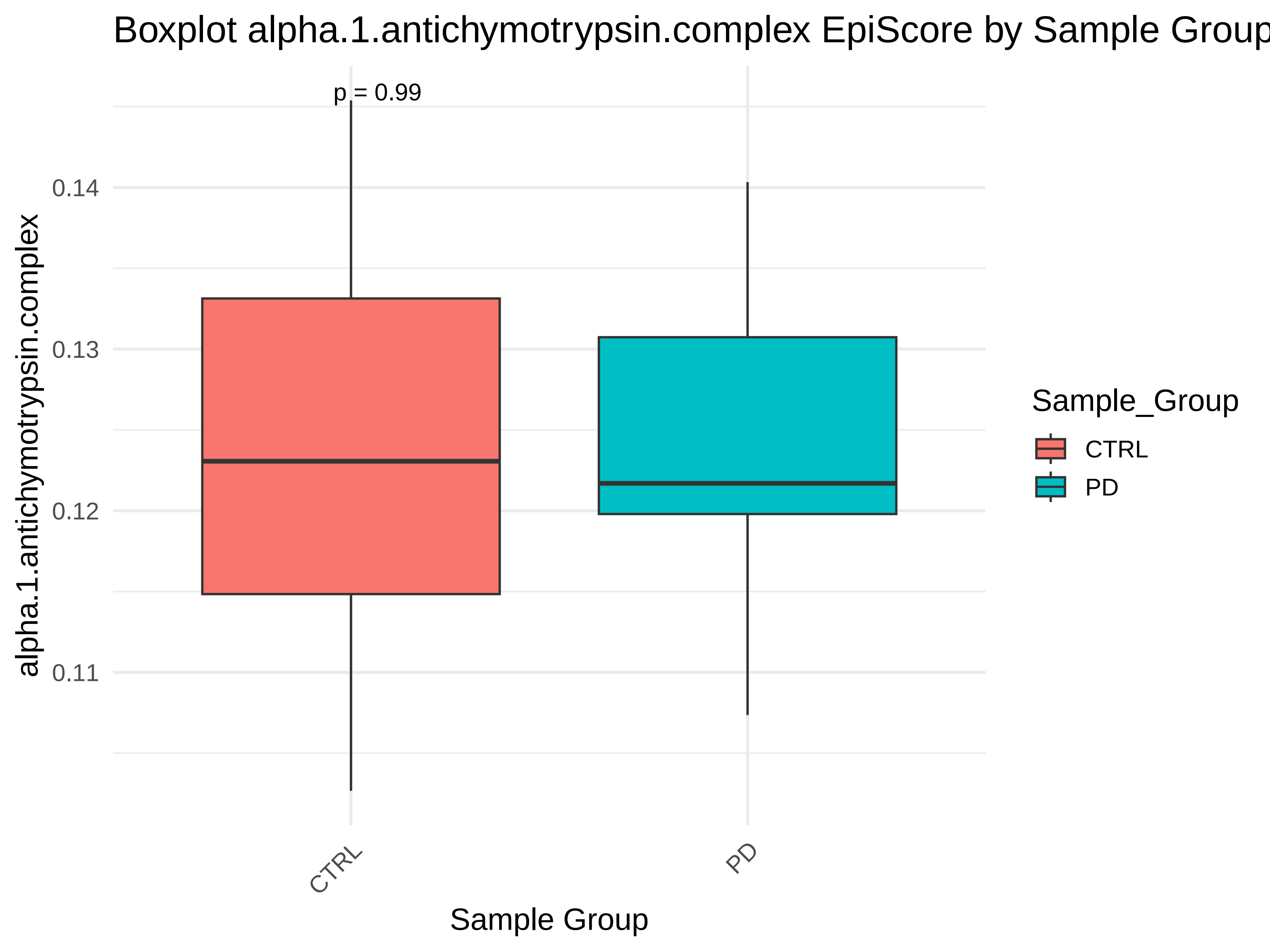

Boxplot alpha.1.antichymotrypsin.complex EpiScore by Sample Group
p = 0.99
0.14
0.13
0.12
0.11
alpha.1.antichymotrypsin.complex
CTRL
PD
Sample Group
Sample_Group
CTRL
PD

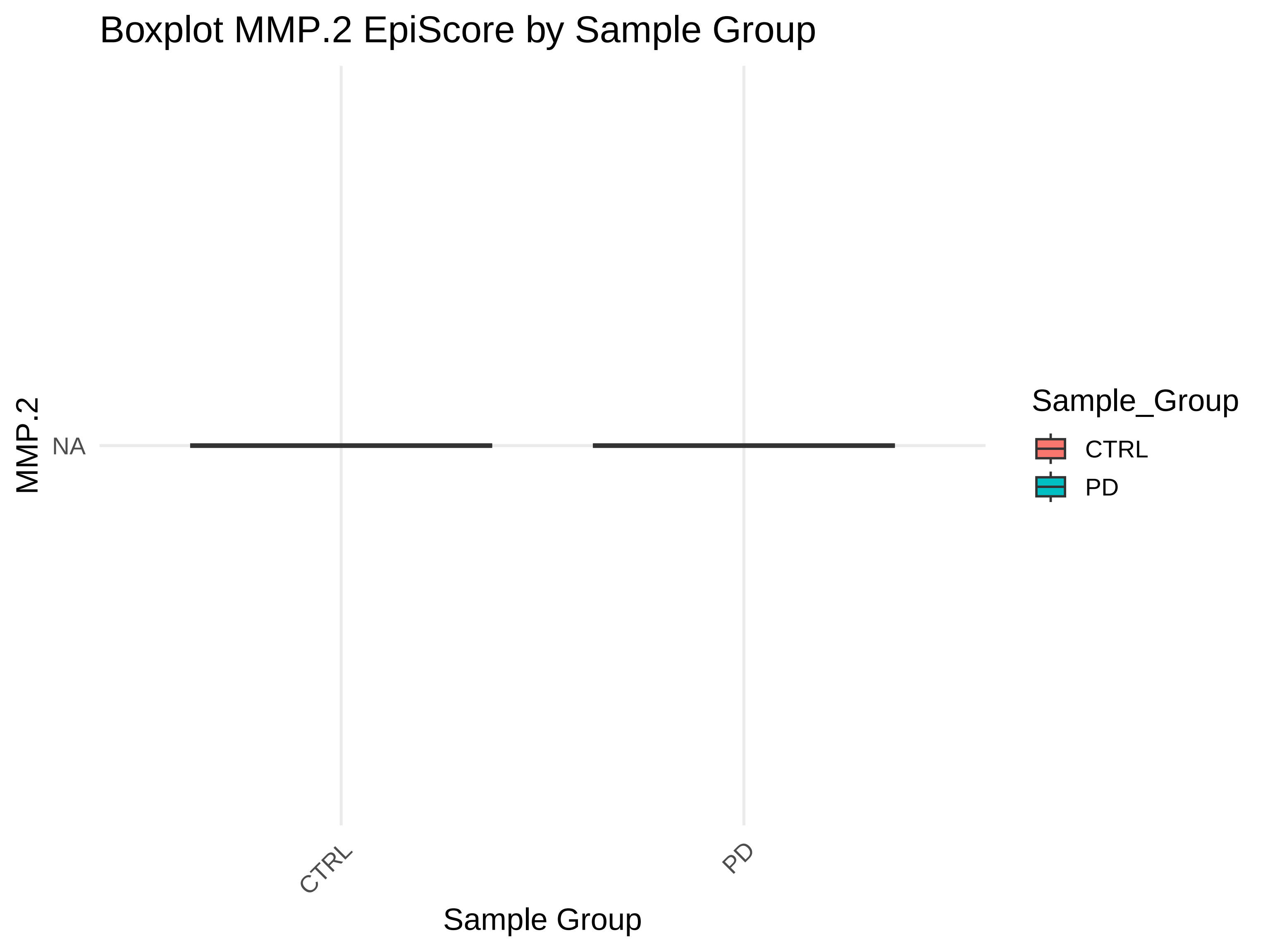
Boxplot MMP.2 EpiScore by Sample Group
MMP.2
NA
CTRL
PD
Sample Group
Sample_Group
CTRL
PD

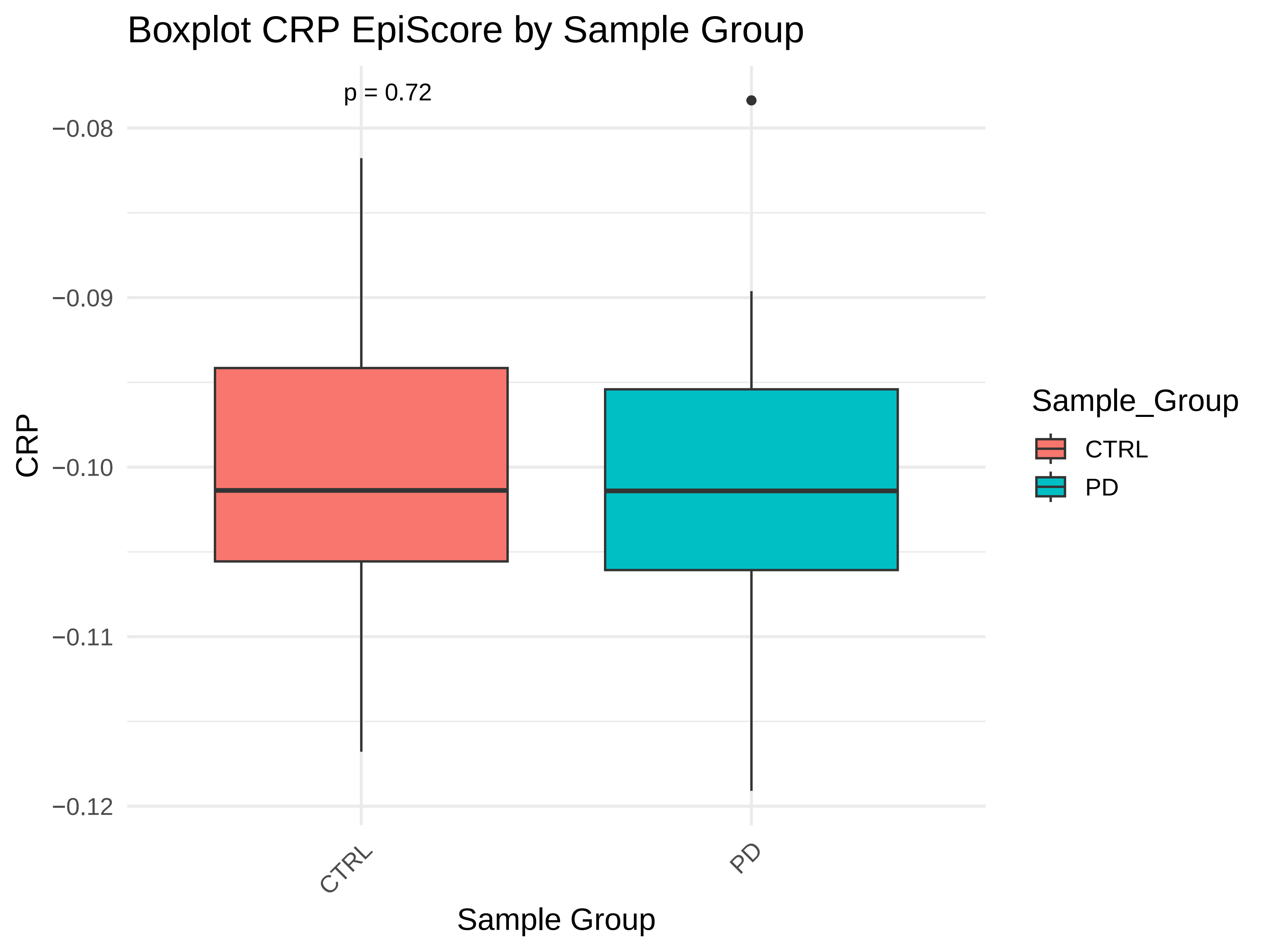
Boxplot CRP EpiScore by Sample Group
p = 0.72
−0.08
−0.09
−0.10
−0.11
−0.12
CRP
CTRL
PD
Sample Group
Sample_Group
CTRL
PD

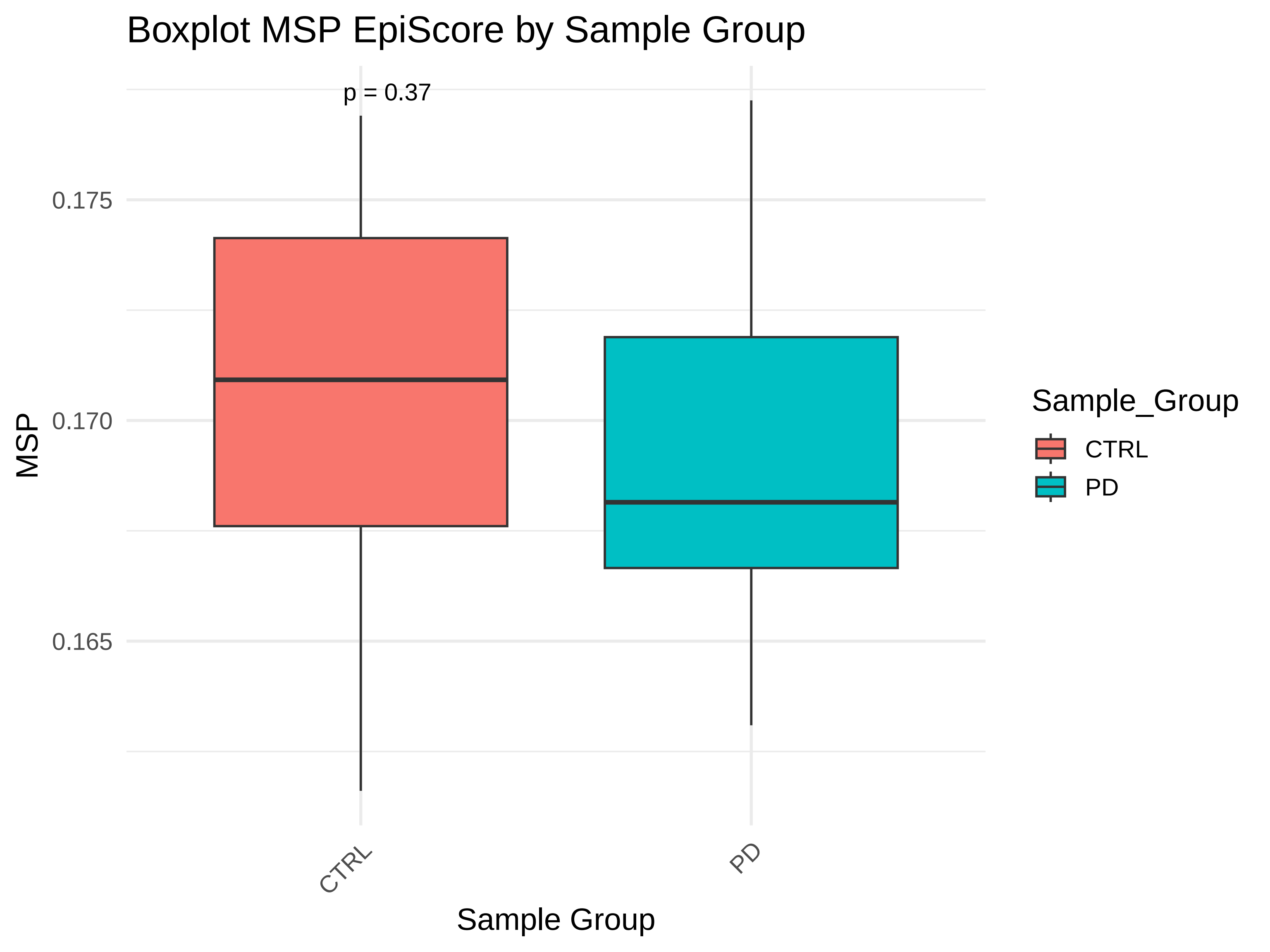

Boxplot MSP EpiScore by Sample Group
p = 0.37
0.175
0.170
0.165
MSP
CTRL
PD
Sample Group
Sample_Group
CTRL
PD

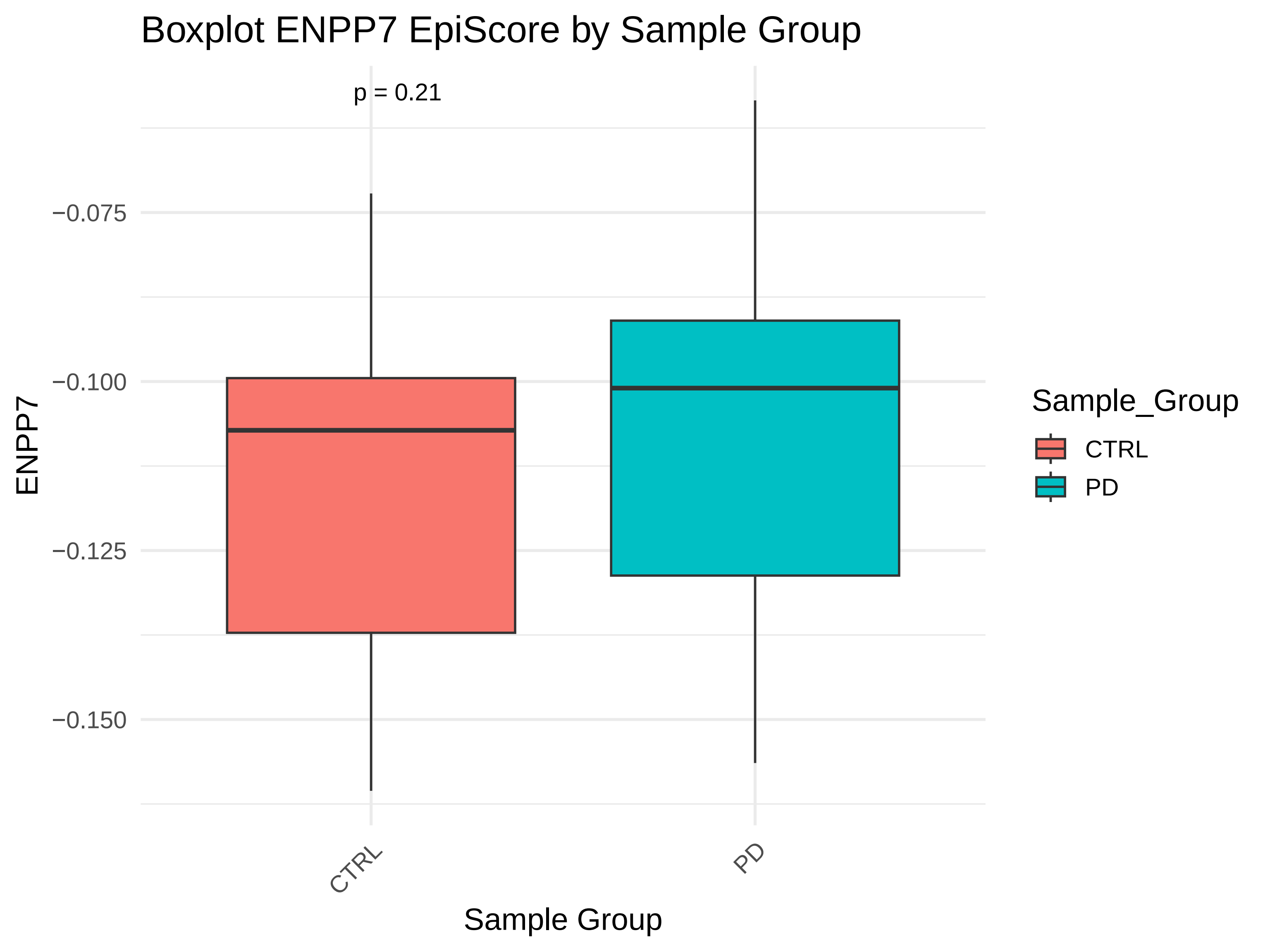

Boxplot ENPP7 EpiScore by Sample Group
p = 0.21
−0.075
−0.100
−0.125
−0.150
ENPP7
CTRL
PD
Sample Group
Sample_Group
CTRL
PD

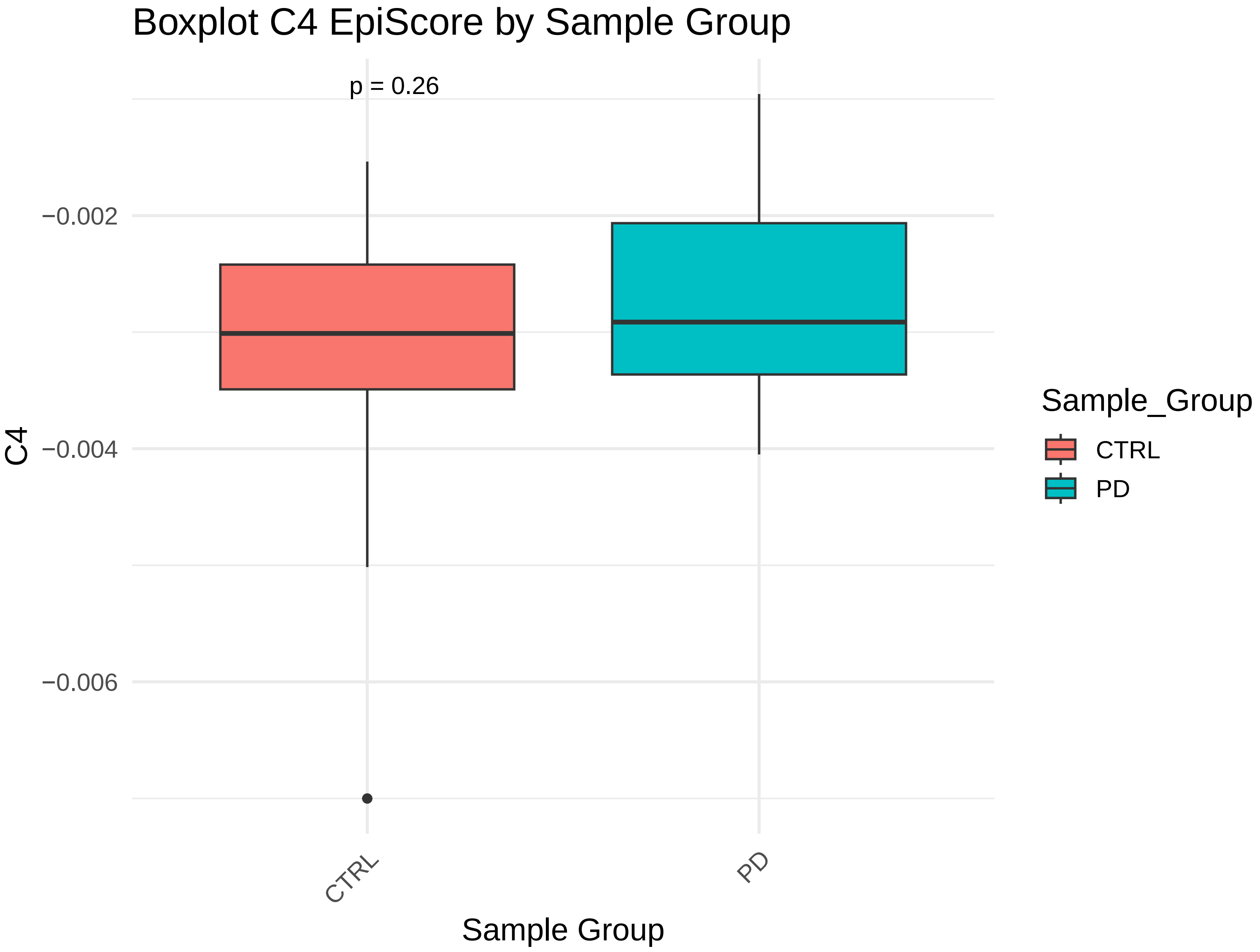
Boxplot C4 EpiScore by Sample Group
p = 0.26
−0.002
−0.004
−0.006
C4
CTRL
PD
Sample Group
Sample_Group
CTRL
PD

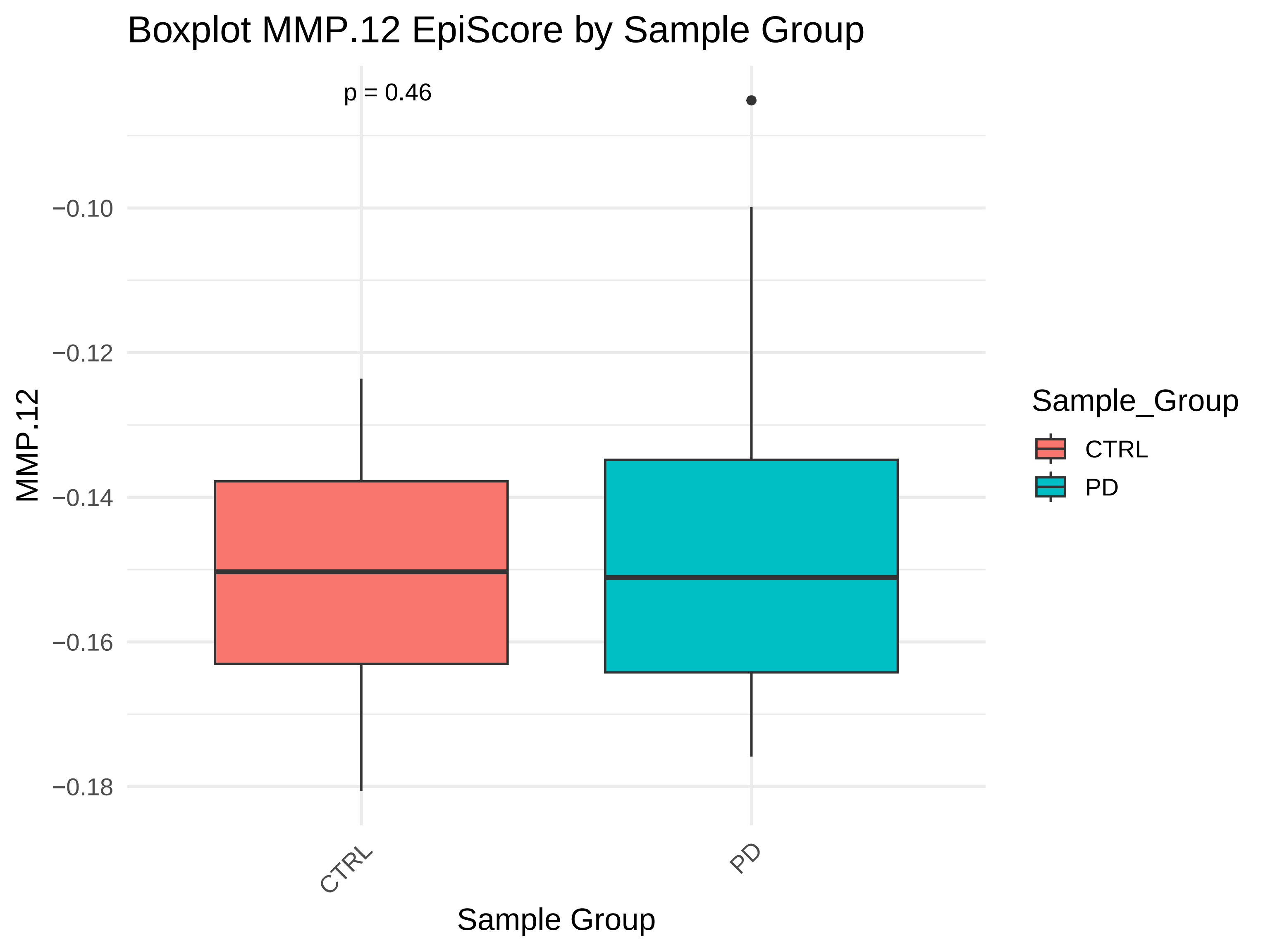

Boxplot MMP.12 EpiScore by Sample Group
p = 0.46
−0.10
−0.12
−0.14
−0.16
−0.18
MMP.12
CTRL
PD
Sample Group
Sample_Group
CTRL
PD

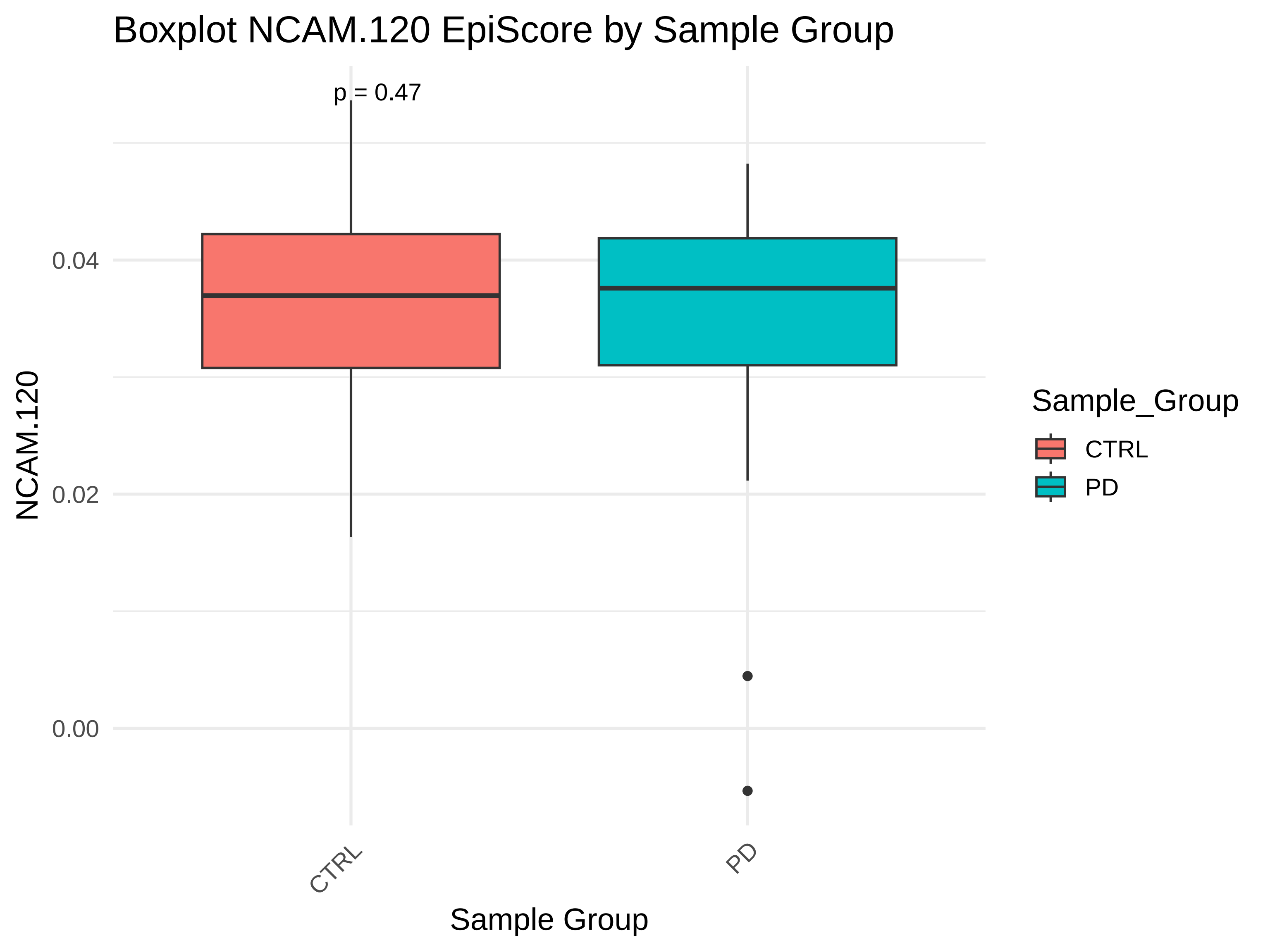
Boxplot NCAM.120 EpiScore by Sample Group
p = 0.47
0.04
0.02
0.00
NCAM.120
CTRL
PD
Sample Group
Sample_Group
CTRL
PD

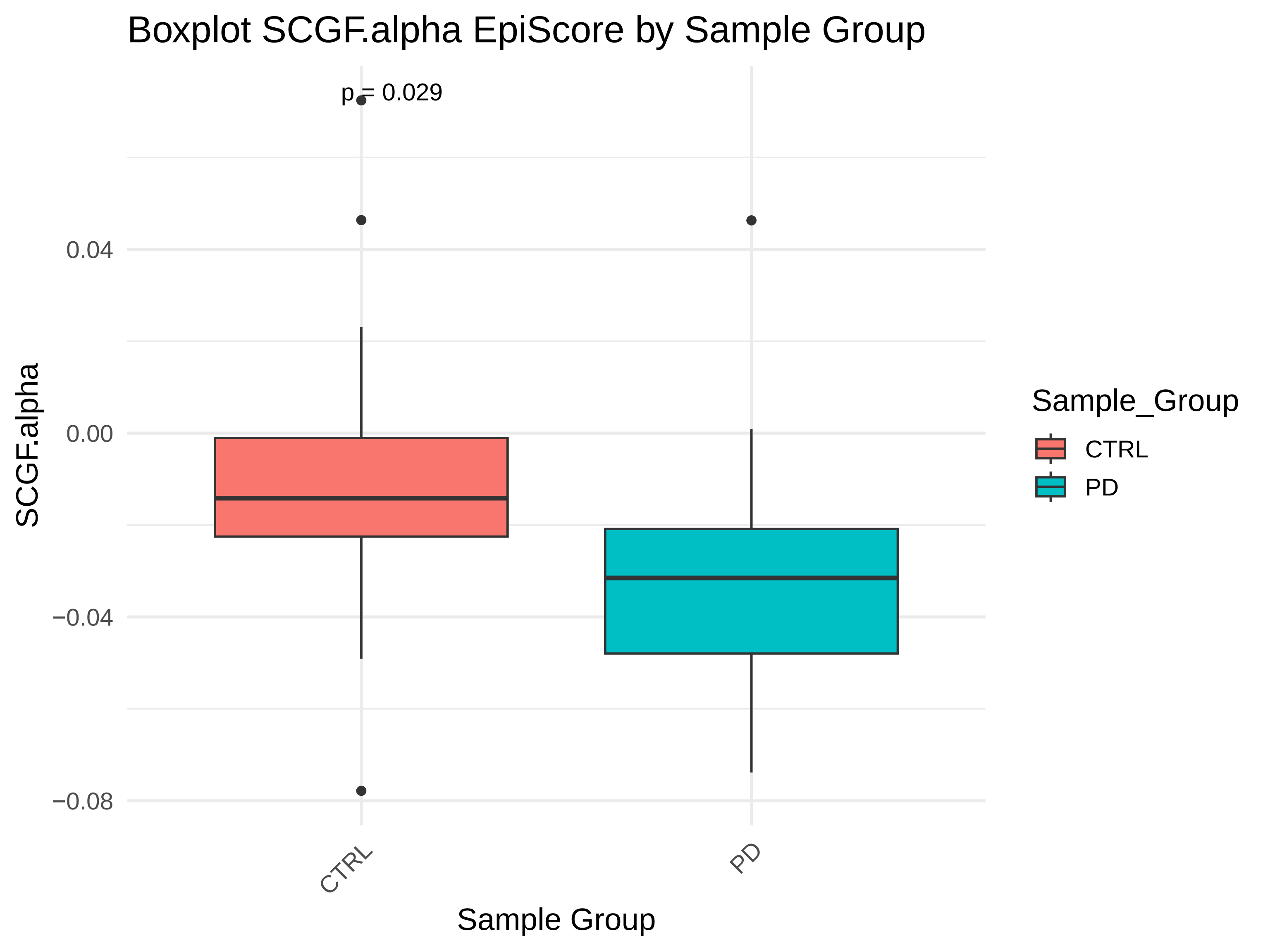
Boxplot SCGF.alpha EpiScore by Sample Group
p = 0.029
0.04
0.00
−0.04
−0.08
SCGF.alpha
CTRL
PD
Sample Group
Sample_Group
CTRL
PD

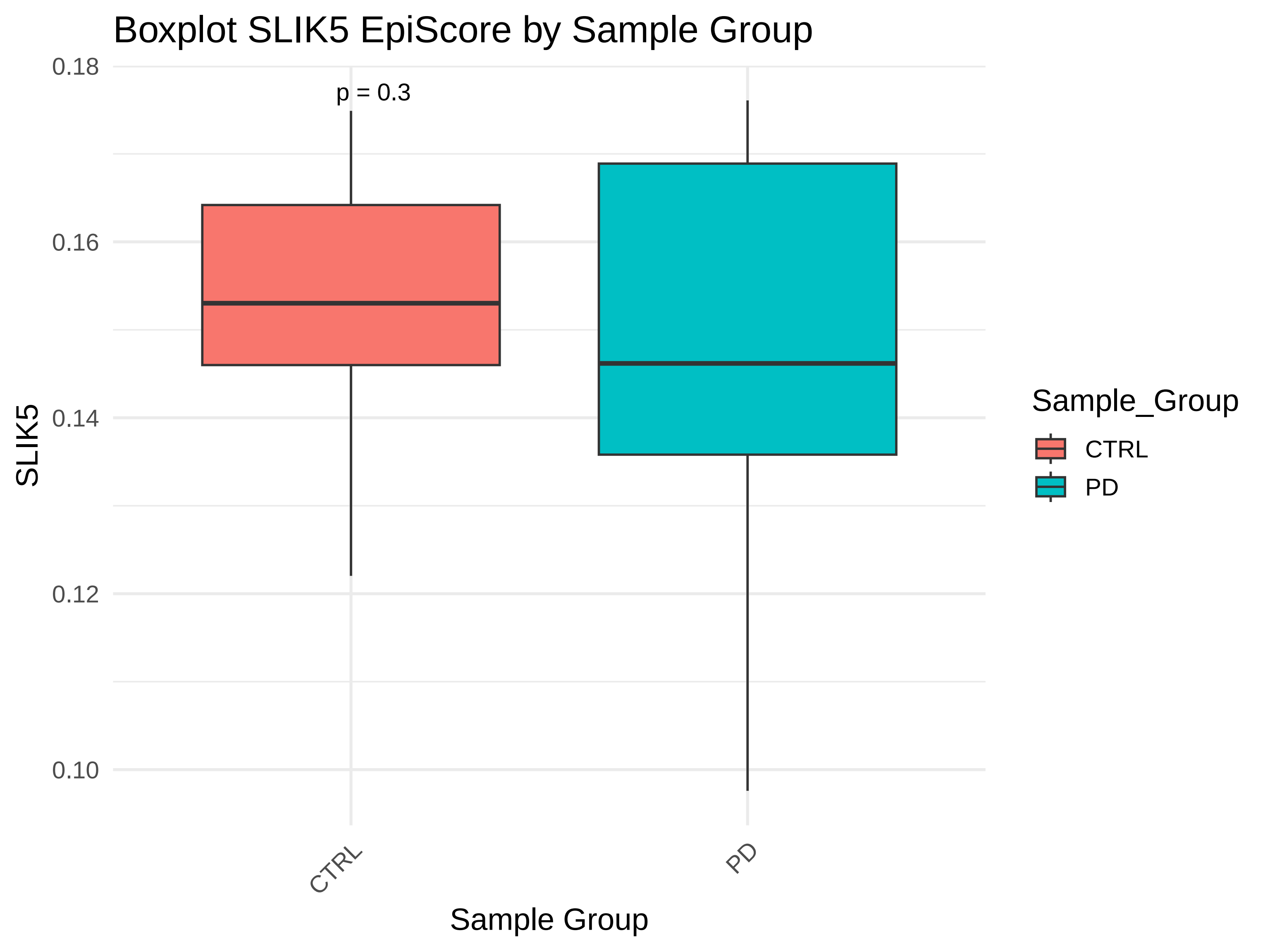

Boxplot SLIK5 EpiScore by Sample Group
p = 0.3
0.18
0.16
0.14
0.12
0.10
SLIK5
CTRL
PD
Sample Group
Sample_Group
CTRL
PD

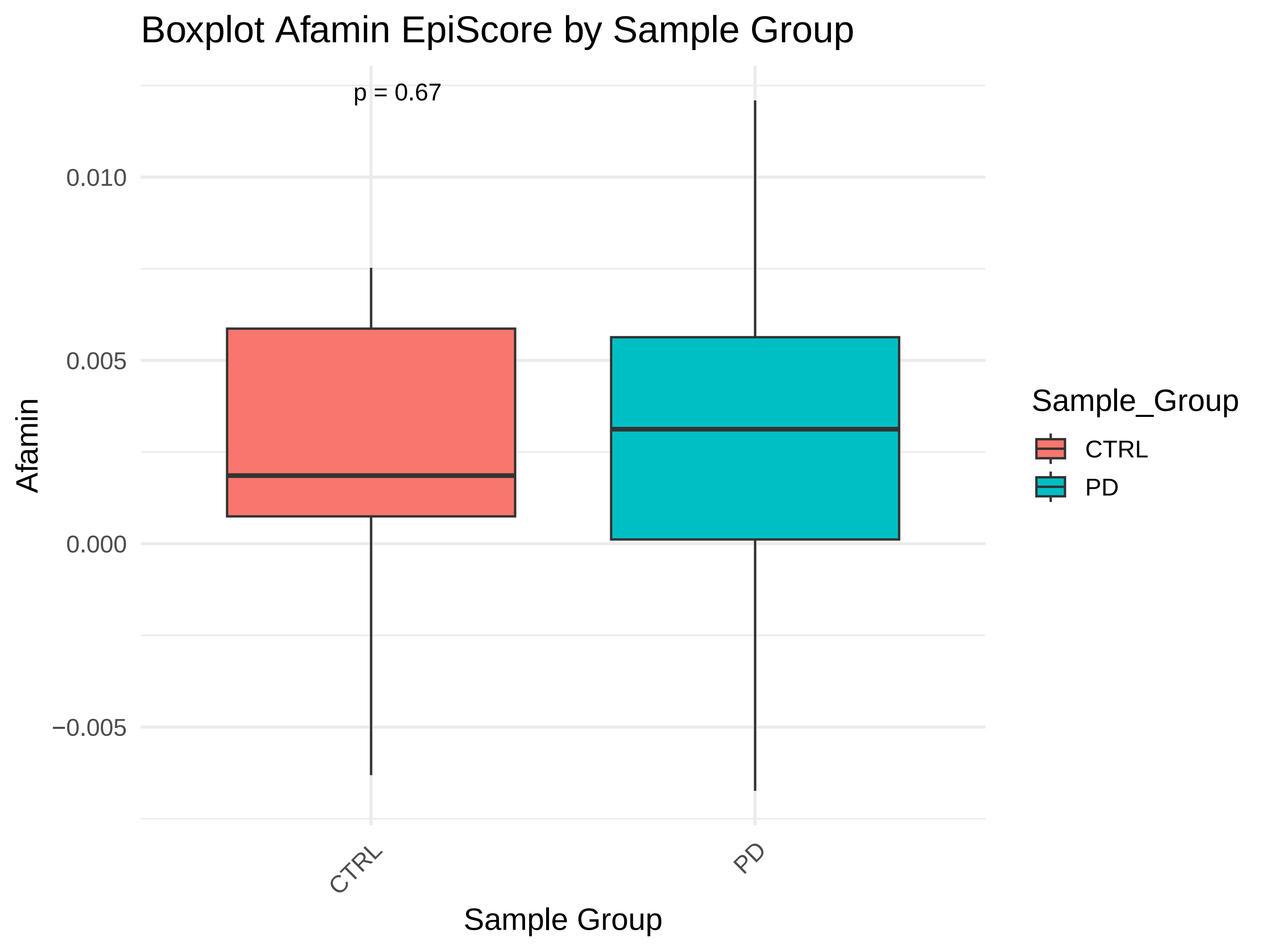

Boxplot Afamin EpiScore by Sample Group
p = 0.67
0.010
0.005
0.000
−0.005
Afamin
CTRL
PD
Sample Group
Sample_Group
CTRL
PD

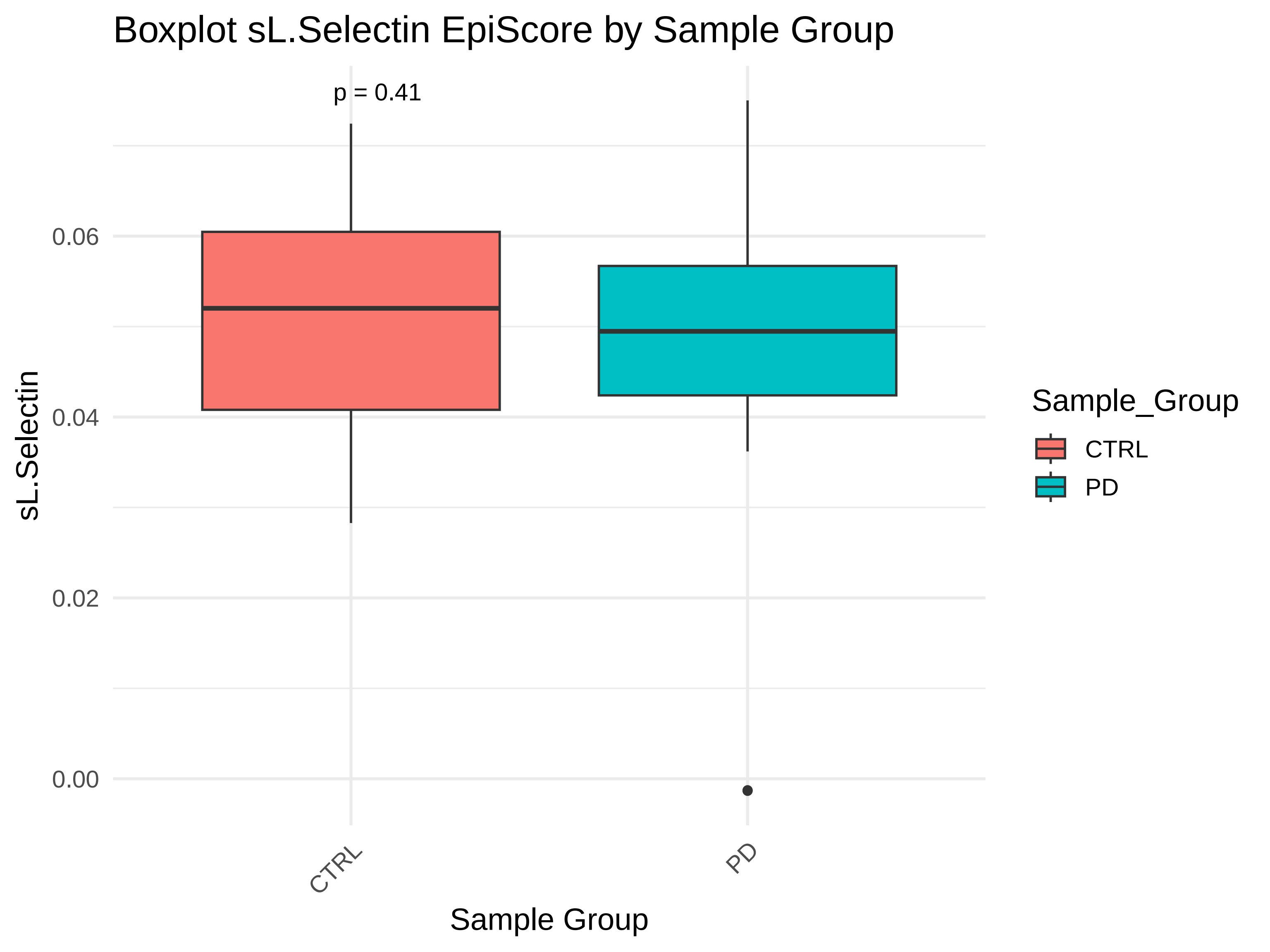
Boxplot sL.Selectin EpiScore by Sample Group
p = 0.41
0.06
0.04
0.02
0.00
sL.Selectin
CTRL
PD
Sample Group
Sample_Group
CTRL
PD

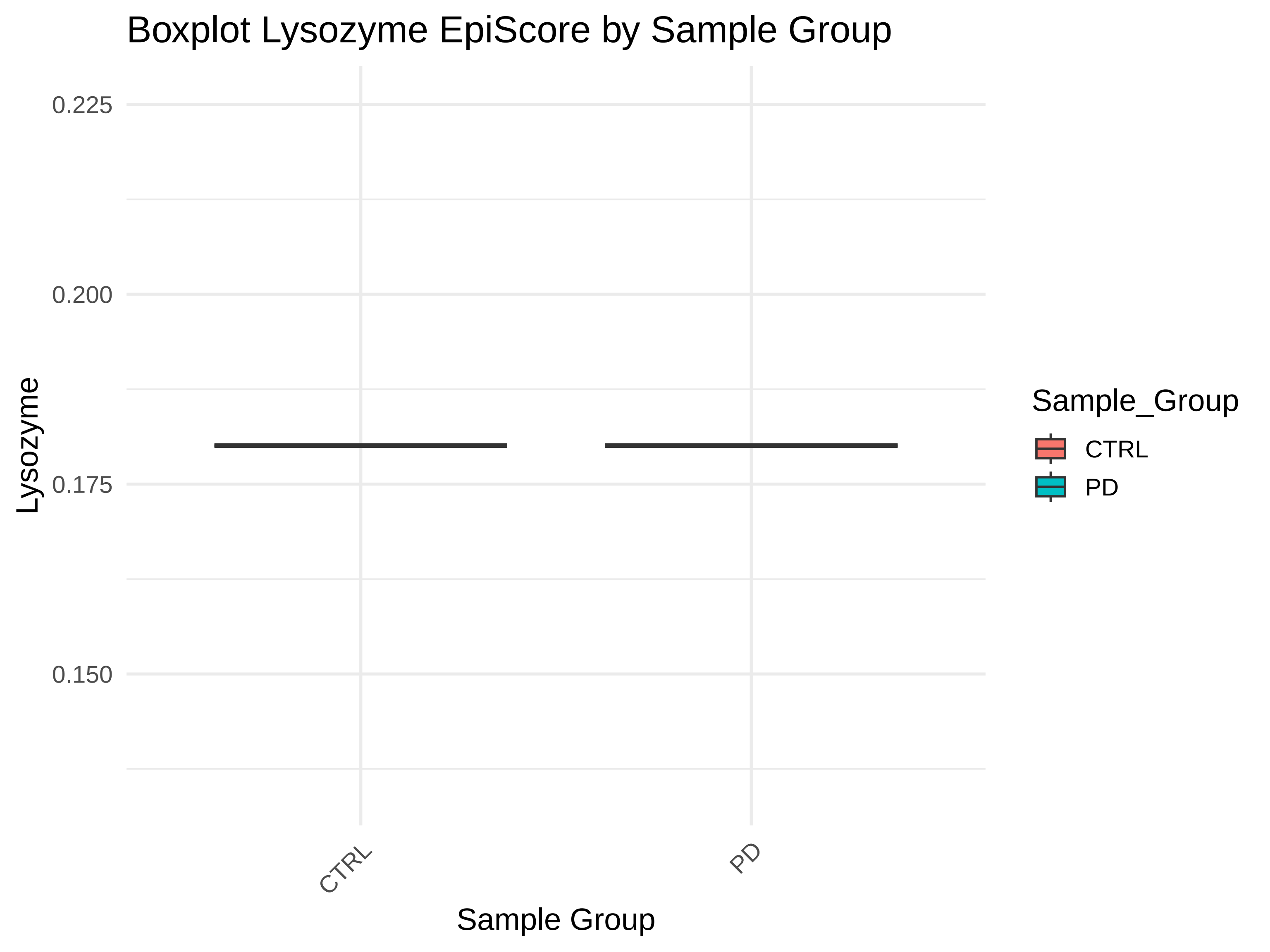
Boxplot Lysozyme EpiScore by Sample Group
0.225
0.200
0.175
0.150
Lysozyme
CTRL
PD
Sample Group
Sample_Group
CTRL
PD

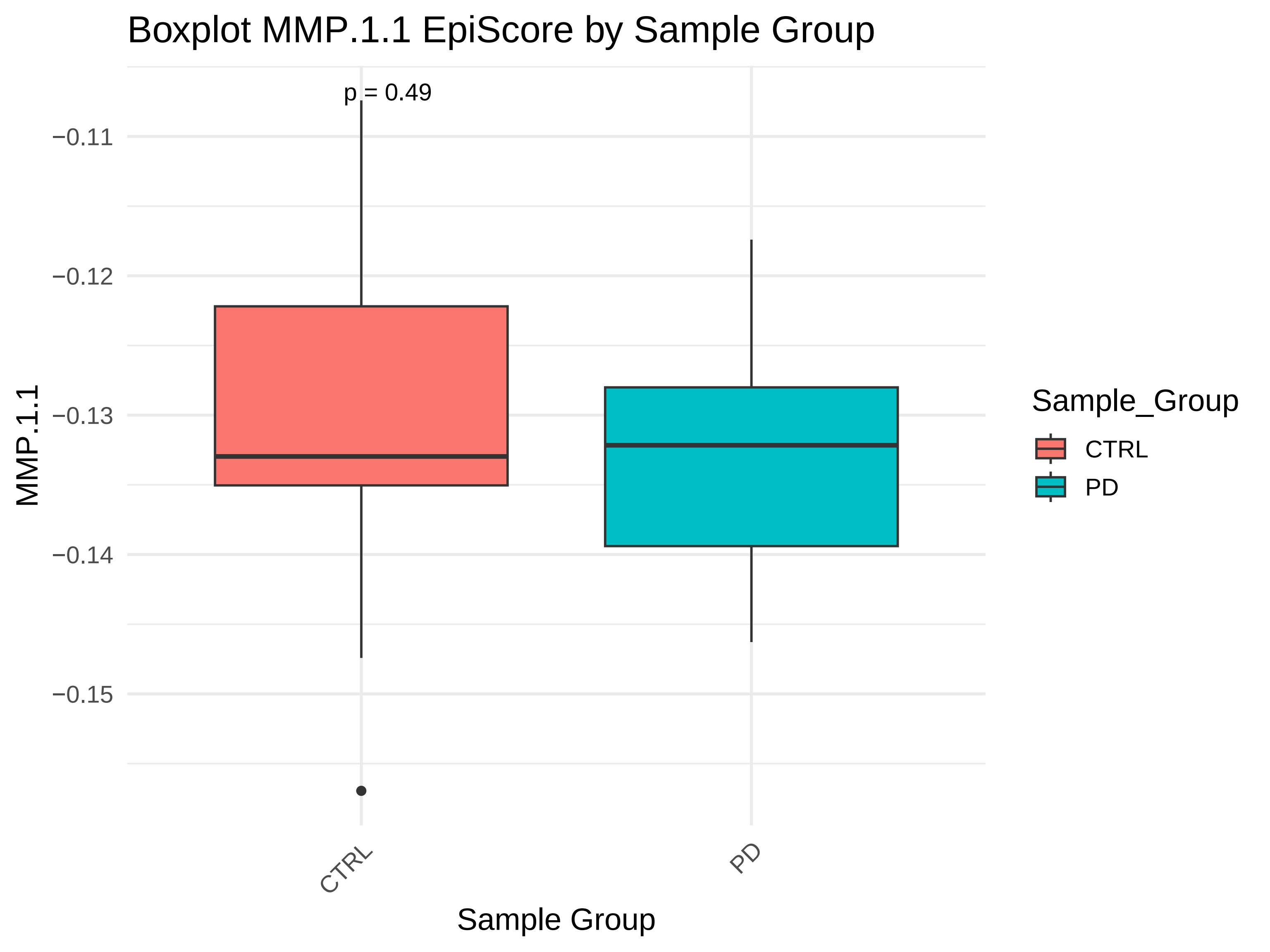
Boxplot MMP.1.1 EpiScore by Sample Group
p = 0.49
−0.11
−0.12
−0.13
−0.14
−0.15
MMP.1.1
CTRL
PD
Sample Group
Sample_Group
CTRL
PD

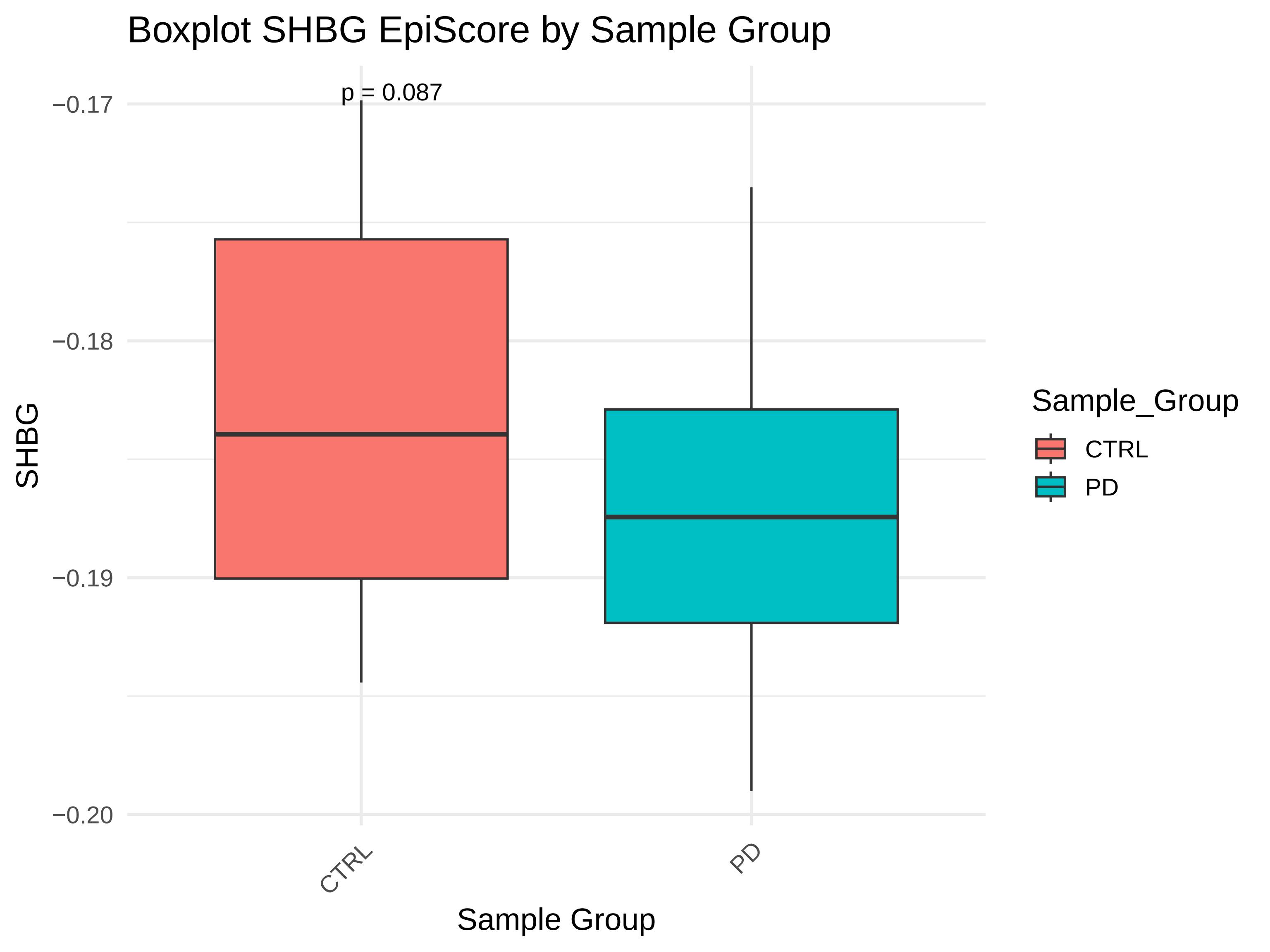
Boxplot SHBG EpiScore by Sample Group
p = 0.087
−0.17
−0.18
−0.19
−0.20
SHBG
CTRL
PD
Sample Group
Sample_Group
CTRL
PD

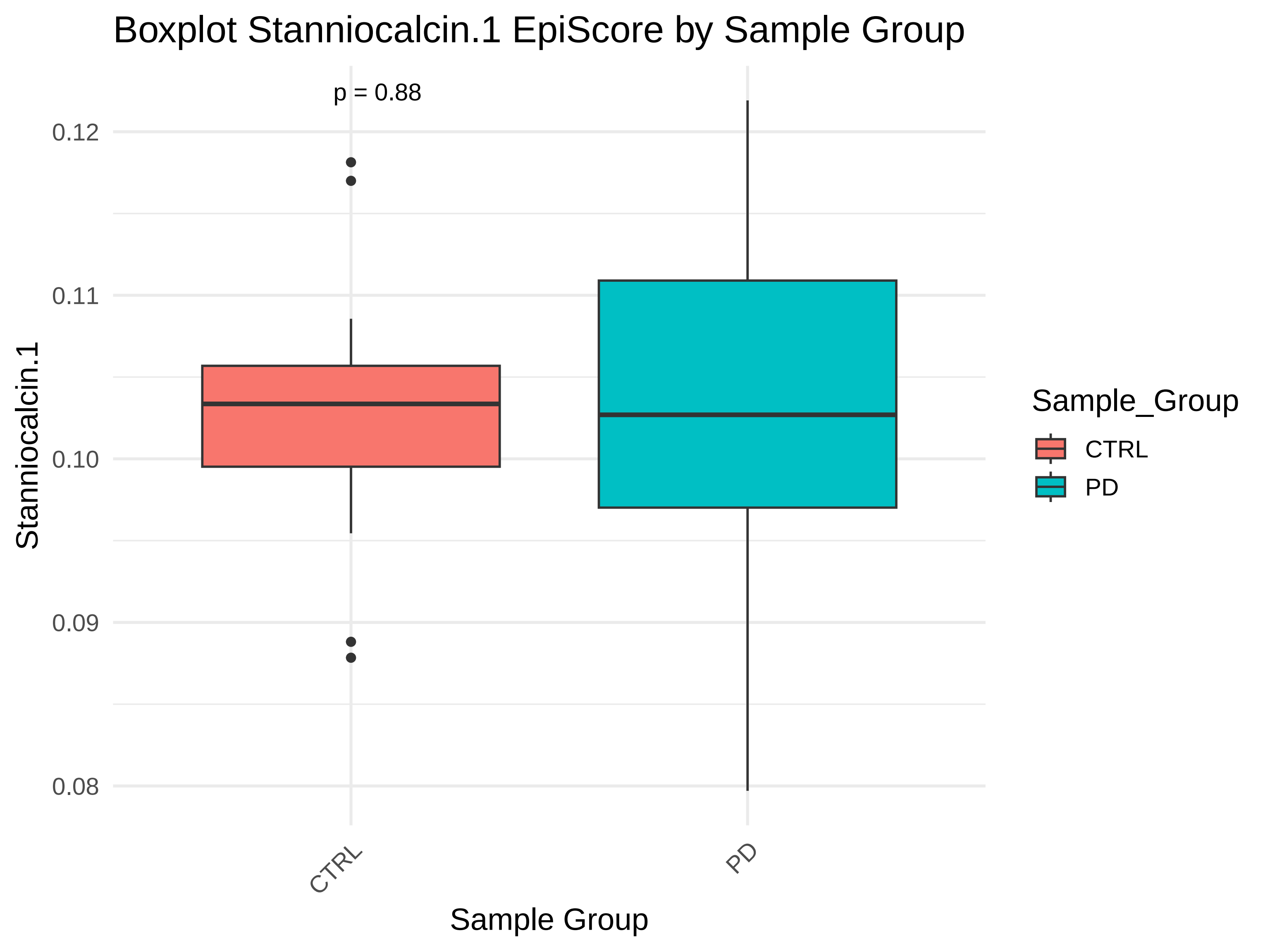
Boxplot Stanniocalcin.1 EpiScore by Sample Group
p = 0.88
0.12
0.11
0.10
0.09
0.08
Stanniocalcin.1
CTRL
PD
Sample Group
Sample_Group
CTRL
PD

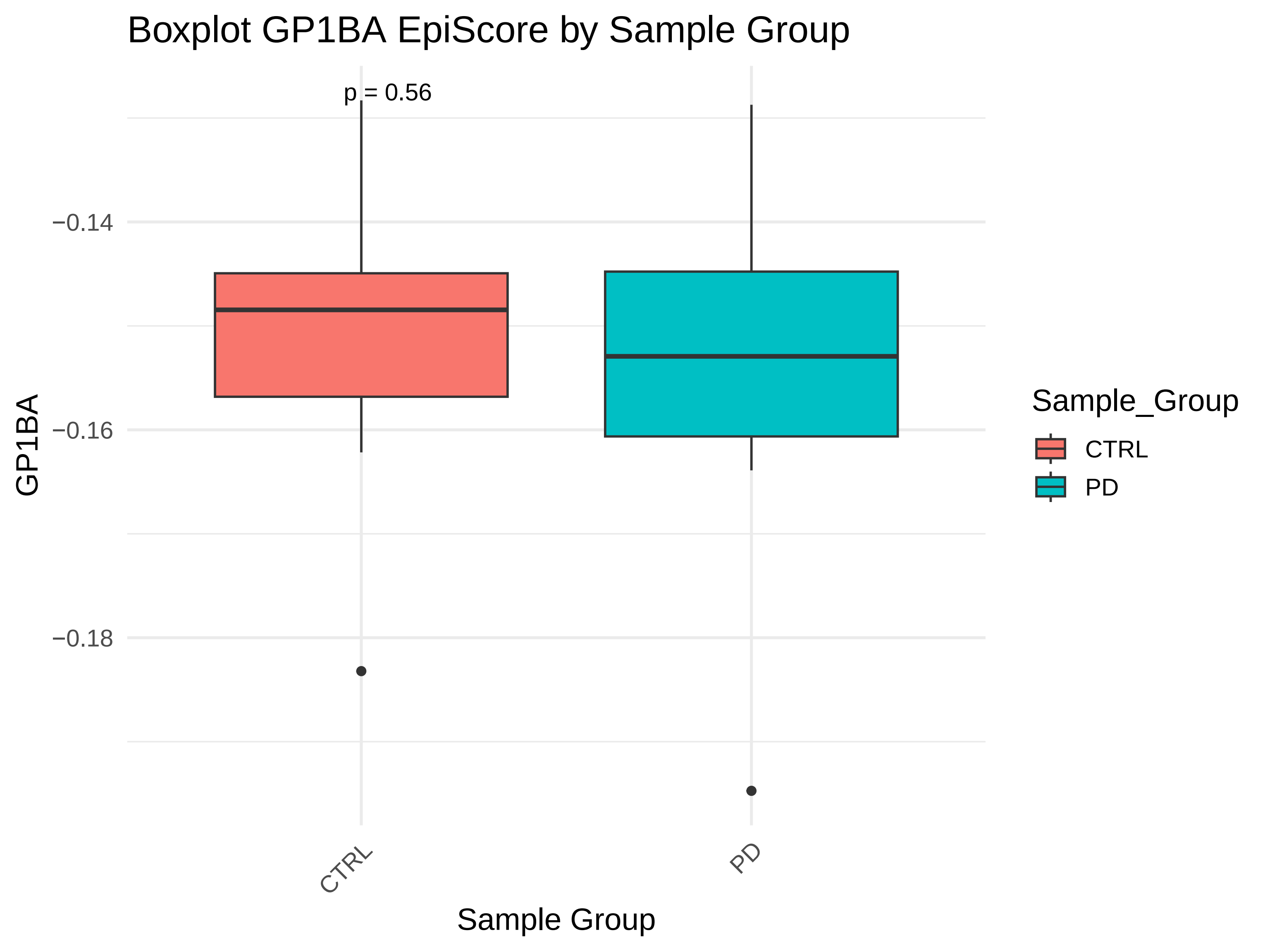

Boxplot GP1BA EpiScore by Sample Group
p = 0.56
−0.14
−0.16
−0.18
GP1BA
CTRL
PD
Sample Group
Sample_Group
CTRL
PD

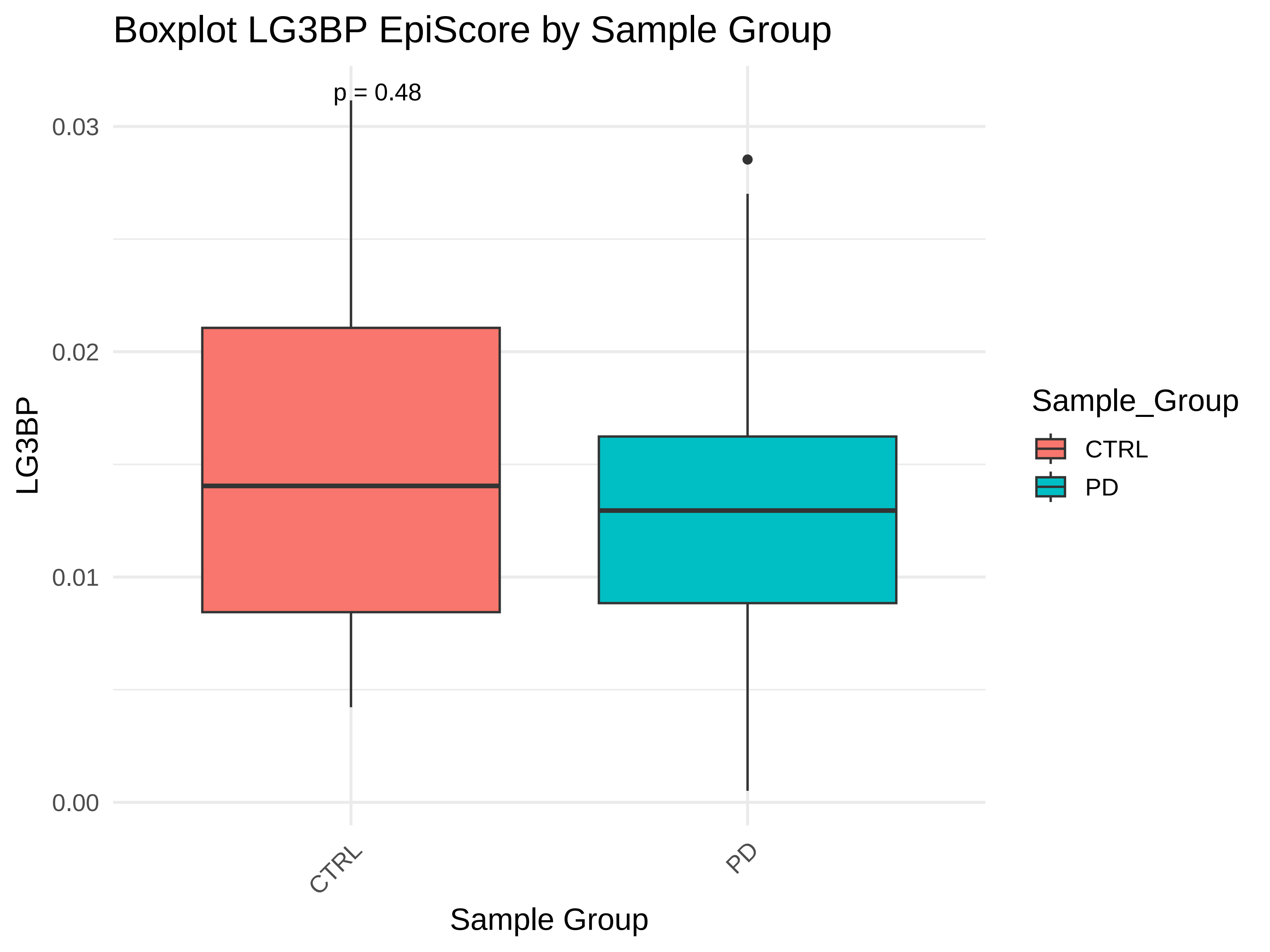

Boxplot LG3BP EpiScore by Sample Group
p = 0.48
0.03
0.02
0.01
0.00
LG3BP
CTRL
PD
Sample Group
Sample_Group
CTRL
PD

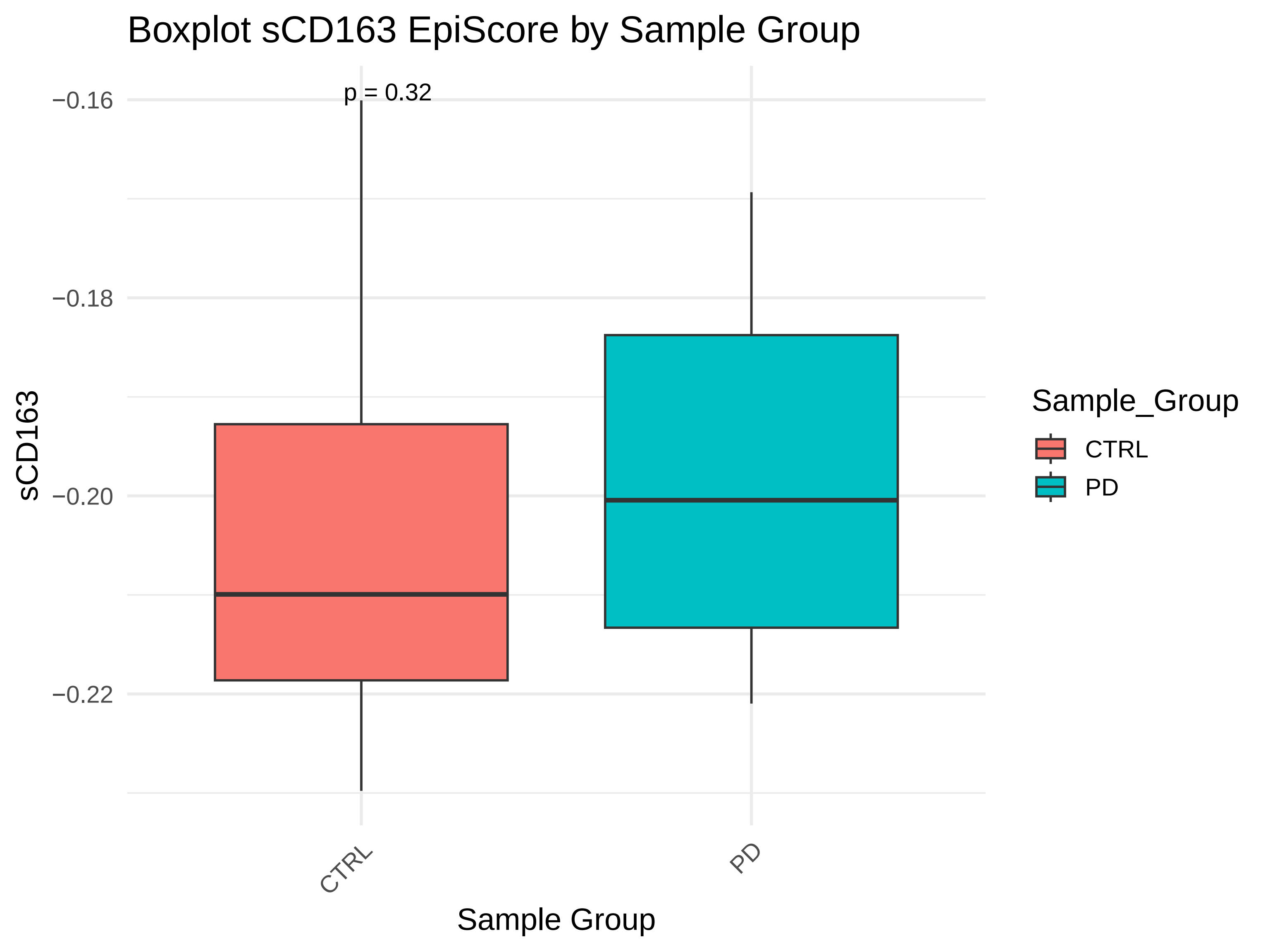

Boxplot sCD163 EpiScore by Sample Group
p = 0.32
−0.16
−0.18
−0.20
−0.22
sCD163
CTRL
PD
Sample Group
Sample_Group
CTRL
PD

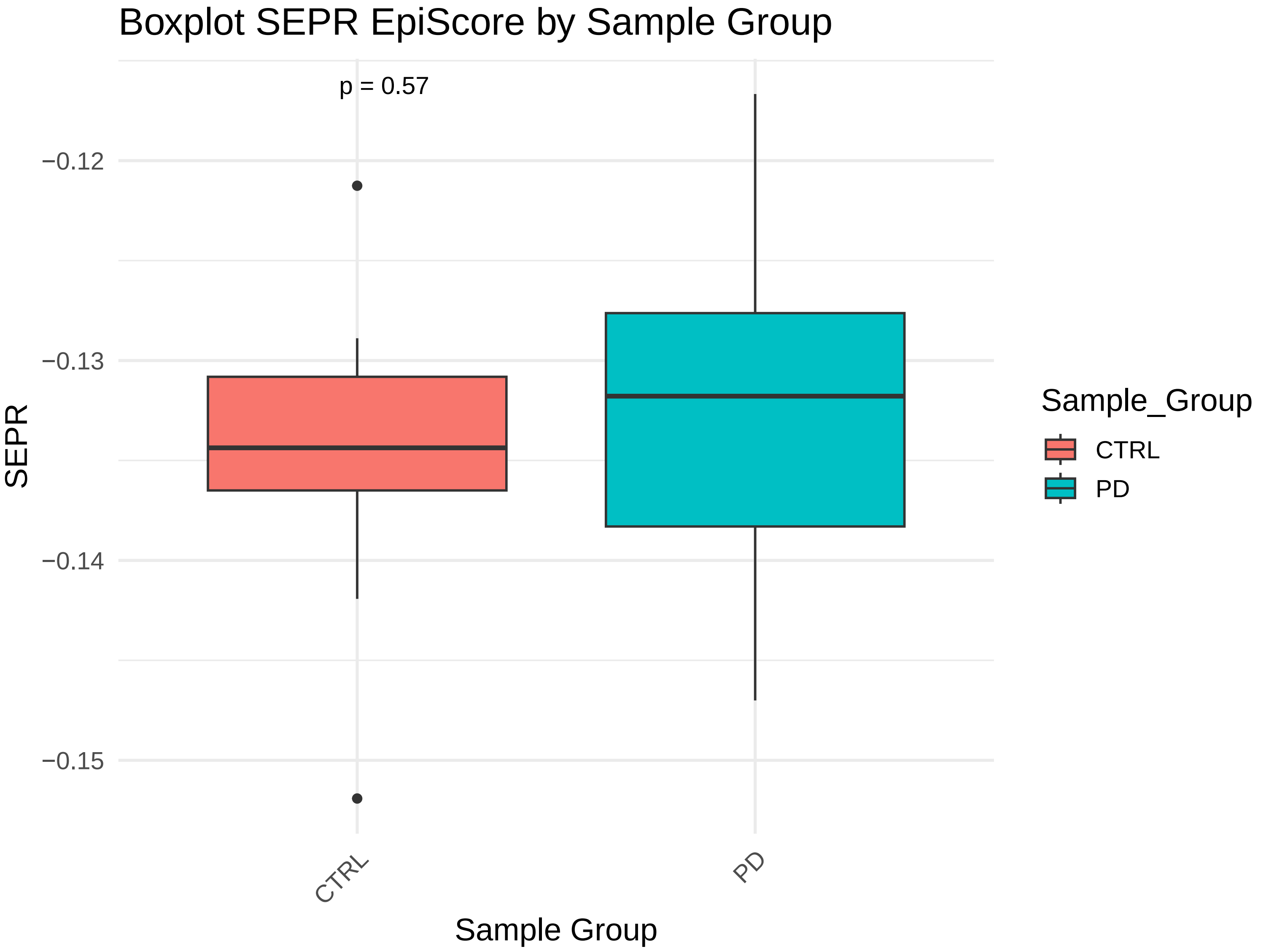

Boxplot SEPR EpiScore by Sample Group
p = 0.57
−0.12
−0.13
−0.14
−0.15
SEPR
CTRL
PD
Sample Group
Sample_Group
CTRL
PD

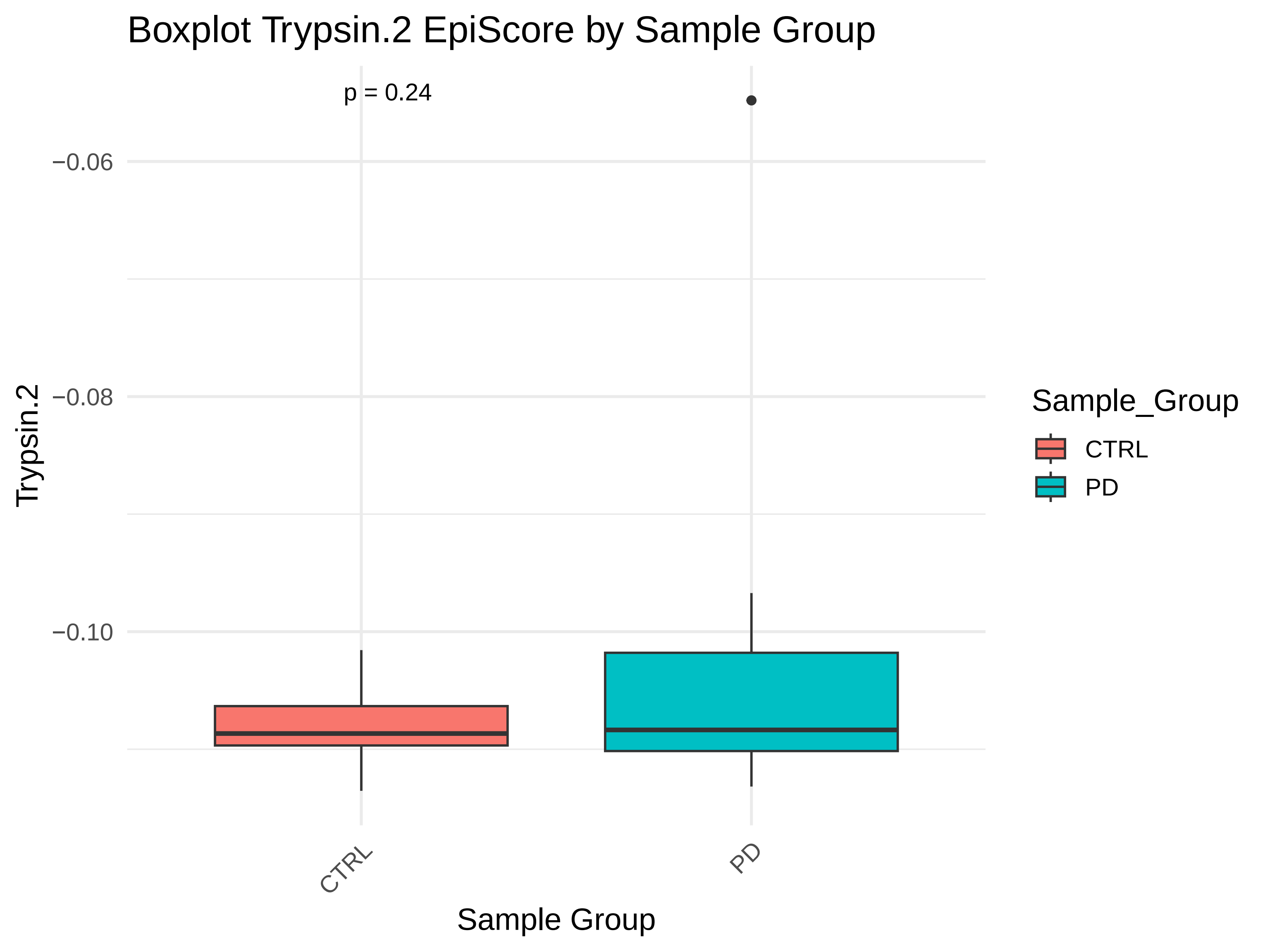

Boxplot Trypsin.2 EpiScore by Sample Group
p = 0.24
−0.06
−0.08
−0.10
Trypsin.2
CTRL
PD
Sample Group
Sample_Group
CTRL
PD

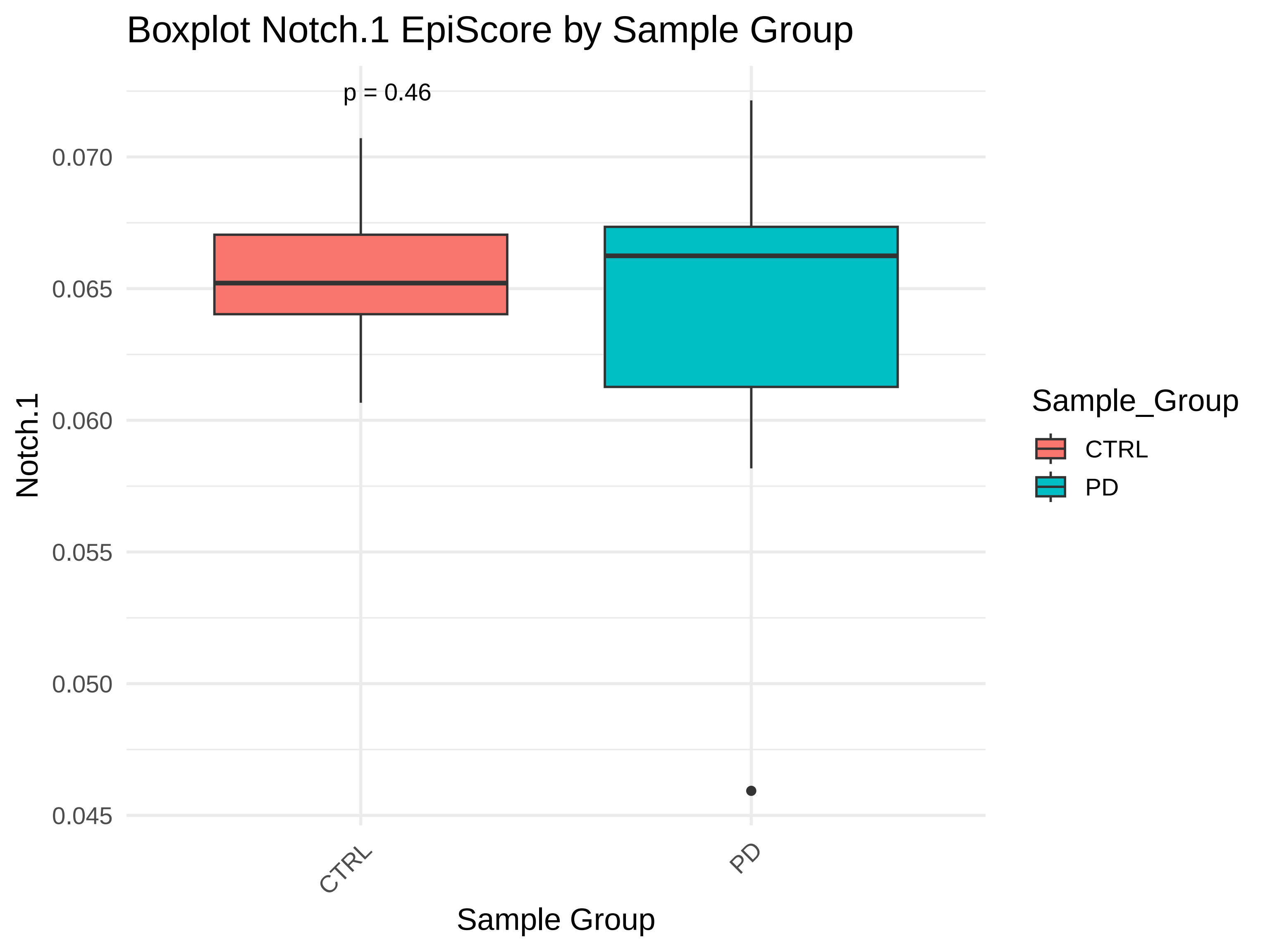
Boxplot Notch.1 EpiScore by Sample Group
p = 0.46
0.070
0.065
0.060
0.055
0.050
0.045
Notch.1
CTRL
PD
Sample Group
Sample_Group
CTRL
PD

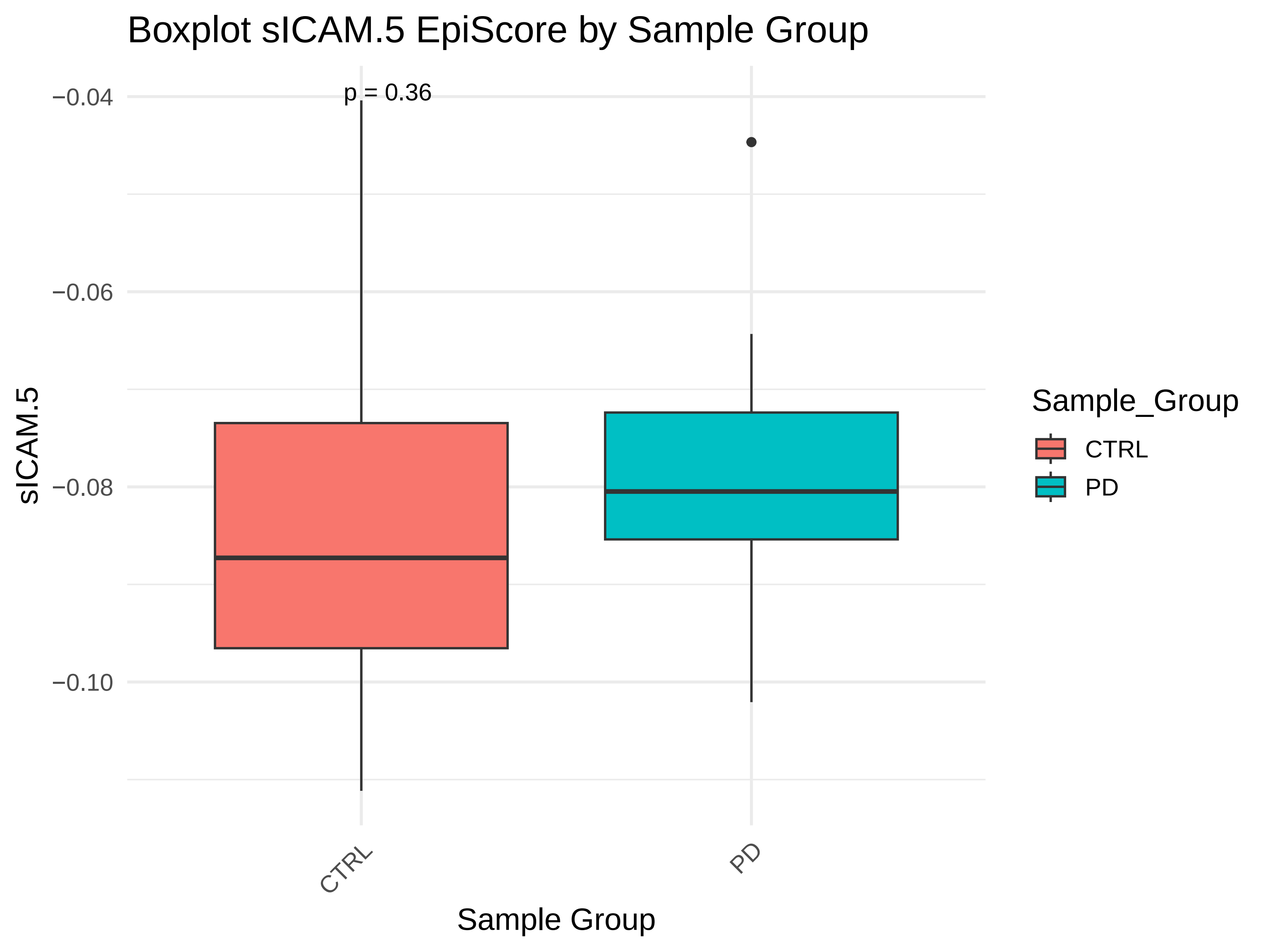

Boxplot sICAM.5 EpiScore by Sample Group
p = 0.36
−0.04
−0.06
−0.08
−0.10
sICAM.5
CTRL
PD
Sample Group
Sample_Group
CTRL
PD

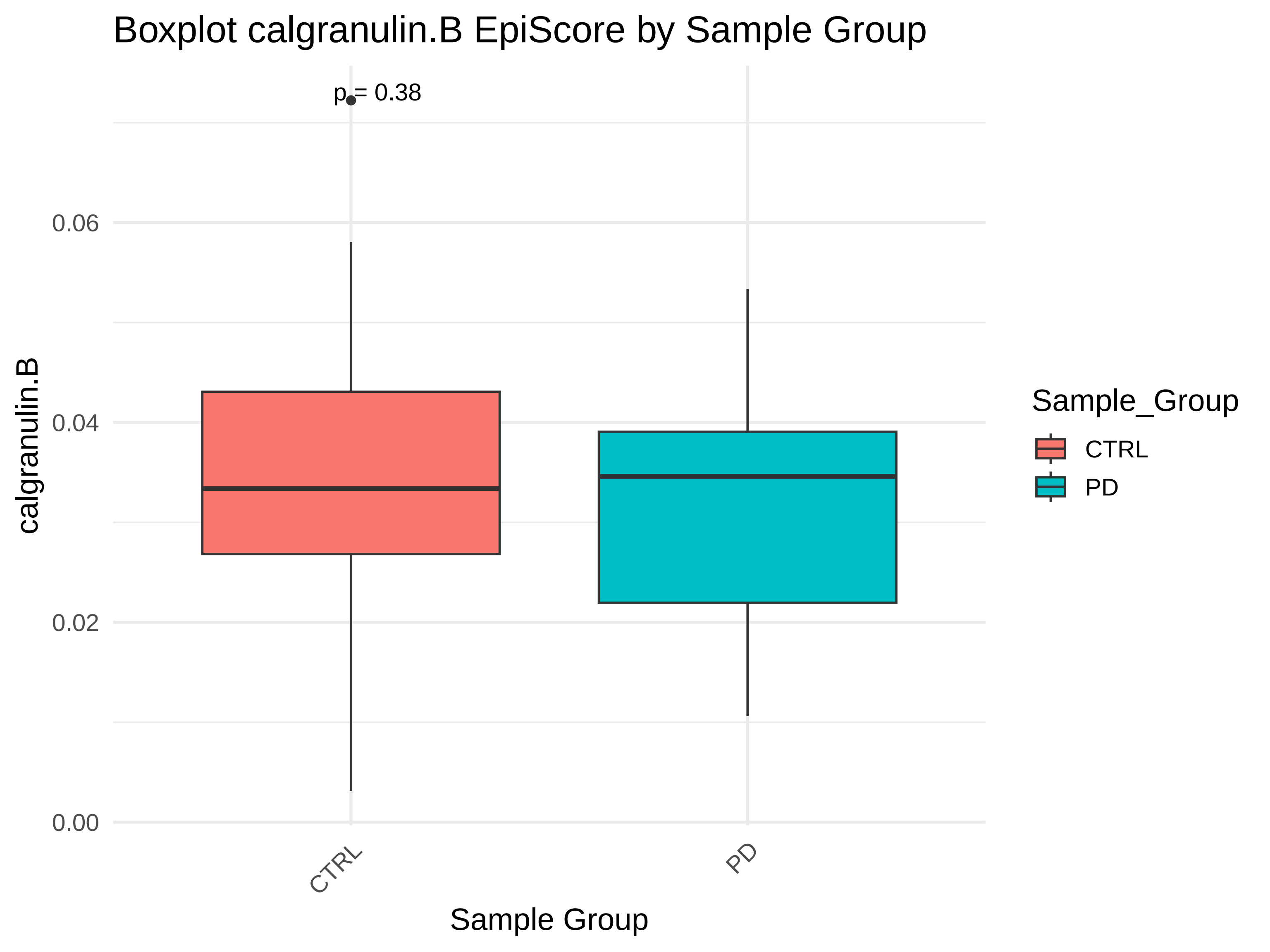
Boxplot calgranulin.B EpiScore by Sample Group
p = 0.38
0.06
0.04
0.02
0.00
calgranulin.B
CTRL
PD
Sample Group
Sample_Group
CTRL
PD

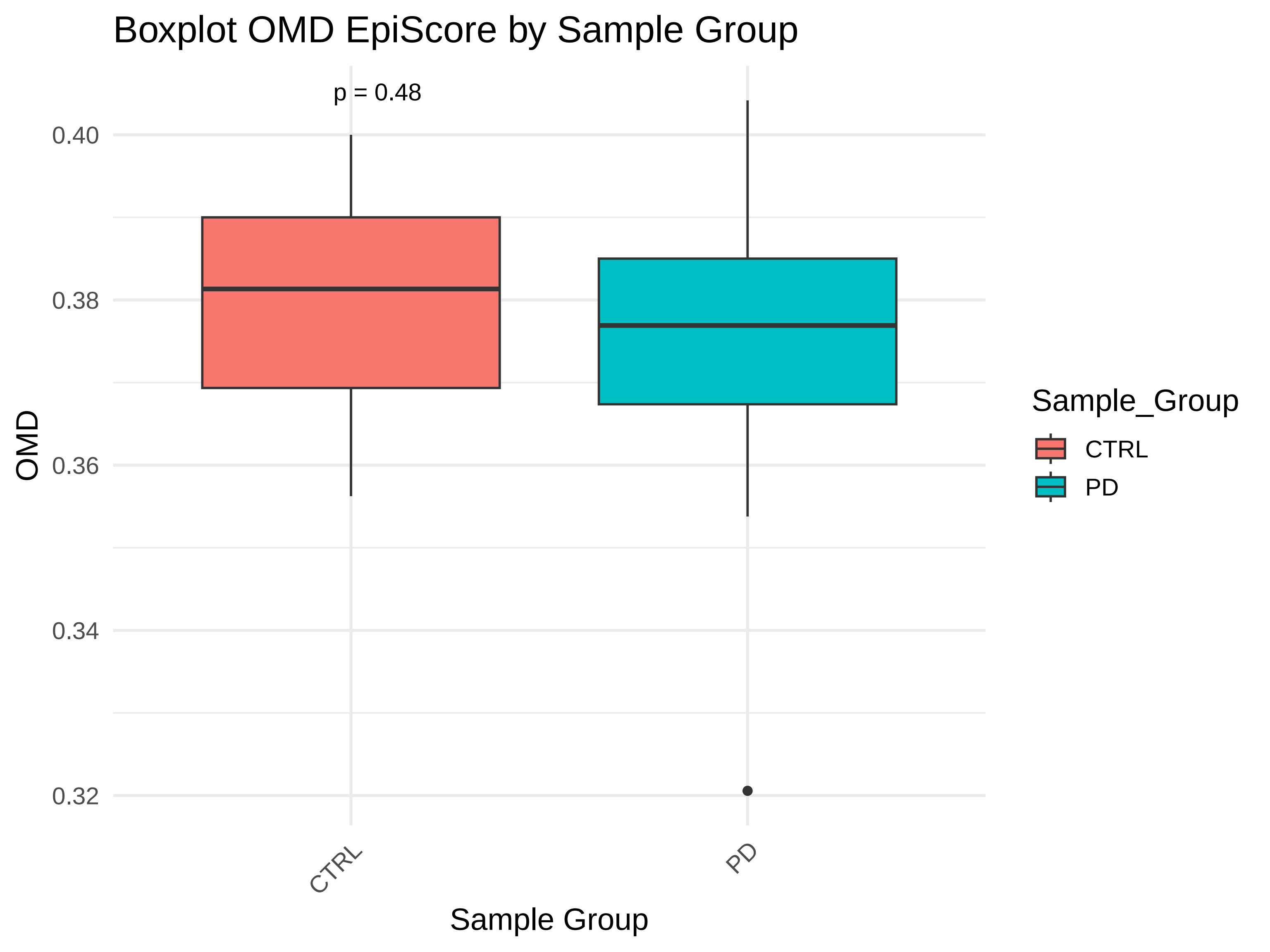

Boxplot OMD EpiScore by Sample Group
p = 0.48
0.40
0.38
0.36
0.34
0.32
OMD
CTRL
PD
Sample Group
Sample_Group
CTRL
PD

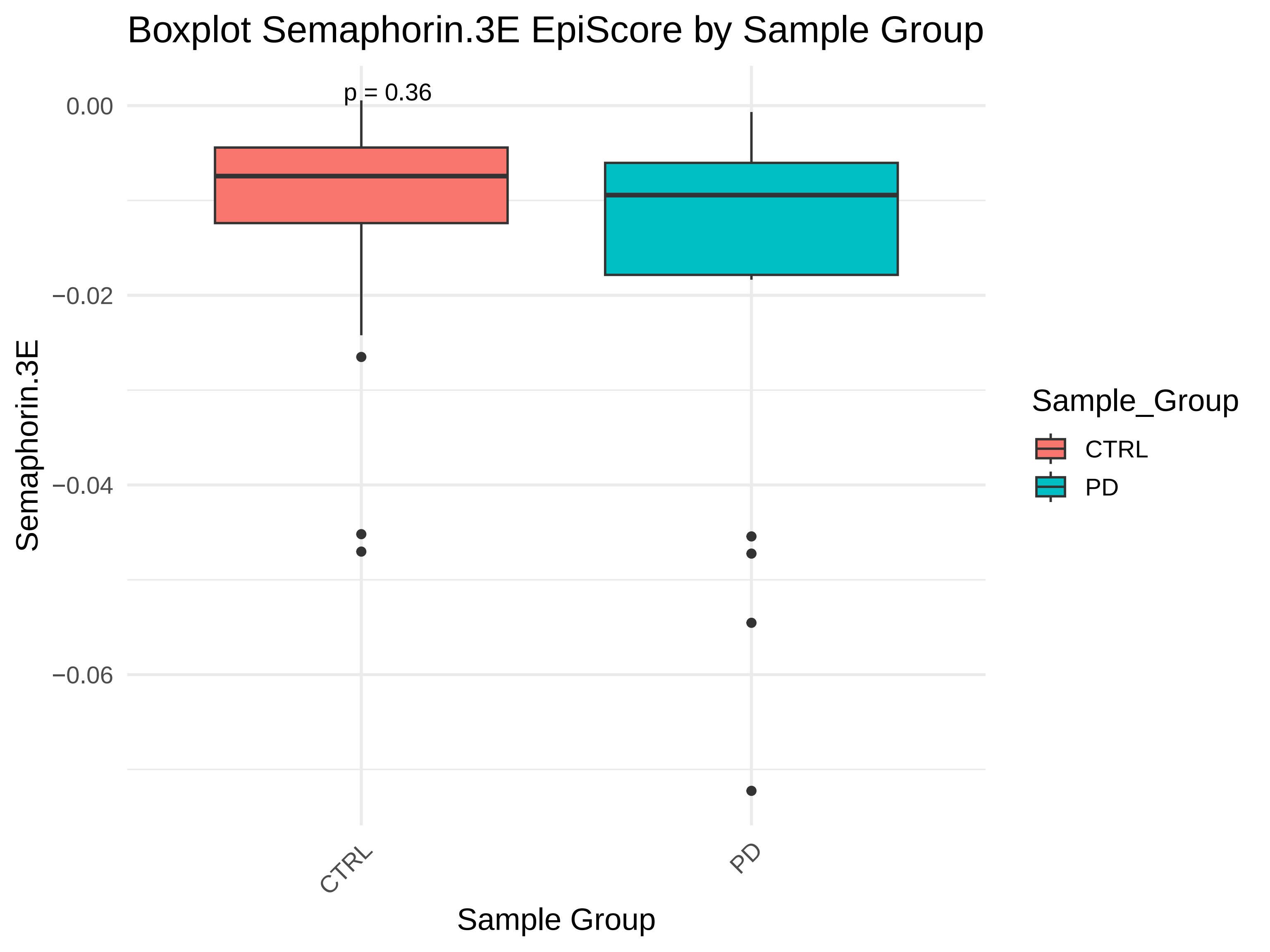
Boxplot Semaphorin.3E EpiScore by Sample Group
p = 0.36
0.00
−0.02
−0.04
−0.06
Semaphorin.3E
CTRL
PD
Sample Group
Sample_Group
CTRL
PD

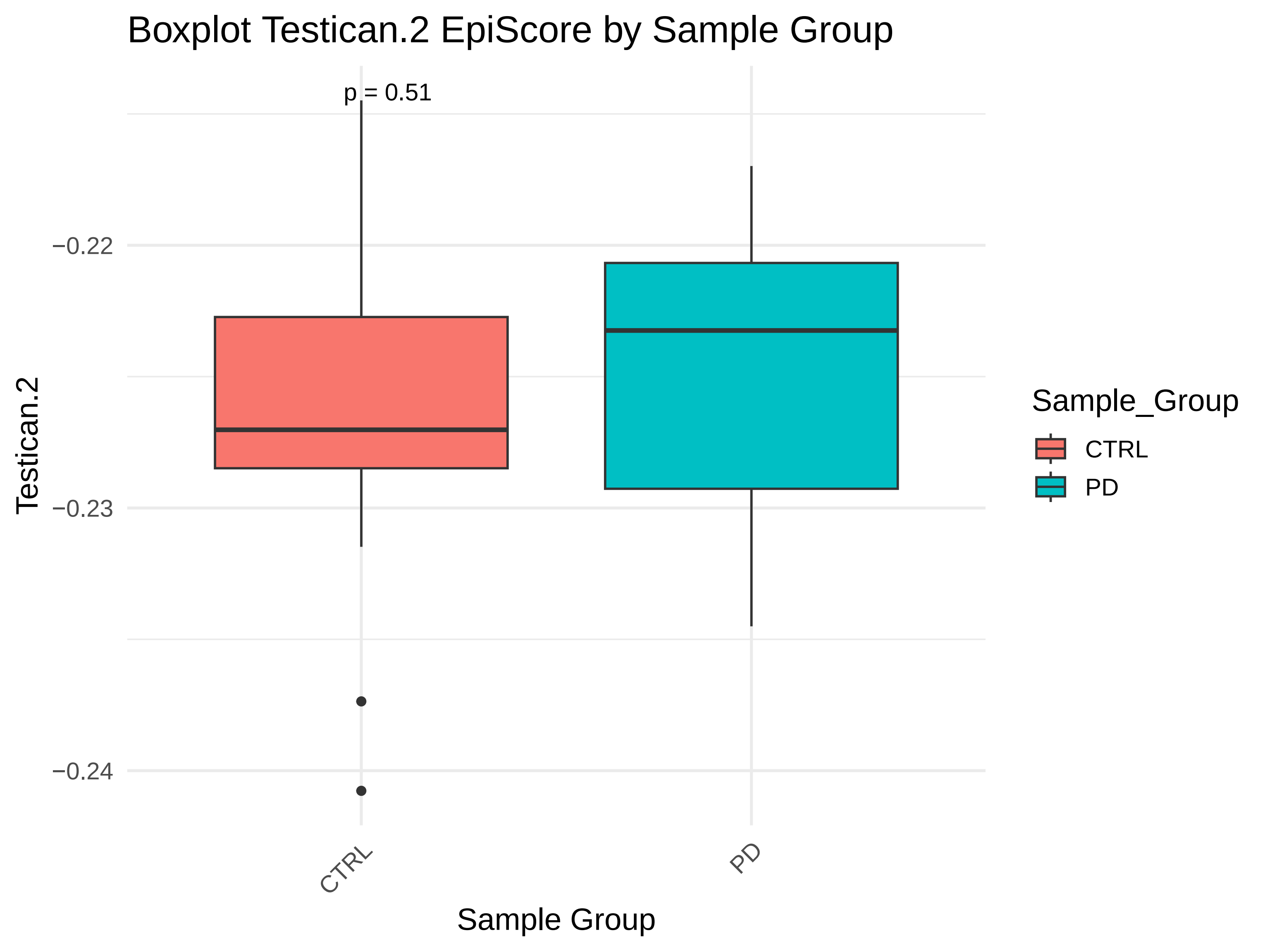

Boxplot Testican.2 EpiScore by Sample Group
p = 0.51
−0.22
−0.23
−0.24
Testican.2
CTRL
PD
Sample Group
Sample_Group
CTRL
PD

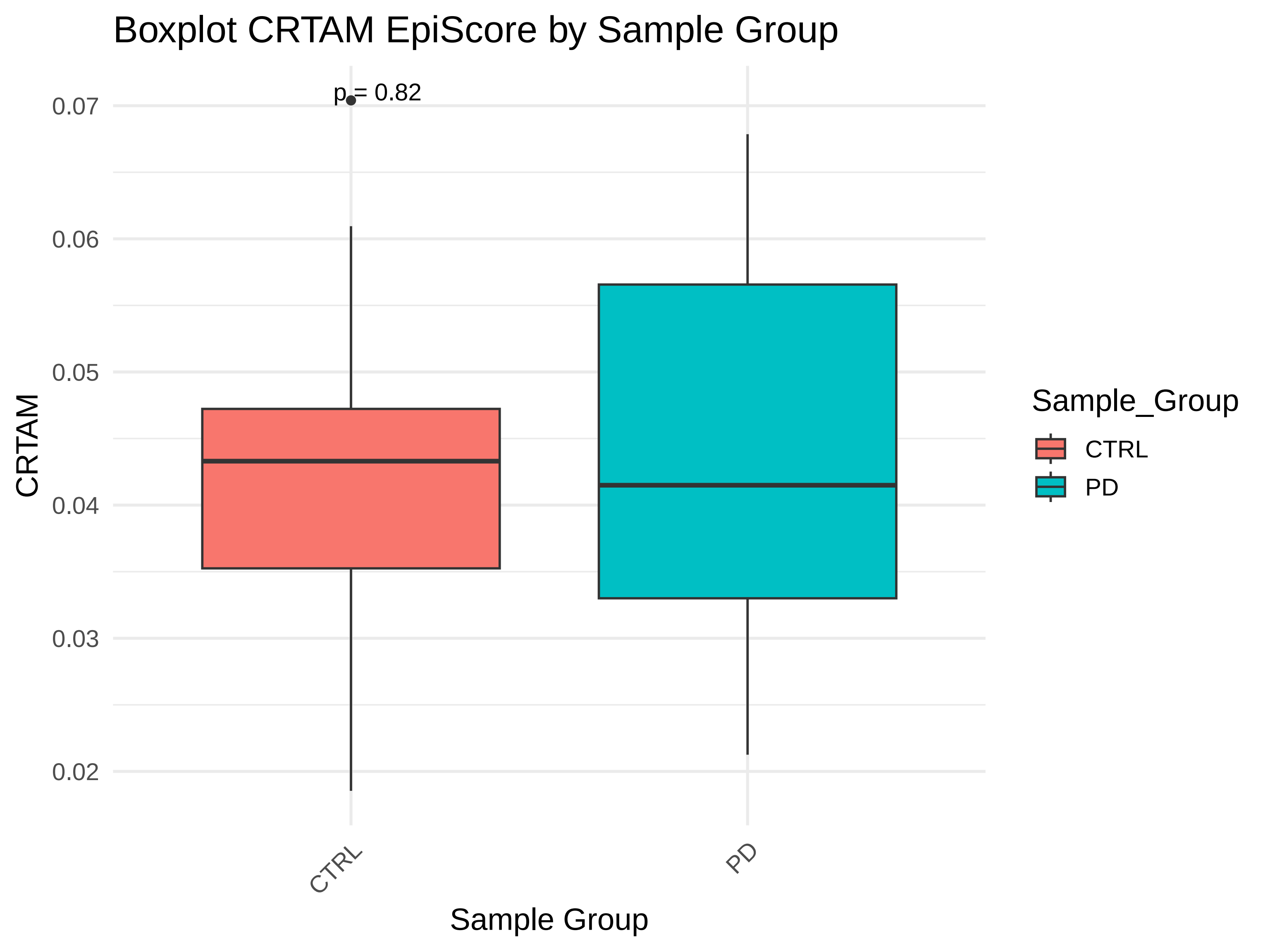

Boxplot CRTAM EpiScore by Sample Group
p = 0.82
0.07
0.06
0.05
0.04
0.03
0.02
CRTAM
CTRL
PD
Sample Group
Sample_Group
CTRL
PD

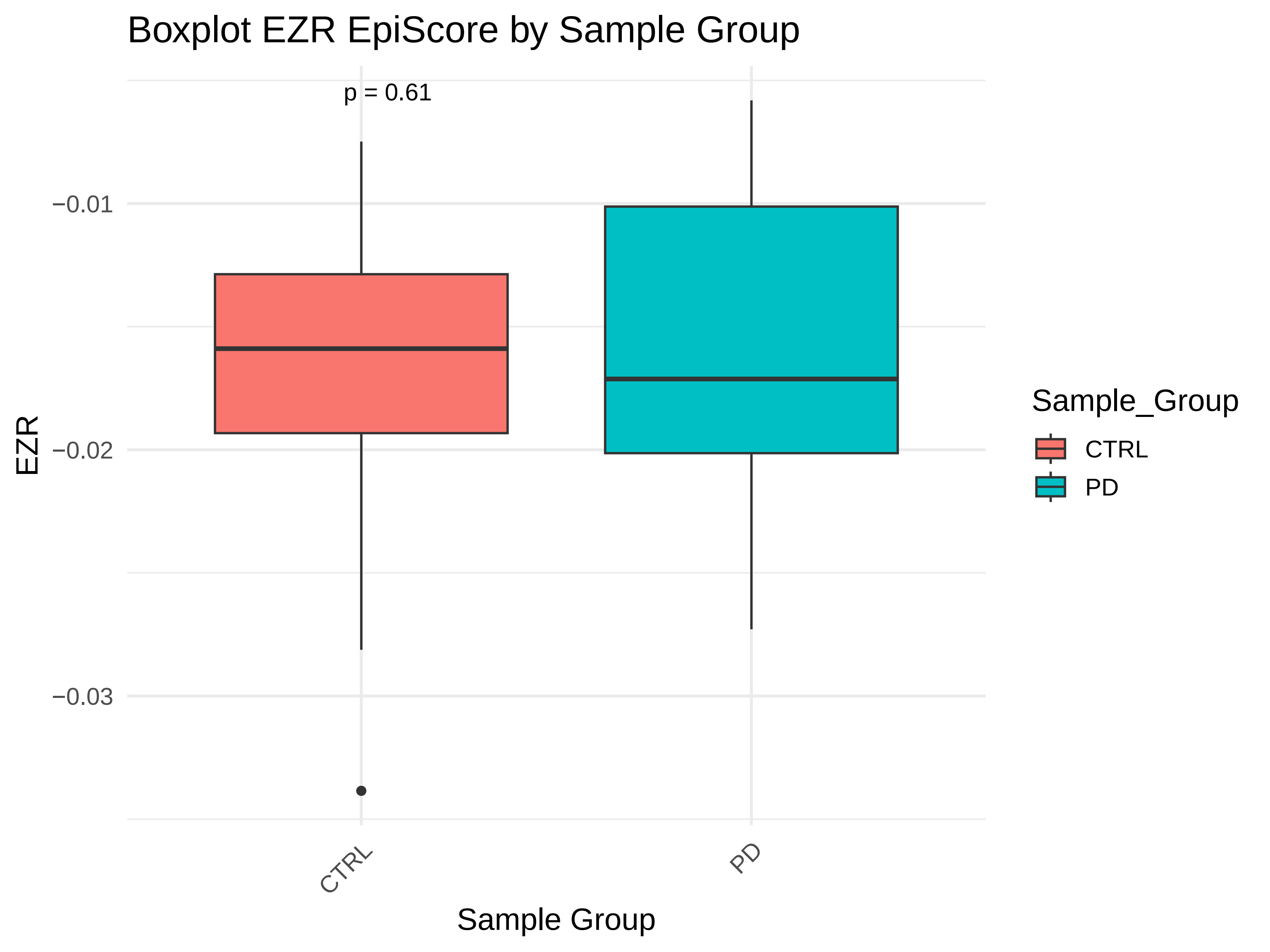
Boxplot EZR EpiScore by Sample Group
p = 0.61
EZR
−0.01
−0.02
−0.03
CTRL
PD
Sample Group
Sample_Group
CTRL
PD

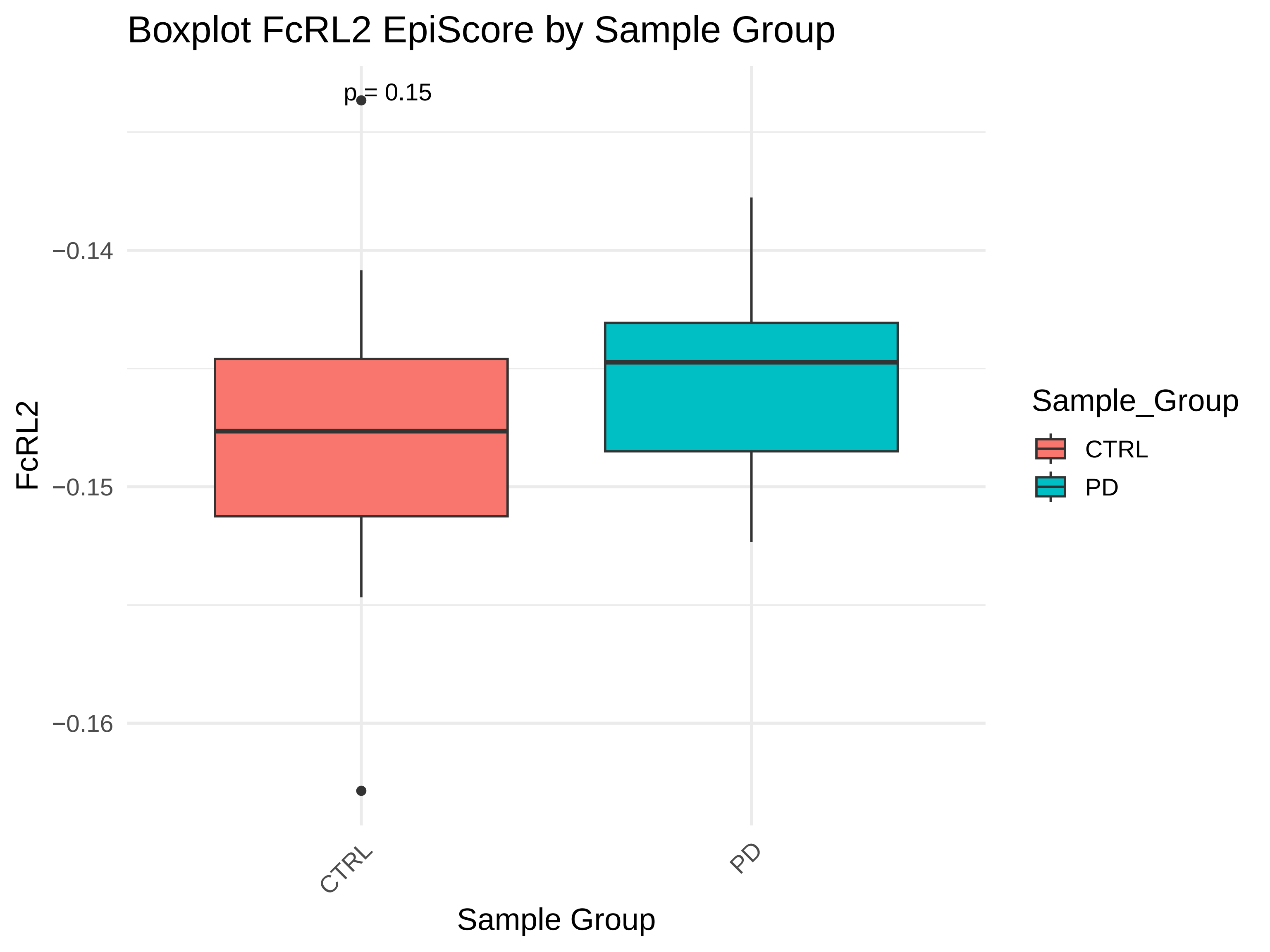
Boxplot FcRL2 EpiScore by Sample Group
p = 0.15
FcRL2
−0.14
−0.15
−0.16
CTRL
PD
Sample Group
Sample_Group
CTRL
PD

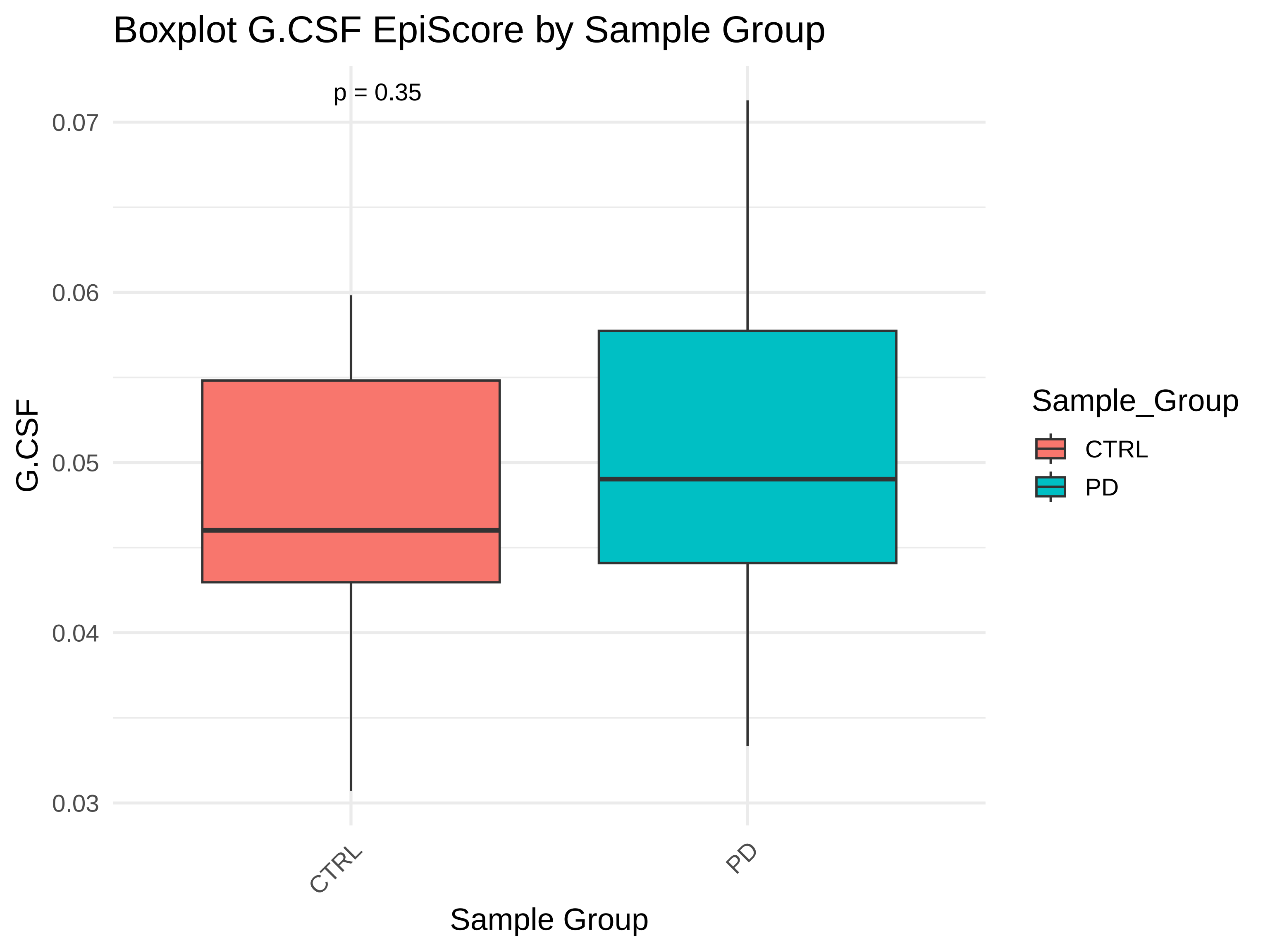

Boxplot G.CSF EpiScore by Sample Group
p = 0.35
0.07
0.06
0.05
0.04
0.03
G.CSF
CTRL
PD
Sample Group
Sample_Group
CTRL
PD

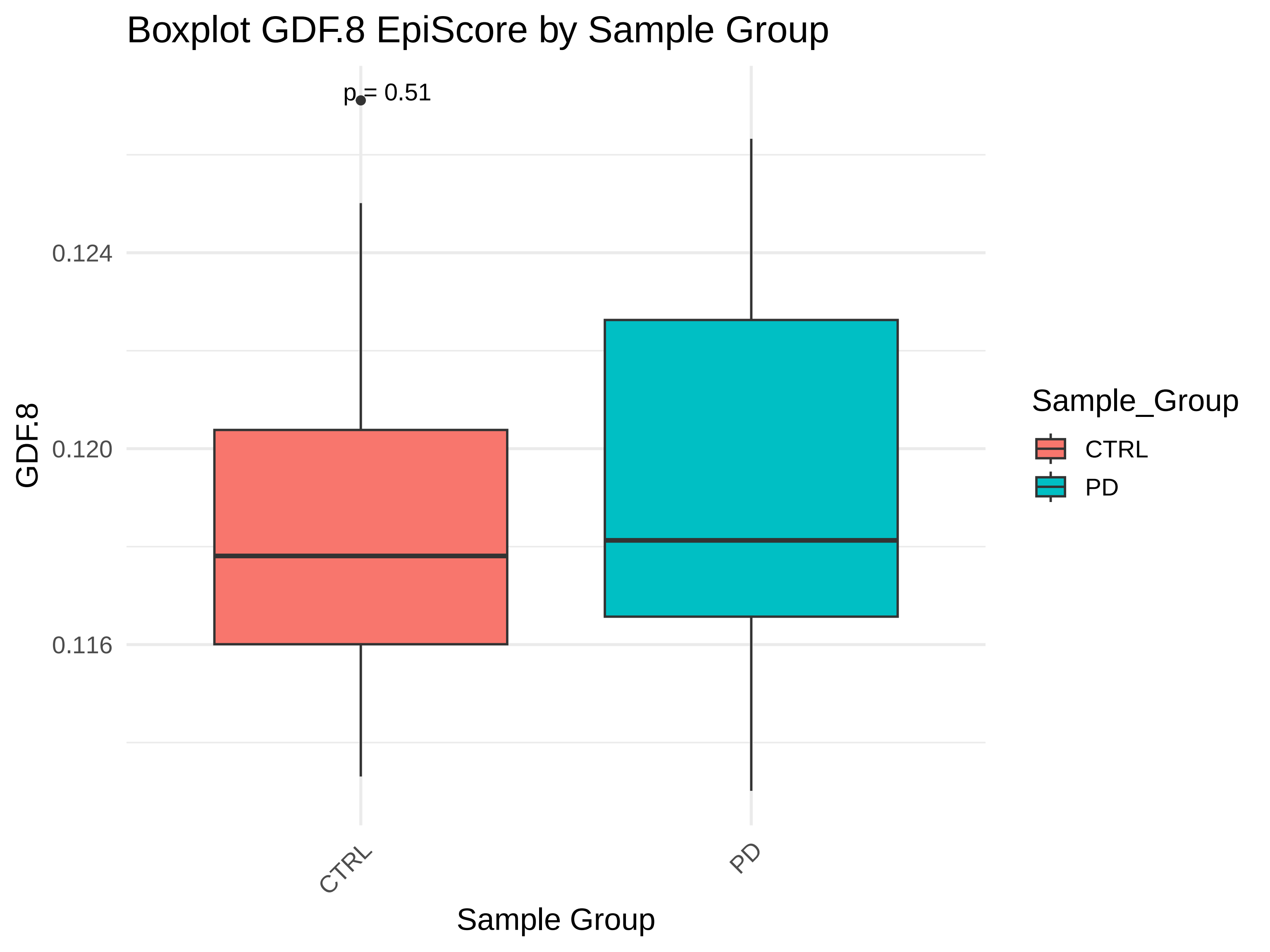
Boxplot GDF.8 EpiScore by Sample Group
p = 0.51
0.124
0.120
0.116
GDF.8
CTRL
PD
Sample Group
Sample_Group
CTRL
PD

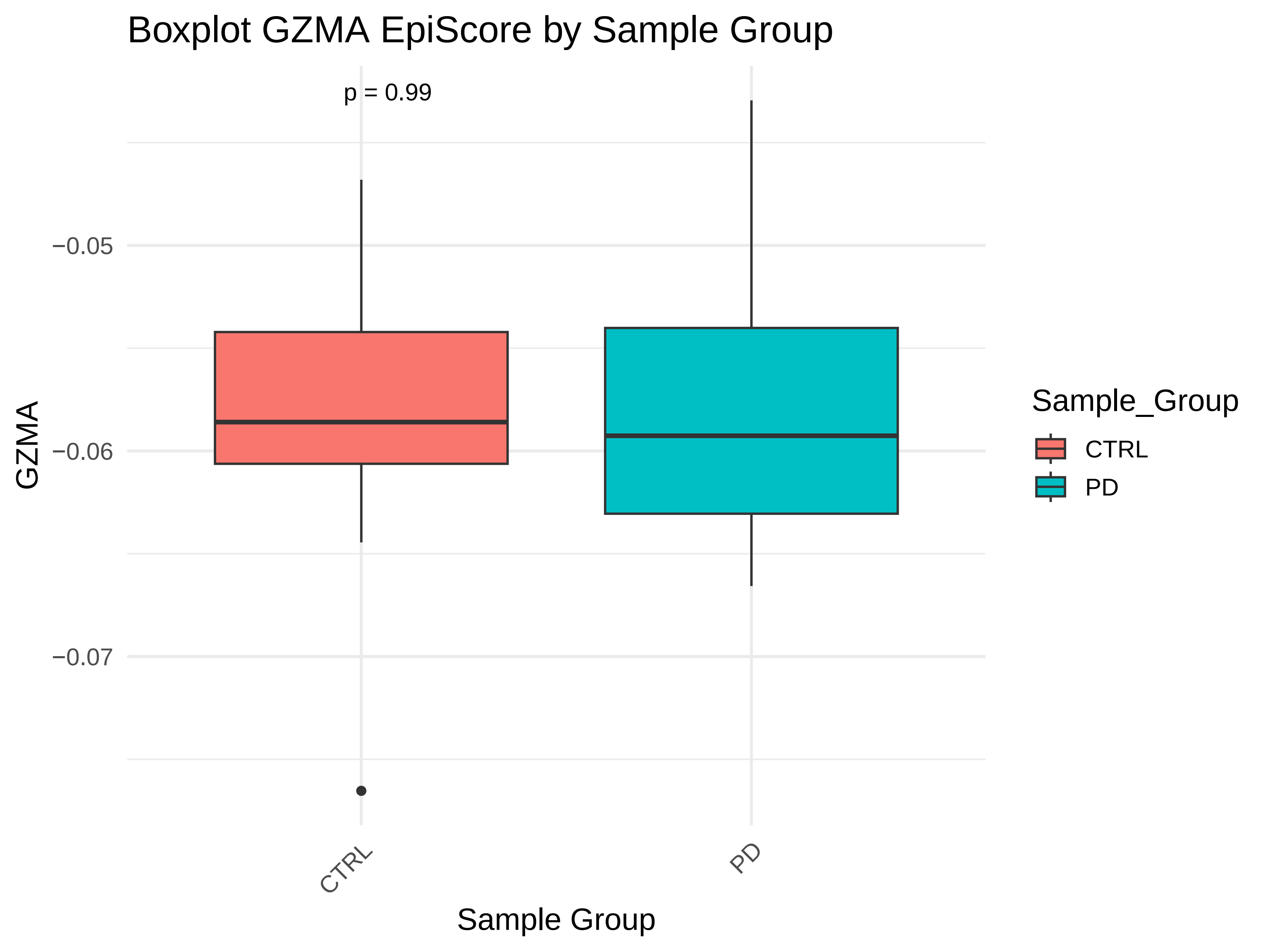
Boxplot GZMA EpiScore by Sample Group
p = 0.99
−0.05
−0.06
−0.07
GZMA
CTRL
PD
Sample Group
Sample_Group
CTRL
PD

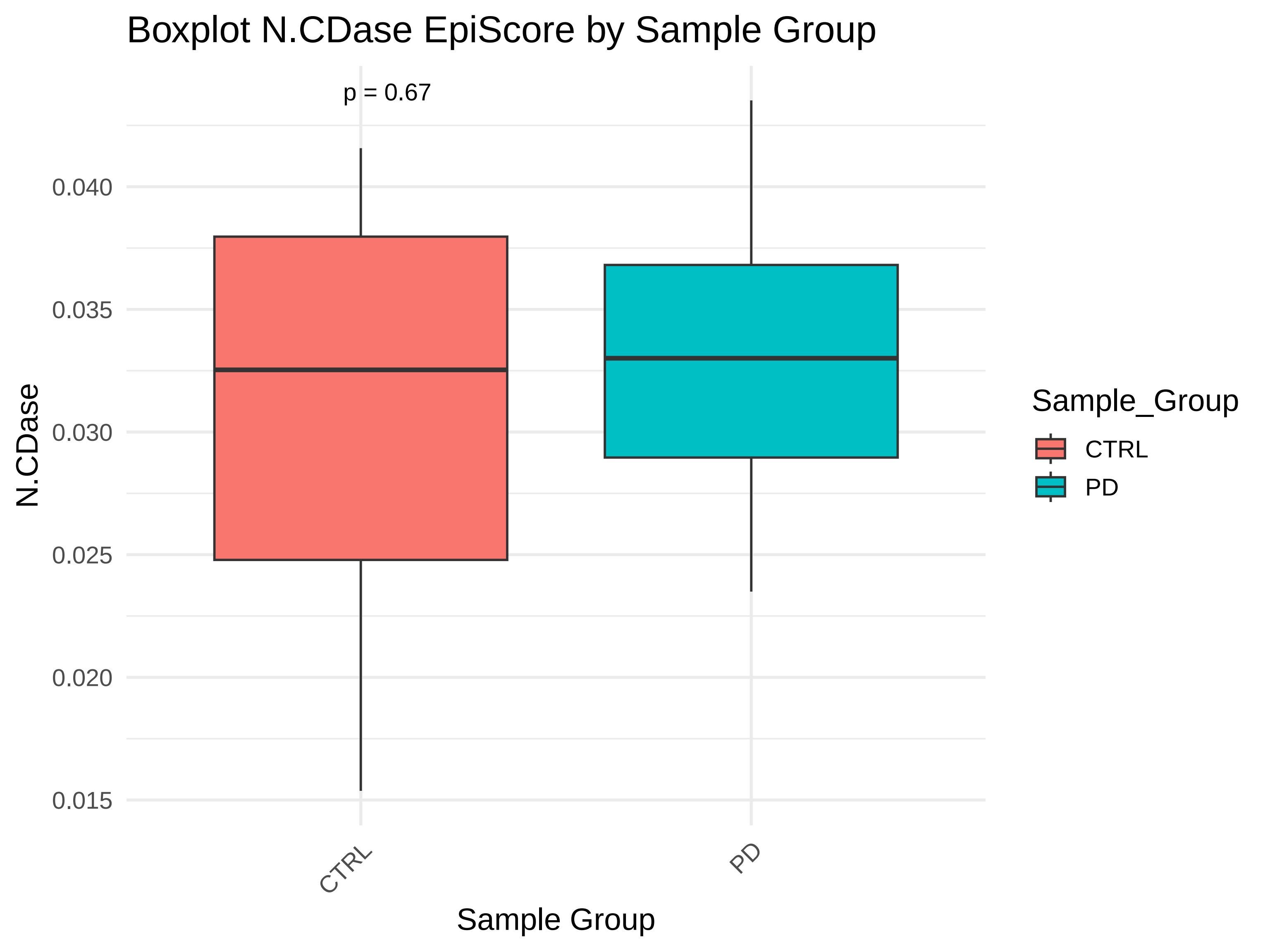
Boxplot N.CDase EpiScore by Sample Group
p = 0.67
0.040
0.035
0.030
0.025
0.020
0.015
N.CDase
CTRL
PD
Sample Group
Sample_Group
CTRL
PD

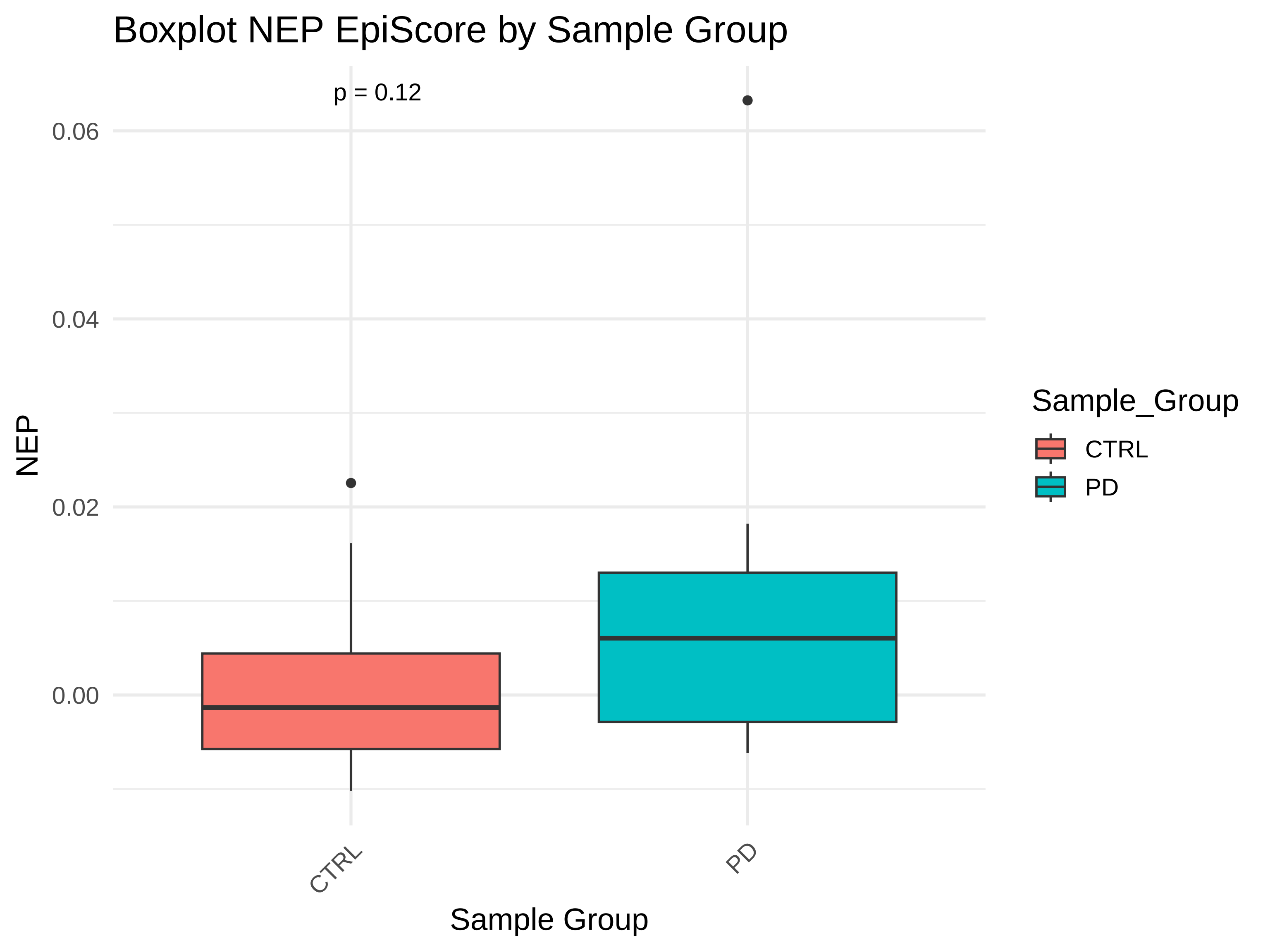
Boxplot NEP EpiScore by Sample Group
p = 0.12
0.06
0.04
0.02
0.00
NEP
CTRL
PD
Sample Group
Sample_Group
CTRL
PD

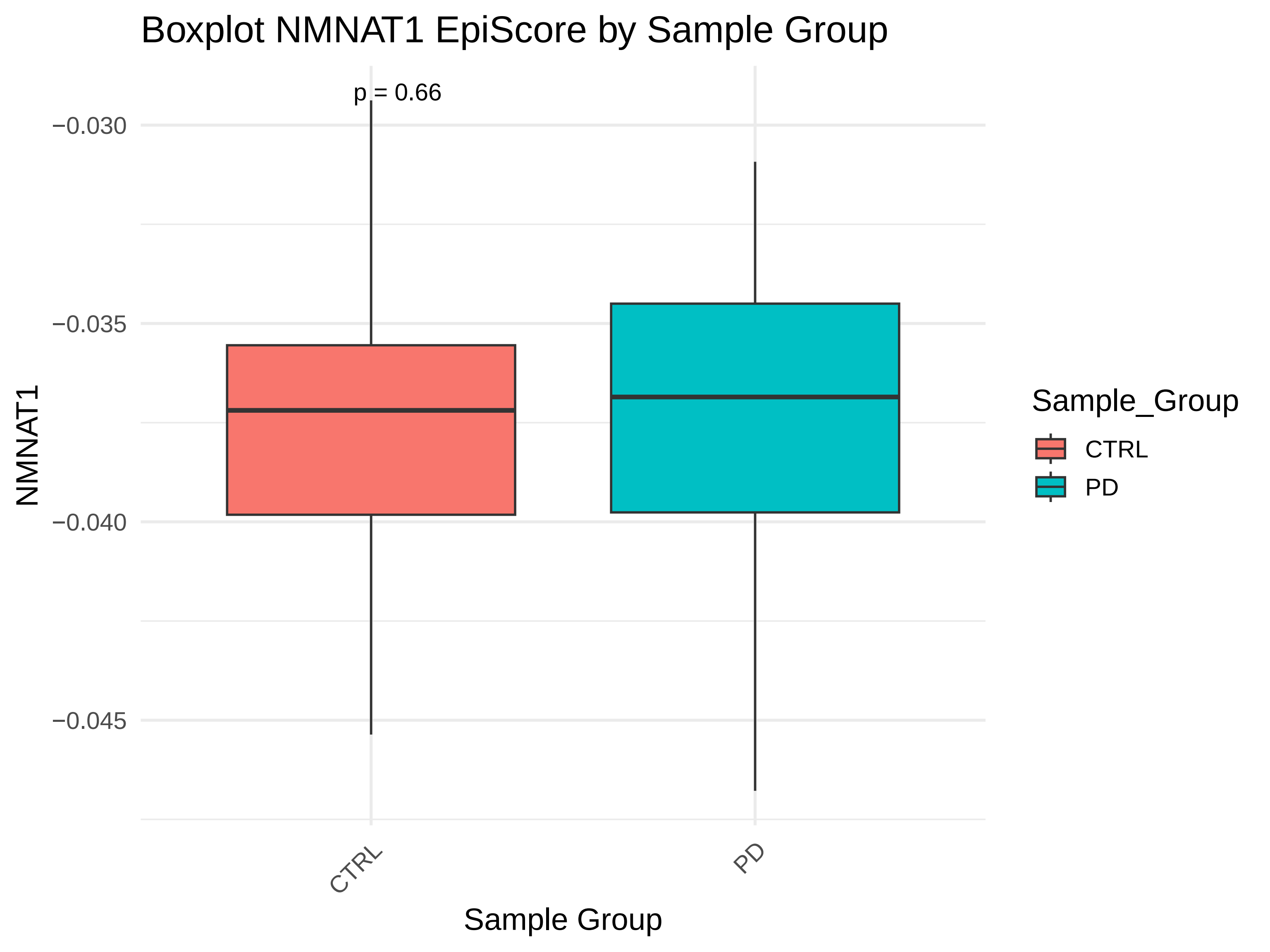
Boxplot NMNAT1 EpiScore by Sample Group
p = 0.66
−0.030
−0.035
−0.040
−0.045
NMNAT1
CTRL
PD
Sample Group
Sample_Group
CTRL
PD

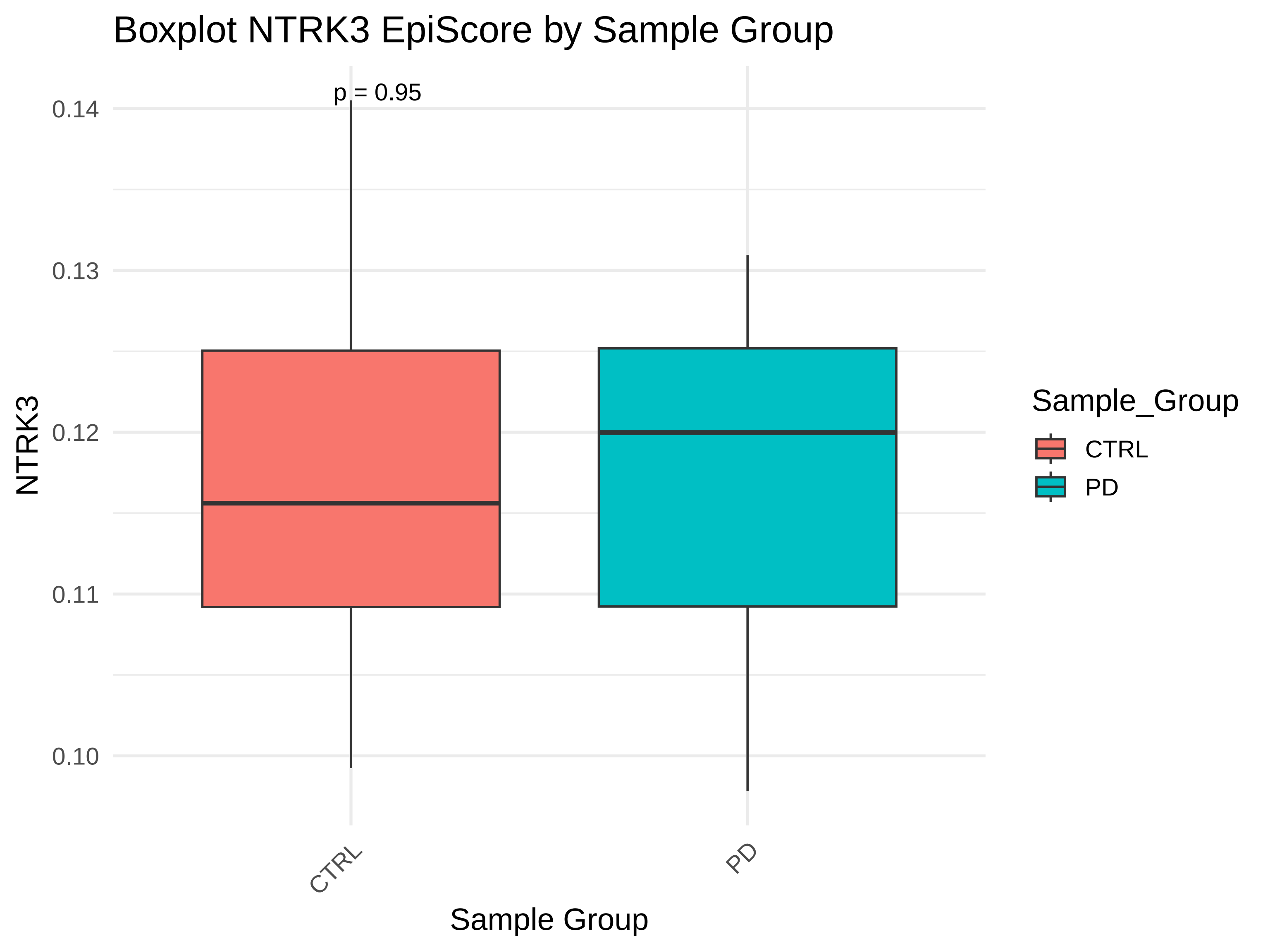

Boxplot NTRK3 EpiScore by Sample Group
p = 0.95
0.14
0.13
0.12
0.11
0.10
NTRK3
CTRL
PD
Sample Group
Sample_Group
CTRL
PD

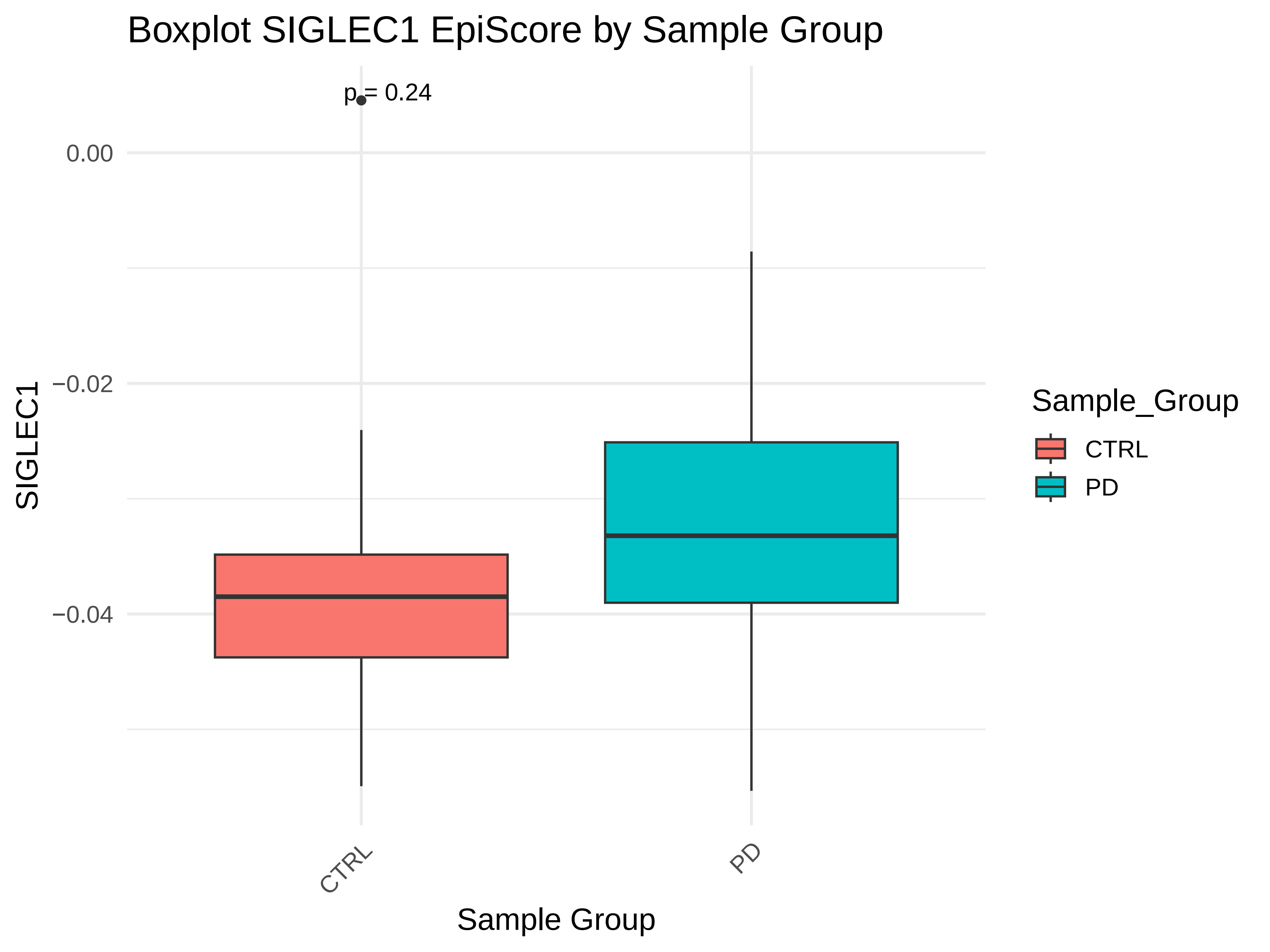
Boxplot SIGLEC1 EpiScore by Sample Group
p = 0.24
0.00
−0.02
−0.04
SIGLEC1
CTRL
PD
Sample Group
Sample_Group
CTRL
PD

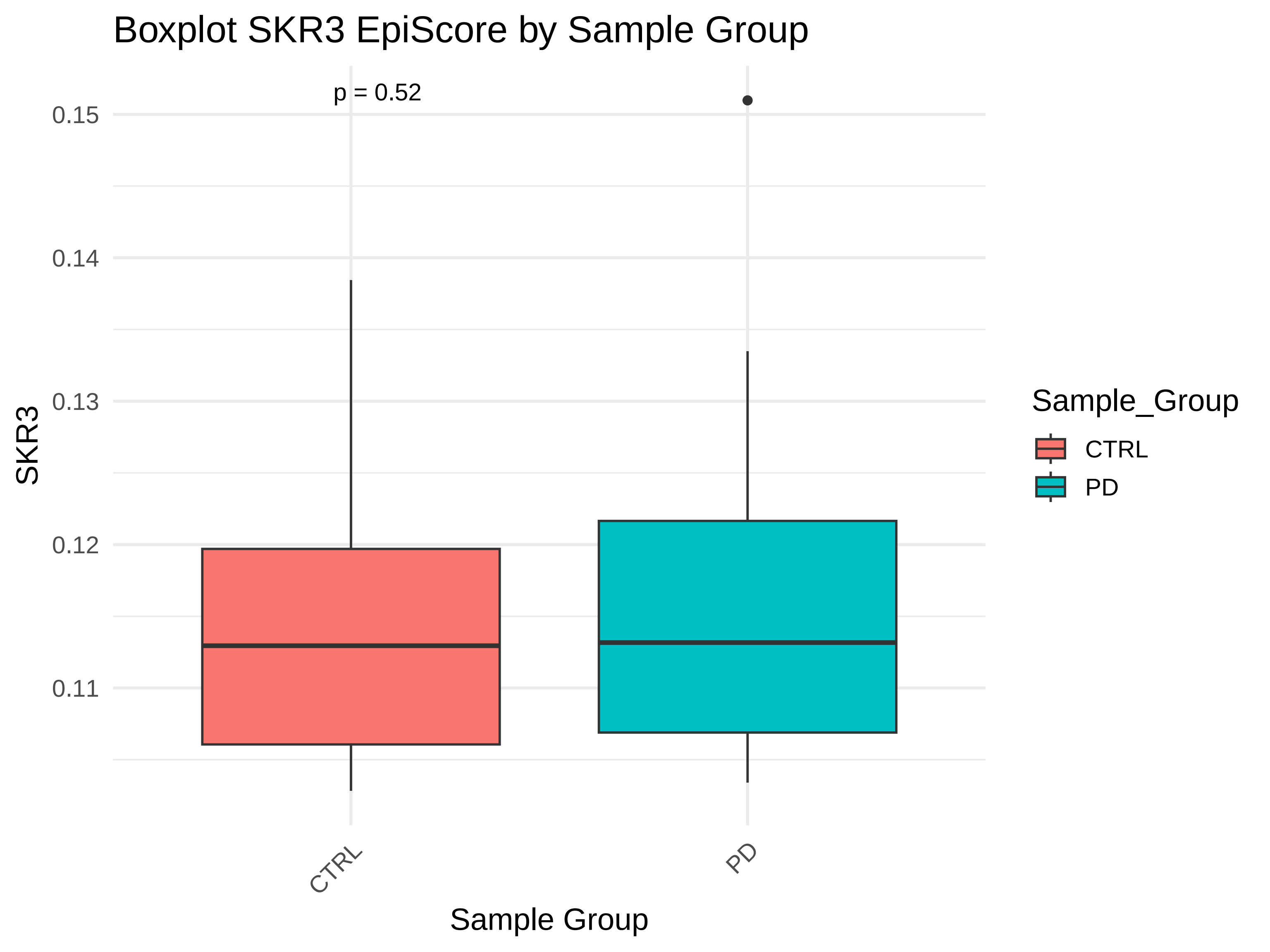

Boxplot SKR3 EpiScore by Sample Group
p = 0.52
0.15
0.14
0.13
0.12
0.11
SKR3
CTRL
PD
Sample Group
Sample_Group
CTRL
PD

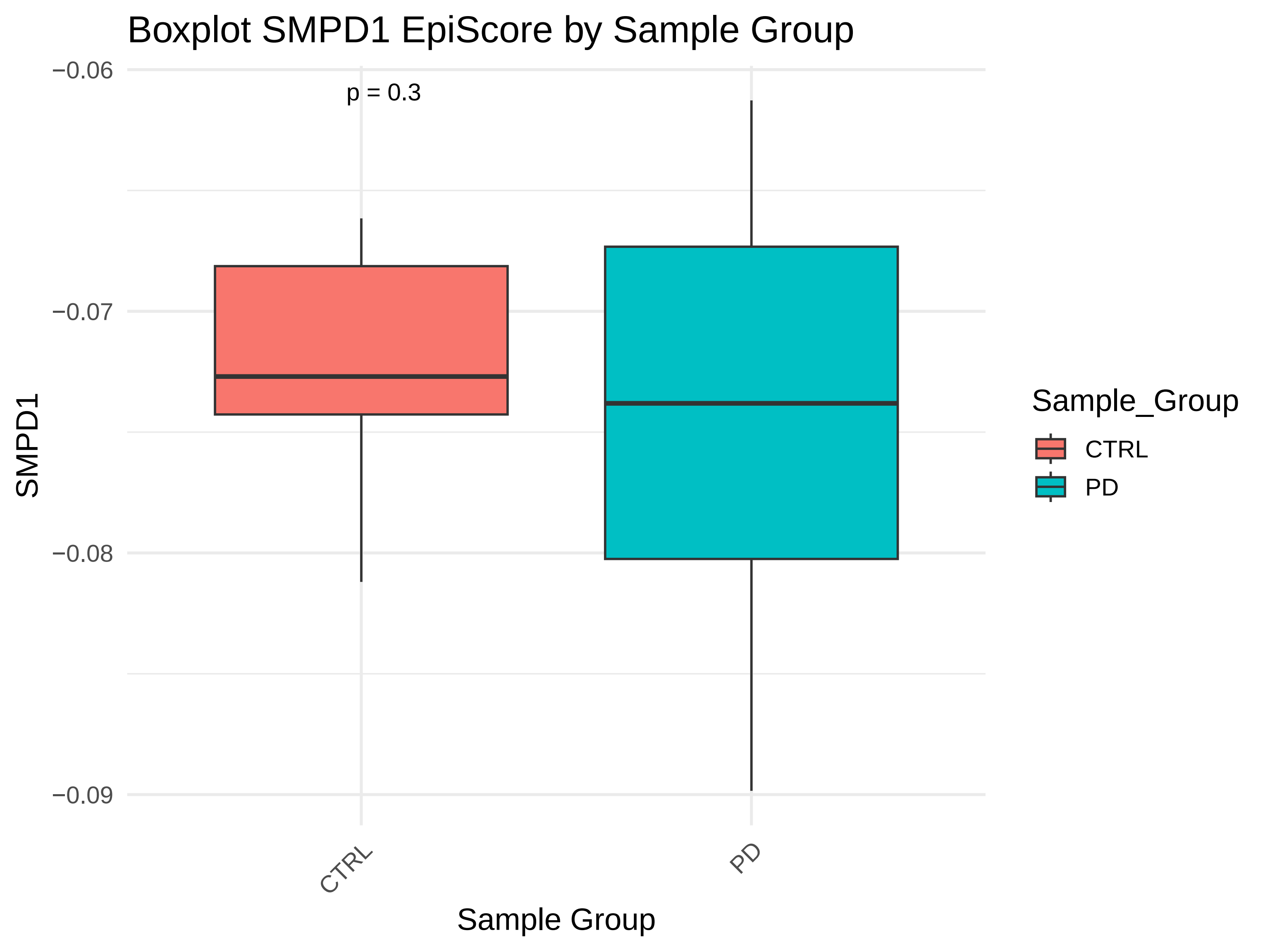

Boxplot SMPD1 EpiScore by Sample Group
p = 0.3
−0.06
−0.07
−0.08
−0.09
SMPD1
CTRL
PD
Sample Group
Sample_Group
CTRL
PD

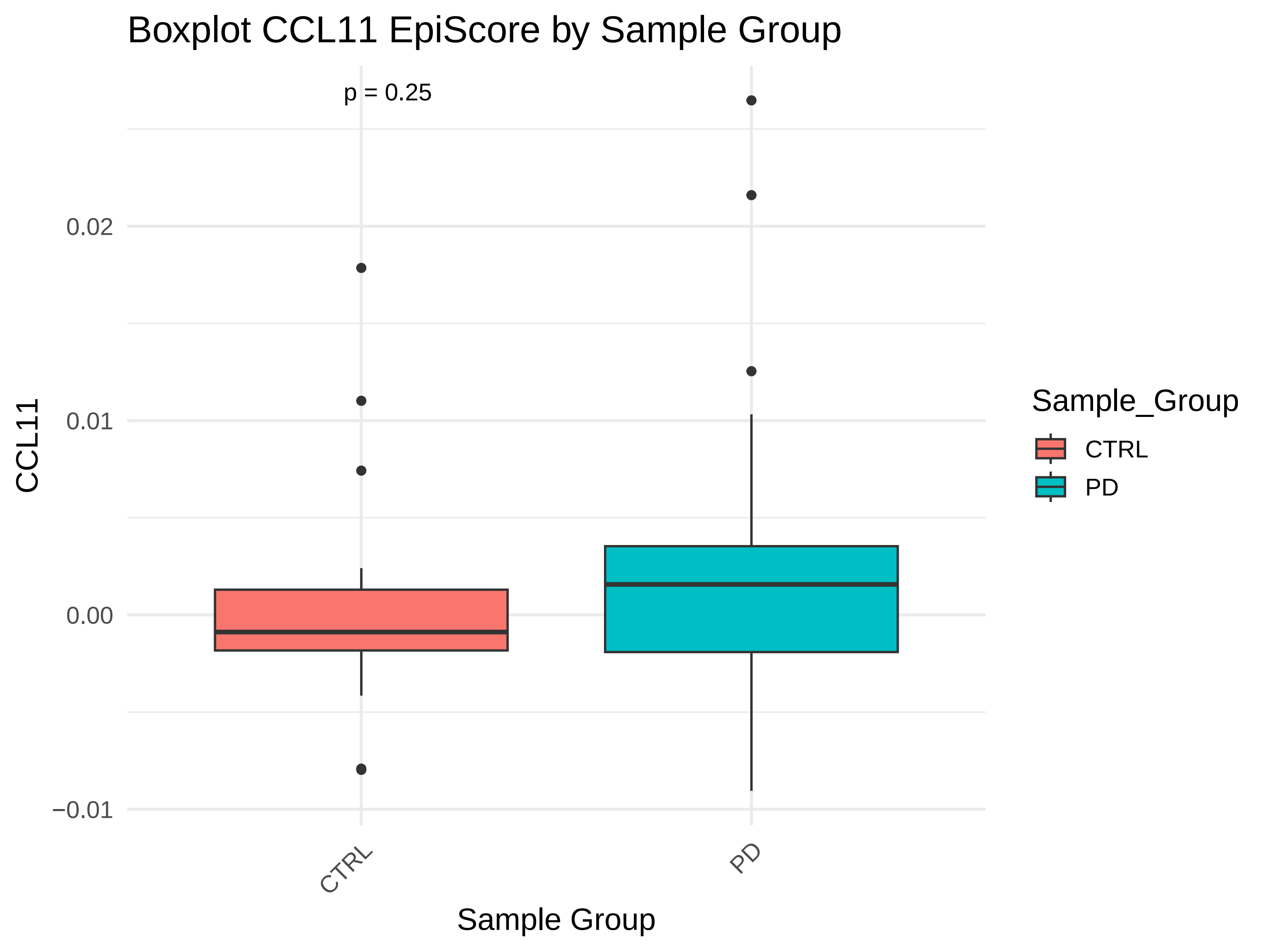

Boxplot CCL11 EpiScore by Sample Group
p = 0.25
CCL11
0.02
0.01
0.00
−0.01
CTRL
PD
Sample Group
Sample_Group
CTRL
PD

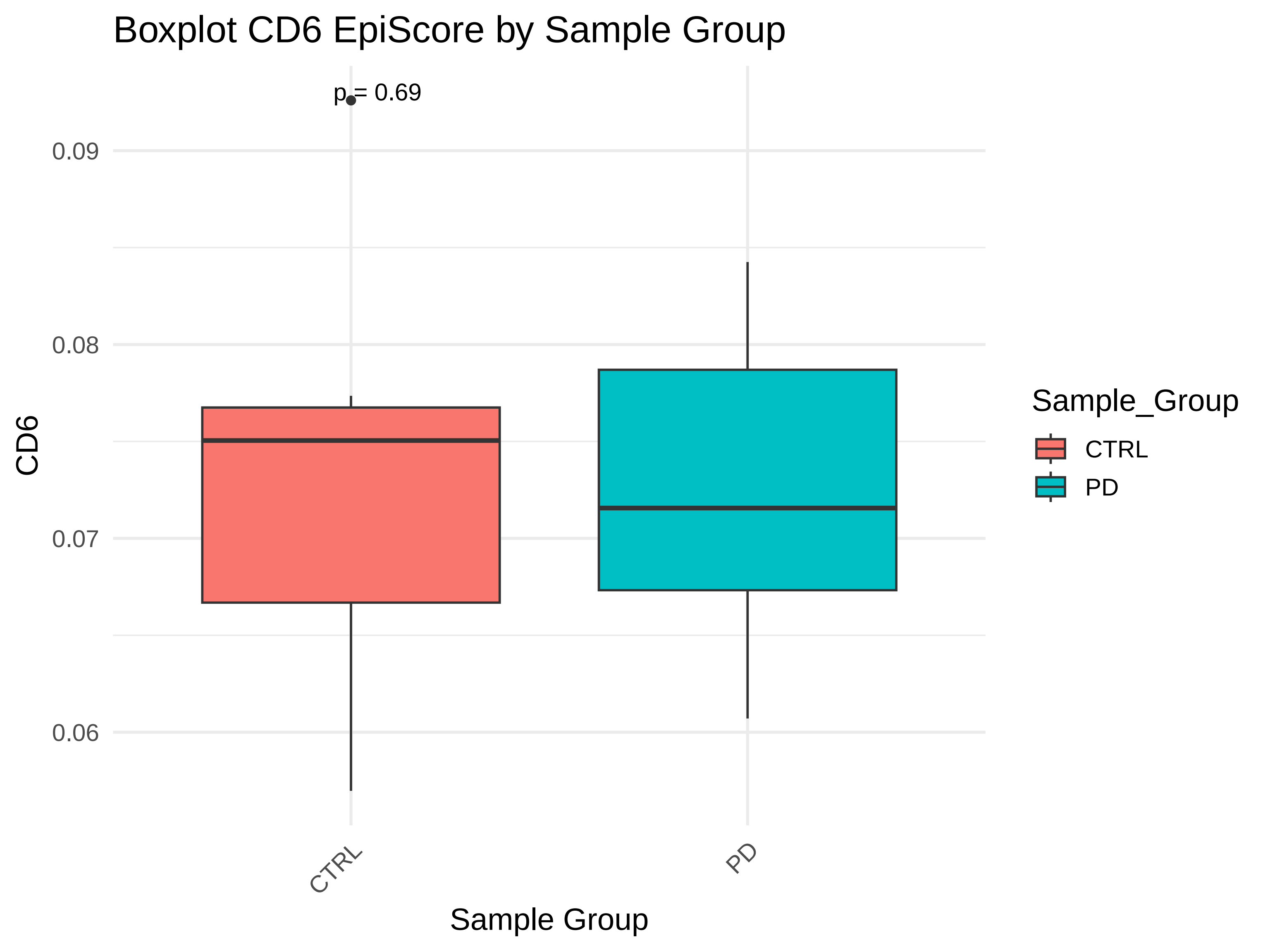
Boxplot CD6 EpiScore by Sample Group
p = 0.69
0.09
0.08
0.07
0.06
CD6
CTRL
PD
Sample Group
Sample_Group
CTRL
PD

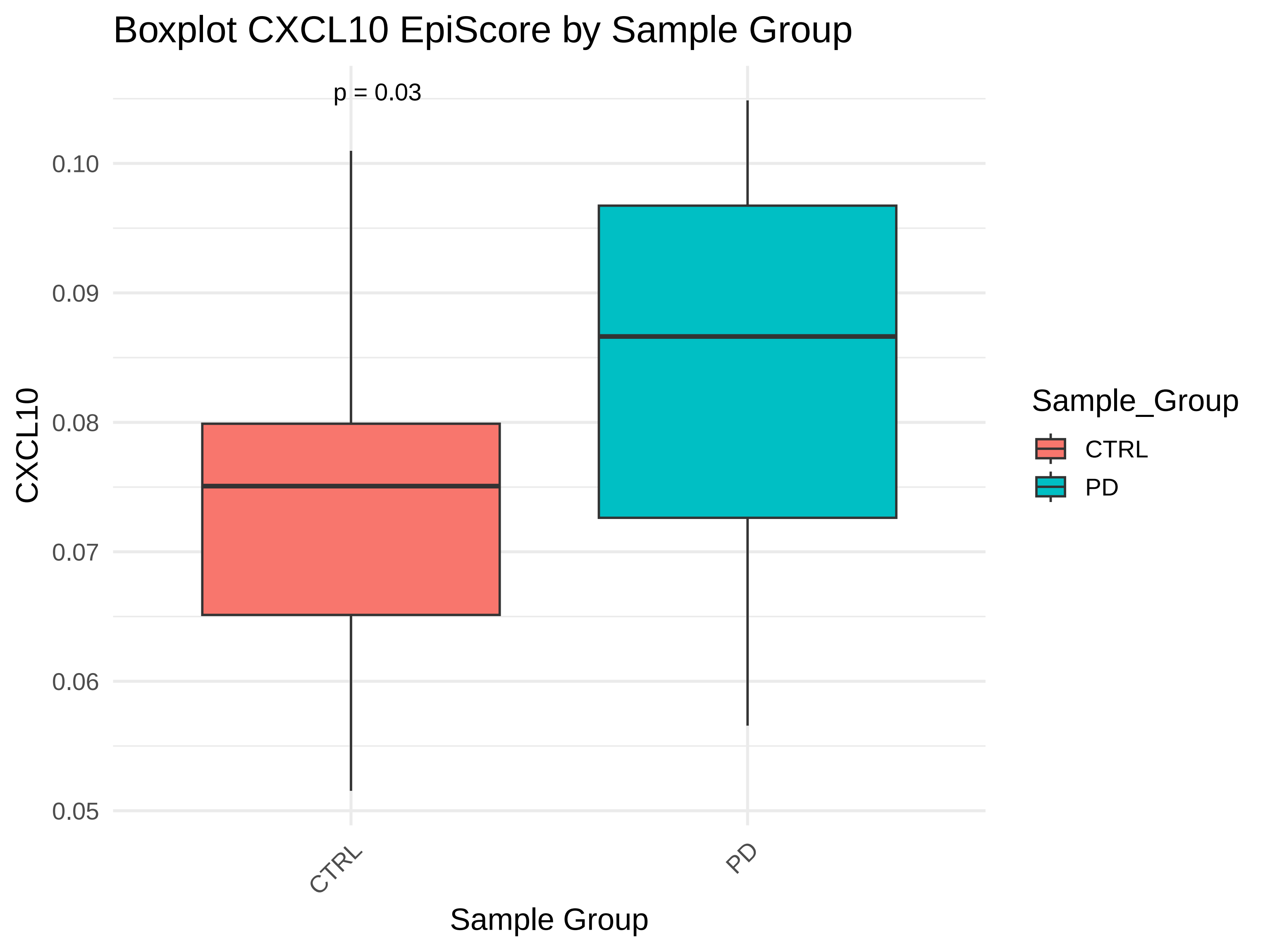
Boxplot CXCL10 EpiScore by Sample Group
p = 0.03
0.10
0.09
0.08
0.07
0.06
0.05
CXCL10
CTRL
PD
Sample Group
Sample_Group
CTRL
PD

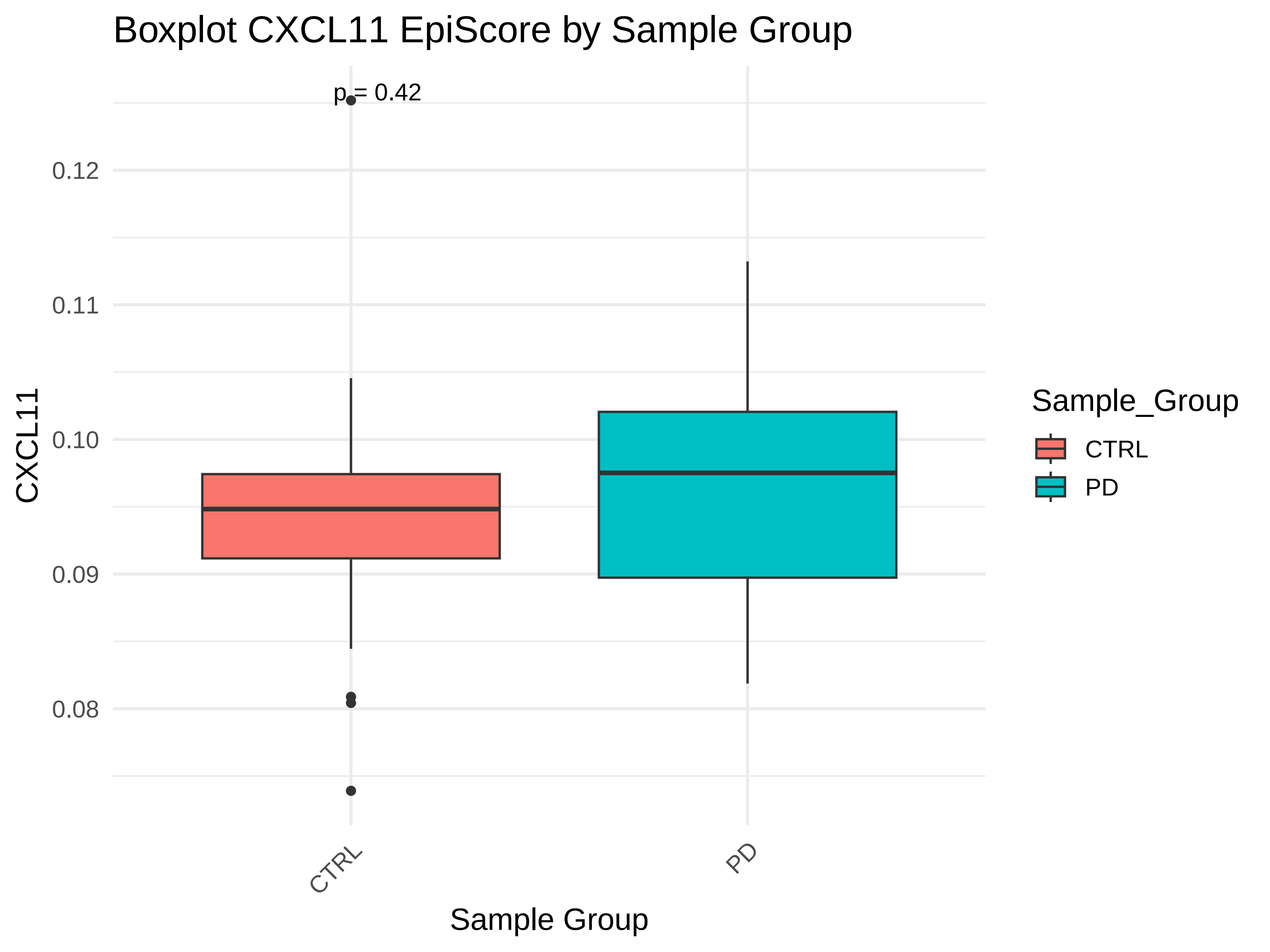
Boxplot CXCL11 EpiScore by Sample Group
p = 0.42
0.12
0.11
0.10
0.09
0.08
CXCL11
CTRL
PD
Sample Group
Sample_Group
CTRL
PD

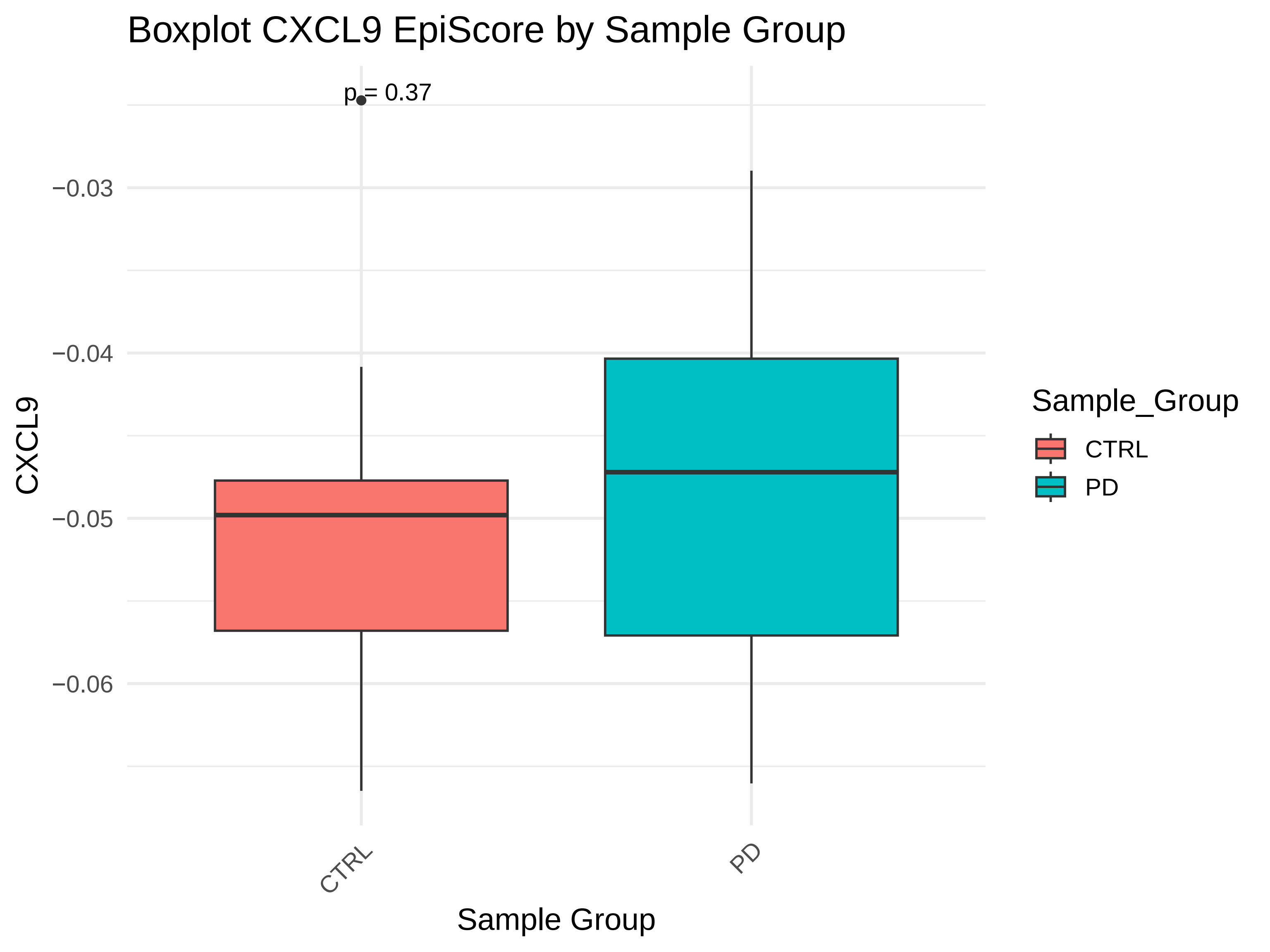

Boxplot CXCL9 EpiScore by Sample Group
p = 0.37
−0.03
−0.04
−0.05
−0.06
CXCL9
CTRL
PD
Sample Group
Sample_Group
CTRL
PD

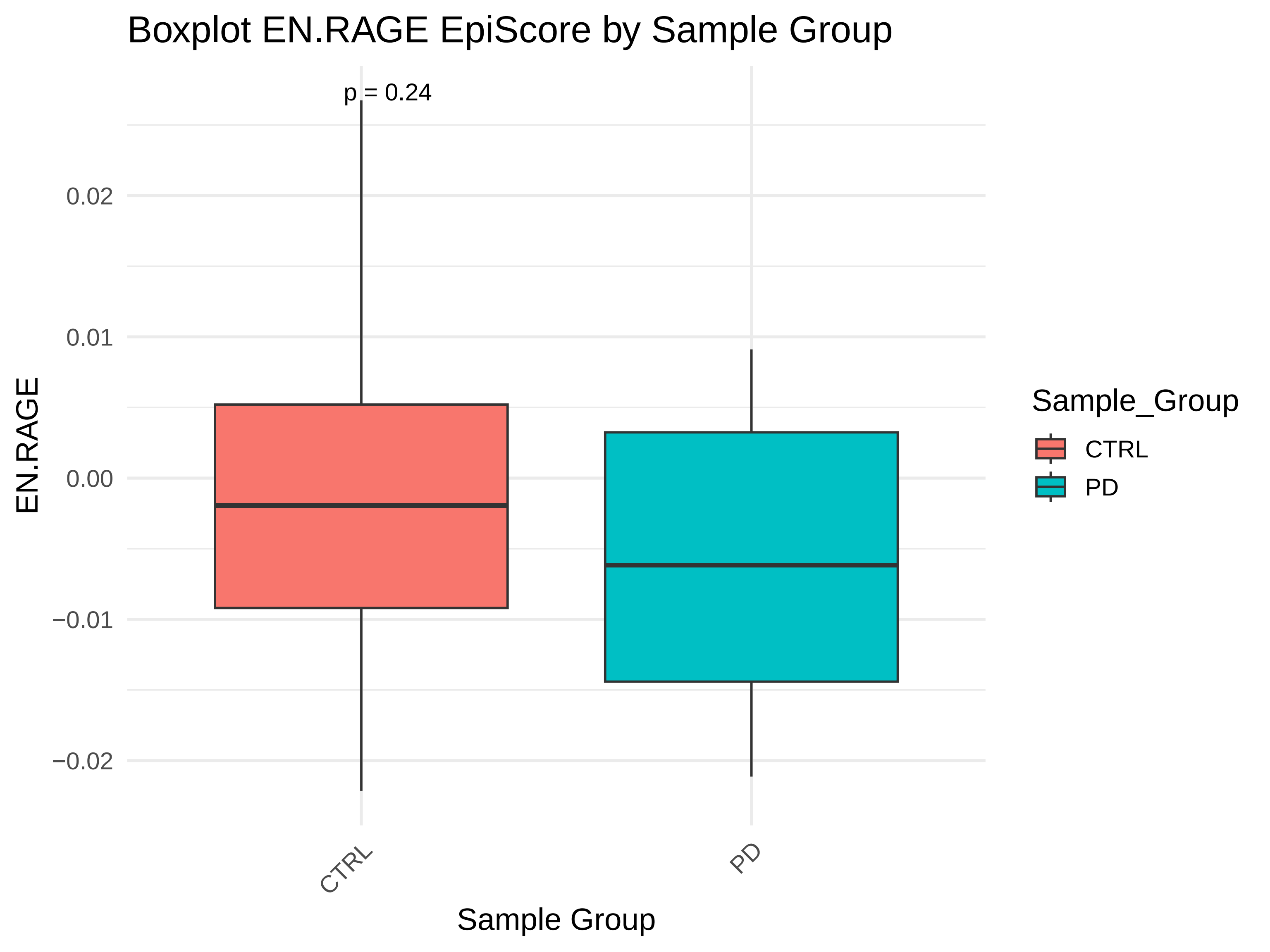
Boxplot EN.RAGE EpiScore by Sample Group
p = 0.24
0.02
0.01
0.00
−0.01
−0.02
EN.RAGE
CTRL
PD
Sample Group
Sample_Group
CTRL
PD

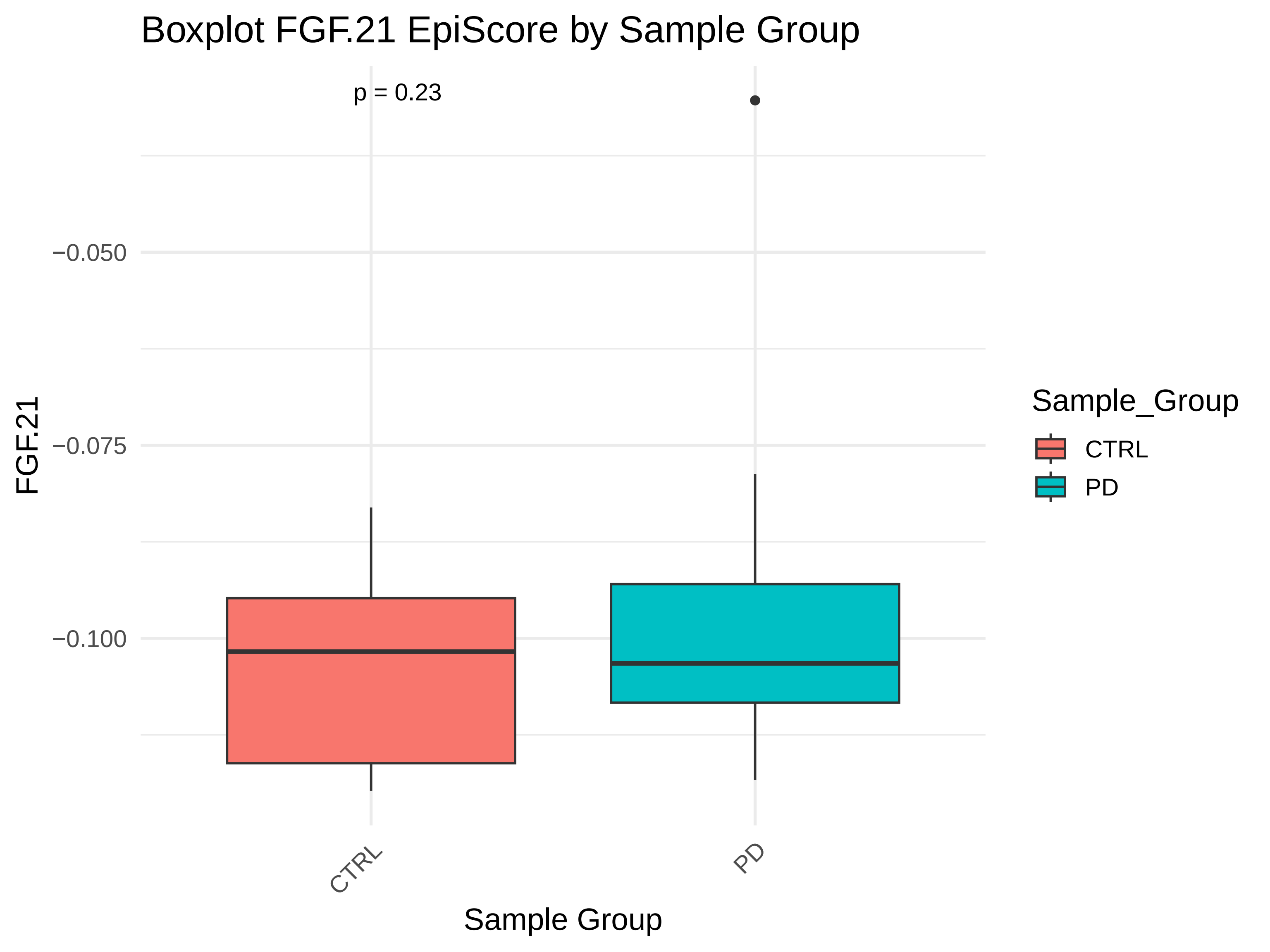
Boxplot FGF.21 EpiScore by Sample Group
p = 0.23
−0.050
−0.075
−0.100
FGF.21
CTRL
PD
Sample Group
Sample_Group
CTRL
PD

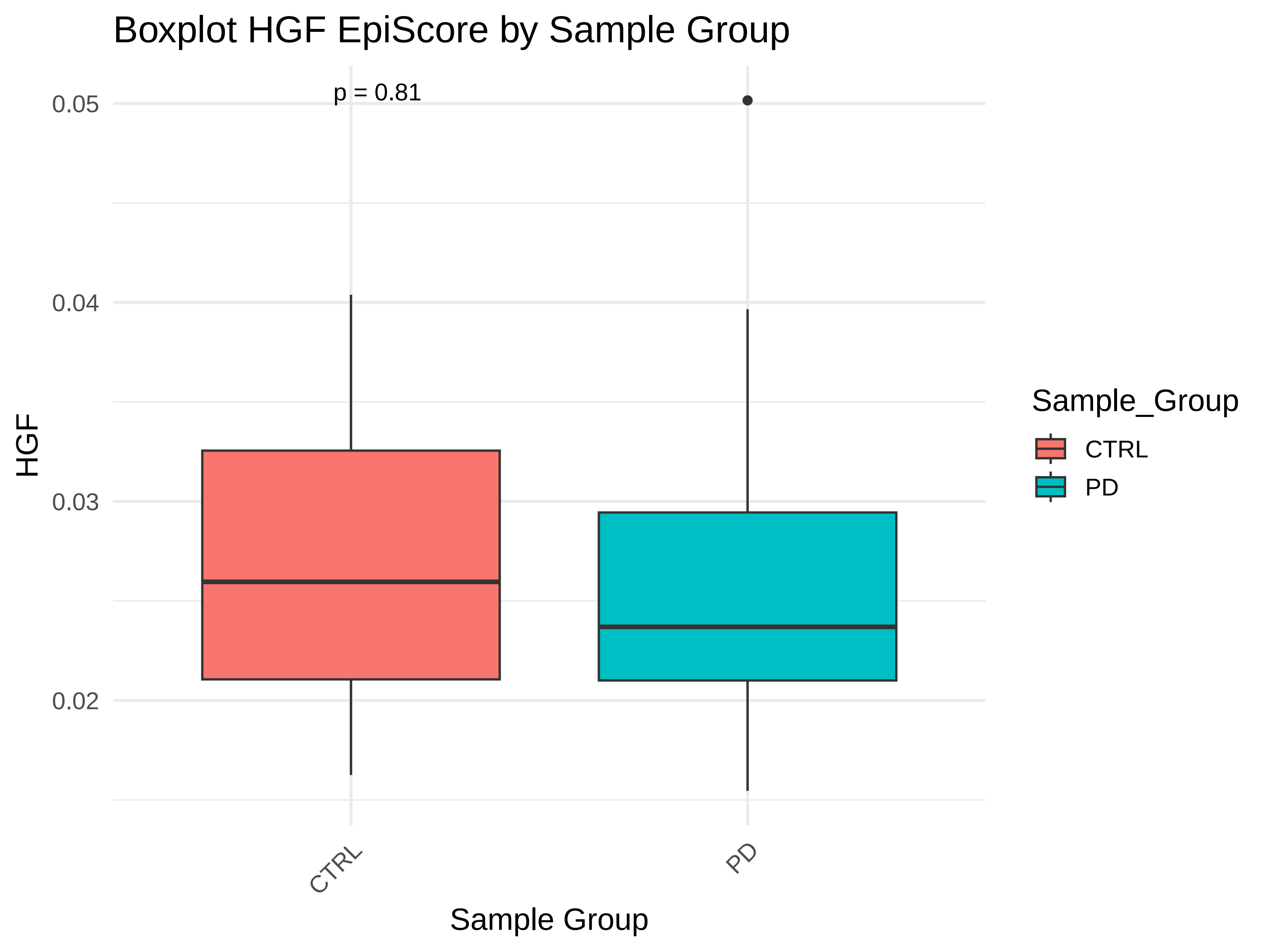

Boxplot HGF EpiScore by Sample Group
p = 0.81
0.05
0.04
0.03
0.02
HGF
CTRL
PD
Sample Group
Sample_Group
CTRL
PD

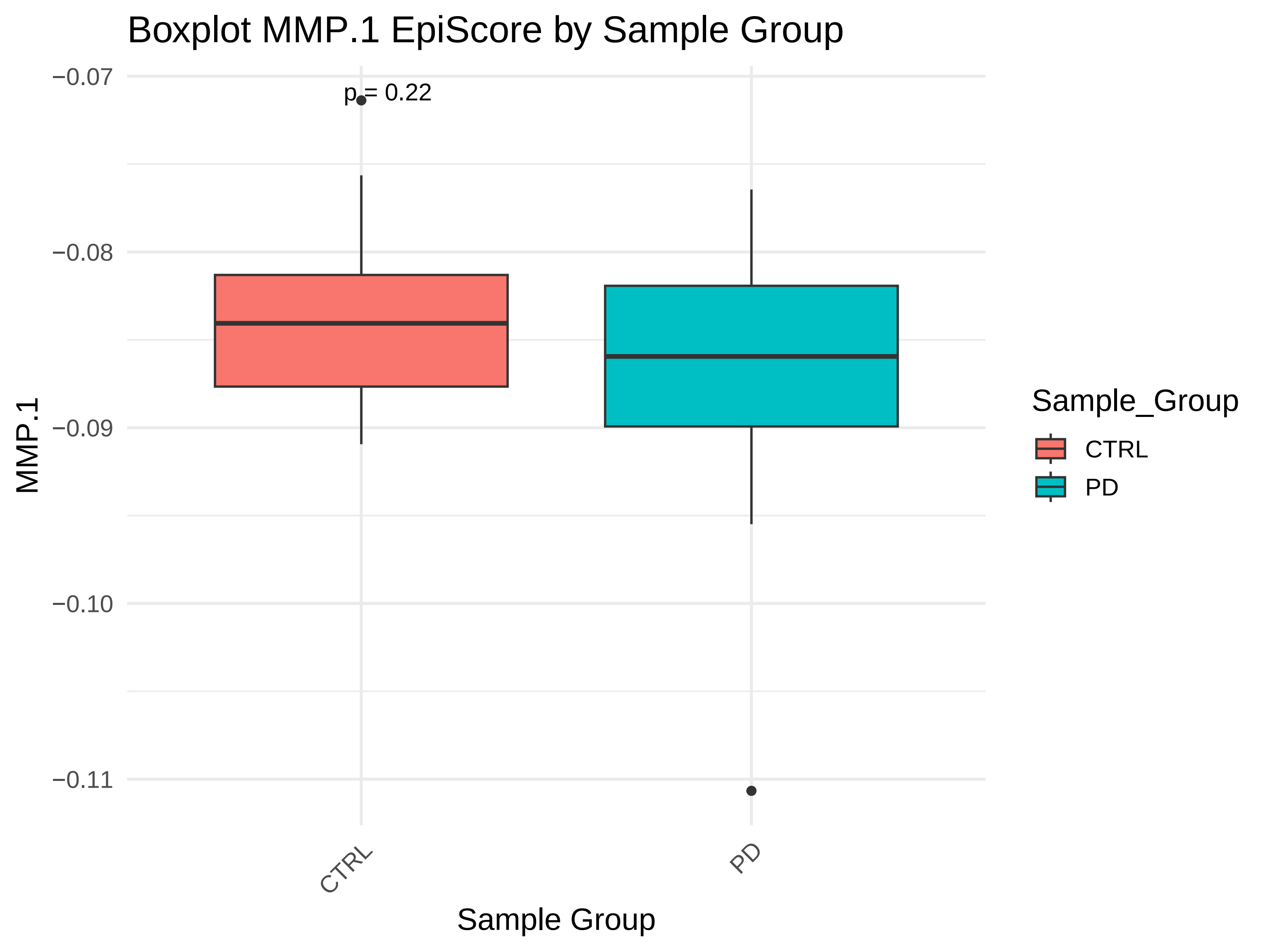
Boxplot MMP.1 EpiScore by Sample Group
p = 0.22
−0.07
−0.08
−0.09
−0.10
−0.11
MMP.1
CTRL
PD
Sample Group
Sample_Group
CTRL
PD

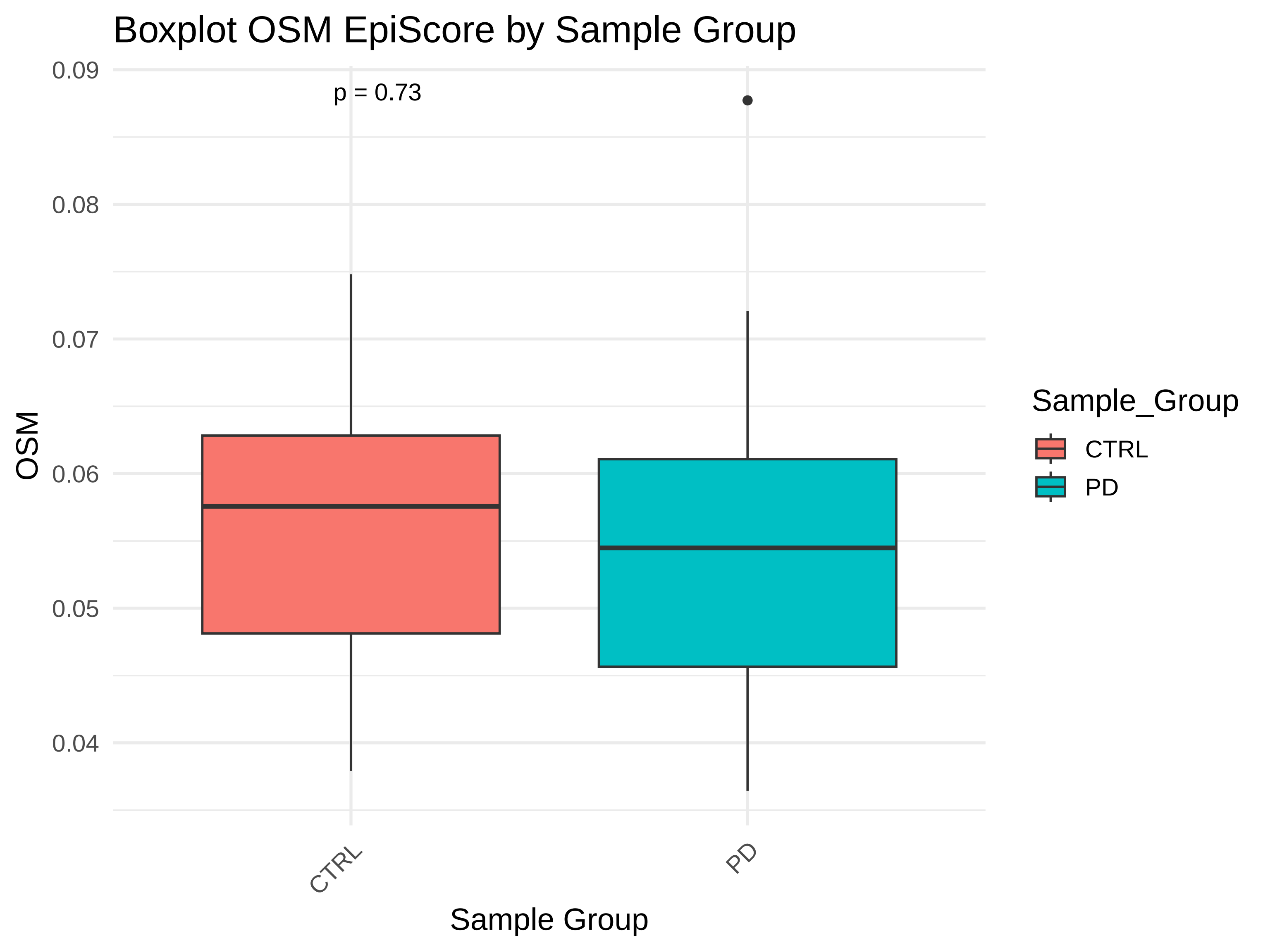

Boxplot OSM EpiScore by Sample Group
p = 0.73
0.09
0.08
0.07
0.06
0.05
0.04
OSM
CTRL
PD
Sample Group
Sample_Group
CTRL
PD

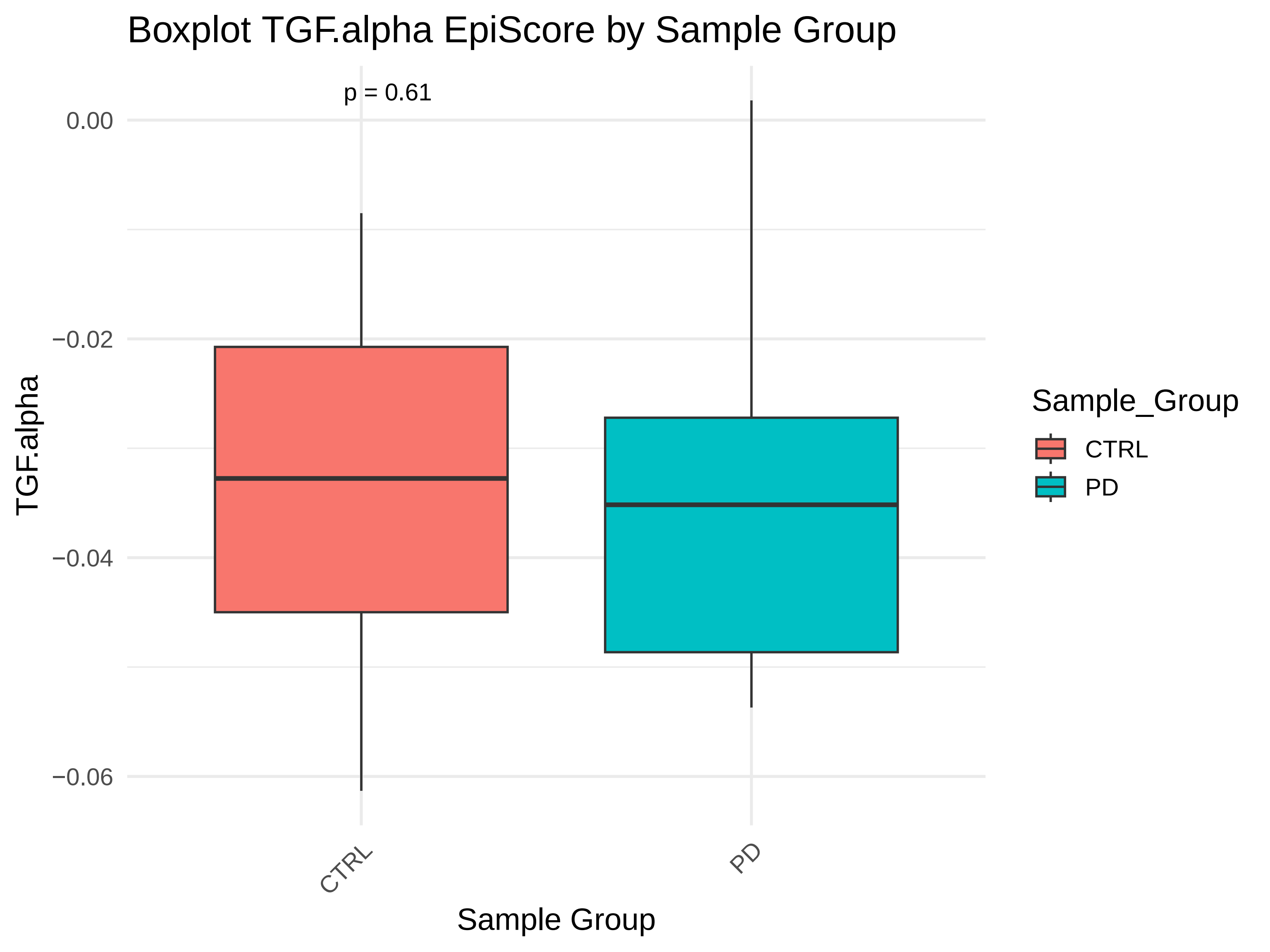

Boxplot TGF.alpha EpiScore by Sample Group
p = 0.61
0.00
−0.02
−0.04
−0.06
TGF.alpha
CTRL
PD
Sample Group
Sample_Group
CTRL
PD

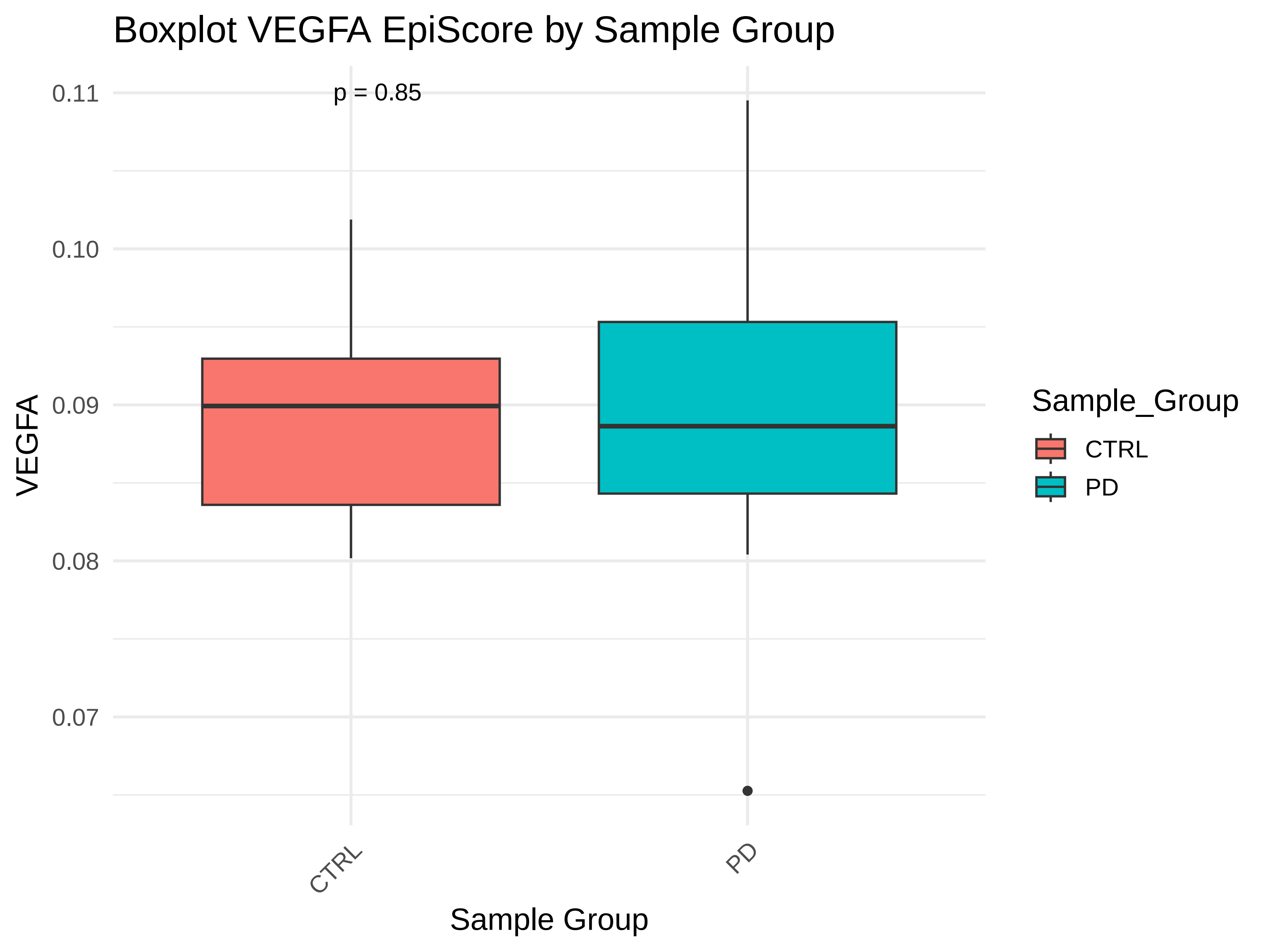

Boxplot VEGFA EpiScore by Sample Group
p = 0.85
0.11
0.10
0.09
0.08
0.07
VEGFA
CTRL
PD
Sample Group
Sample_Group
CTRL
PD

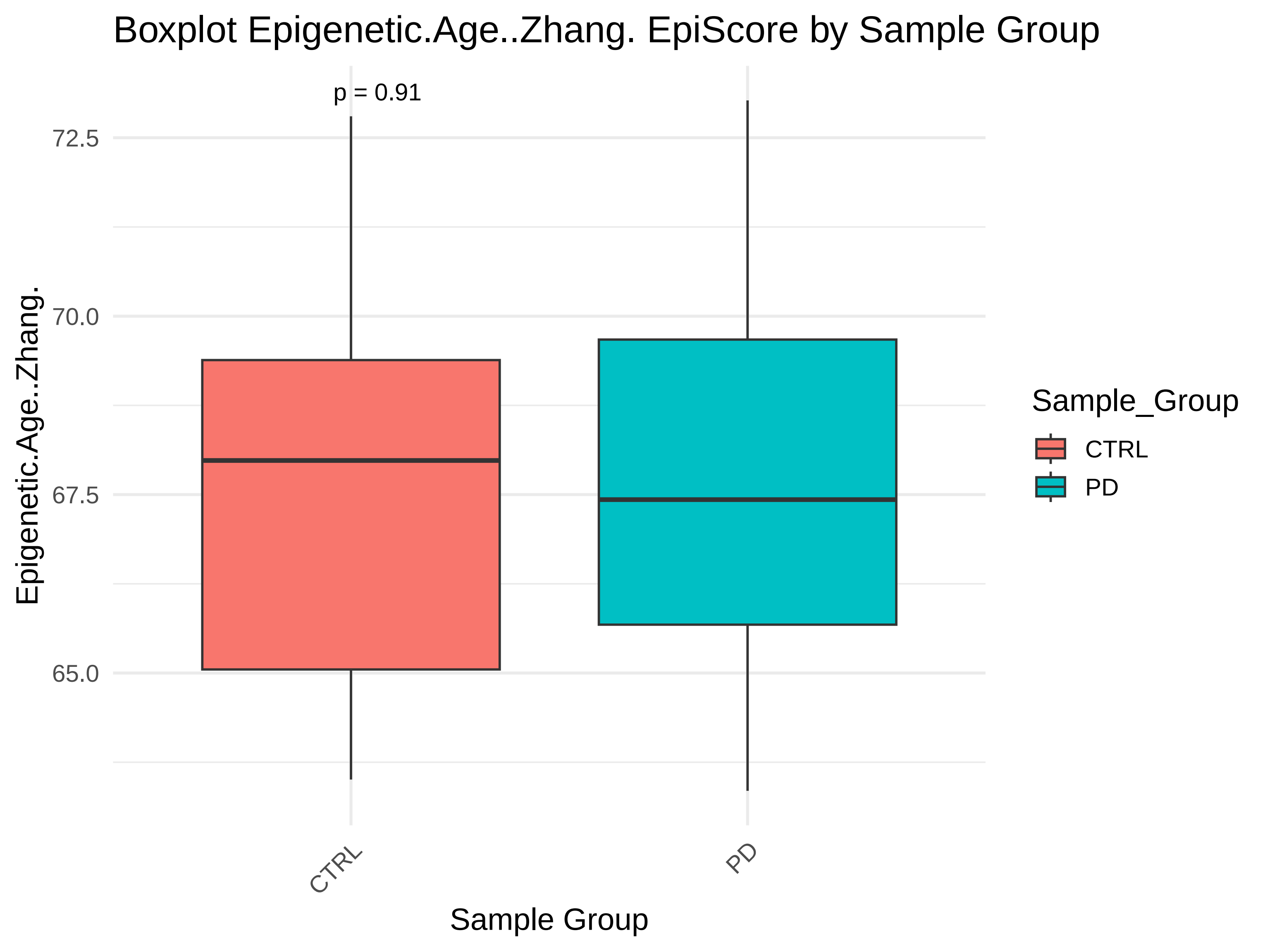
Boxplot Epigenetic.Age..Zhang. EpiScore by Sample Group
p = 0.91
72.5
70.0
67.5
65.0
Epigenetic.Age..Zhang.
CTRL
PD
Sample Group
Sample_Group
CTRL
PD

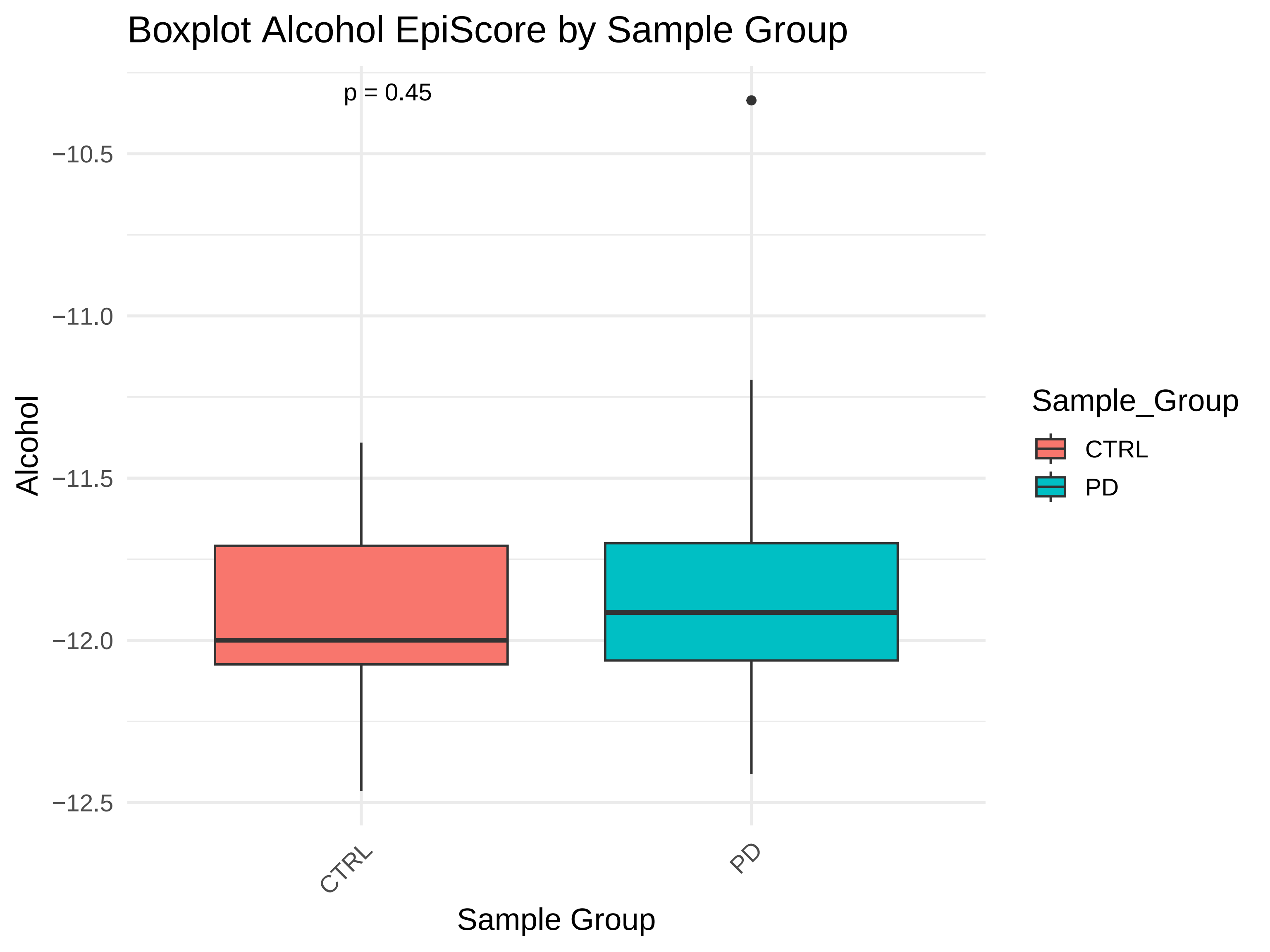

Boxplot Alcohol EpiScore by Sample Group
p = 0.45
−10.5
−11.0
−11.5
−12.0
−12.5
Alcohol
CTRL
PD
Sample Group
Sample_Group
CTRL
PD

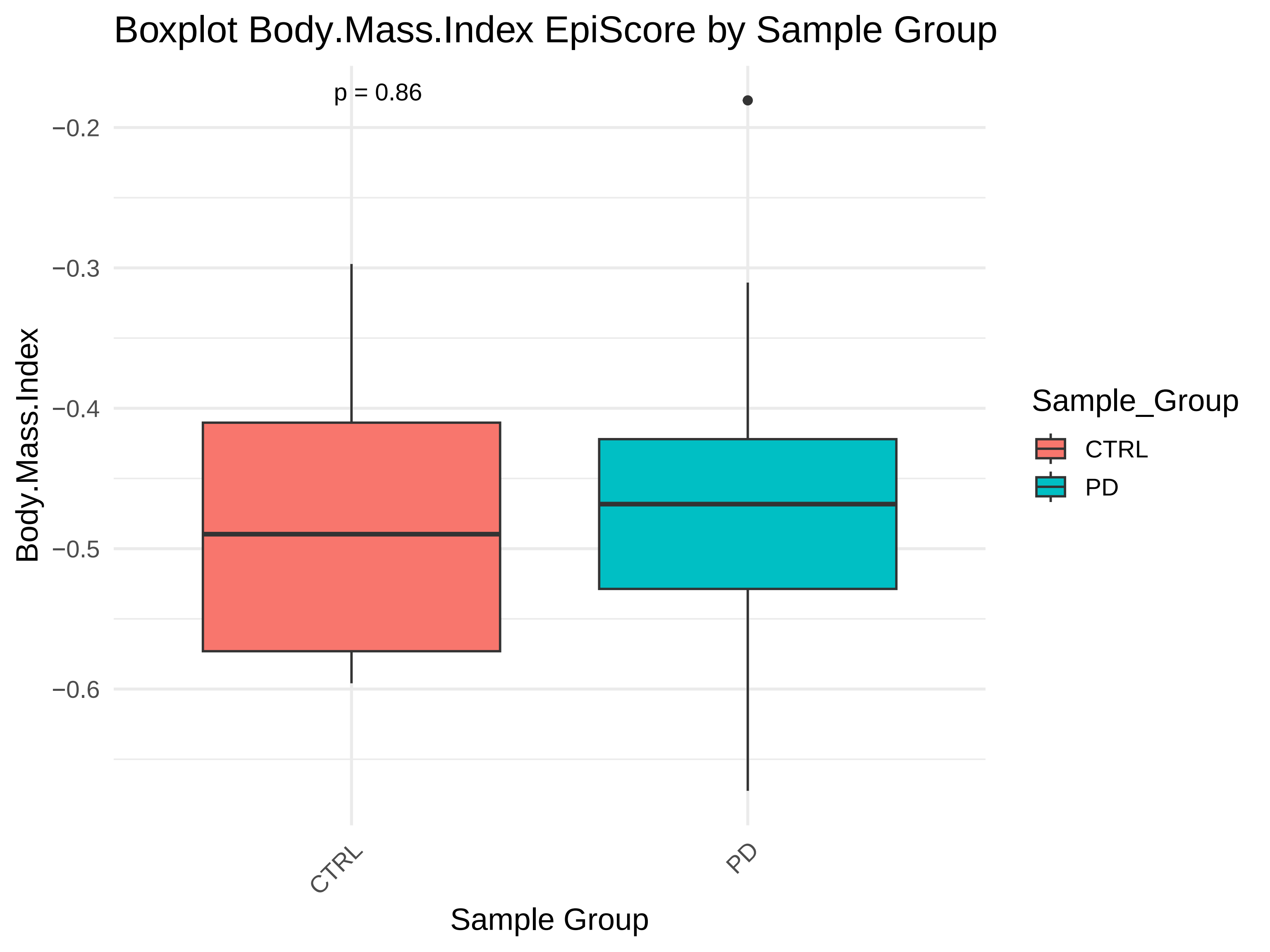
Boxplot Body.Mass.Index EpiScore by Sample Group
p = 0.86
−0.2
−0.3
−0.4
−0.5
−0.6
Body.Mass.Index
CTRL
PD
Sample Group
Sample_Group
CTRL
PD

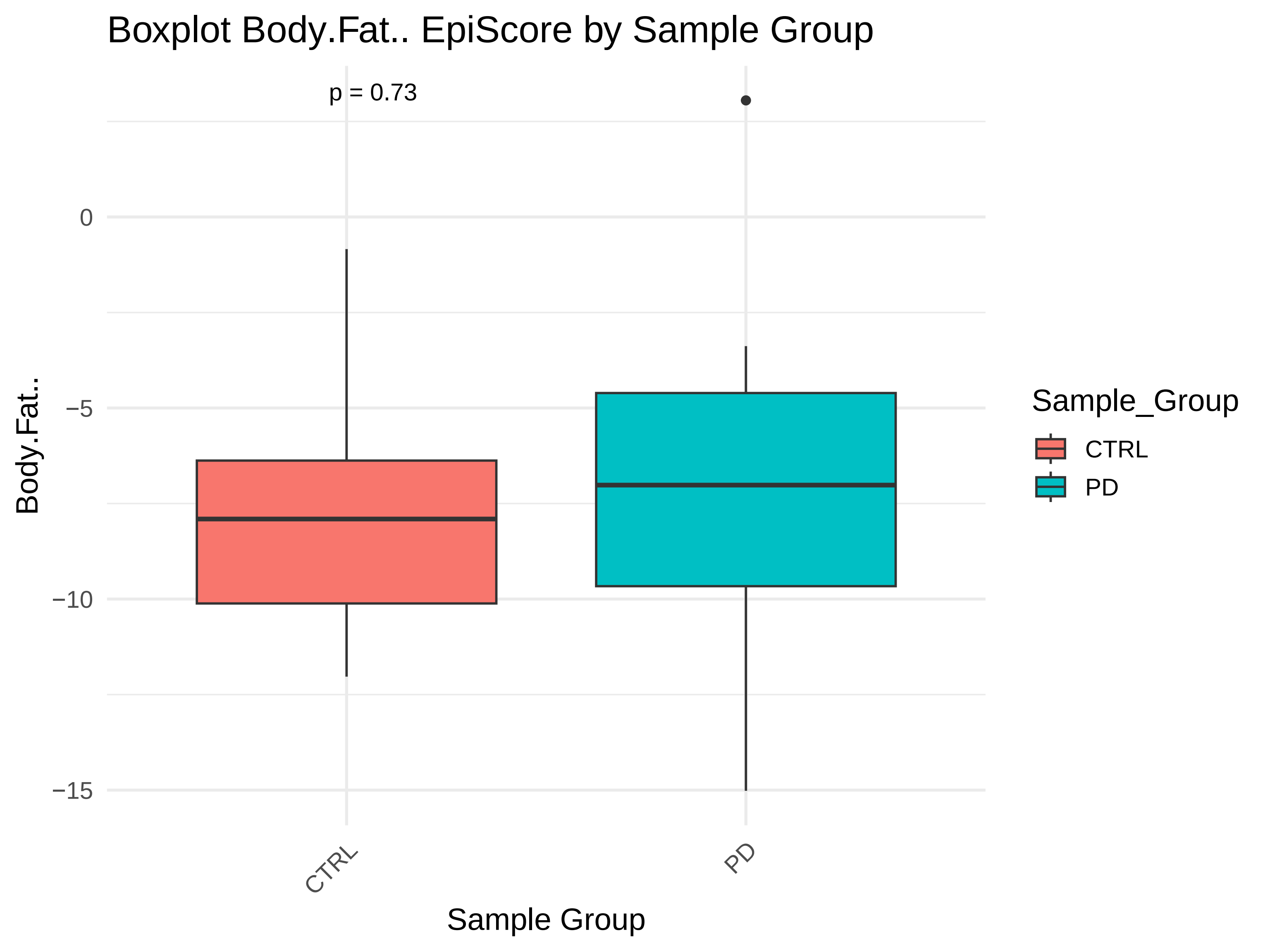
Boxplot Body.Fat.. EpiScore by Sample Group
p = 0.73
0
−5
−10
−15
Body.Fat..
CTRL
PD
Sample Group
Sample_Group
CTRL
PD

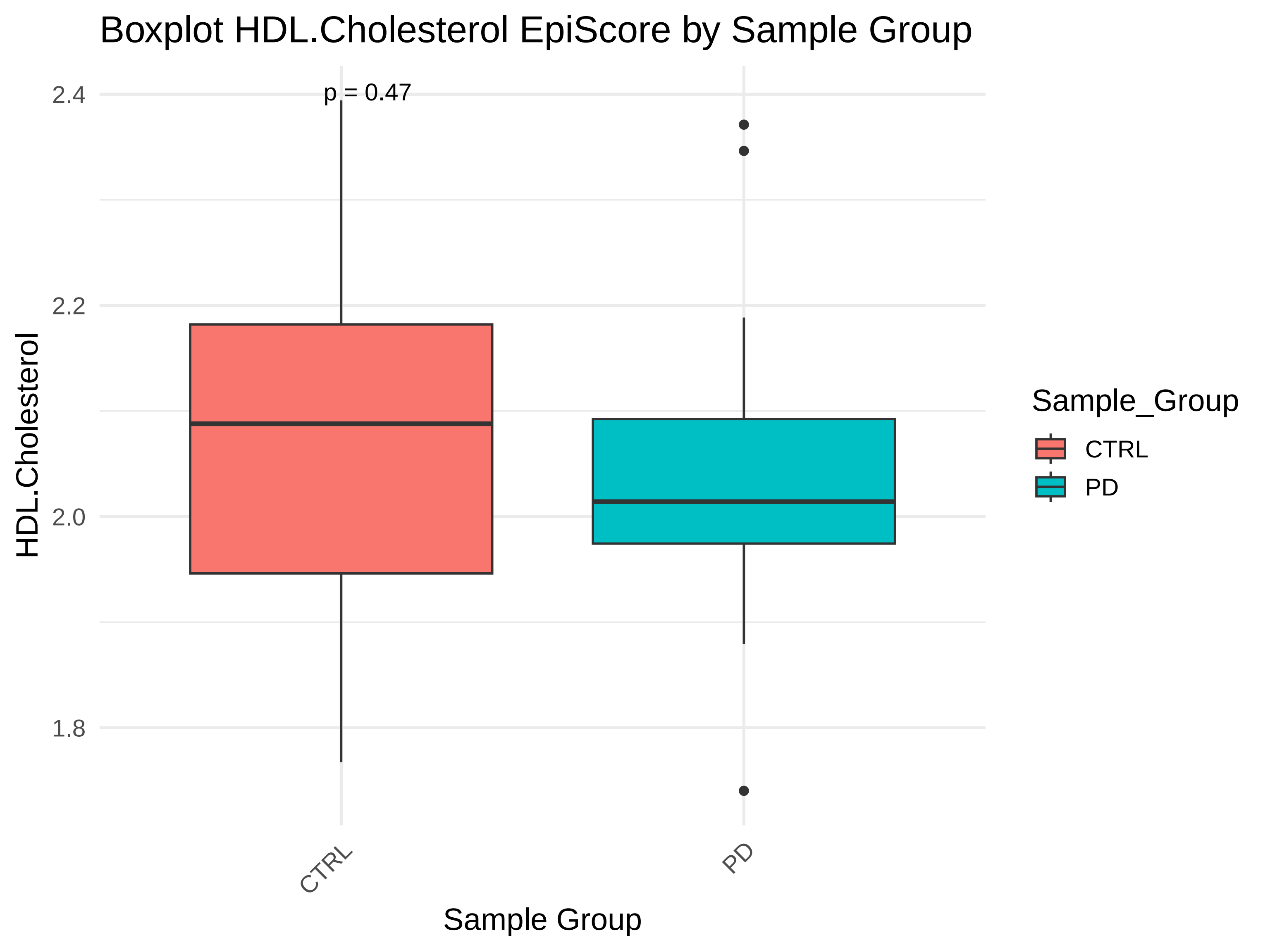
Boxplot HDL.Cholesterol EpiScore by Sample Group
p = 0.47
2.4
2.2
2.0
1.8
HDL.Cholesterol
CTRL
PD
Sample Group
Sample_Group
CTRL
PD

Figure S5. Boxplot of log(SEMs). Stochastic epigenetic mutations across group. p-value calculated from a linear regression model adjusted for age, sex and cell count.

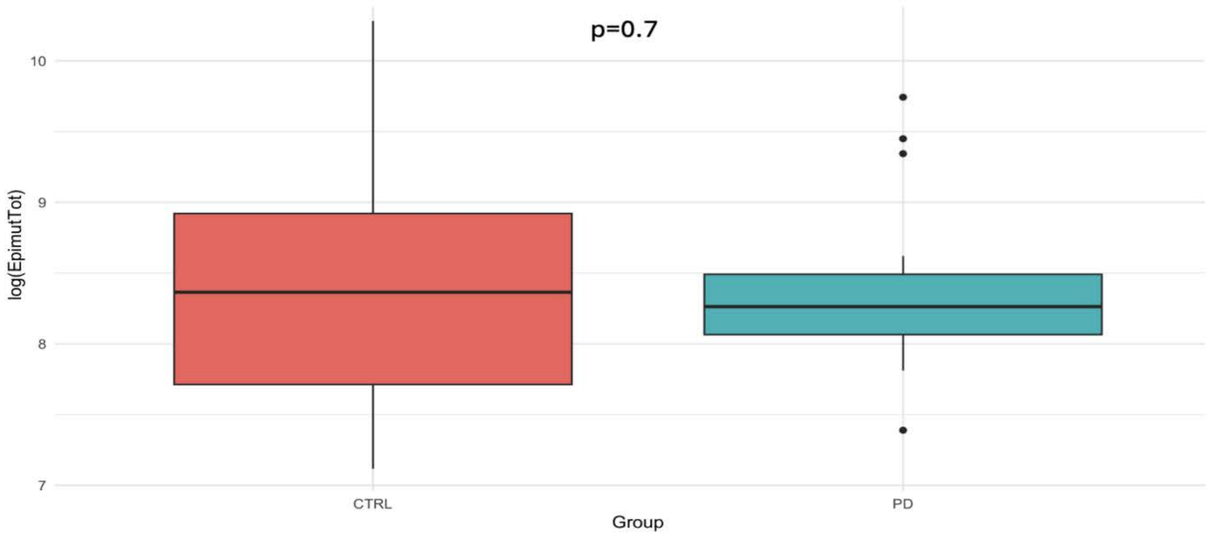

Figure S6. Heatmap of the 54cg DMP (threshold: adjusted p-value < 0.05 and Δβ > 0.05) annotated in unique genes.

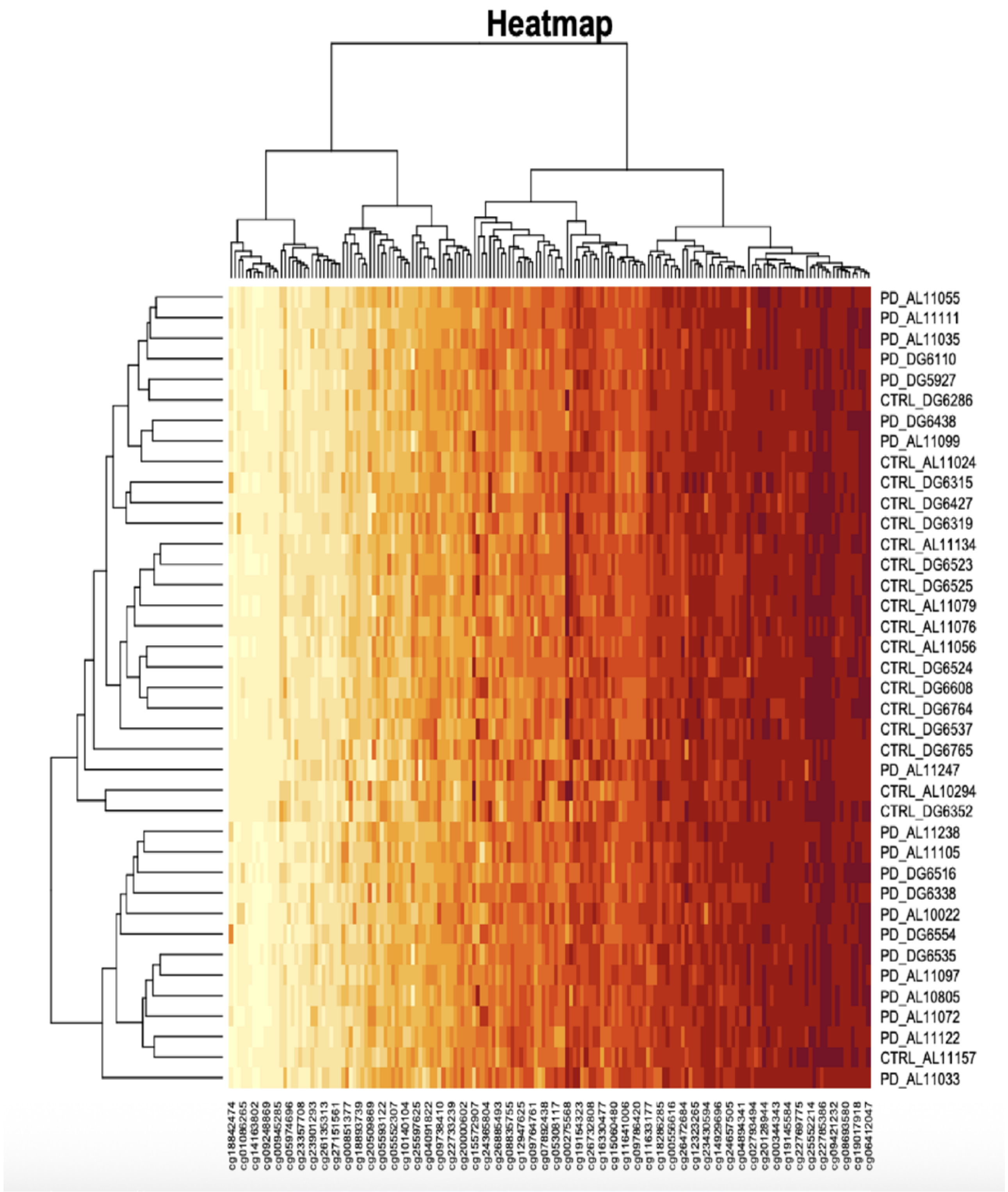

Figure S7. Methylation profile for the DMR annotated in CABP5 gene. The x axis is the genomic position of chromosome 19 (chr19:48044057_48044106, hg38). The y axis is the mean of beta value.

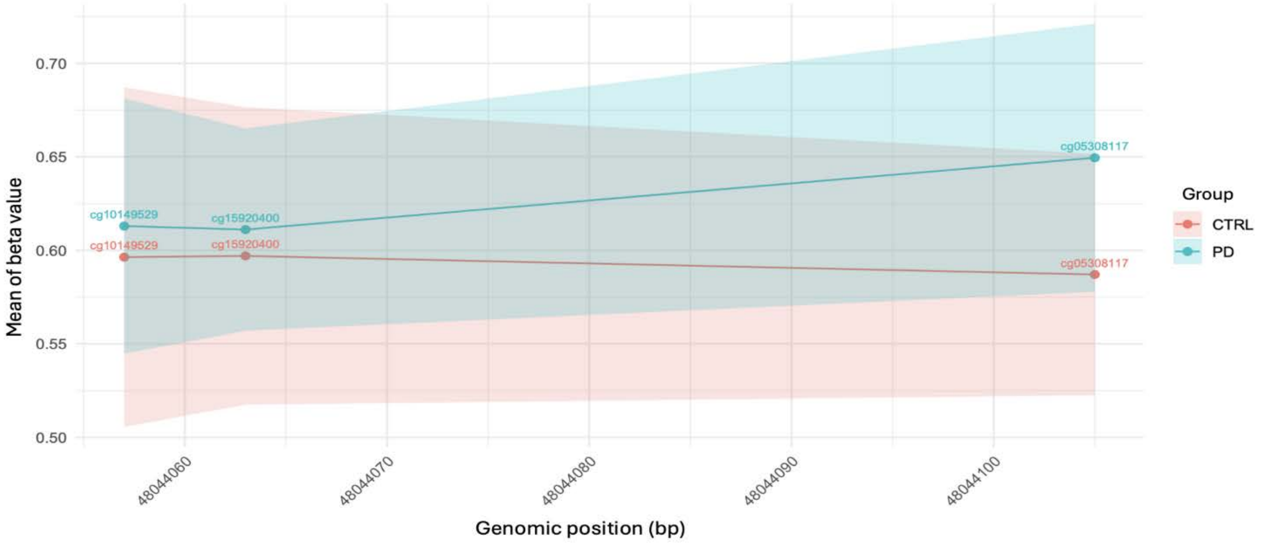

Table S1. Epivariations: list of genes with epivariations in CTRL and PD subjects. Gene: Gene name (annotation hg38); Meth: Methylation status (Down = hypomethylated, Up = hypermethylated). CTRL and PD: number of subjects with at least one epivariation in that gene.

Gene	Meth	CTRL	PD
GLRX3; MIR378C	Down	1	1
QRFPR; ANXA5	Down	1	0
RFLNB; VPS53	Down	1	1
TPM3	Down	1	0
TRIM26	Down	1	0
DYM	Down	0	1
LINC02103; LSP1P3	Down	0	1
MIR3159; LDHA	Down	0	1
SYVN1; SPDYC	Down	0	1
CLINT1; LINC02227	Down	0	1
EXOC2	Down	0	1
HUS1B	Down	0	1
ACSL5	Down	0	4
LINC01623; HCG14	Down	0	1
ERICH1	Down	0	3
LINC01782; ZNF507	Down	0	2
PGAP1	Down	0	1
BRDT	Down	0	1
BRDT; EPHX4	Down	0	1

Table S2. Pathway enrichment analysis of 54 genes annotated from 167 DMPs ($\Delta\beta > 0.05$). Only pathways with nominal $p < 0.08$ are shown. None of the pathways reached significance after multiple testing correction (FDR).

Gene Set	Description	Ratio	P Value	FDR
hsa04080	Neuroactive ligand-receptor interaction	4.42	0.0282	1
hsa01240	Biosynthesis of cofactors	7.06	0.0318	1
hsa00770	Pantothenate and CoA biosynthesis	25.73	0.0382	1
hsa05022	Pathways of neurodegeneration	3.40	0.0544	1
hsa00051	Fructose and mannose metabolism	15.89	0.0612	1
hsa04024	cAMP signaling pathway	4.80	0.0637	1
hsa00512	Mucin type O-glycan biosynthesis	15.01	0.0646	1
hsa01250	Biosynthesis of nucleotide sugars	14.60	0.0664	1
hsa05033	Nicotine addiction	13.51	0.0716	1
hsa04962	Vasopressin-regulated water reabsorption	12.28	0.0785	1
